# Genome-wide association for agro-morphological traits in a triploid banana population with large chromosome rearrangements

**DOI:** 10.1093/hr/uhae307

**Published:** 2024-11-06

**Authors:** Simon Rio, Lucile Toniutti, Frédéric Salmon, Catherine Hervouet, Céline Cardi, Pierre Mournet, Chantal Guiougou, Franck Marius, Claude Mina, Jean-Marie Eric Delos, Frédéric Lambert, Camille Madec, Jean-Claude Efile, Corinne Cruaud, Jean Marc Aury, Angélique D’Hont, Jean-Yves Hoarau, Guillaume Martin

**Affiliations:** CIRAD, UMR AGAP Institut, F-34398 Montpellier, France; AGAP Institut, CIRAD, INRAE, Institut Agro, Université de Montpellier, Montpellier, France; AGAP Institut, CIRAD, INRAE, Institut Agro, Université de Montpellier, Montpellier, France; CIRAD, UMR AGAP Institut, F-97130 Capesterre-Belle-Eau, Guadeloupe, France; AGAP Institut, CIRAD, INRAE, Institut Agro, Université de Montpellier, Montpellier, France; CIRAD, UMR AGAP Institut, F-97130 Capesterre-Belle-Eau, Guadeloupe, France; CIRAD, UMR AGAP Institut, F-34398 Montpellier, France; AGAP Institut, CIRAD, INRAE, Institut Agro, Université de Montpellier, Montpellier, France; CIRAD, UMR AGAP Institut, F-34398 Montpellier, France; AGAP Institut, CIRAD, INRAE, Institut Agro, Université de Montpellier, Montpellier, France; CIRAD, UMR AGAP Institut, F-34398 Montpellier, France; AGAP Institut, CIRAD, INRAE, Institut Agro, Université de Montpellier, Montpellier, France; AGAP Institut, CIRAD, INRAE, Institut Agro, Université de Montpellier, Montpellier, France; CIRAD, UMR AGAP Institut, F-97130 Capesterre-Belle-Eau, Guadeloupe, France; AGAP Institut, CIRAD, INRAE, Institut Agro, Université de Montpellier, Montpellier, France; CIRAD, UMR AGAP Institut, F-97130 Capesterre-Belle-Eau, Guadeloupe, France; AGAP Institut, CIRAD, INRAE, Institut Agro, Université de Montpellier, Montpellier, France; CIRAD, UMR AGAP Institut, F-97130 Capesterre-Belle-Eau, Guadeloupe, France; AGAP Institut, CIRAD, INRAE, Institut Agro, Université de Montpellier, Montpellier, France; CIRAD, UMR AGAP Institut, F-97130 Capesterre-Belle-Eau, Guadeloupe, France; AGAP Institut, CIRAD, INRAE, Institut Agro, Université de Montpellier, Montpellier, France; CIRAD, UMR AGAP Institut, F-97130 Capesterre-Belle-Eau, Guadeloupe, France; AGAP Institut, CIRAD, INRAE, Institut Agro, Université de Montpellier, Montpellier, France; CIRAD, UMR AGAP Institut, F-97130 Capesterre-Belle-Eau, Guadeloupe, France; AGAP Institut, CIRAD, INRAE, Institut Agro, Université de Montpellier, Montpellier, France; CIRAD, UMR AGAP Institut, F-97130 Capesterre-Belle-Eau, Guadeloupe, France; Génomique Métabolique, Genoscope, Institut François Jacob, CEA, CNRS, Univ Evry, Université Paris-Saclay, 91057 Evry, France; Génomique Métabolique, Genoscope, Institut François Jacob, CEA, CNRS, Univ Evry, Université Paris-Saclay, 91057 Evry, France; CIRAD, UMR AGAP Institut, F-34398 Montpellier, France; AGAP Institut, CIRAD, INRAE, Institut Agro, Université de Montpellier, Montpellier, France; AGAP Institut, CIRAD, INRAE, Institut Agro, Université de Montpellier, Montpellier, France; CIRAD, UMR AGAP Institut, F-97494 Sainte-Clotilde, La Réunion, France; CIRAD, UMR AGAP Institut, F-34398 Montpellier, France; AGAP Institut, CIRAD, INRAE, Institut Agro, Université de Montpellier, Montpellier, France

## Abstract

Banana breeding is hampered by the very low fertility of domesticated bananas and the lack of knowledge about the genetic determinism of agronomic traits. We analysed a breeding population of 2723 triploid hybrids resulting from crosses between diploid and tetraploid *Musa acuminata* parents, which was evaluated over three successive crop cycles for 24 traits relating to yield components and plant, bunch, and fruit architectures. A subset of 1129 individuals was genotyped by sequencing, revealing 205 612 single-nucleotide polymorphisms (SNPs). Most parents were heterozygous for one or several large reciprocal chromosomal translocations, which are known to impact recombination and chromosomal segregation. We applied two linear mixed models to detect associations between markers and traits: (i) a standard model with a kinship calculated using all SNPs and (ii) a model with chromosome-specific kinships that aims at recovering statistical power at alleles carried by long non-recombined haplotypic segments. For 23 of the 24 traits, we identified one to five significant quantitative trait loci (QTLs) for which the origin of favourable alleles could often be determined amongst the main ancestral contributors to banana cultivars. Several QTLs, located in the rearranged regions, were only detected using the second model. The resulting QTL landscape represents an important resource to support breeding programmes. The proposed strategy for recovering power at SNPs carried by long non-recombined rearranged haplotypic segments is an important methodological advance for future association studies in banana and other species affected by chromosomal rearrangements.

## Introduction

Dessert and cooking bananas (*Musa* spp.) are staple foods and an important source of income in many tropical and subtropical producing countries. There are about a thousand different banana cultivars, but the world banana production is based on a very limited number of natural hybrid cultivars and their somaclonal variants [1]. The ‘Cavendish’ bananas alone, which represent a few natural phenotypic somaclonal variants, account for ~57% of world banana production [2]. Such a narrow genetic base makes the world’s banana cultivation very vulnerable to the outbreak of diseases and pests, and variations caused by climate change or human practises. In this context, breeding for more diverse disease-resistant varieties that meet yield and quality commercial production criteria is essential for achieving sustainable banana production.

Cultivated bananas are natural hybrids between species and subspecies of the genus *Musa* initially selected in Southeast Asia [3–6]. One of the main selected traits of cultivated bananas has been their ability to produce edible seedless fleshy fruits, due to sterility and parthenocarpy [7, 8]. For banana, one way to achieve complete or almost complete sterility is through the production of triploid individuals (3x), a ploidy level that provides more vigorous plants with larger bunches than diploids [1]. A common breeding strategy for obtaining progenies of triploid individuals involves crossing a diploid parent (2x) with a tetraploid parent (4x) [1, 9–12]. The tetraploid parents are doubled diploid accessions obtained from a colchicine treatment or selected from crosses between triploid and diploid parents. Alongside this cross-breeding step leading to the selection of commercial triploid hybrids, a recurrent breeding step involving genetic improvement of diploid parents can be carried out. This strategy of improving parents through cycles of recombination and selection is likely to facilitate the simultaneous improvement of a larger number of agronomic traits of interest. However, banana breeding remains difficult as the most interesting banana progenitors have very low levels of fertility and germination rates, requiring embryo rescue. In addition, selecting triploid hybrids in the field requires a lot of space and time, given the large plant biomass and the relatively long cultivation cycles. In this context, knowledge about the genetic architecture of the main target agronomic traits could greatly help choosing the best resources and crossing schemes to accelerate the production of new cultivars.

Genome-wide association studies (GWAS) have been successfully applied in numerous crop species to identify quantitative trait loci (QTLs) controlling a wide range of agronomic and biochemical traits [see Gupta *et al.* [13] for a review]. These studies exploit linkage disequilibrium (LD) between single-nucleotide polymorphisms (SNPs) and causal variants at QTLs. Regarding banana, very few QTLs have been detected using GWAS approaches and for a limited number of traits: seedless phenotype [14], bunch weight, and its morphological components [15]. QTLs for organoleptic fruit quality during banana ripening [16] and resistance to subtropical race 4 of *Fusarium oxysporum* f. sp. *cubense* [17] have also been identified using QTL mapping.

A common issue in GWAS is controlling the detection of spurious associations caused by population structure, which generates LD between loci not necessarily physically linked. The most common way of limiting these false-positive associations is to take into account genetic structure or kinship amongst individuals in the model [18]. A drawback of this standard approach is that it limits statistical power (i.e. the probability of detecting true signals) in genomic regions with a large extent of LD. This is due to the fact that markers are used both for testing associations and estimating kinship. Rincent *et al.* [19] proposed a method for efficiently recovering statistical power in regions with large extent of LD, in which SNPs present on the same chromosome as the tested SNP are discarded to estimate kinship.

Banana cultivar genomes are a mosaic of ancestral contributions [6, 20–22]. Some of the contributing species and subspecies differ by a few large chromosomal rearrangements, mainly large reciprocal translocations, sometimes associated with inversions [23–28], resulting in structural heterozygosities in hybrid cultivars. So far, zero to four large chromosome rearrangements have been observed in the genome of cultivars (https://banana-genome-hub.southgreen.fr/translocation). These structural heterozygosities generated segregation distortions and the inversions prevented recombination [20, 23, 25, 26]. In the QTL mapping study reported by Biabiany *et al.* [16], the presence of a large structural heterozygosity in one parent—resulting from a reciprocal translocation between chromosome 1 and 7 associated with an inversion—blocked recombination along chromosome 1 and generated cosegregation between chromosome 1 and 7. This cosegregation prevented the precise location of the fruit quality QTLs. The consequences of these structural heterozygosities have not yet been assessed in QTL banana studies based on GWAS approaches.

In this work, we analysed a large breeding population of 2723 triploid hybrids from CIRAD’s banana varietal improvement programme [11, 29]. This triploid population was bred from representative *Musa acuminata* accessions containing some large chromosomal rearrangements. They were phenotyped for 24 agro-morphological traits of breeding interest relating to yield components as well as plant, bunch, and fruit architectures. The objectives of the study were to i) evaluate the impact of large chromosome rearrangements on QTL detection, ii) propose a new GWAS model to limit their negative impact on the ability to detect QTL, and iii) obtain an extensive overview of the QTL landscape for the traits in the *M. acuminata* resources studied.

## Results

In this study, we analysed a large breeding population of 2723 triploid banana hybrids evaluated for 24 traits relating to yield components as well as plant, bunch, and fruit architectures (Table 1). A subset of 1129 hybrids was genotyped for 205 612 polymorphic bi-allelic SNPs and used for GWAS.

**Table 1:** Description of traits.

Category	Description	Unit
Plant architecture	Pseudostem height (PH) - Measured at flowering	cm
Plant architecture	Pseudostem girth (PG) - Measured at 1 m above soil level (at flowering?)	cm
Plant architecture	Robustness index (PH/PG) - Robustness of the pseudostem	
Plant architecture	Number of leaves at flowering - Counted on standing leaves	
Plant architecture	Number of leaves at harvesting - Counted on standing leaves	
Plant architecture	Leaf blade length (LL) - Measured on rank 3 leaf	cm
Plant architecture	Leaf blade width (LW) - Measured on rank 3 leaf	cm
Plant architecture	Leaf index (LL/LW)	
Bunch architecture	Bunch angle - Angle between the bunch and the pseudostem	°
Bunch architecture	Peduncle length (PL)	cm
Bunch architecture	Peduncle diameter (PD)	cm
Bunch architecture	Peduncle index (PL/PD)	
Bunch architecture	Bunch length at maturity (BL) - Measured at maturity	cm
Bunch architecture	Bunch compactness index (BL/NH)	cm
Fruit architecture	Fruit pedicel length	mm
Fruit architecture	Fruit pedicel diameter	mm
Fruit architecture	Fruit length	mm
Fruit architecture	Fruit grade	mm
Yield component	Number of hands on a bunch (NH) - Counted on a bunch	
Yield component	Number of fruits on a bunch (NF) - Counted on a bunch	
Yield component	Number of fruits per hand (NF/NH)	
Yield component	Bunch weight - Measured at maturity	kg
Yield component	Fruit weight - Measured at maturity	g
Yield component	Days to fruit maturity - Interval between flowering and harvesting	days

### Impact of large chromosome rearrangements on population structure

**Figure 1:**
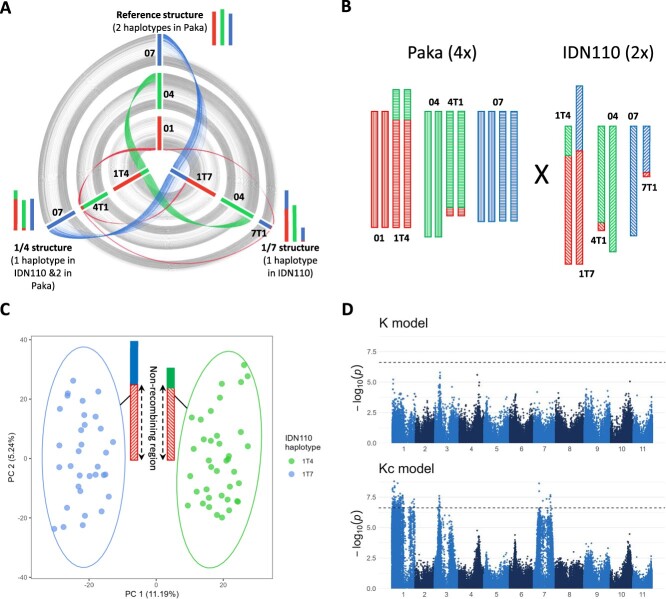
Impact of large chromosome rearrangements on GWAS. **A**) Comparison of two reciprocal translocations (1/4 and 1/7) involving chromosomes 1, 4, and 7 with the reference chromosome structure. **B**) Chromosome structural heterozygosities in the tetraploid Paka (4x) and in the diploid IDN110 (2x) accessions. **C**) SNP-based principal component analysis performed on the 71 genotypes of the Paka (4x) × IDN110 (2x) population. **D**) Manhattan plots obtained for bunch angle from a standard model (K model) compared to the model proposed to recover signals (Kc model)

Hybrids resulted from crosses between diploid and tetraploid *M. acuminata* parents, most of which were heterozygous for one to three large reciprocal translocations (Table 2). These translocations involved four couples of chromosomes: 1/4, 1/7, 2/8, and 3/8. Parents heterozygous for translocations 1/4, 1/7, and 3/8 display absence or reduction of recombination involving large chromosome segments, whilst for translocations 2/8 a reduction of recombination is observed only at the breakpoints [26]. Moreover, some chromosomes are involved in distinct translocations. For example, chromosome 1 is involved in three distinct chromosome structures: the reference chromosome structure, the 1/4 reciprocal translocation (1T4 and 4T1 haplotypes), and the 1/7 reciprocal translocation (1T7, 7T1 haplotypes) (Fig. 1a).

**Table 2:** Diploid and tetraploid parents of the 1129 hybrids used in the GWAS.

	Number of crosses		Large reciprocal translocations			
Parents	Diploid	Tetraploid	1/4	1/7	2/8	3/8
Akondro Mainty^a^	0	5	heterozygous	−	−	heterozygous
Chicame^a^	0	8	heterozygous	−	−	heterozygous
Gu Nin Chiao^b^	1	0	heterozygous	heterozygous	−	−
Khi Maeo^c^	3	0	heterozygous	heterozygous	−	heterozygous
IDN 077	1	1	heterozygous	heterozygous	−	−
IDN 110^b^	3	5	heterozygous	heterozygous	−	−
IRFA 903^b^	0	2	heterozygous	heterozygous	−	−
Malaccensis nain	3	0	homozygous	−	−	−
Manang	3	1	−	heterozygous	heterozygous	−
Microcarpa	1	0	−	−	−	heterozygous
Nzumoheli II^a^	0	1	heterozygous	−	−	heterozygous
Pa (Patthalong)^c^	2	0	heterozygous	heterozygous	−	heterozygous
Pahang	2	0	heterozygous	−	−	−
Paka	2	6	heterozygous	−	−	−
Pisang Jaran	2	0	−	heterozygous	−	heterozygous
Pisang Lilin	1	4	heterozygous	−	−	−
Pisang Madu	7	2	−	heterozygous	−	−
Pisang Pipit	0	3	−	heterozygous	−	heterozygous
Sinwobogi	2	0	−	−	−	−
THA 052^c^	3	0	heterozygous	heterozygous	−	heterozygous
Thong Det	2	0	−	heterozygous	−	−

Presence of large reciprocal translocation in heterozygous or homozygous states or absence (−) involving 4 couples of chromosomes: 1/4, 1/7, 2/8, and 3/8.

a are groups of somaclones

b are groups of somaclones

c are groups of somaclones

To evaluate the impact of distinct chromosome structures on the estimation of population structure, we exploited a progeny from the cross Paka (4x) × IDN110 (2x). Paka has two copies of the reference chromosome 1 and two copies of the 1/4 translocated chromosomes (1T4 and 4T1), whilst IDN110 has one copy of the 1/4 translocated chromosomes and one copy of the 1/7 translocated chromosomes (1T7 and 7T1) (Fig. 1b). A principal component analysis performed with the SNP data (Fig. 1c) clustered the hybrids of the Paka (4x) × IDN110 (2x) progeny into two groups. These groups corresponded to the presence of the 1T4 haplotype or the 1T7 haplotype inherited from IDN110, presenting an absence of recombination on the chromosome 1 portion of these haplotypes. This illustrates the impact that large chromosomal rearrangements can have on population structure.

### Accounting for population structure generated by large chromosomal rearrangements in GWAS

In standard GWAS models, a polygenic background effect is generally included whose covariance is proportional to a kinship matrix calculated with SNPs. The aim is to control for false positives by limiting statistical power at SNPs whose polymorphism is correlated with population structure. As a consequence, the statistical power at SNPs tagging the segregation of the 1T4 and 1T7 haplotypes from IDN110 was also limited when applying the standard K model. We circumvented this problem by proposing an alternative GWAS model, the Kc model, in which a kinship is calculated specifically for each chromosome carrying the SNPs to be tested, and by excluding SNPs from that chromosome and from other chromosomes involved in structural variation with it. The added value provided by this new GWAS methodology can be exemplified by the analysis of the bunch angle. For this trait, the standard K model did not reveal any significant associations for a 5% Bonferroni threshold, whilst the new Kc model helped to recover signals on chromosomes 1, 3, and 7 (Fig. 1d). The associations on chromosomes 1 and 7 likely identified a QTL with an allele located on a 1T7 haplotype that did not recombine in the population, such as that carried by IND110. The Kc model also helped recovering signals on chromosome 3 that may have been hidden in the K model GWAS due to limited recombination of rearranged 3/8 haplotypes in other crosses, the 3/8 reciprocal translocation being absent in Paka and IDN110.

### QTL detection for agro-morphological traits

**Figure 2:**
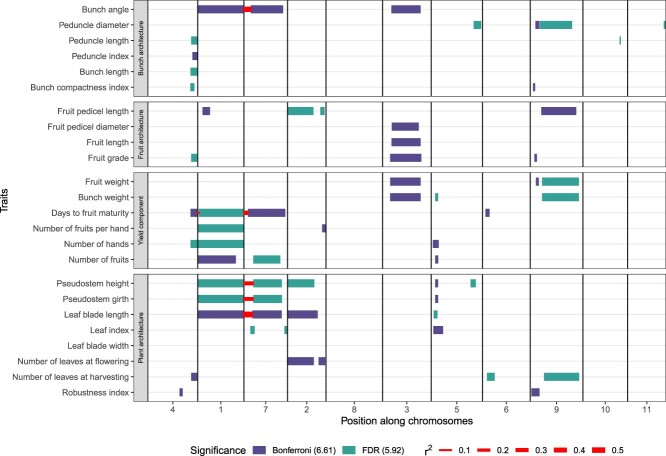
Localization of consensus QTL LD intervals along chromosomes according to two significance thresholds: Bonferroni (−log_10_(p) = 6.61) and FDR (−log_10_(p) = 5.92). Cosegregations between the most significant SNPs of each interval are indicated by a segment with a width proportional to their LD (r^2^) whose values are shown in Table S2. The continuous r^2^ size scale is represented by discrete values from 0.1 to 0.5. The order of the chromosomes on the x-axis was chosen so as to position the chromosomes involved in a reciprocal translocation close to each other.

The moderate to high heritability estimated for the 24 traits over the experimental design confirmed the relevance of this dataset to perform GWAS (Table S1). Both K and Kc GWAS models were applied to all traits and SNPs (see summary statistics in Dataverse repository, QQ-plots, and Manhattan plots in Figs. S1 and S2 for the K and Kc models, respectively) with two significance thresholds, i.e. a 5% Bonferroni threshold and a 5% false discovery rate (FDR). Significant associations were clustered into 62 consensus QTLs over the two models, and LD intervals were calculated between the most significant SNP of the QTL and neighbouring SNPs (Fig. 2). Cosegregation between QTLs located on different chromosomes was identified and could always be related to the presence of the 1/7 and 1/4 translocations (Table S2). Regarding the comparison of GWAS models, a same number of 43 QTLs was detected for each model considering a 5% FDR, but a higher number of QTLs was detected using the Kc model [6] than using the K model [2] considering a 5% Bonferroni threshold. A comparison of QTL LD intervals according to the model is presented in Fig. S3 and information on each QTL is presented in Table S3.

### QTL allele ancestries

**Figure 3:**
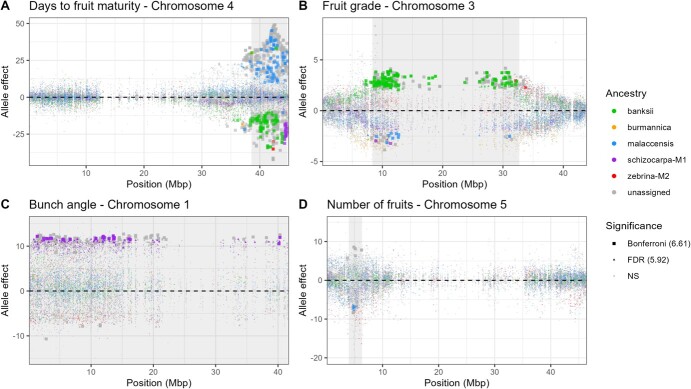
Estimated allele effects for (**A**) days to fruit maturity QTL on chromosome 4, (**B**) fruit grade QTL on chromosome 3, (**C**) bunch angle QTL on chromosome 1, and (**D**) number of fruits QTL on chromosome 5. The plotted effects were obtained from the Kc model, except for number of fruits for which the effects were obtained from the K model. Dots were coloured according to allele ancestry and shaped according to the level of significance of the test. When no ancestry could be assigned, the effect represented was that of the alternative allele. The QTL interval is indicated by a grey area.

The determination of the ancestral origin of some of the SNP alleles made it possible to characterize the origin of the favourable and unfavourable alleles of part of the QTLs detected in this study. A focus was done on four QTLs involved in the genetic determinism of days to fruit maturity, fruit grade, bunch angle, and number of fruits (Fig. 3). Figures representing the characterization of the allele ancestry of each QTL are available in Fig. S4 and S5 for the K and Kc model, respectively.

For days to fruit maturity (Fig. 3a), a QTL has been detected with both models at the end of chromosome 4, with an LD interval ranging from 38.55 to 44.72 Mbp. As cycle length is one of the components of banana yield due to the asynchronism of growth cycle between different plants, shortening the interval between flowering and harvesting is of interest from a breeding perspective. As a consequence, the effects associated with the presence of favourable alleles have a negative sign. Most favourable alleles showed banksi ancestry, except for two zebrina-M2 alleles as well as a large number of schizocarpa-M1 alleles at the end of the LD interval. In contrast, most unfavourable alleles showed malaccensis ancestry, with the exception of two banski alleles.

For fruit grade (Fig. 3b), a QTL has been detected with both models around the centromere of chromosome 3, with a large LD interval ranging from 8.33 to 32.70 Mbp. Provided that a breeder seeks to increase fruit diameter, favourable alleles have a positive sign. Again, most favourable alleles showed banksii ancestry, with the exception of a zebrina-M2 allele at the end of the interval. In contrast, unfavourable alleles showed malaccensis or schizocarpa-M1 ancestry.

For bunch angle (Fig. 3c), a QTL has been detected with the Kc model only and spans the entire chromosome 1. From a breeding perspective, the smallest angle between the bunch and the pseudostem is generally desirable in order to harvest fruits of uniform dimension from the bunch. Most alleles with positive signs had schizocarpa-M1 ancestry with few exceptions including burmannica, malaccensis, and zebrina-M2 ancestry. No allele whose presence is associated with an effect of negative sign (favourable alleles) could be assigned to an ancestry. Note that this QTL cosegregated with a QTL on chromosome 7 as a result of the segregation of rearranged 1/7 haplotypes, and this cosegregating QTL showed a similar ancestry pattern with several schizocarpa-M1 favourable alleles (Fig. S5).

Finally, for number of fruits (Fig. 3d), a QTL has been detected with both models at the beginning of chromosome 5, with an LD interval ranging from 3.98 to 6.34 Mbp. Few alleles with negative signs (unfavourable alleles for bunch weight) suggested a malaccensis ancestry. No allele whose presence is associated with an effect of positive sign could be assigned.

### Meta-analysis

**Figure 4:**
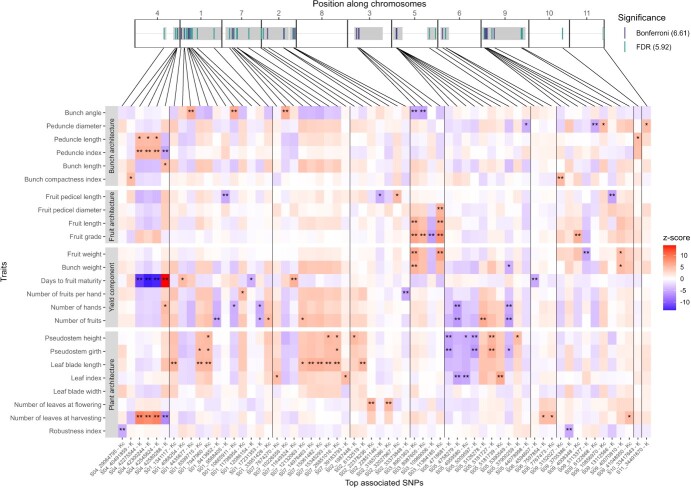
QTL meta-analysis. For each QTL, the effect of the most associated SNP in the interval was transformed into a z-score using the estimate obtained from the model for which it was most significant. The effect considered was that of the allele alternative to the reference genome. The model was reported after each SNP name on the x-axis and the level of significance is indicated using ‘*’ and ‘**’ if the SNP was detected using FDR or both FDR and Bonferroni, respectively. The order of the chromosomes on the x-axis was chosen so as to position the chromosomes involved in a reciprocal translocation close to each other.

Based on Fig. 2, some QTLs detected for different traits colocalized to the same genomic regions. They may result from a single causal locus with pleiotropic effects on traits. To investigate the existence of such effects, we performed a meta-analysis using the most associated SNPs of all QTLs by transforming the effect of alleles alternative to those of the reference genome into z-scores (Fig. 4). Note that colocalizing QTLs did not necessarily have the same most significant SNP. Three sets of colocalized QTLs with a high level of significance for several traits are described hereafter.

A first set of colocalized QTLs involved peduncle length, peduncle index, bunch length, bunch compactness index, days to fruit maturity, number of hands, and number of leaves at harvesting, for which at least one of the following SNPs located at the end of chromosome 4 was significantly detected: S04_40491859, S04_42275544, S04_42300244, S04_42545624, and S04_42589288. For instance, the presence of the alternative allele of SNP S04_42545624 was significantly associated with a decrease in days to fruit maturity but an increase in number of leaves at harvesting, peduncle length, and peduncle index. In contrast, the alternative allele of SNP S04_42589288 showed opposite effect signs compared to SNP S04_42545624 for these same traits. This opposition of signs was not simply due to the arbitrary coding of the alleles of the two SNPs (based on the reference genome), as both SNPs showed similar genotypic classes frequencies (see Table S3). It rather resulted from the existence of two parental haplotypes with contrasted effects segregating in some crosses, each haplotype being tagged by different SNPs.

A second set of colocalizing QTLs involved bunch angle, fruit pedicel diameter, fruit length, fruit grade, fruit weight, and bunch weight, for which at least one of the following SNPs located on chromosome 3 was significantly detected: S03_8901363, S03_8987605, S03_10969006, and S03_11364185. For instance, the presence of the alternative allele of S03_8901363 was significantly associated with an increase in fruit length, fruit grade, fruit weight, and bunch weight but a decrease in bunch angle.

A last set of colocalizing QTLs involved number of hands, number of fruits, pseudostem height, pseudostem girth, and leaf index, for which at least one of the following SNPs located on chromosome 5 was significantly detected: S05_4718681, S05_4758279, S05_4959580, S05_5009597, S05_5126278, S05_5181727, S05_5181739, and S05_5390549. Whilst none of these SNPs were significant for all traits, the sign of the effect associated with the presence of the alternative allele was consistent across all traits.

## Discussion

### Accounting for large chromosome rearrangements in GWAS

Large chromosome rearrangements at the heterozygous state in accessions disrupt recombination and segregation during the meiosis [30–33]. In banana, Martin *et al.* [26] reported the presence of several reciprocal translocations, sometimes associated with inversions, in the various genetic groups involved in cultivars. They also showed that some of these translocations at heterozygous state in parents led to haplotype segments showing an absence or a reduction of recombination and/or cosegregations with other haplotype segments. At the scale of our multiparental banana population involving such parents, limited recombination of haplotype segments generated large blocks of markers in LD. The transmission of non-recombined rearranged haplotypes was associated with population structure in the progeny. This genetic structuring of the progeny could theoretically translate into phenotypic structuring provided that the non-recombined haplotypes carry one or more QTLs with strong effects.

Because the markers involved in such haplotypes were used both for testing associations with traits and estimating kinship, they were found to correlate with the population structure, which limited their statistical detection power in the GWAS mixed model. To overcome this limitation, we followed the method of Rincent *et al.* [19] by proposing the Kc model in which a kinship is calculated with all markers, excluding those located on the same chromosome as the marker tested. We adapted the original method by also excluding the markers that are located on chromosomes involved in a network of reciprocal translocations (e.g. chromosomes 1, 4, and 7 for the 1/4 and 1/7 reciprocal translocations). In our population, this strategy was supported by the existence of LD between markers located on different chromosomes according to the reference genome structure, but located on a same chromosome in rearranged chromosomes.

The Kc model helped recover several QTLs when compared to the standard K model that considered a kinship estimated using all SNPs, especially on chromosomes 1 and 7. The 1/7 alternative chromosome structures segregated in a large proportion of crosses, which is known to be associated with suppressed recombination on a large portion of the 1T7 haplotype [26]. Because of the limitation of statistical power mentioned here above, any QTL allele specifically present on the 1T7 haplotype would be particularly difficult to detect using a standard K model. Conversely, some QTLs were only detected with the K model. This could be explained by the procedure of correcting the Wald statistics using the inflation factor $\lambda$, which penalized more the Kc model than the K model. This stronger penalty for the Kc model probably resulted from the exclusion of all markers from certain chromosomes, which may have limited the accuracy of kinship estimation. We recommend applying both K and Kc models jointly to the data and aggregate results to obtain the most complete QTL landscape for the studied traits.

Because chromosome rearrangements in the heterozygous state are pervasive in the progenitors of banana breeding programmes, it seems important to apply our methodology to future GWAS in banana, so as not to miss out on detecting part of the QTL landscape.

### GWAS design for triploid banana populations

This triploid population, resulting from crosses between tetraploid (doubled-diploid) and diploid parents, was suboptimal for QTL detection with respect to the statistical properties of segregating markers. For a tetraploid parent with genotype 0:0:1:1, the segregation of its markers assuming polysomy at meiosis gives the following gametes: 0:0 (1/6), 0:1 (4/6), and 1:1 (1/6). When crossed to a diploid parent giving a single allele, e.g. 0, it generates three genotypic classes with the same frequencies as the gametes mentioned above. With the diploid SNP coding used in this study, this is equivalent to observing two genotypic classes (i.e. homozygous 0:0 and heterozygous 0:1), one of which is rare. It has been demonstrated that the existence of rare genotypic classes is associated with poor statistical power in GWAS [34]. Even supposing a triploid SNP coding, two-thirds of the progeny would be grouped into a single genotypic class (i.e. 0:0:1), thus recovering only a modest amount of statistical power. Note that assuming preferential pairing between doubled/identical chromosomes at meiosis in the tetraploid parent, the statistical power would decrease further with increasing frequency of 0:1 gametes. Alternatively, the segregation of markers in diploid parents allow for balanced genotypic classes in the progeny, which should allow for optimal power for detecting QTLs. In the future, we may consider generating crosses between diploid parents for the detection of QTLs and use triploid populations for validation only. However, it should be noted that the studied triploid population offered the advantage of detecting and evaluating directly the effect of QTLs in a triploid genetic background, which is that of most cultivars. In addition, it allowed to exploit the phenotyping effort that had already been carried out as part of CIRAD’s banana breeding programme, providing a large population phenotyped for several traits.

The choice of diploid coding for a triploid population for this analysis can be questioned. This choice was motivated by two reasons: (i) the genotyping-by-sequencing (GBS) approach does not allow the two heterozygous classes (0:0:1 and 0:1:1) to be easily distinguished; and (ii) the segregation of tetraploid parents produces a non-negligible proportion of aneuploid individuals, whose proportion is only increased by the presence of structural variations [20]. These two phenomena together mean that attempting to predict dosage in a triploid will produce a non-negligible number of errors. In this context, it seemed safer to genotype individuals only for their homozygous/heterozygous state.

The GWAS implemented in this study relied on hybrid genotypic values estimated over three growth cycles, which amounts to detecting QTLs with a relatively stable effect over all cycles. These QTL are of priority interest for breeding, as bananas are generally grown over many cycles. Any detection of QTL effects specific to particular cycles would require a multicycle GWAS including an interaction effect between the tested marker and the cycle. However, unlike in our study, replicates of each hybrid would be required to correctly estimate the cycle-specific genotypic values to be used as response variables in the multicycle GWAS. In addition, it would be interesting to evaluate each hybrid in several environments to distinguish the QTLs with a stable effect in all environments from those that interact with the environment.

### Genetic architecture of agro-morphological traits

In this study, a set of 62 consensus QTLs was detected for 23 of 24 traits, which were located on 10 of the 11 chromosomes of the banana genome. These results consist of the second GWAS results for yield components and fruit size (bunch weight, number of hands and fruits, fruit length and diameter) after Nyine *et al.* [15], and the first GWAS results for all other traits related to plant architecture and bunch architecture. Compared with this first study, the size of the population we studied was much larger (almost four times) and the density of SNP markers was also much higher (more than seven times). This greater experimental input has made it possible to increase the power of QTL detection for the traits shared between the two studies relating to yield components and fruit size. For these traits, Nyine *et al.* [15] detected 25 genomic loci mostly localized on chromosome 3. In our study, for these same traits, we also identified a large genomic region around the centromere of this chromosome 3 with significant QTL signals. In addition, the QTL landscape obtained for all 24 traits shows QTLs spread across all but one chromosome in the genome (chromosome 8). QTLs were detected for most traits, with one to five significant QTL detected for each trait, with the exception of leaf blade width for which no QTL were detected.

This relatively modest number of significant QTL per trait and their relatively modest effects suggested that variation of most traits in the studied population is controlled by many other genetic factors not detectable (due to small effect and/or unbalanced genotypic classes, causal loci in low LD with our SNPs, non-additive genetic effects). Most traits appeared essentially quantitative in nature. In general, QTLs displayed a pleiotropic effect on different traits, which is known to cause genetic correlations between traits (Falconer and Mackay, 1996). Such correlations between banana agro-morphological traits have already been reported by Nyine *et al.* [35] using a population from an East African highland banana breeding programme.

The QTL associated with bunch angle, bunch weight, and four of its components relative to fruit dimension (fruit weight, fruit length, fruit grade, and fruit pedicel diameter) on chromosome 3 is a first example of connected genetic architecture between traits. Bunch weight and its fruit components showed a QTL effect of the same sign, whilst the QTL effect of bunch angle was of the opposite sign. This QTL probably corresponded to the QTL detected by Nyine *et al.* [15] for bunch weight component traits. Based on the allele ancestry assignments of Martin *et al.* [6], we highlighted alleles of banksii origin associated with increased values for bunch weight and its fruit components and with decreasing values for bunch angle (Figs. 3b, S4 and S5). This pleiotropic effect of opposite sign between bunch weight and its fruit dimensions, on the one hand, and bunch angle with the pseudo-stem, on the other, is congruent with the fact that heaviest-bunch cultivars tend to have pendulous orientation whilst the smallest-bunch cultivars tend to have subhorizontal inflorescences [36]. It is conceivable that this QTL has a direct positive effect on bunch weight, which leads to greater bending of the peduncle, resulting in a smaller angle between the bunch and the pseudostem.

A second example consists of the QTL at the end of chromosome 4, which is associated with several traits and whose effect is particularly large for days to fruit maturity and number of leaves at harvesting, but with opposite sign. The negative relationship between these two traits can be explained by the fact that leaf emission stops after flowering and leaves are more likely to disappear when the interval between flowering and harvest increases due to senescence, wind damage, or diseases. From a breeding perspective, a short interval between flowering and harvesting is of interest, as it increases the number of cycles in a given period. Again, we showed that the favourable allele for this QTL had essentially a banksii origin.

A last example is the QTL at the beginning of chromosome 5, which is notably associated with the number of hands and fruits per bunch and pseudostem height and girth, with the same sign of effect. Whilst a greater number of fruits is desirable to reach higher yields, taller plants are undesirable because of their vulnerability to lodging. The allele statistically associated with smaller plants and a smaller number of fruits had a malaccensis origin.

Because of the multiparental nature of the population, QTL intervals were too wide to identify candidate genes. They covered >10 Mbp when they were located close to centromeres or when they tagged haplotypes whose recombination was limited by chromosomal rearrangements. As a result, the number of genes annotated from the *M. acuminata* reference sequence V4 [37] in each QTL interval was very large, ranging from 169 to 2743. Further work is needed to reduce the size of these intervals to be able to identify candidate genes.

### New perspectives for banana breeding

Based on these QTLs, it is possible to suggest ways of improving banana breeding schemes, which have so far made little use of the genomic information.

Firstly, parents can be characterized at QTLs so that crosses between parents carrying favourable alleles can be prioritized. These cross-choices could be done amongst the set of *M. acuminata* parents currently available in the breeding programme, as well as future improved parents obtained from recurrent parental selection.

Secondly, an early selection of progeny could be made prior to field evaluation based on their genotype at QTLs. This could enable a larger set of promising hybrids to be evaluated during the first phase of field evaluation. However, for quantitative traits, marker-assisted selection (MAS) has often proved disappointing [38], which can be explained by the insufficient proportion of the genetic variance explained by detected QTLs. Genomic prediction has often proved to be a more promising strategy than MAS [39–41], as it is not limited by statistical power associated with QTL detection. In banana, genomic prediction has already been evaluated for agro-morphological traits with moderate to high prediction accuracies [10], confirming the interest of this approach.

The characterization of allele origin has enabled us to identify the ancestral groups that have contributed numerous QTL favourable alleles. The most striking example is the banskii group, which has contributed the favourable allele for the bunch and fruit weight QTL as well as for the QTL allele associated with shorter cycle length. These results confirm the major role of the banksii group in the formation of dessert banana cultivars and suggest that particular attention should be paid to germplasm-carrying alleles of banksii origin.

The disruption of recombination generated by large chromosome rearrangements in heterozygous state in parents needs careful consideration in breeding. Both favourable and unfavourable alleles may cosegregate due to their localization on non-recombined haplotypic segments. This situation of genetic load makes it difficult to take advantage of the potential genetic variability associated with cross-breeding of the diploid and tetraploid parents so far available for triploid breeding. To solve this issue, prebreeding programmes at the diploid level could be set up to generate new improved parents that are homozygous for chromosome rearrangements. In the homozygous state, the alternative chromosome structures could recombine normally and unfavourable alleles would be purged more easily by selection. Prebreeding programmes at the diploid level would also enable an improvement in the cross-breeding value of the parents, prior to final crosses leading to triploid hybrids. The extensive QTL information produced in this study could be useful to guide such prebreeding programmes.

## Materials and methods

### Breeding population

The breeding population consisted of 2723 triploid hybrids resulting from biparental crosses implying 38 *M. acuminata* accessions including wild accessions and cultivars [29]. Triploid hybrids were obtained by crossing a diploid parent with a tetraploid parent, the latter resulting from chromosome doubling of a diploid accession using a colchicine treatment. The population represented 116 full-sib families, giving a relatively modest average number of progenies per family (23.47) due to the generally low fertility levels of most parental combinations. Hybrids were all produced and evaluated at CIRAD Neufchateau station, Capesterre Belle-Eau, Guadeloupe, French West Indies (16°05’N, 61°35’W, elevation 250 m, average rainfall 3500 mm, average temperature of 25°C, and soil classified as andosol). A subset of 1463 hybrids was genotyped using a GBS approach, which led after SNP quality filtering (see below) to a total of 1129 hybrids available for the GWAS analysis. This final subset of hybrids was derived from 21 *M. acuminata* accessions involved in 38 biparental combinations as diploid and/or tetraploid parents (Table 2). These accessions comprised three groups of somaclonal mutants (three mutant triplets) that are genetically indistinguishable but phenotypically distinct. The genome of these 21 accessions taken as a whole encompassed four major reciprocal translocations between four pairs of chromosomes, as compared with the ancestral *M. acuminata* structure [26], which is the structure of the *M. acuminata* reference sequence V4 [37]. All accessions were structurally heterozygous for one to three of these large reciprocal translocations, with the exception of one accession (Sinwogobi).

### Experimental design and phenotyping

All 2723 hybrids planted in field experiments were evaluated for the 24 agronomic traits listed in Table 1 that were related to yield components as well as plant, bunch, and fruit architectures.

The experimental design consisted of 12 trials comprising two to nine experimental unit blocks (48 in total) successively planted from 2011 to 2016. Each block contained 64 plants comprising 56 unreplicated hybrids and eight checks (five Cavendish, one Pisang Ceylan, one Pisang Madu, and one Calcutta 4) [29]. Phenotypic data were collected over three successive growth cycles (from 2012 to 2017). For each trait, best linear unbiased prediction (BLUP) of hybrid performance was calculated over the experimental design using the linear mixed model described in Toniutti [29] that accounts for diploid and tetraploid parental effects. Inference of model parameters was performed using ASReml-R (v3, [42]). Their estimates and trait heritability are presented in Table S1.

### Genotyping

A subset of 1463 individuals from the phenotyped triploid population were genotyped using GBS. Leaf samples were collected on the third leaf after the cigar leaf on adult individuals and DNA was extracted from 3 g of leaf according to a modified MATAB method [43]. Libraries were made at the GPTR platform (https://umr-agap.cirad.fr/en/plateformes/plateformes-regionales/genotyping) using PstI and MseI restriction enzymes and single-end sequencing was performed on the GeTPlaGe platform (https://get.genotoul.fr) or Genoscope (http://www.genoscope.cns.fr) using an Illumina HiSeq sequencer (Illumina, San Diego, CA, USA). Raw sequence reads were demultiplexed using GBSX, version 1.2 [44]. Adapters were removed and reads were quality trimmed using the CUTADAPT programme [45].

A triploid variant calling was performed on individuals using the *M. acuminata* reference sequence V4 [37] with vcfhunter toolbox (https://github.com/SouthGreenPlatform/VcfHunter) [46] as described in Baurens *et al.* [20]. Only bi-allelic sites with no indels were selected for the analyses. The genotype call was then diploidized in the sense that the two difficult-to-distinguish triploid heterozygous classes (0:0:1 and 0:1:1) were combined into a single heterozygous class (0:1), 0 and 1 being the reference and alternative allele, respectively. Genotypic data points were set as missing values if their read depth was <10 or >10 000 and, for heterozygous data points, if an allele was supported by less than three reads or with an allele depth ratio (i.e. allele depth to total read depth) <0.05. A prefiltered vcf was obtained by first eliminating SNPs with >50% missing values and then eliminating 334 individuals with >50% missing values. Polymorphic sites were additionally filtered following several criteria: (i) Removal of sites with >20% missing data on the 1129 remaining individuals using vcfFilter.1.0.py of vcfhunter toolbox. (ii) Selection of sites for which a proportion of heterozygous individuals is comprised within [0.1; 0.9] in at least one biparental population using the vcf2PopStat.py script added to vcfhunter toolbox. (iii) Selection of sites with minor allele frequency (MAF) >0.01 and a global heterozygosity comprised within [0.01; 0.99].

The final vcf file included 205 612 SNPs for 1129 individuals representing 38 families ranging in size from 2 to 141 individuals with a median number of individuals of 28.

### GWAS

The standard GWAS model of Yu *et al.* [18] was applied at each of the $M$ SNPs and is referred to as the ‘K model’ further in the text:


(1) \begin{equation*} {Y}_{ik}=\mu +{\alpha}_k+{\beta}^m{x}_{ik}^m+{G}_{ik}+{E}_{ik} \end{equation*}

where ${Y}_{ik}$ is the reference phenotypic value of hybrid $i$ from family $k$ (i.e. the BLUP calculated over the experimental design), $\mu$ is the intercept, ${\alpha}_k$ is effect of family $k$, ${\beta}^m$ is the effect of SNP $m$, ${x}_{ik}^m\in \left\{\mathrm{0,0.5,1}\right\}$ is the genotypic score of hybrid $i$ from family $k$ at SNP $m$, ${G}_{ik}$ is the polygenic background effect with $\boldsymbol{g}\sim N\left(0,{\sigma}_G^2\boldsymbol{K}\right)$, $\boldsymbol{g}$ is the vector of all ${G}_{ik}$, ${\sigma}_G^2$ is the genetic variance, $\boldsymbol{K}$ is the genomic relationship matrix, ${E}_{ik}$ is the error with $\boldsymbol{e}\sim N\left(0,{\sigma}_E^2\boldsymbol{I}\right)$, $\boldsymbol{e}$ is the vector of all ${E}_{ik}$, ${\sigma}_E^2$ is the error variance, $\boldsymbol{I}$ is the identity matrix, $\boldsymbol{g}$ and $\boldsymbol{e}$ being independent.

The genomic relationship ${K}_{ij}$ between two hybrids $i$ and $j$ is calculated following VanRaden [47]:


(2) \begin{equation*} {K}_{ij}=\frac{\sum_{m=1}^M{w}_{im}{w}_{jm}}{\sum_{m=1}^M{f}_m\left(1-{f}_m\right)} \end{equation*}

where ${w}_{im}={x}_{im}-{f}_m$is the centered genotypic score of hybrid $i$ at SNP $m$ and ${f}_m$ is the frequency of the alternative allele at SNP $m$. For the calculation of ${K}_{ij}$ only, missing ${x}_{im}$ values were imputed as ${f}_m$.

A second GWAS model adapted from Rincent *et al.* [19] was applied and is referred to as the ‘Kc model’ further in the text. It aimed at preventing the limitation of statistical power for large haplotypic segments showing reduced recombination and/or cosegregations with another haplotype segment due to reciprocal translocations at heterozygous state in parents. At each tested SNP $m$ from chromosome $c$, the GWAS model of Eq. (1) was adapted by computing the following genomic relationship ${K}_{ij}^c$ specific to chromosome $c$:


(3) \begin{equation*} {K}_{ij}^c=\frac{\sum_{m\in{S}_c}{w}_{im}{w}_{jm}}{\sum_{m\in{S}_c}{f}_m\left(1-{f}_m\right)} \end{equation*}

where ${S}_c$ is the set of markers to be included in the calculation of ${K}_{ij}^c$, all other terms being identical to those presented in Eq. (2). Each ${S}_c$ excludes all markers from its own chromosome $c$. When additional chromosomes are involved in a network of reciprocal translocations with chromosome $c$ in some parents, they were also excluded from ${S}_c$: (i) chromosomes 1, 4, and 7 were all excluded from their respective ${S}_c$ because of the existence of the 1/7 and 1/4 reciprocal translocations, and (ii) chromosomes 2, 3, and 8 were all excluded from their respective ${S}_c$ because of the existence of the 2/8 and 3/8 reciprocal translocations.

Model parameters were estimated using restricted maximum likelihood and the effect of each marker ${\beta}^m$ was tested using a Wald test, both implemented in the R-package ‘MM4LMM’ [48] available from the CRAN. As the second GWAS model tended not to control sufficiently for false positive, an inflation factor $\lambda$ was calculated as the median value of the Wald statistic over the $M$ SNPs divided by the expected median. Following Delvin and Roeder [49], the Wald statistic of each test was adjusted by dividing it by $\lambda$. The familywise error rate was controlled using either (i) a Bonferroni correction by dividing the type I error ($\alpha =5\%$) by the number of SNPs $M$ or (ii) by applying the false discovery rate procedure of Benjamini and Yekutieli [50] jointly to the set of *P*-values of all traits and GWAS methods.

For all GWAS, quantile–quantile (Q-Q) and Manhattan plots were generated. Significant SNPs were aggregated into QTL LD-based intervals using the following procedure: (i) adjacent significant SNPs were first grouped into clusters when they were <2 Mbp apart, (ii) LD interval was calculated using the position of the first and last SNPs (significant or not) in LD of at least 0.25 with the most significant SNP of the cluster, (iii) when overlapping LD intervals were observed for a given trait, they were merged into a single interval, and (iv) LD intervals shorter than 1 kb were discarded as they likely resulted from one or few markers incorrectly positioned on the reference genome. The LD between two SNPs $m$ and ${m}^{\prime }$ from chromosome $c$ was adapted from Mangin *et al.* [51] to correct for bias due to relatedness between hybrids:


(4) \begin{equation*} {r}_{m,m\prime}^2=\frac{{\left({\boldsymbol{w}}_m^T{\boldsymbol{K}}_c^{-1}{\boldsymbol{w}}_{m\prime}\right)}^2}{\left({\boldsymbol{w}}_m^T{\boldsymbol{K}}_c^{-1}{\boldsymbol{w}}_m\right)\left({\boldsymbol{w}}_{m\prime}^T{\boldsymbol{K}}_c^{-1}{\boldsymbol{w}}_{m\prime}\right)} \end{equation*}

where ${\boldsymbol{w}}_m^T=\left({w}_{1m},\dots, {w}_{im},\dots, {w}_{Nm}\right)$ and${\boldsymbol{K}}_c$ is the genomic relationship matrix of Eq. (3). Cosegregation between QTL was highlighted by computing the ${r}_{m,m\prime}^2$ between the most significant SNPs of QTL LD intervals. A representation of the consensus QTL intervals for each trait was made by merging overlapping LD intervals obtained for models K and Kc.

### Ancestral origin of alleles

Amongst all SNPs, 40 340 presented an allele for which an ancestral origin was determined in Martin *et al.* [6]. Ancestral origins correspond to the following *Musa* species and genetic groups: banksii, burmannica, malaccensis, shizocarpa, zebrina, and two unknown ancestral groups M1 and M2. The two unknown ancestral groups M1 and M2 were associated with the groups to which they are most closely related, i.e. schizocarpa for M1 and zebrina for M2 [6]. This strategy was motivated by the fact that a large number of alleles of M1 and M2 origin were probably incorrectly attributed to schizocarpa and zebrina, respectively. This is due to the small number of M1 and M2 representatives (always admixed) that allowed these alleles to be assigned. For each detected QTL, estimated allele effects were plotted along the chromosome with colouring according to ancestral origin.

### Meta-analysis

A meta-analysis of all traits and methods was performed to assess possible pleiotropic effects of identified QTLs. Using the most significant markers of each LD interval, a z-score ${Z}_{mt}$ of marker $m$ for trait $t$ was calculated as following:


(5) \begin{equation*} {Z}_{mt}=-{\varPhi}^{-1}\left(0.5{p}_{mt}\right)\times \mathit{\operatorname{sign}}\left({\beta}_{mt}\right) \end{equation*}

where $\varPhi (.)$ stands for the standard Gaussian cumulative distribution function and $\mathit{\operatorname{sign}}\left({\beta}_{mt}\right)$ is the the sign of the estimated effect of marker marker $m$ for trait $t$. Note that the z-scores were calculated using the *P*-value and sign of the effect corresponding to those of the method (i.e. K or Kc model) by which the marker was detected. When a same marker was detected using both methods, a single z-score was calculated using the method with the most significant *P*-value. The effect considered was that of the allele alternative to the reference genome.

## Supplementary Material

Web_Material_uhae307
