# Supplementary material for: Genome-wide association for agro-morphological traits in a triploid banana population with large chromosome rearrangements
Source: Hortic Res. 2024 Nov 6;12(2):uhae307. doi: 10.1093/hr/uhae307 (PMC11817881; doi:10.1093/hr/uhae307)
Supplement: Web_Material_uhae307 [file web_material_uhae307.zip › Figure_S1.pdf]

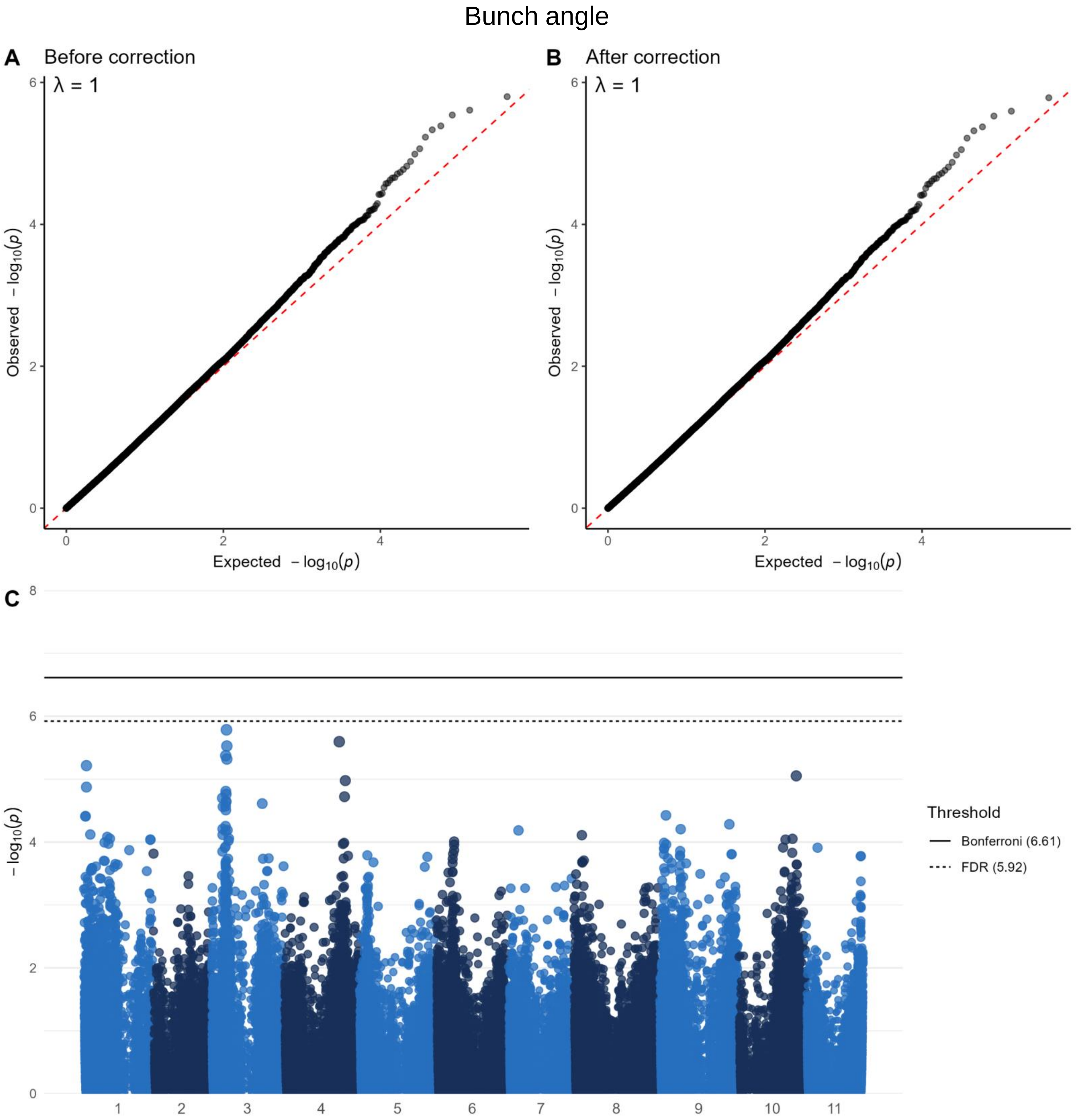

Figure S1A: QQ-plots of the p-values of the K model for bunch angle before (**A**) and after (**B**) the correction by the inflation factor λ, and Manhattan plot (**C**) of the corrected p-values with the Bonferroni and FDR -$\log_{10}$(p-value) thresholds

Peduncle diameter

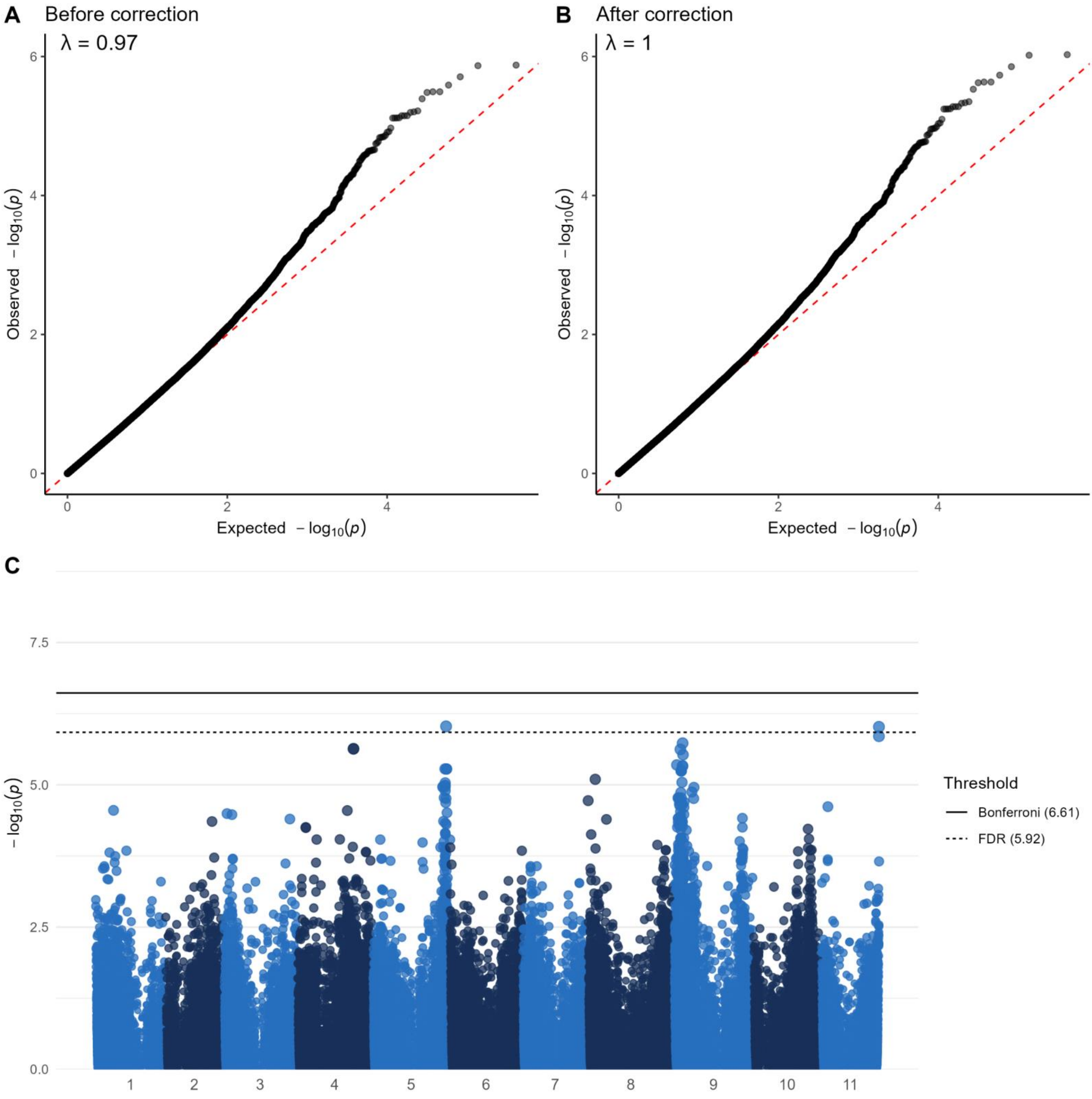

Figure S1B: QQ-plots of the p-values of the K model for peduncle diameter before (**A**) and after (**B**) the correction by the inflation factor λ, and Manhattan plot (**C**) of the corrected p-values with the Bonferroni and FDR -$\log_{10}$(p-value) thresholds

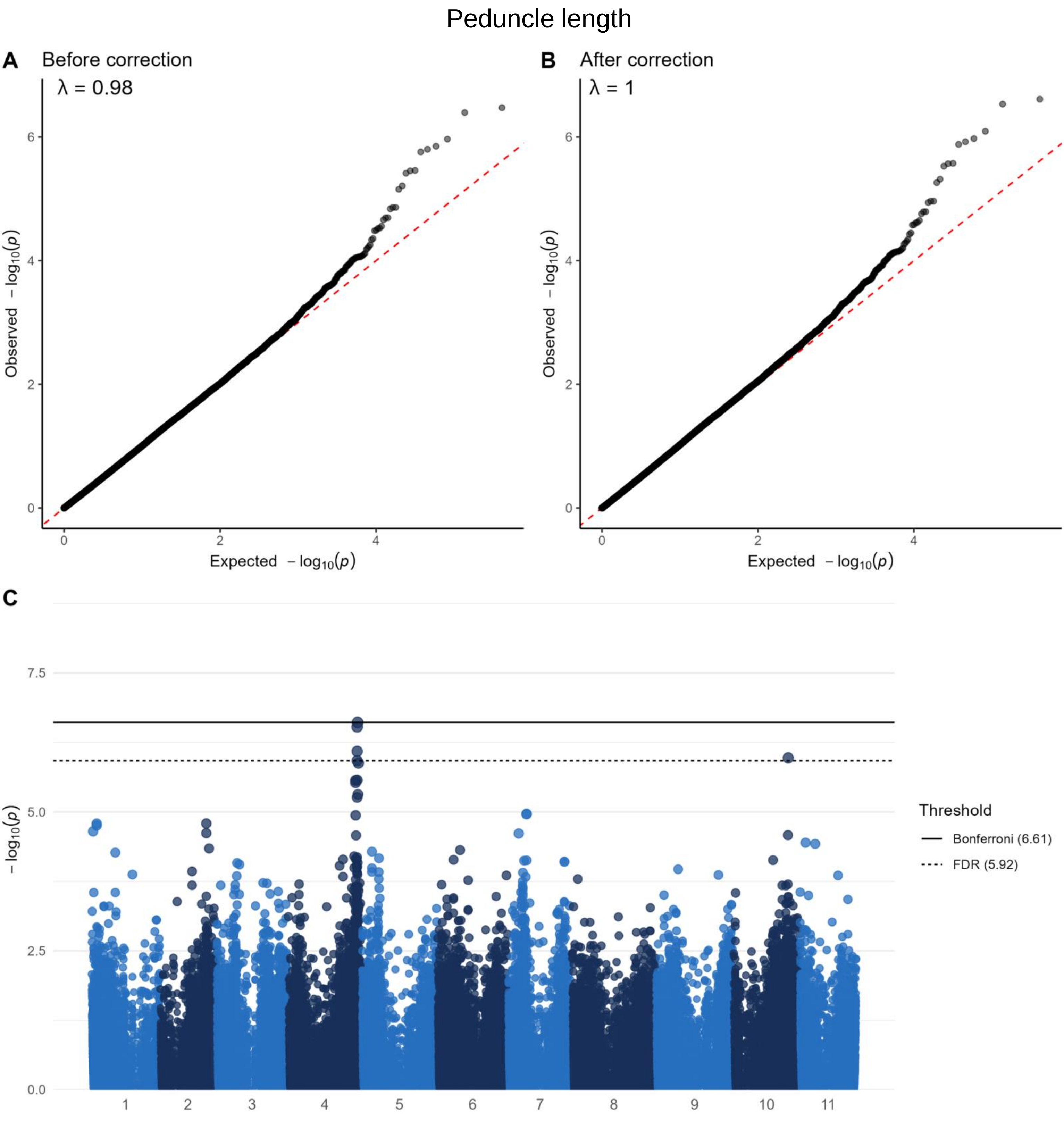

Figure S1C: QQ-plots of the p-values of the K model for peduncle length before (**A**) and after (**B**) the correction by the inflation factor λ, and Manhattan plot (**C**) of the corrected p-values with the Bonferroni and FDR -$\log_{10}$(p-value) thresholds

Peduncle index

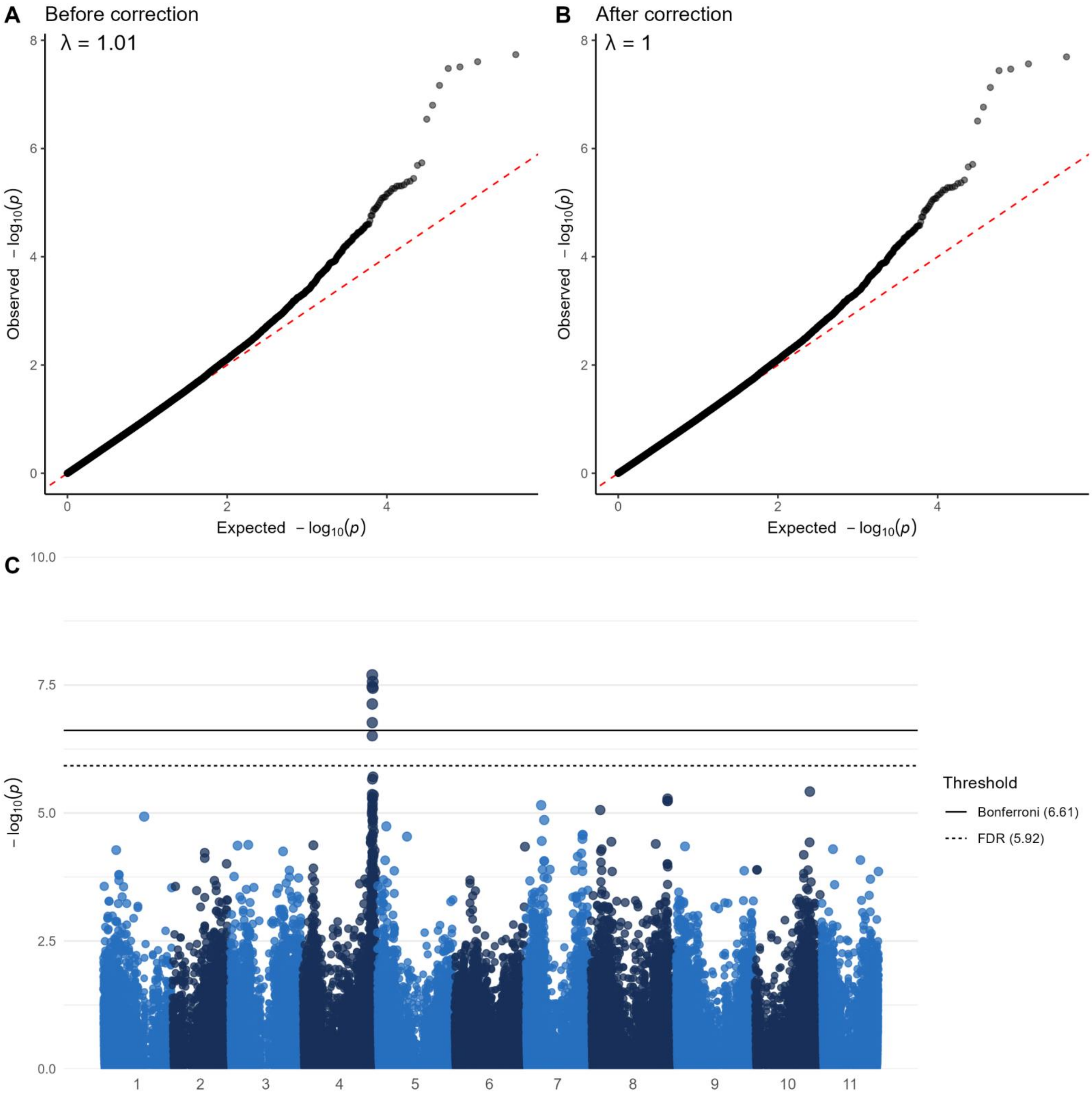

Figure S1D: QQ-plots of the p-values of the K model for peduncle index before (**A**) and after (**B**) the correction by the inflation factor λ, and Manhattan plot (**C**) of the corrected p-values with the Bonferroni and FDR -$\log_{10}$(p-value) thresholds

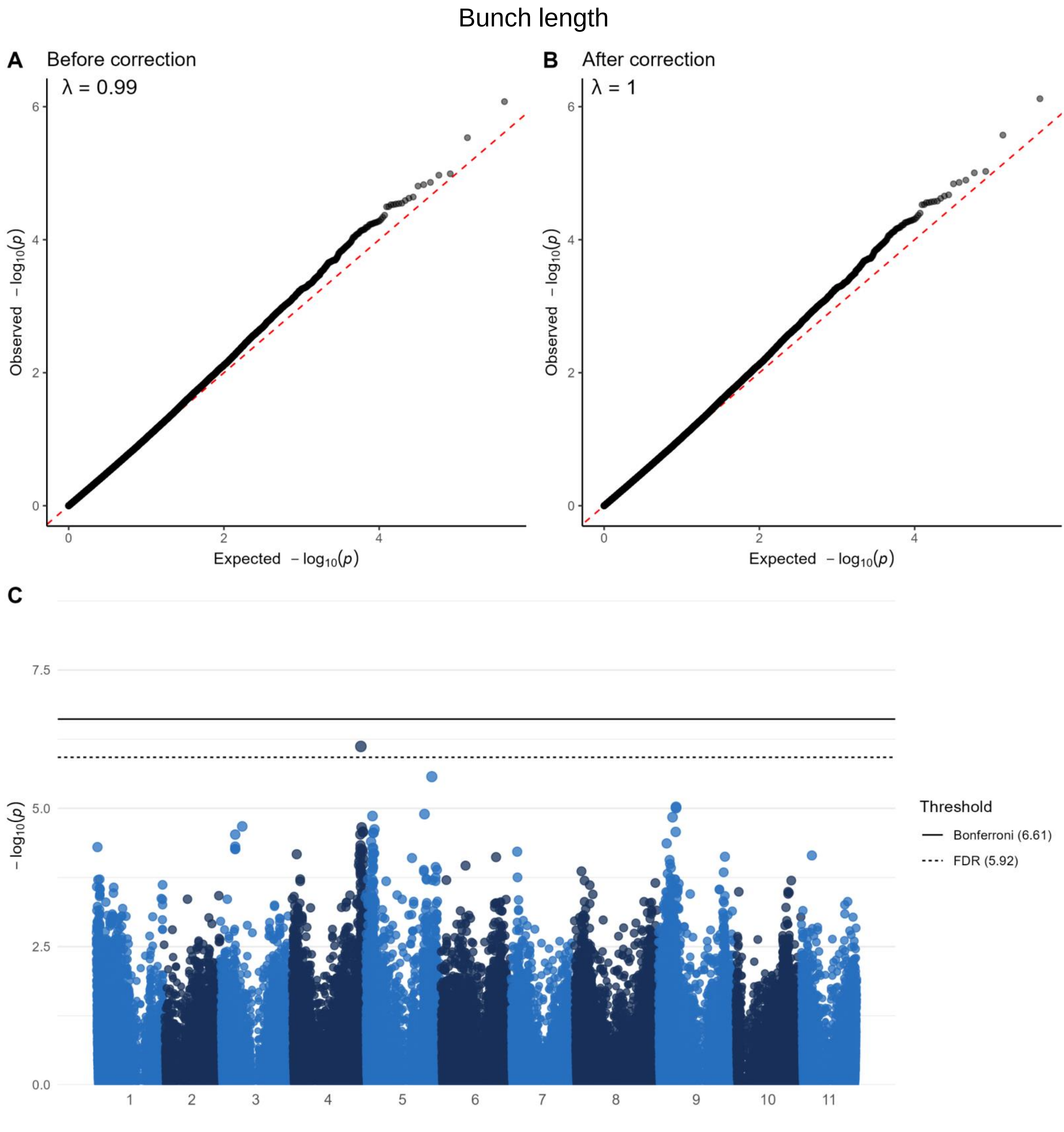

Figure S1E: QQ-plots of the p-values of the K model for bunch length before (**A**) and after (**B**) the correction by the inflation factor λ, and Manhattan plot (**C**) of the corrected p-values with the Bonferroni and FDR -log_{10}(p-value) thresholds

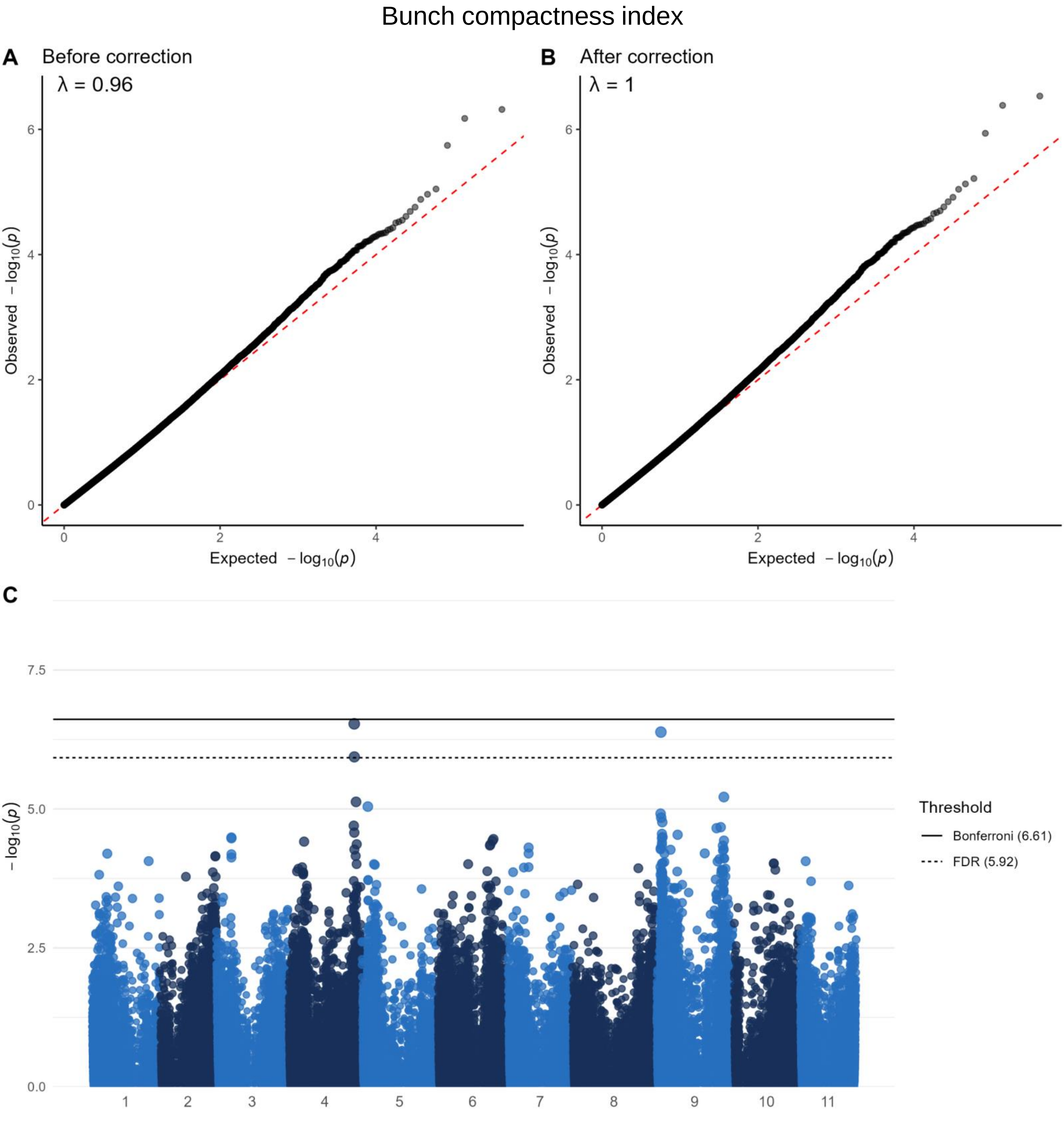

Figure S1F: QQ-plots of the p-values of the K model for bunch compactness index before (**A**) and after (**B**) the correction by the inflation factor λ, and Manhattan plot (**C**) of the corrected p-values with the Bonferroni and FDR -$\log_{10}$(p-value) thresholds

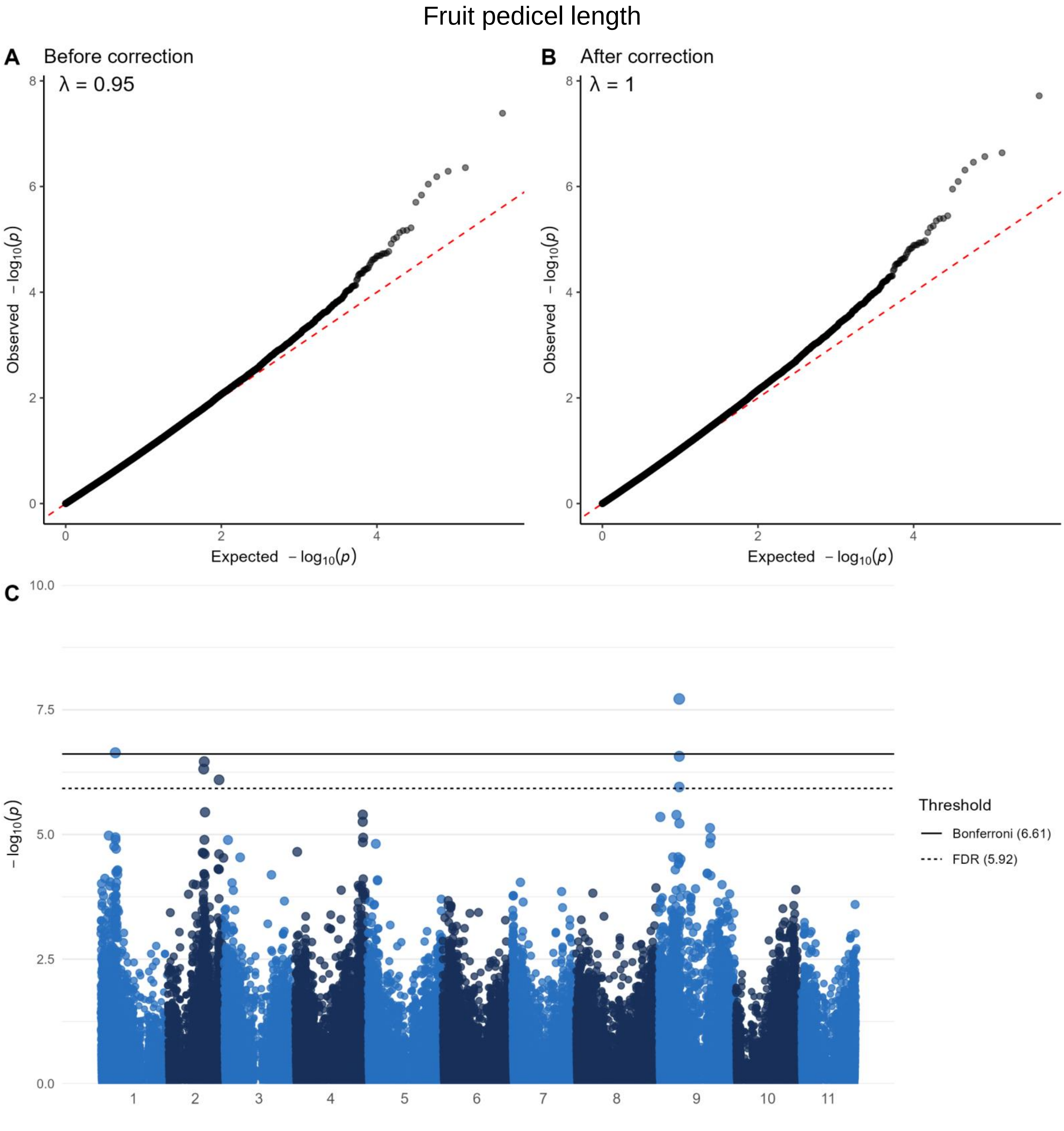

Figure S1G: QQ-plots of the p-values of the K model for fruit pedicel length before (**A**) and after (**B**) the correction by the inflation factor λ, and Manhattan plot (**C**) of the corrected p-values with the Bonferroni and FDR -$\log_{10}$(p-value) thresholds

Fruit pedicel diameter

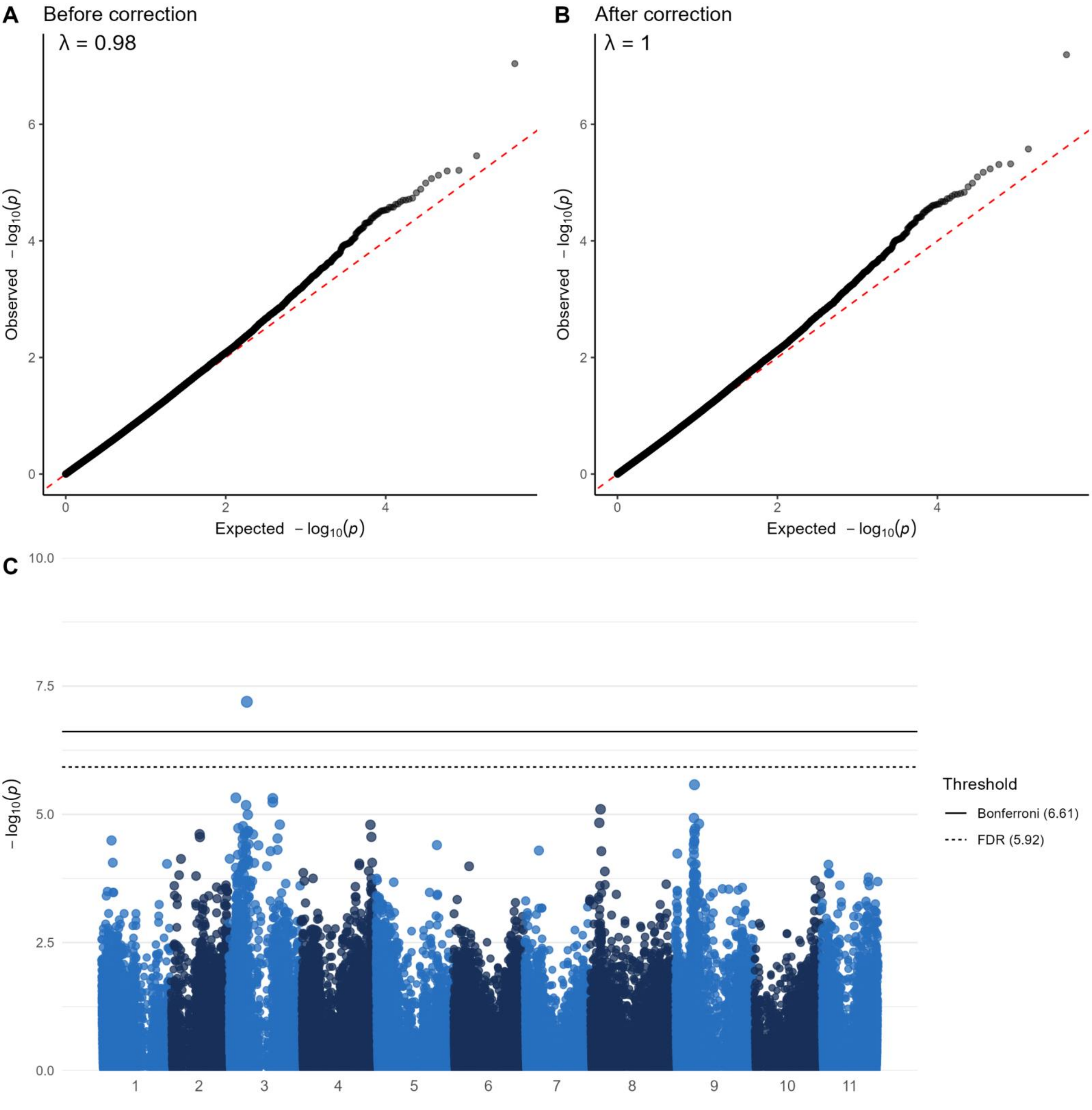

Figure S1H: QQ-plots of the p-values of the K model for fruit pedicel diameter before (**A**) and after (**B**) the correction by the inflation factor λ, and Manhattan plot (**C**) of the corrected p-values with the Bonferroni and FDR -$\log_{10}$(p-value) thresholds

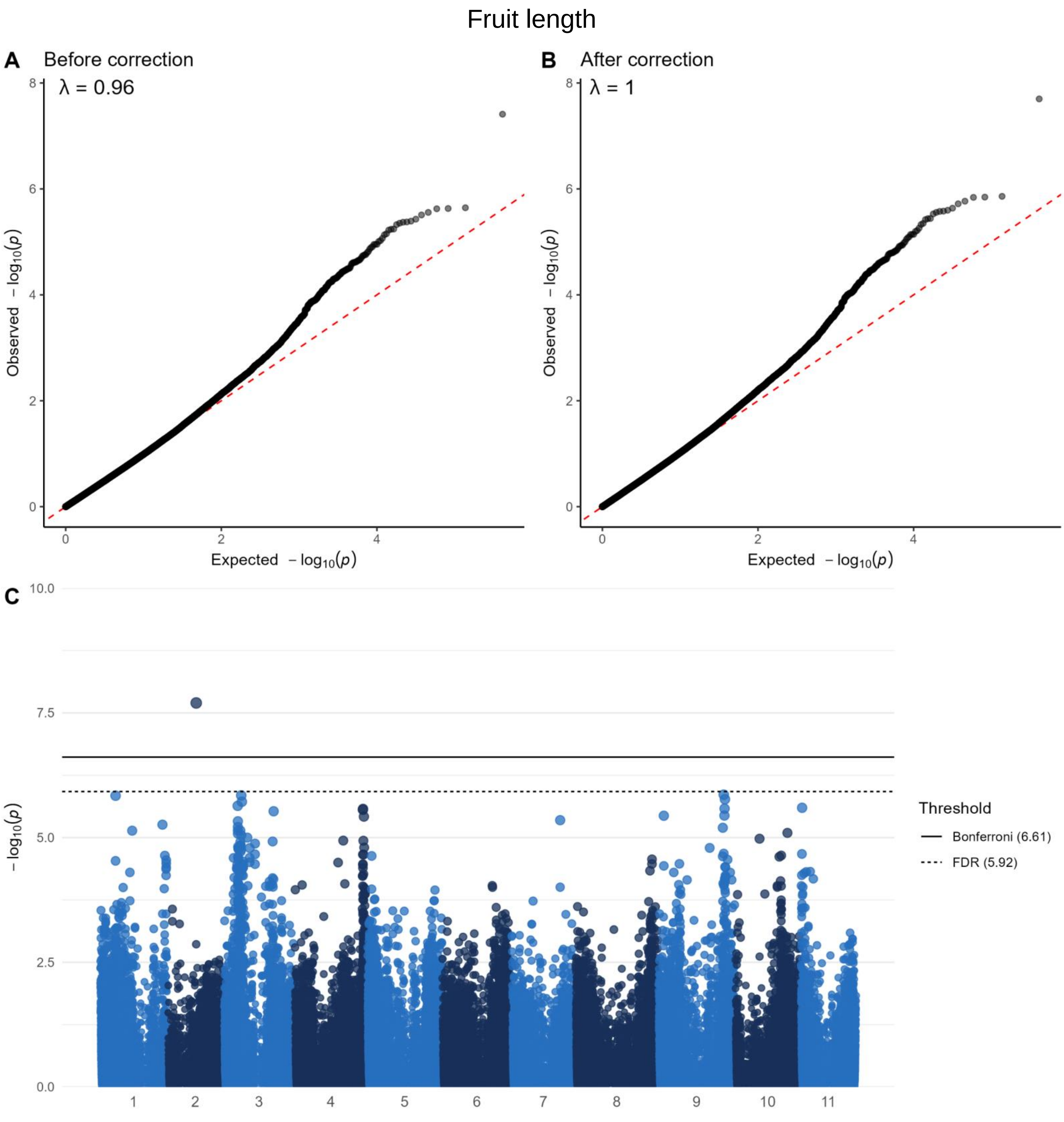

Figure S1I: QQ-plots of the p-values of the K model for fruit length before (**A**) and after (**B**) the correction by the inflation factor λ, and Manhattan plot (**C**) of the corrected p-values with the Bonferroni and FDR -$\log_{10}$(p-value) thresholds

Fruit grade

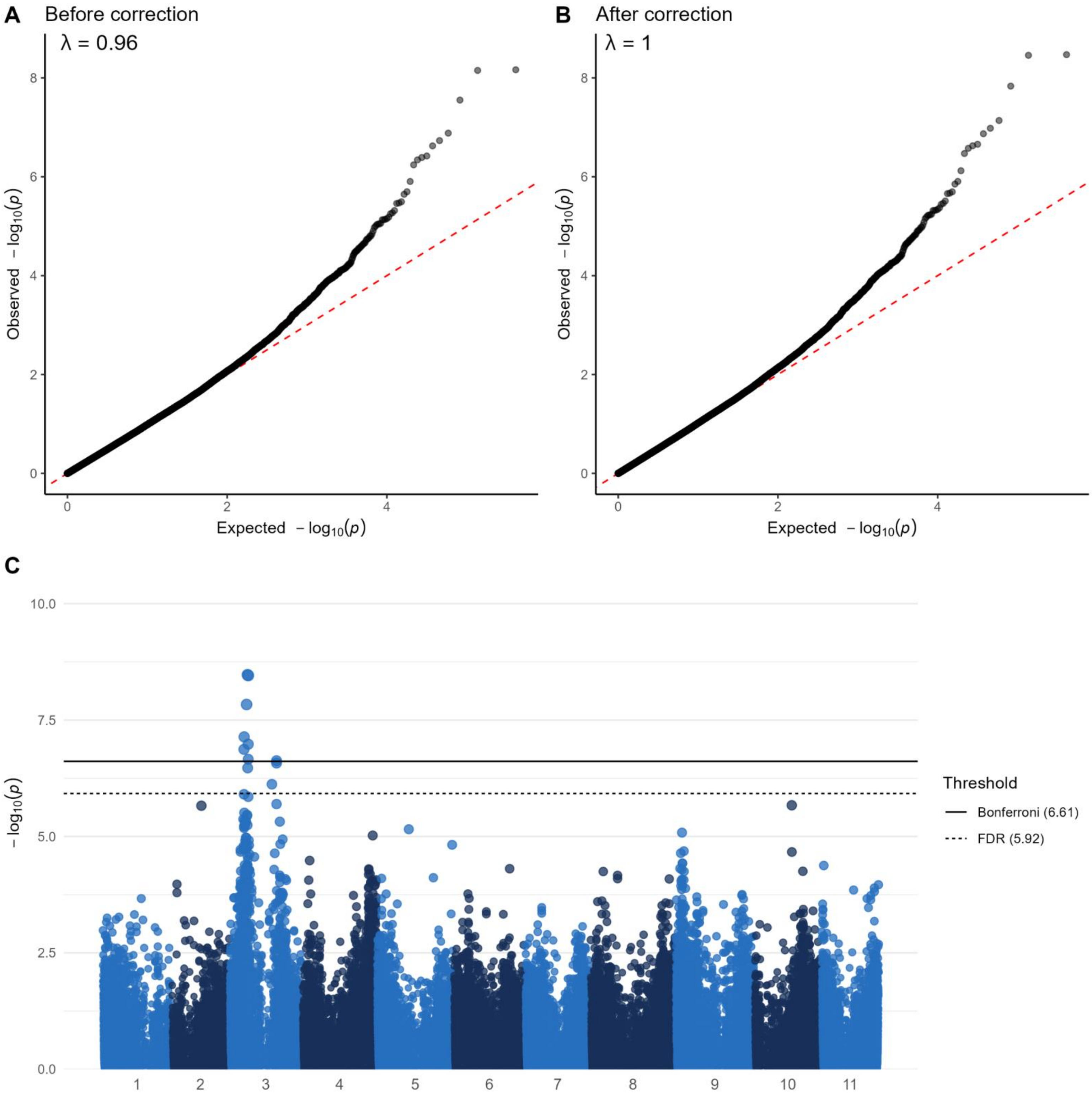

Figure S1J: QQ-plots of the p-values of the K model for fruit grade before (**A**) and after (**B**) the correction by the inflation factor λ, and Manhattan plot (**C**) of the corrected p-values with the Bonferroni and FDR -$\log_{10}$(p-value) thresholds

Fruit weight

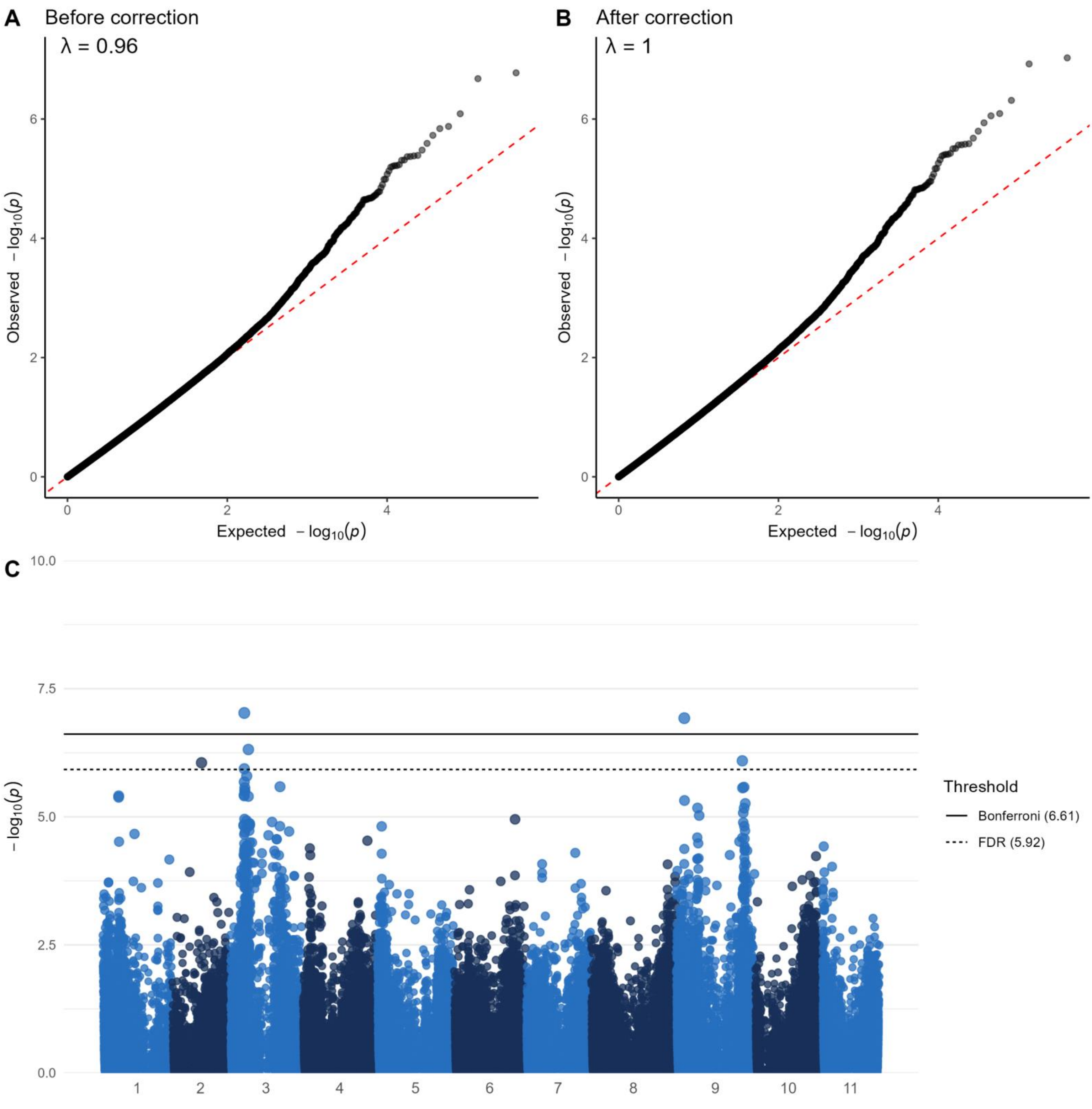

Figure S1K: QQ-plots of the p-values of the K model for fruit weight before (**A**) and after (**B**) the correction by the inflation factor λ, and Manhattan plot (**C**) of the corrected p-values with the Bonferroni and FDR -$\log_{10}$(p-value) thresholds

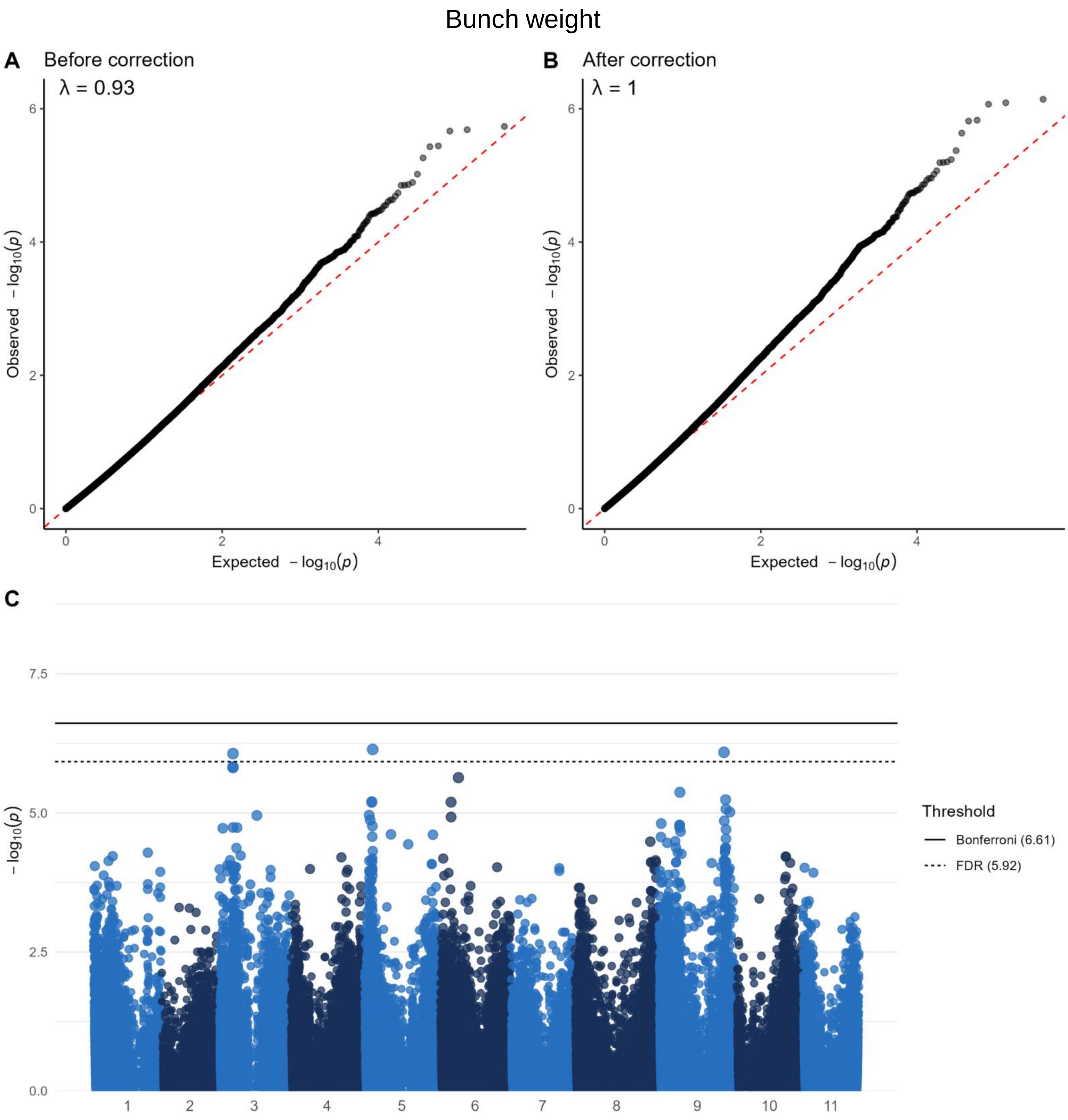

Figure S1L: QQ-plots of the p-values of the K model for fruit weight before (**A**) and after (**B**) the correction by the inflation factor λ, and Manhattan plot (**C**) of the corrected p-values with the Bonferroni and FDR -$\log_{10}$(p-value) thresholds

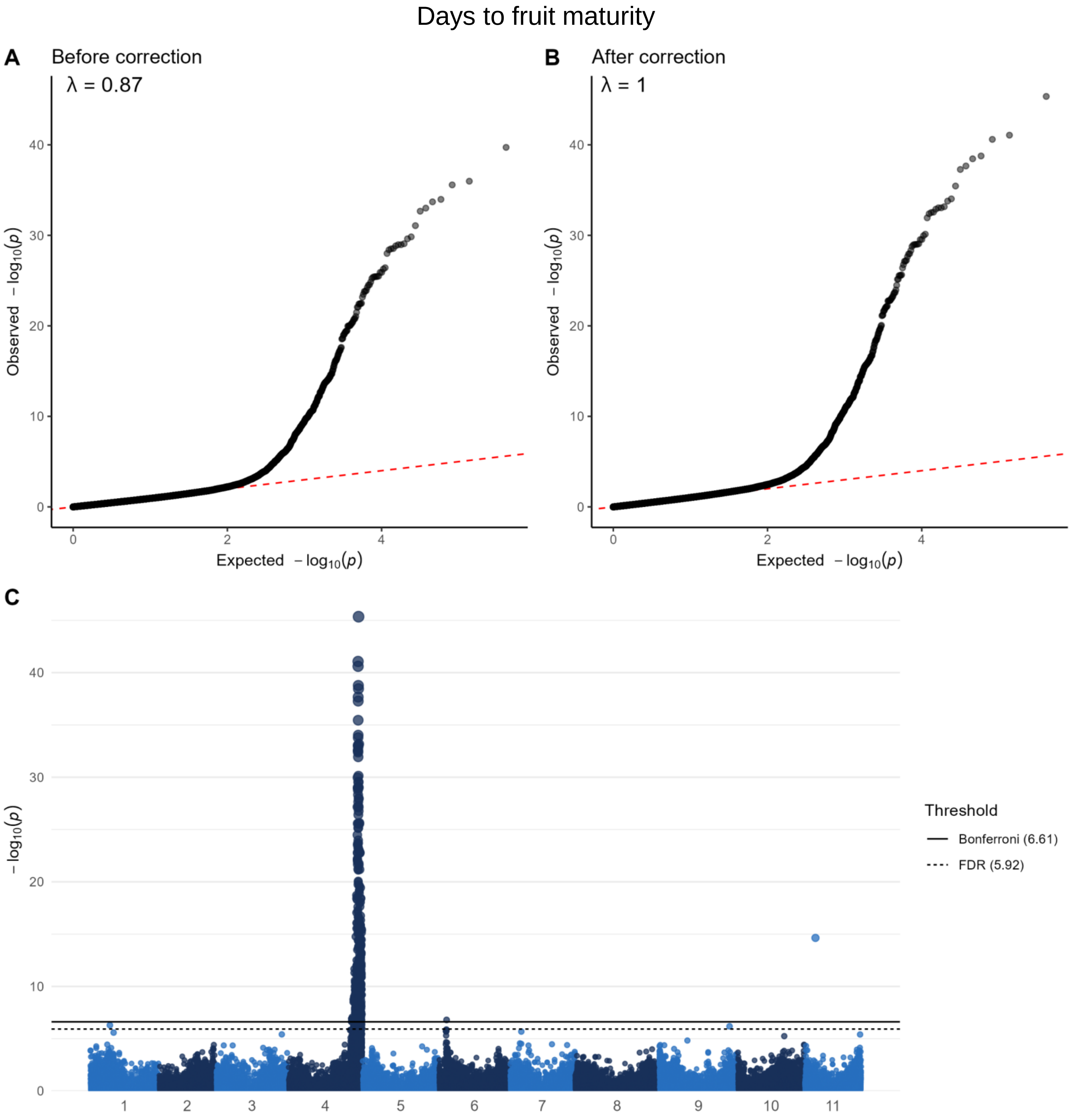

Figure S1M: QQ-plots of the p-values of the K model for days to fruit maturity before (**A**) and after (**B**) the correction by the inflation factor λ, and Manhattan plot (**C**) of the corrected p-values with the Bonferroni and FDR -$\log_{10}$(p-value) thresholds

Number of fruits per hand

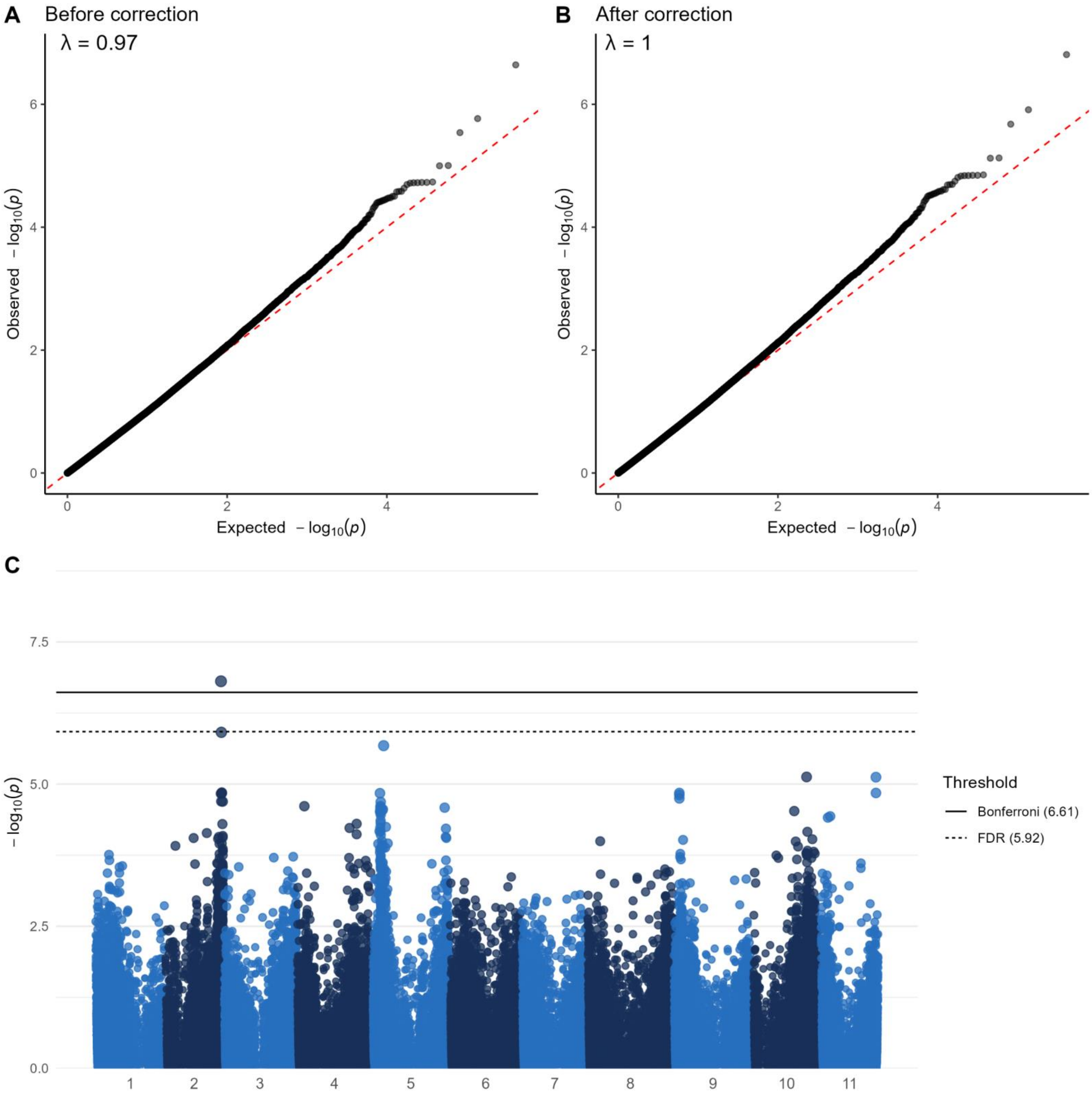

Figure S1N: QQ-plots of the p-values of the K model for number of fruits per hand before (**A**) and after (**B**) the correction by the inflation factor λ, and Manhattan plot (**C**) of the corrected p-values with the Bonferroni and FDR -$\log_{10}$(p-value) thresholds

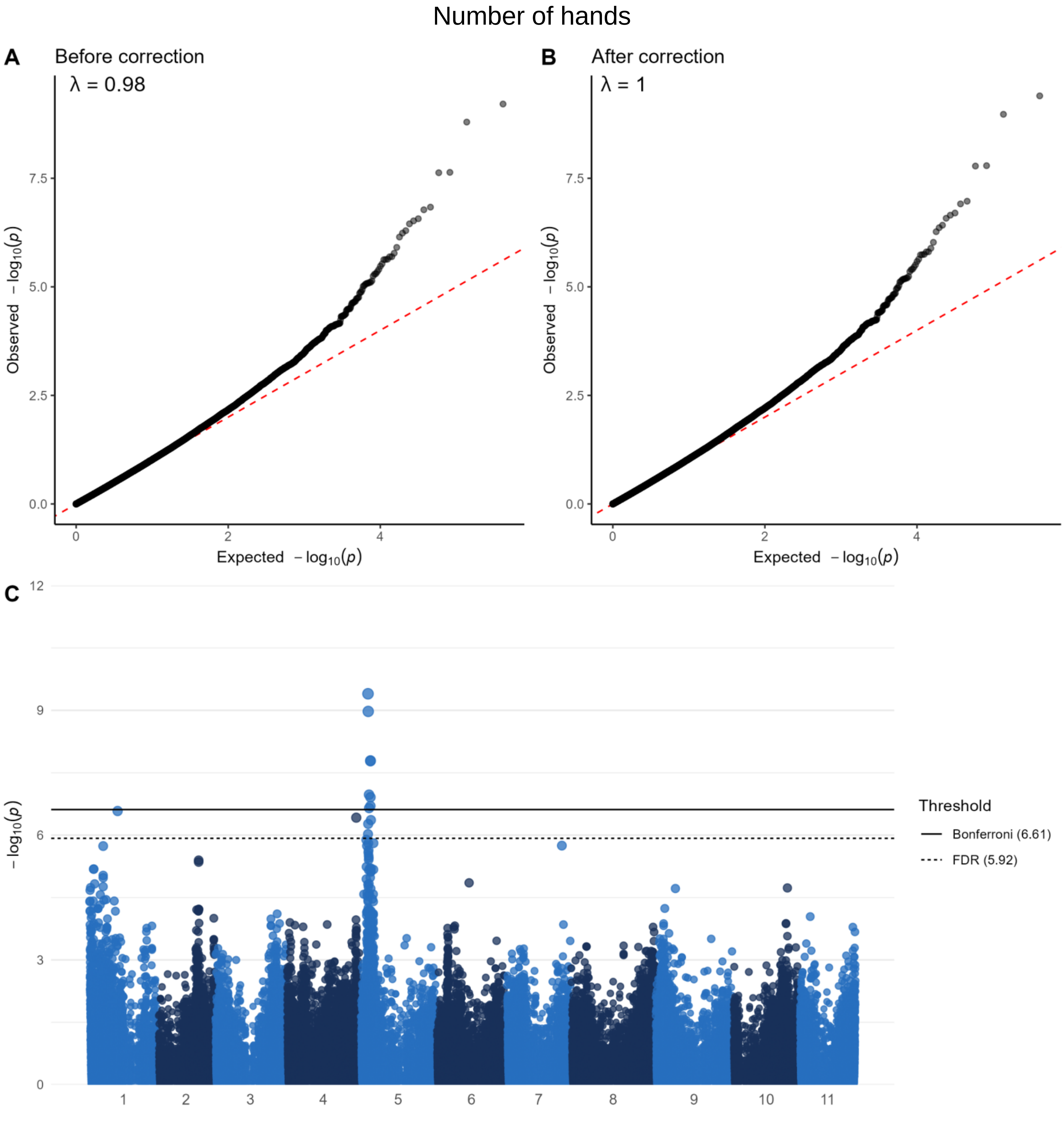

Figure S1O: QQ-plots of the p-values of the K model for number of hands before (**A**) and after (**B**) the correction by the inflation factor λ, and Manhattan plot (**C**) of the corrected p-values with the Bonferroni and FDR -$\log_{10}$(p-value) thresholds

Number of fruits

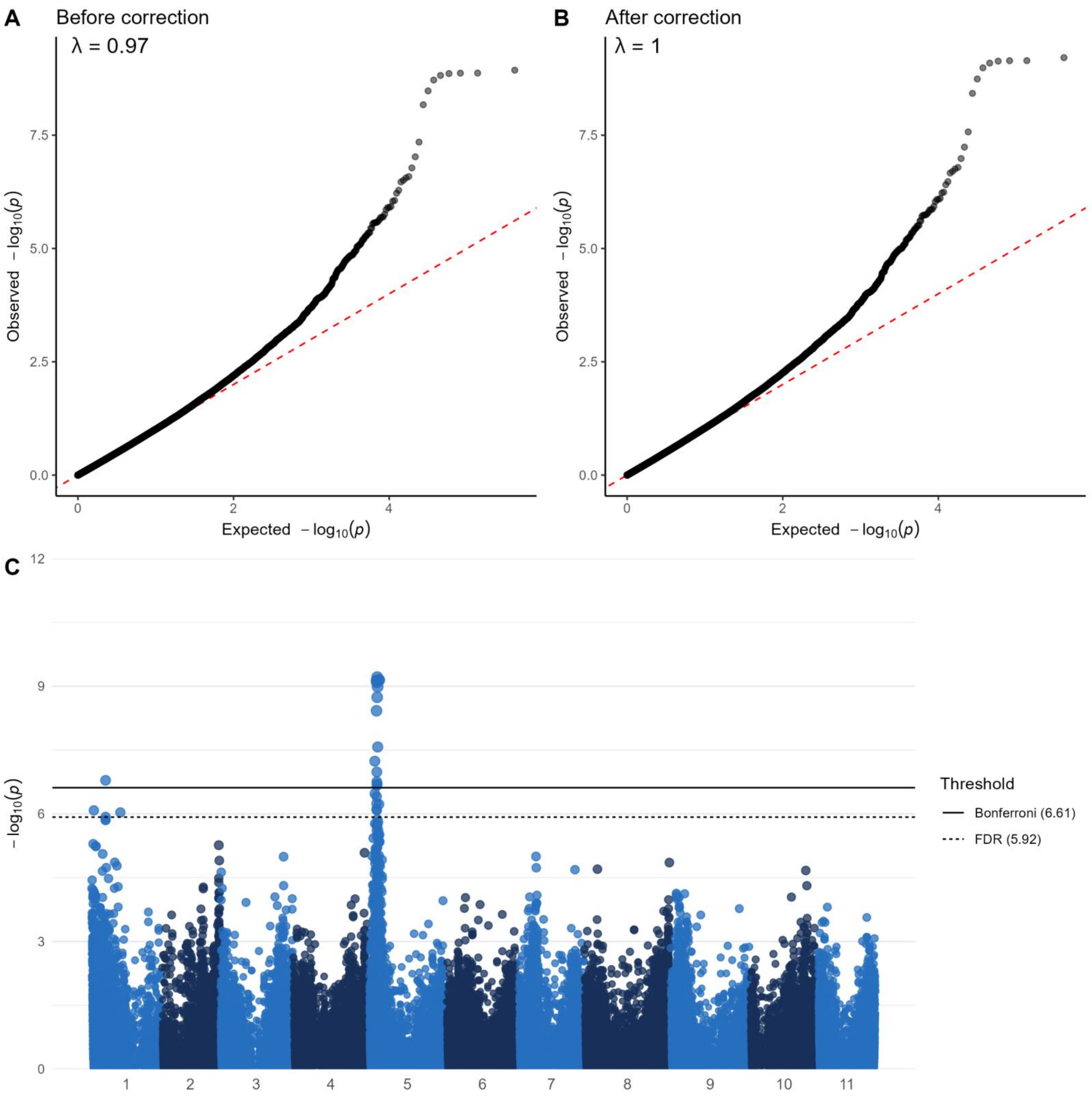

Figure S1P: QQ-plots of the p-values of the K model for number of fruits before (**A**) and after (**B**) the correction by the inflation factor λ, and Manhattan plot (**C**) of the corrected p-values with the Bonferroni and FDR -$\log_{10}$(p-value) thresholds

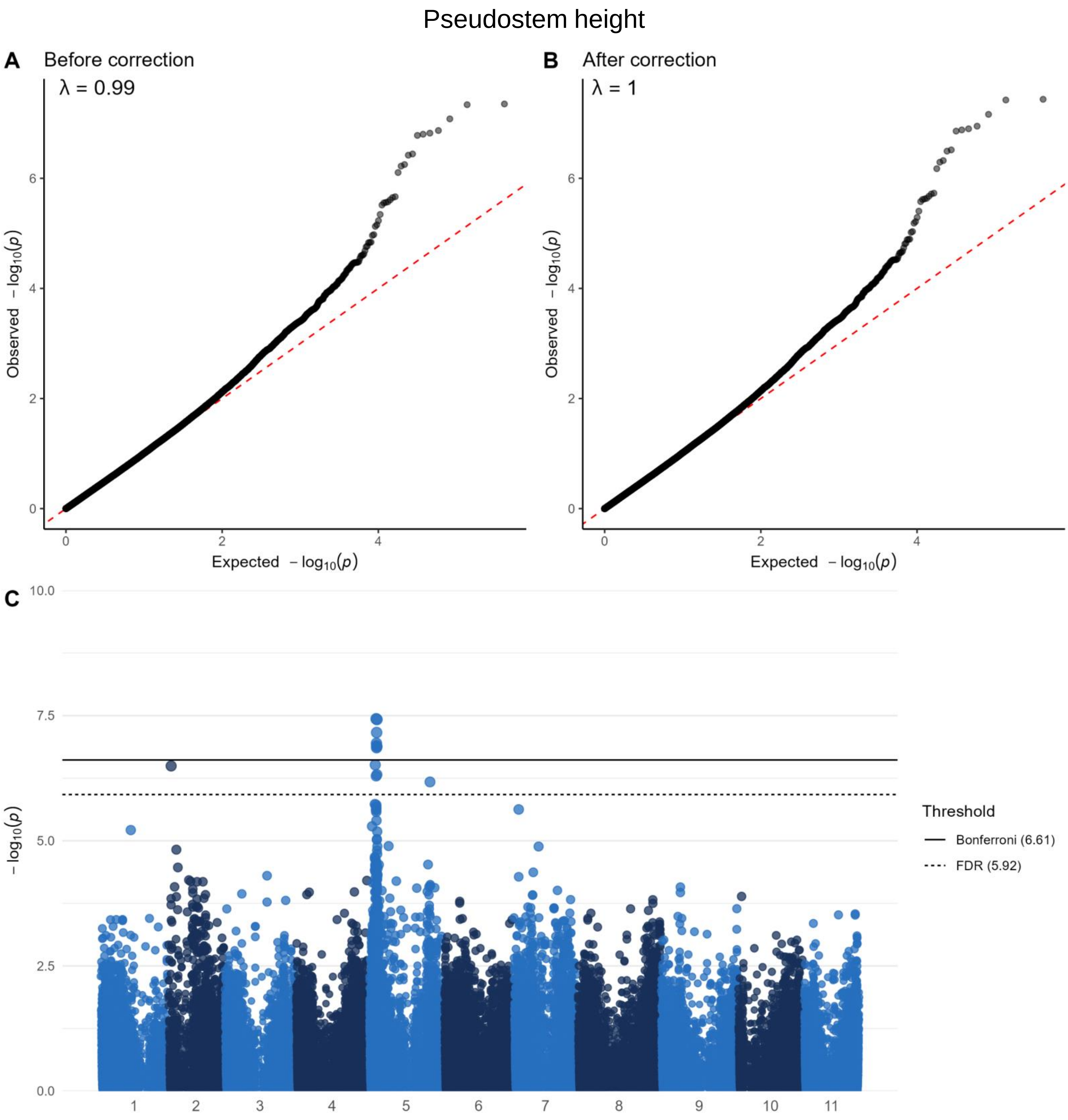

Figure S1Q: QQ-plots of the p-values of the K model for pseudostem height before (**A**) and after (**B**) the correction by the inflation factor λ, and Manhattan plot (**C**) of the corrected p-values with the Bonferroni and FDR -$\log_{10}$(p-value) thresholds

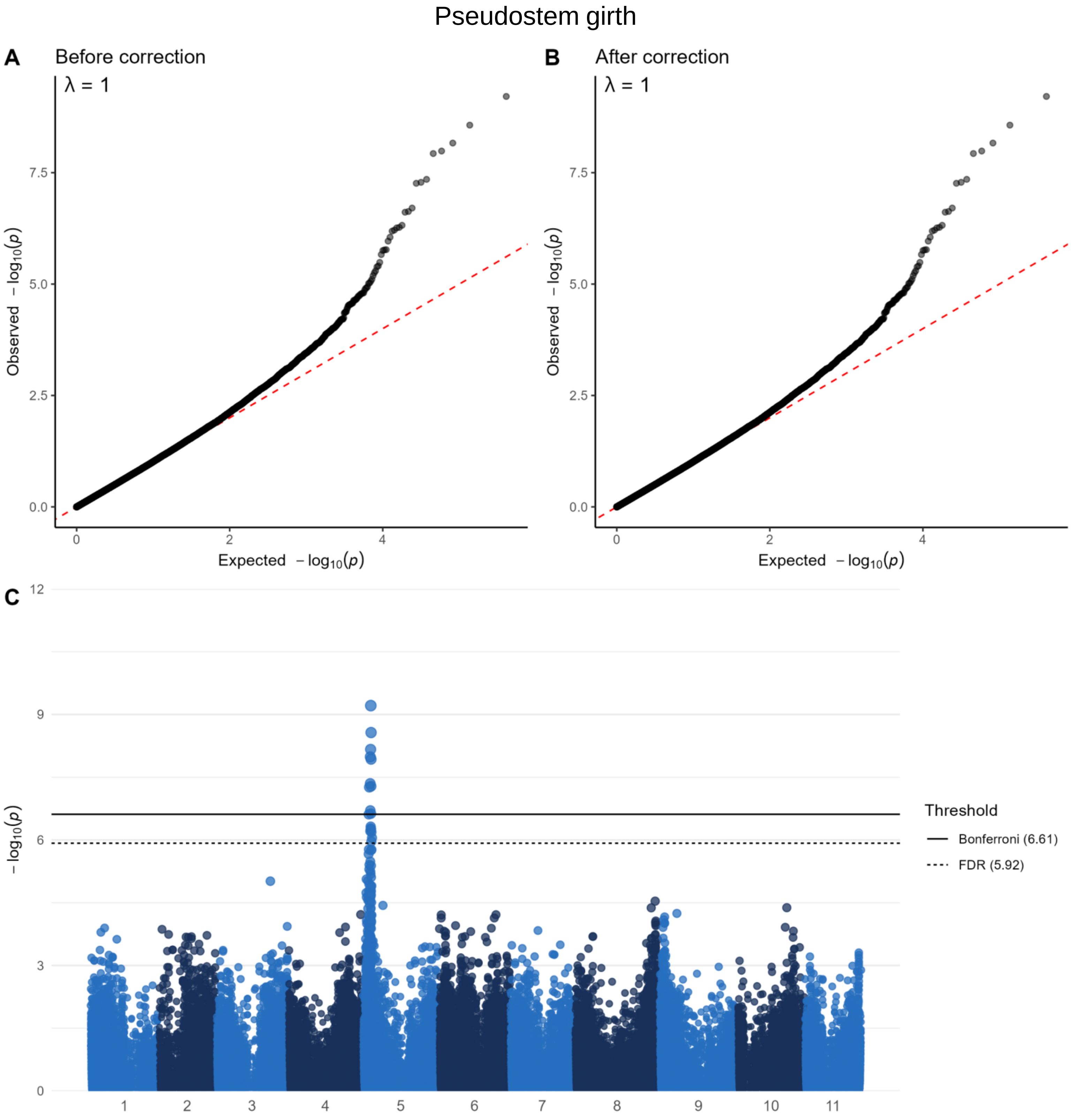

Figure S1R: QQ-plots of the p-values of the K model for pseudostem girth before (**A**) and after (**B**) the correction by the inflation factor λ, and Manhattan plot (**C**) of the corrected p-values with the Bonferroni and FDR -$\log_{10}$(p-value) thresholds

Leaf blade length

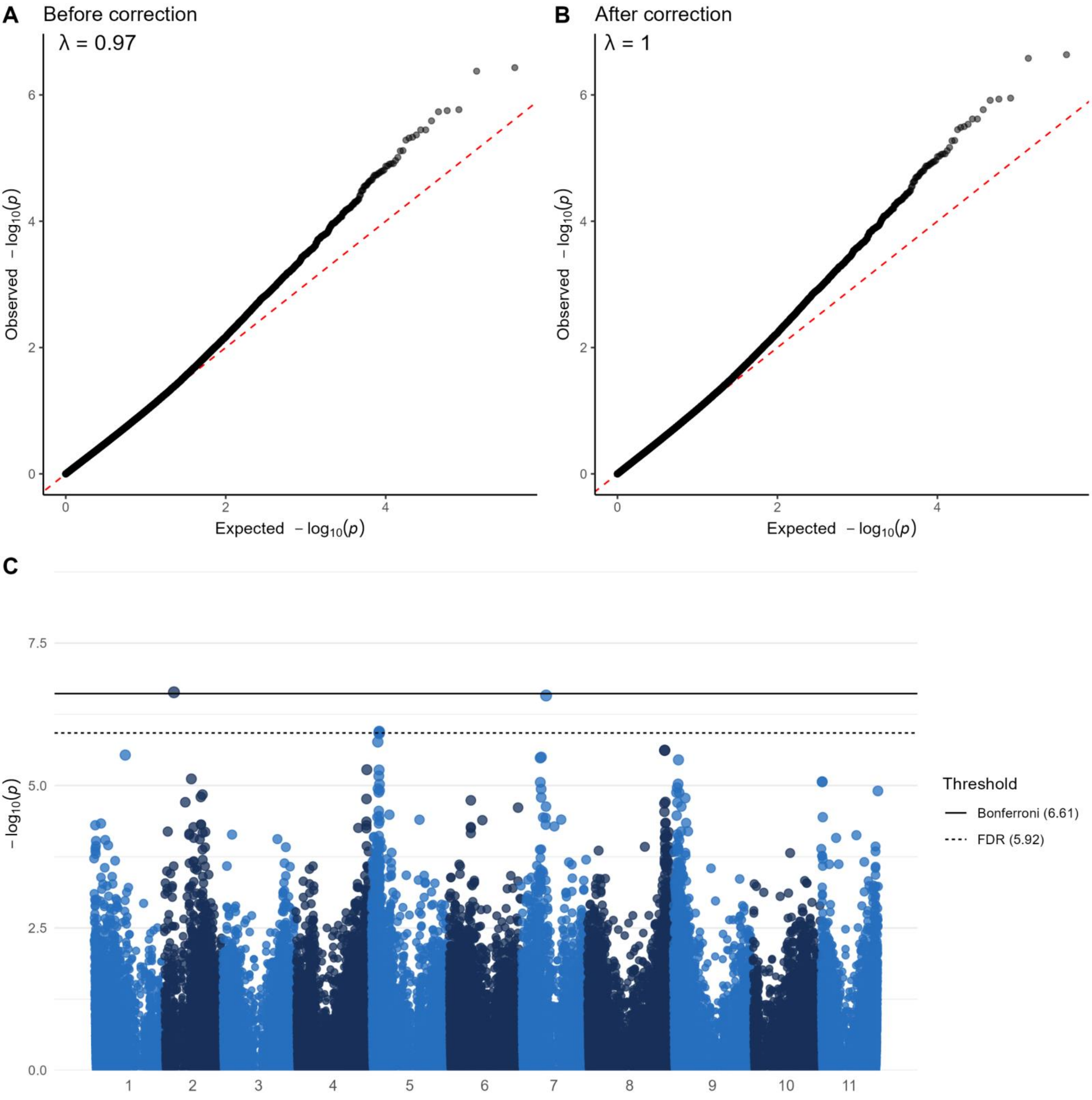

Figure S1S: QQ-plots of the p-values of the K model for leaf blade length before (**A**) and after (**B**) the correction by the inflation factor λ, and Manhattan plot (**C**) of the corrected p-values with the Bonferroni and FDR -$\log_{10}$(p-value) thresholds

Leaf blade width

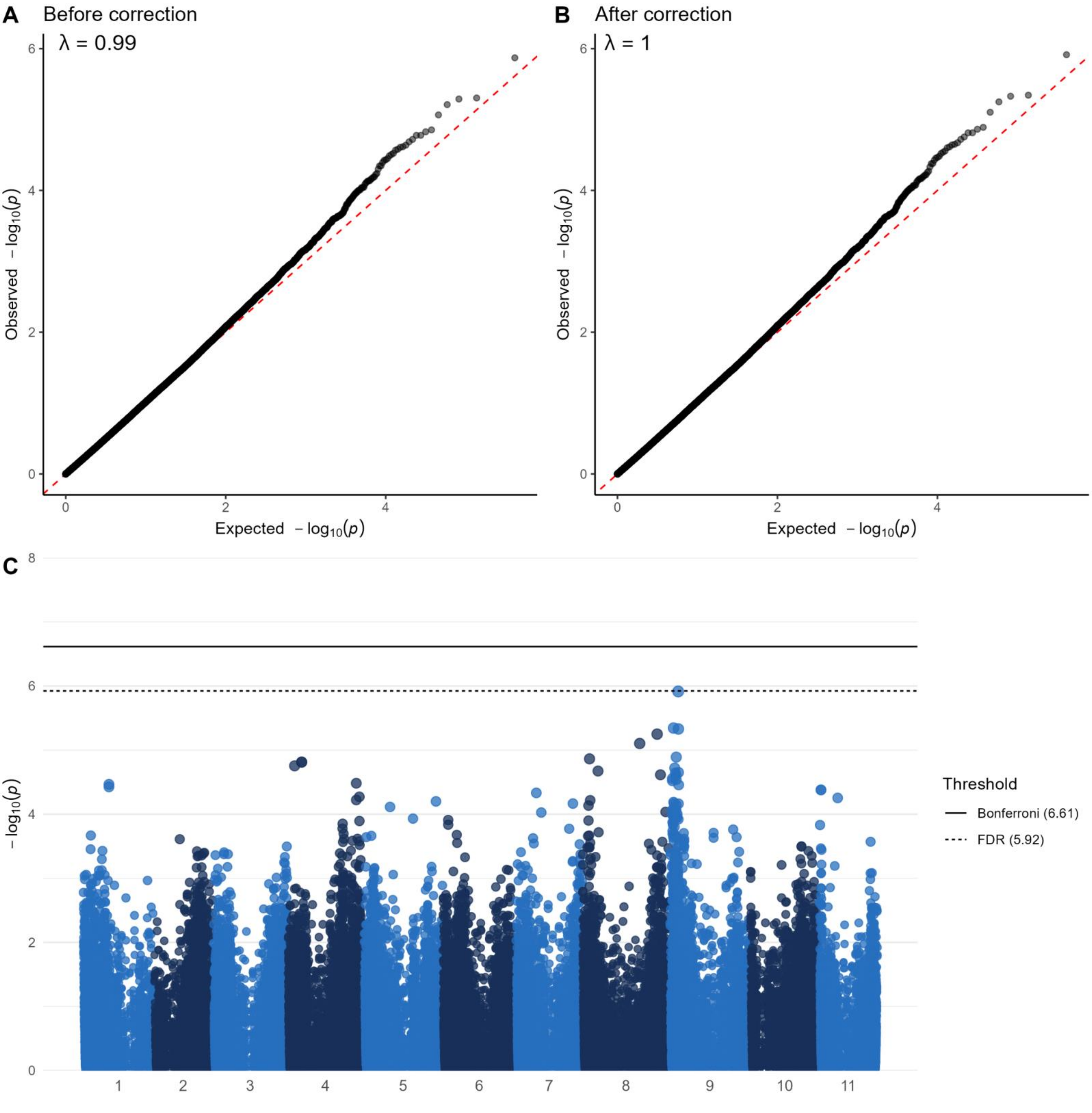

Figure S1T: QQ-plots of the p-values of the K model for leaf blade width before (**A**) and after (**B**) the correction by the inflation factor λ, and Manhattan plot (**C**) of the corrected p-values with the Bonferroni and FDR -$\log_{10}$(p-value) thresholds

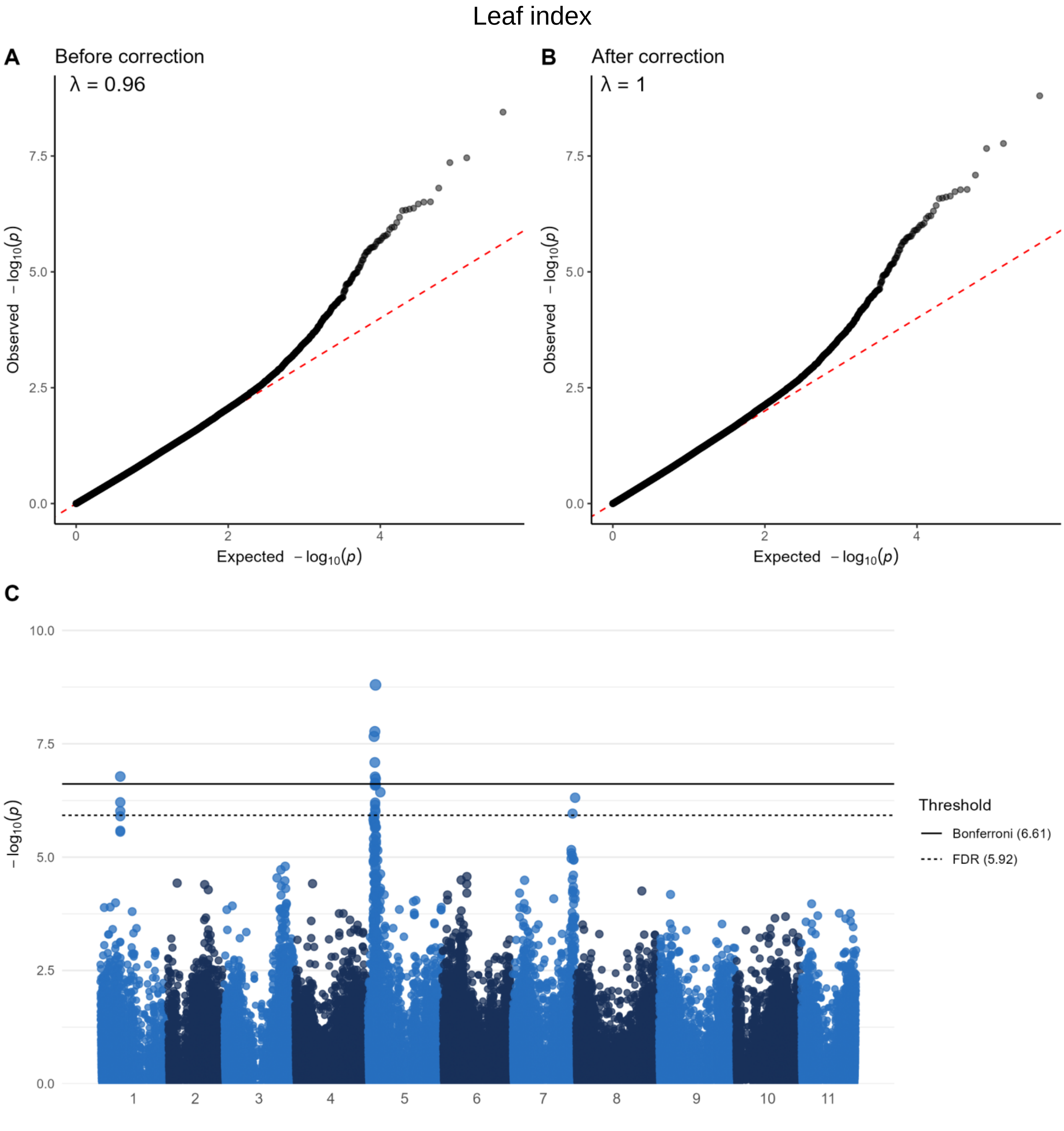

Figure S1U: QQ-plots of the p-values of the K model for leaf index before (**A**) and after (**B**) the correction by the inflation factor λ, and Manhattan plot (**C**) of the corrected p-values with the Bonferroni and FDR -log_{10}(p-value) thresholds

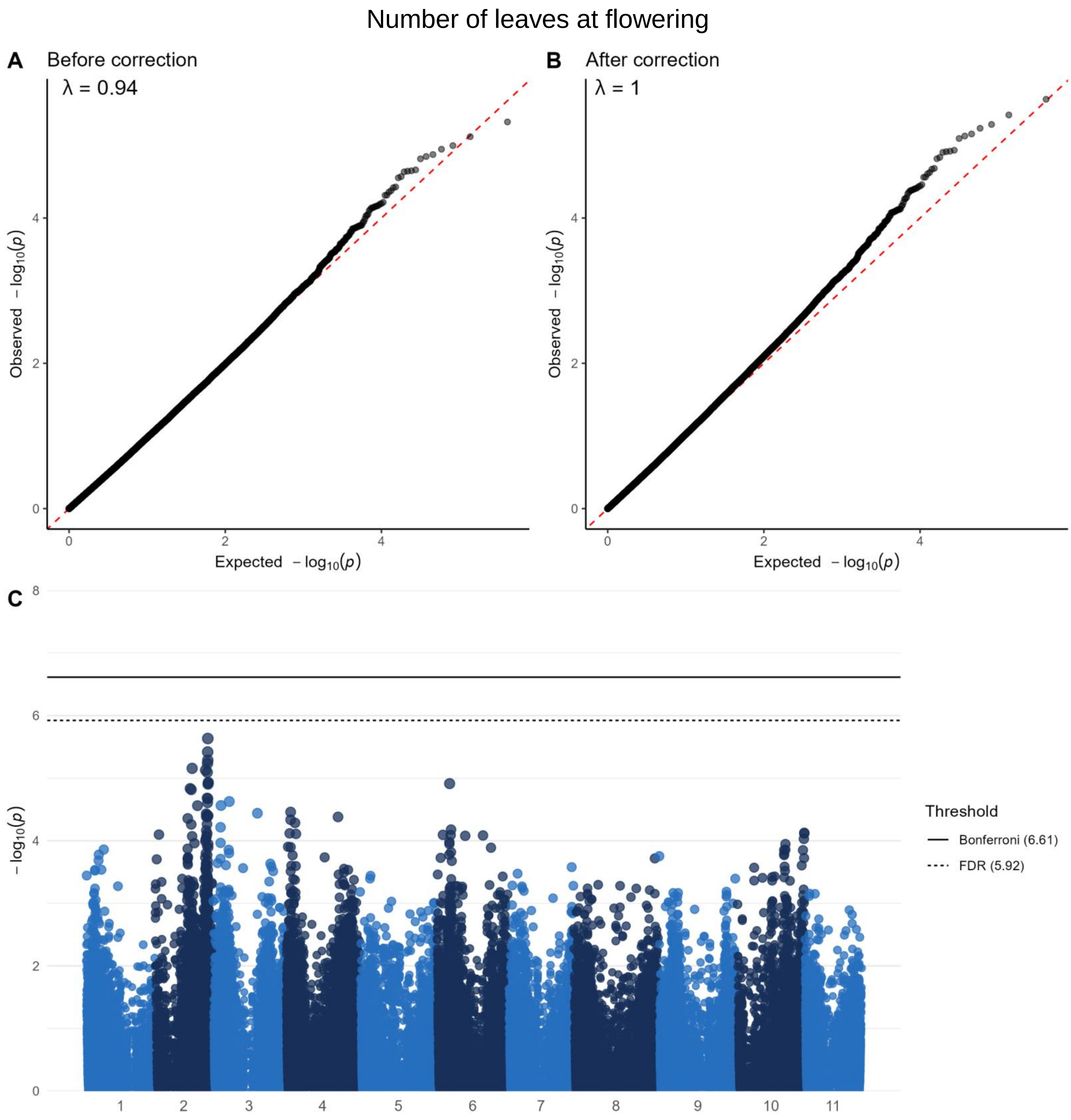

Figure S1V: QQ-plots of the p-values of the K model for number of leaves at flowering before (**A**) and after (**B**) the correction by the inflation factor λ, and Manhattan plot (**C**) of the corrected p-values with the Bonferroni and FDR -$\log_{10}$(p-value) thresholds

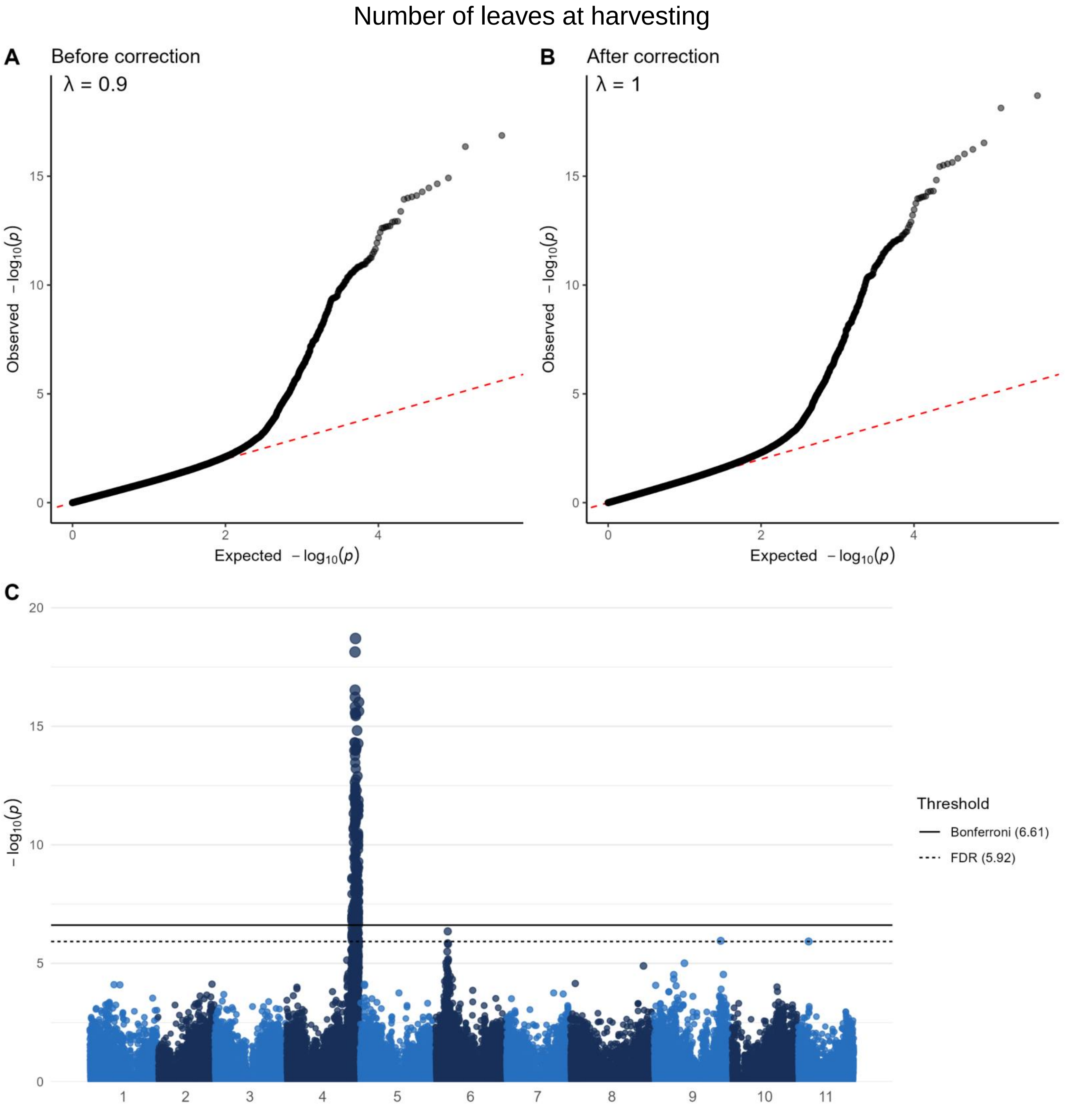

Figure S1W: QQ-plots of the p-values of the K model for number of leaves at harvesting before (**A**) and after (**B**) the correction by the inflation factor λ, and Manhattan plot (**C**) of the corrected p-values with the Bonferroni and FDR -$\log_{10}$(p-value) thresholds

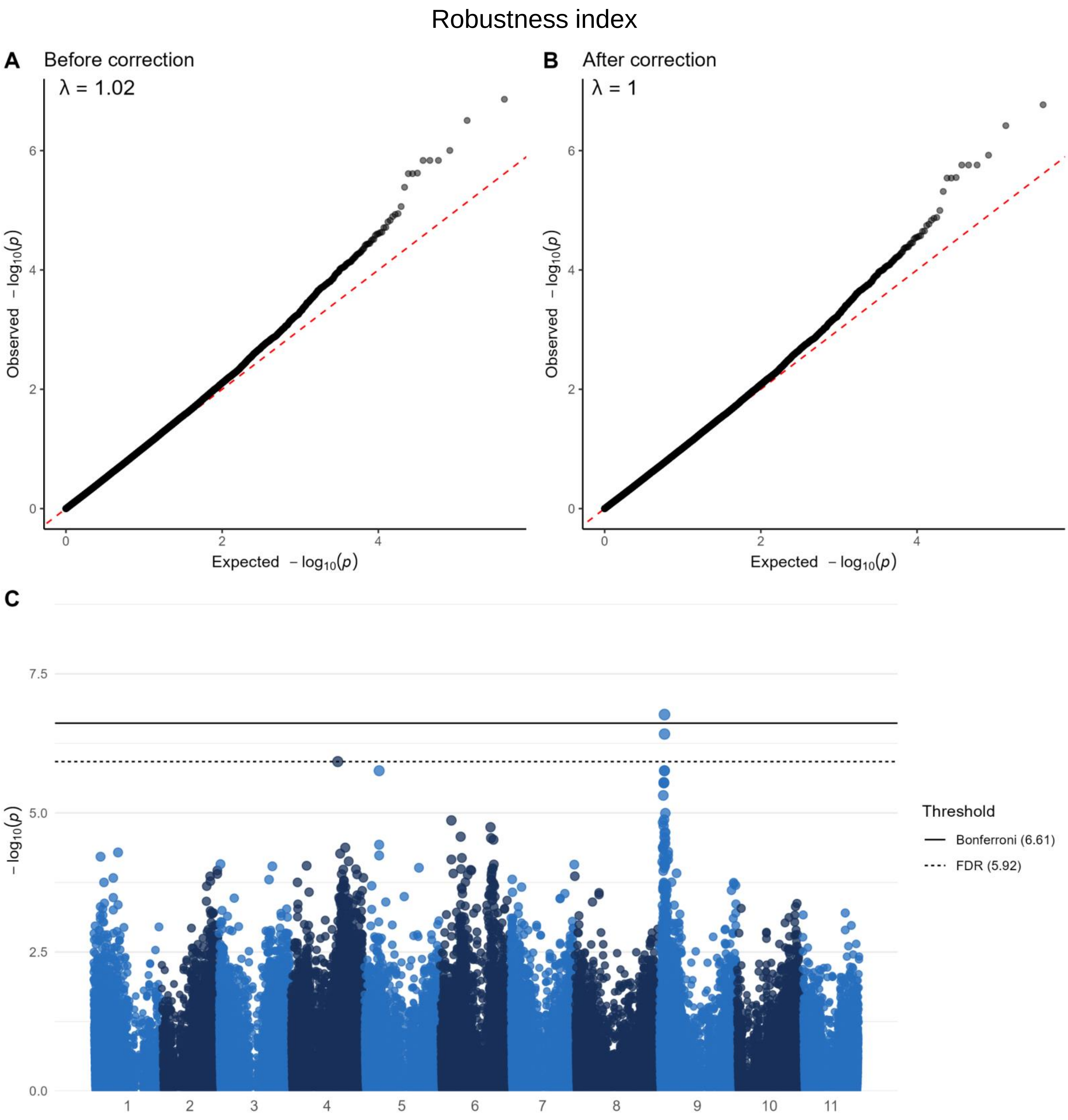

Figure S1X: QQ-plots of the p-values of the K model for robustness index before (**A**) and after (**B**) the correction by the inflation factor λ, and Manhattan plot (**C**) of the corrected p-values with the Bonferroni and FDR -$\log_{10}$(p-value) thresholds
