# Supplementary material for: Genome-wide association for agro-morphological traits in a triploid banana population with large chromosome rearrangements
Source: Hortic Res. 2024 Nov 6;12(2):uhae307. doi: 10.1093/hr/uhae307 (PMC11817881; doi:10.1093/hr/uhae307)
Supplement: Web_Material_uhae307 [file web_material_uhae307.zip › Figure_S2.pdf]

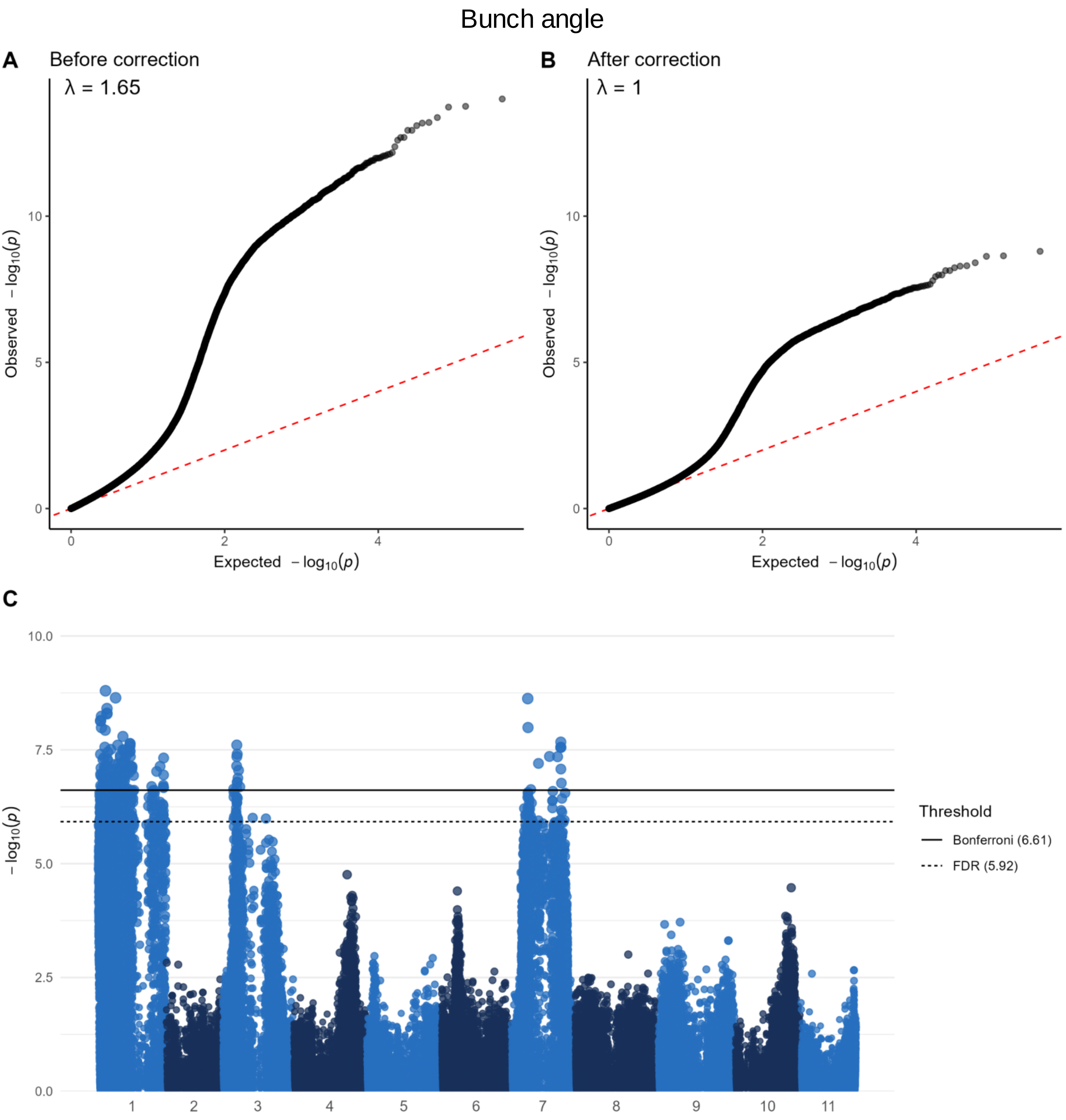

Figure S2A: QQ-plots of the p-values of the Kc model for bunch angle before (**A**) and after (**B**) the correction by the inflation factor λ, and Manhattan plot (**C**) of the corrected p-values with the Bonferroni and FDR -$\log_{10}$(p-value) thresholds

Peduncle diameter

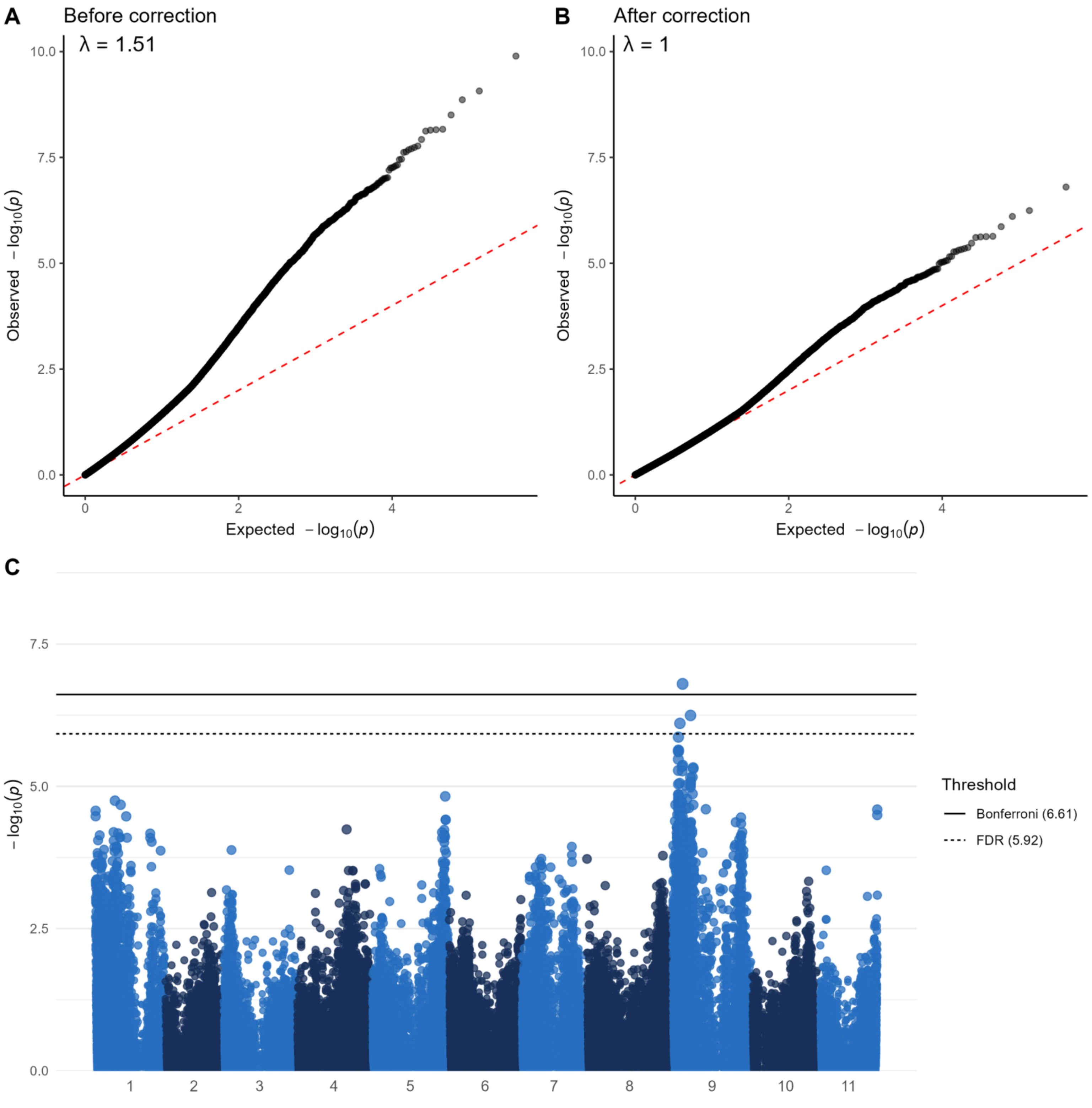

Figure S2B: QQ-plots of the p-values of the Kc model for peduncle diameter before (**A**) and after (**B**) the correction by the inflation factor λ, and Manhattan plot (**C**) of the corrected p-values with the Bonferroni and FDR -log_{10}(p-value) thresholds

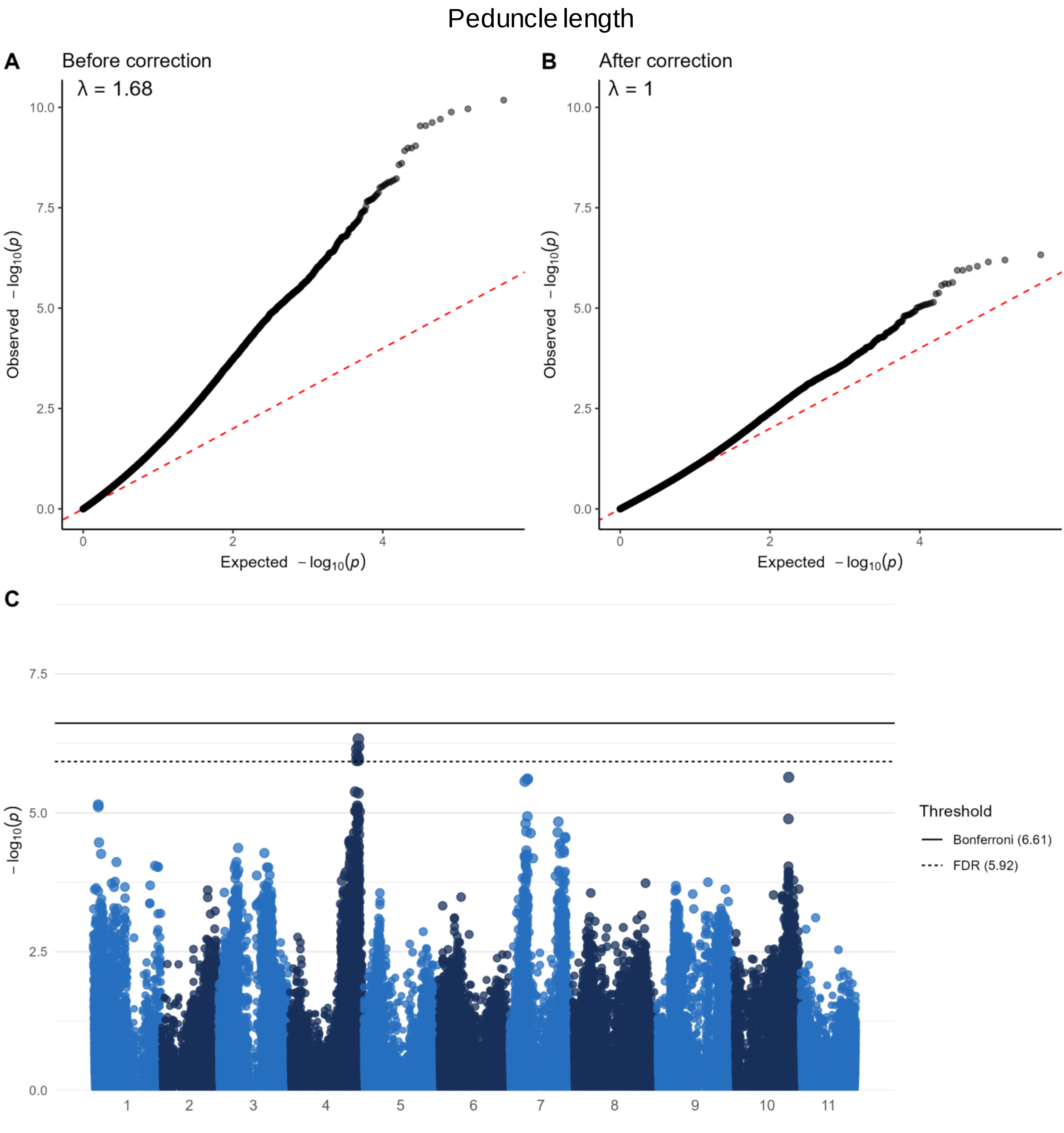

Figure S2C: QQ-plots of the p-values of the Kc model for peduncle length before (**A**) and after (**B**) the correction by the inflation factor λ, and Manhattan plot (**C**) of the corrected p-values with the Bonferroni and FDR -$\log_{10}$(p-value) thresholds

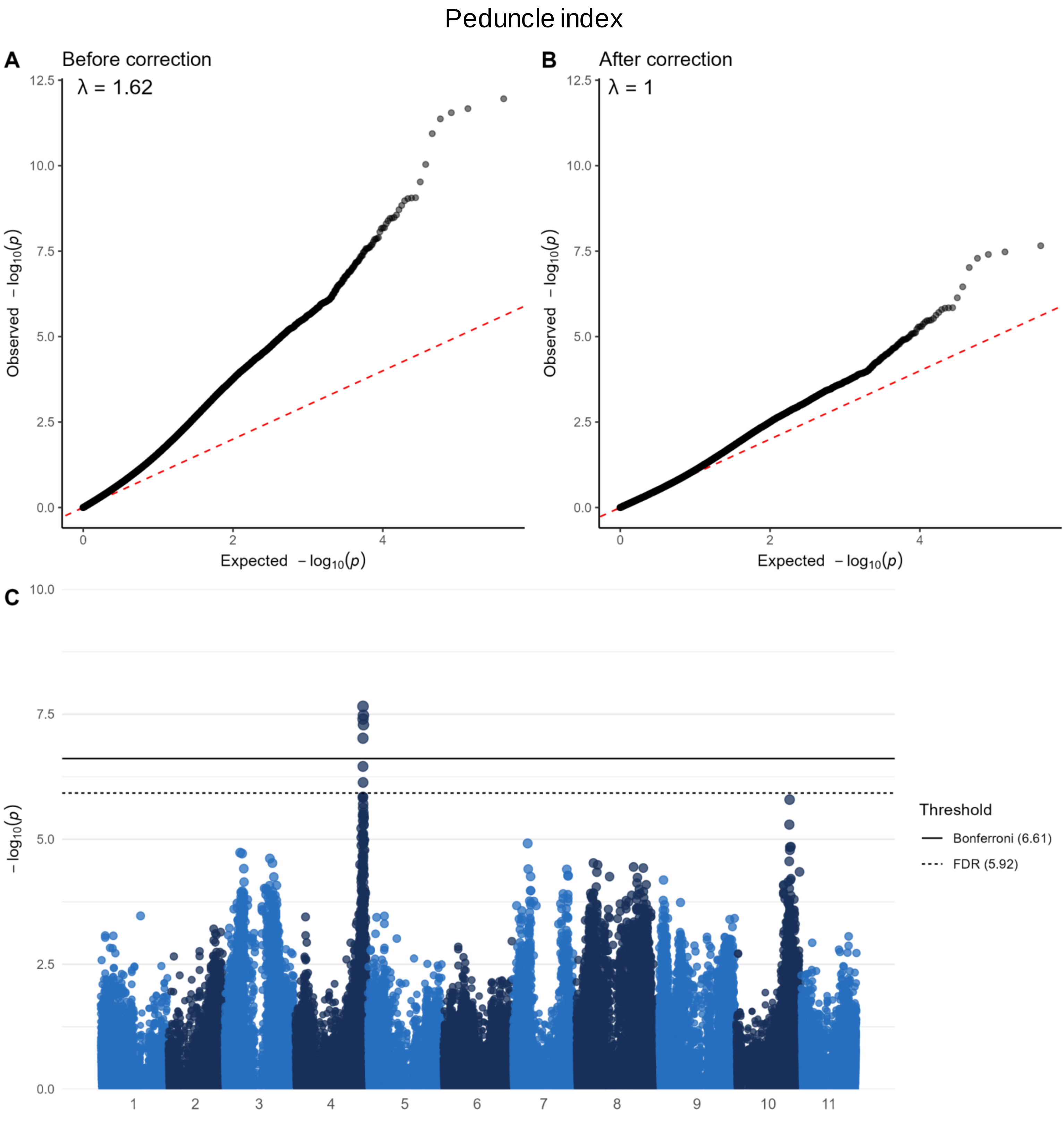

Figure S2D: QQ-plots of the p-values of the Kc model for peduncle index before (**A**) and after (**B**) the correction by the inflation factor λ, and Manhattan plot (**C**) of the corrected p-values with the Bonferroni and FDR -$\log_{10}$(p-value) thresholds

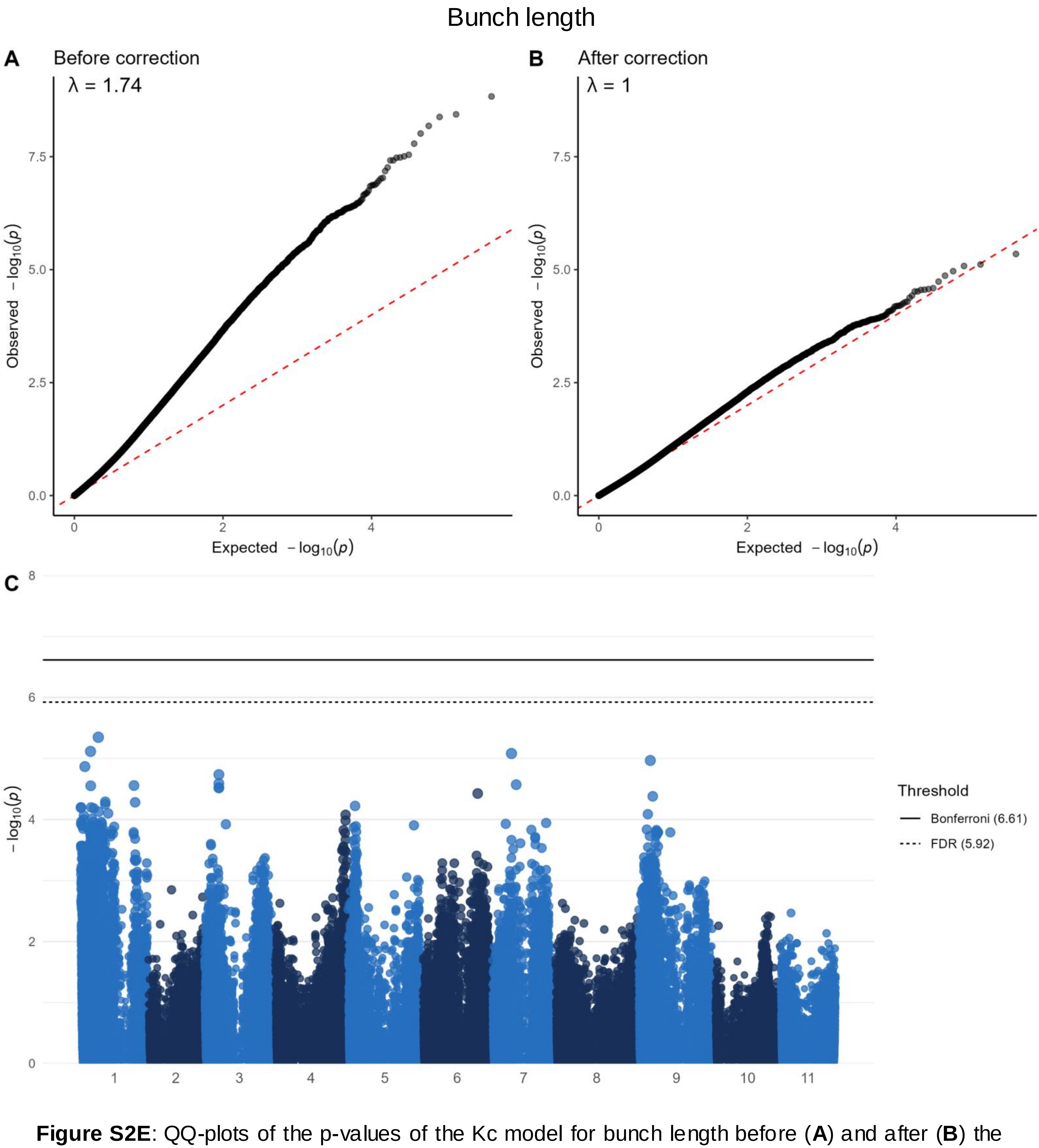

Figure S2E: QQ-plots of the p-values of the Kc model for bunch length before (**A**) and after (**B**) the correction by the inflation factor λ, and Manhattan plot (**C**) of the corrected p-values with the Bonferroni and FDR -$\log_{10}$(p-value) thresholds

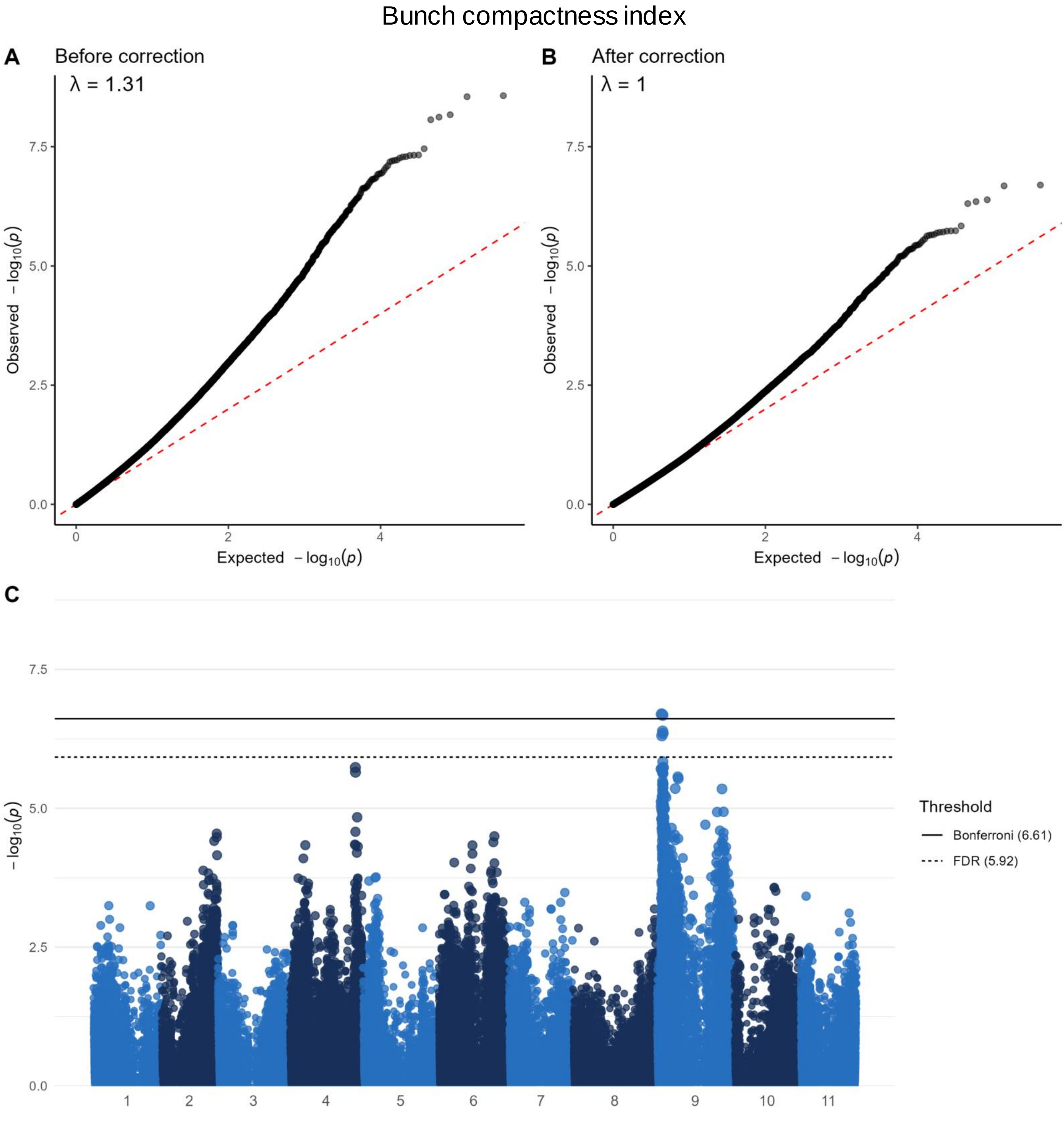

Figure S2F: QQ-plots of the p-values of the Kc model for bunch compactness index before (**A**) and after (**B**) the correction by the inflation factor λ, and Manhattan plot (**C**) of the corrected p-values with the Bonferroni and FDR $-\log_{10}$(p-value) thresholds

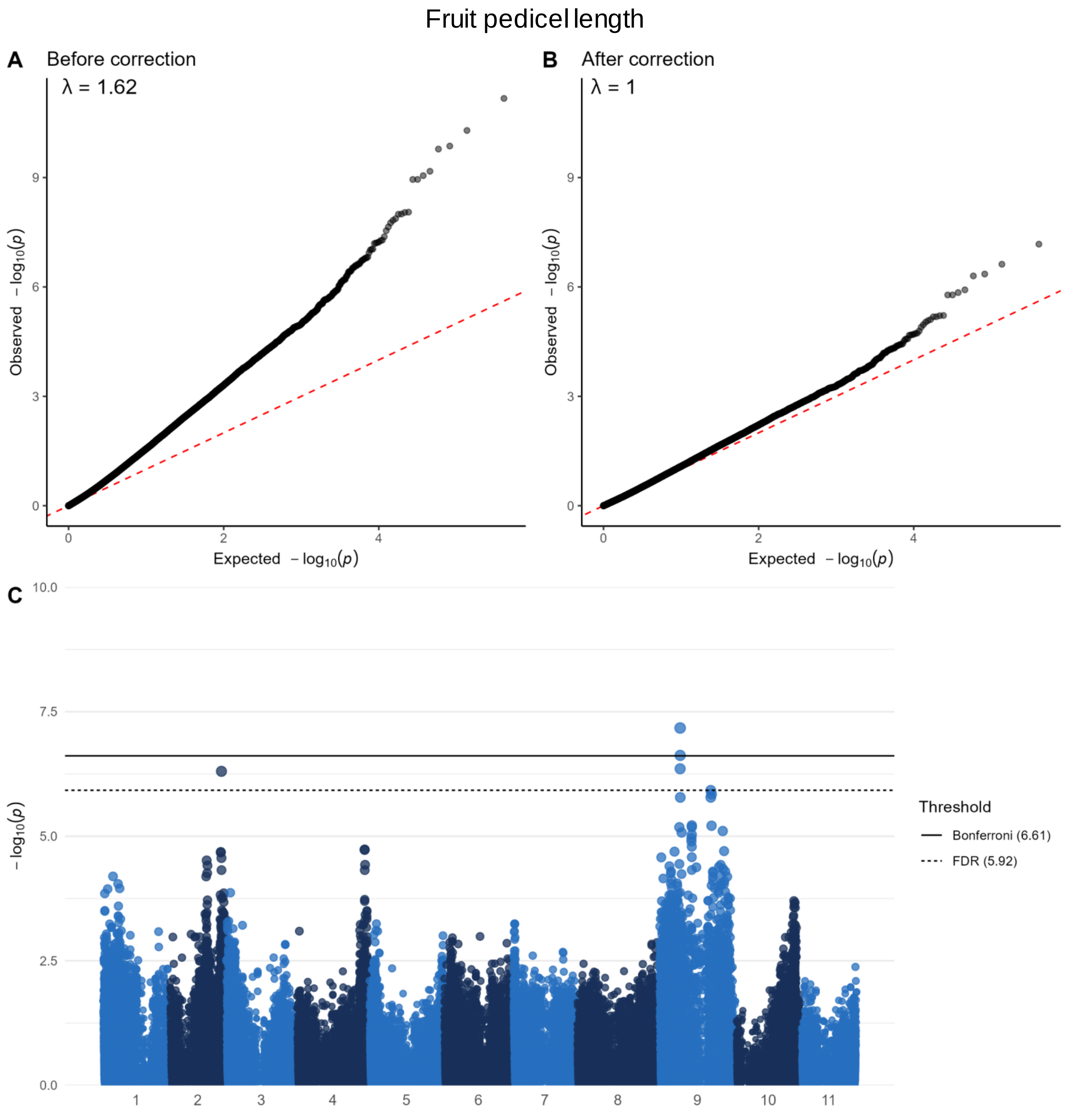

Figure S2G: QQ-plots of the p-values of the Kc model for fruit pedicel length before (**A**) and after (**B**) the correction by the inflation factor λ, and Manhattan plot (**C**) of the corrected p-values with the Bonferroni and FDR -$\log_{10}$(p-value) thresholds

Fruit pedicel diameter

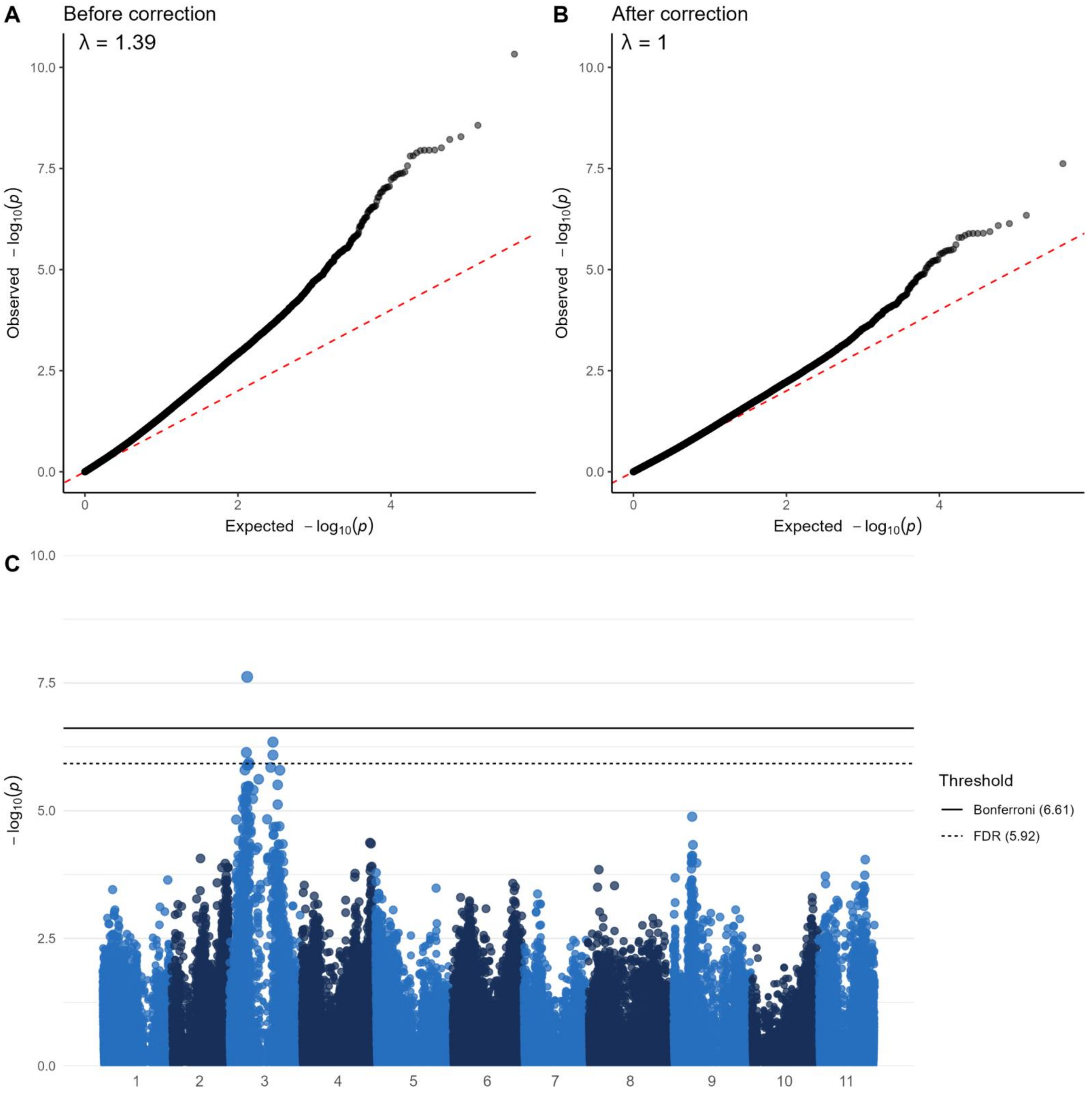

Figure S2H: QQ-plots of the p-values of the Kc model for fruit pedicel diameter before (**A**) and after (**B**) the correction by the inflation factor λ, and Manhattan plot (**C**) of the corrected p-values with the Bonferroni and FDR -$\log_{10}$(p-value) thresholds

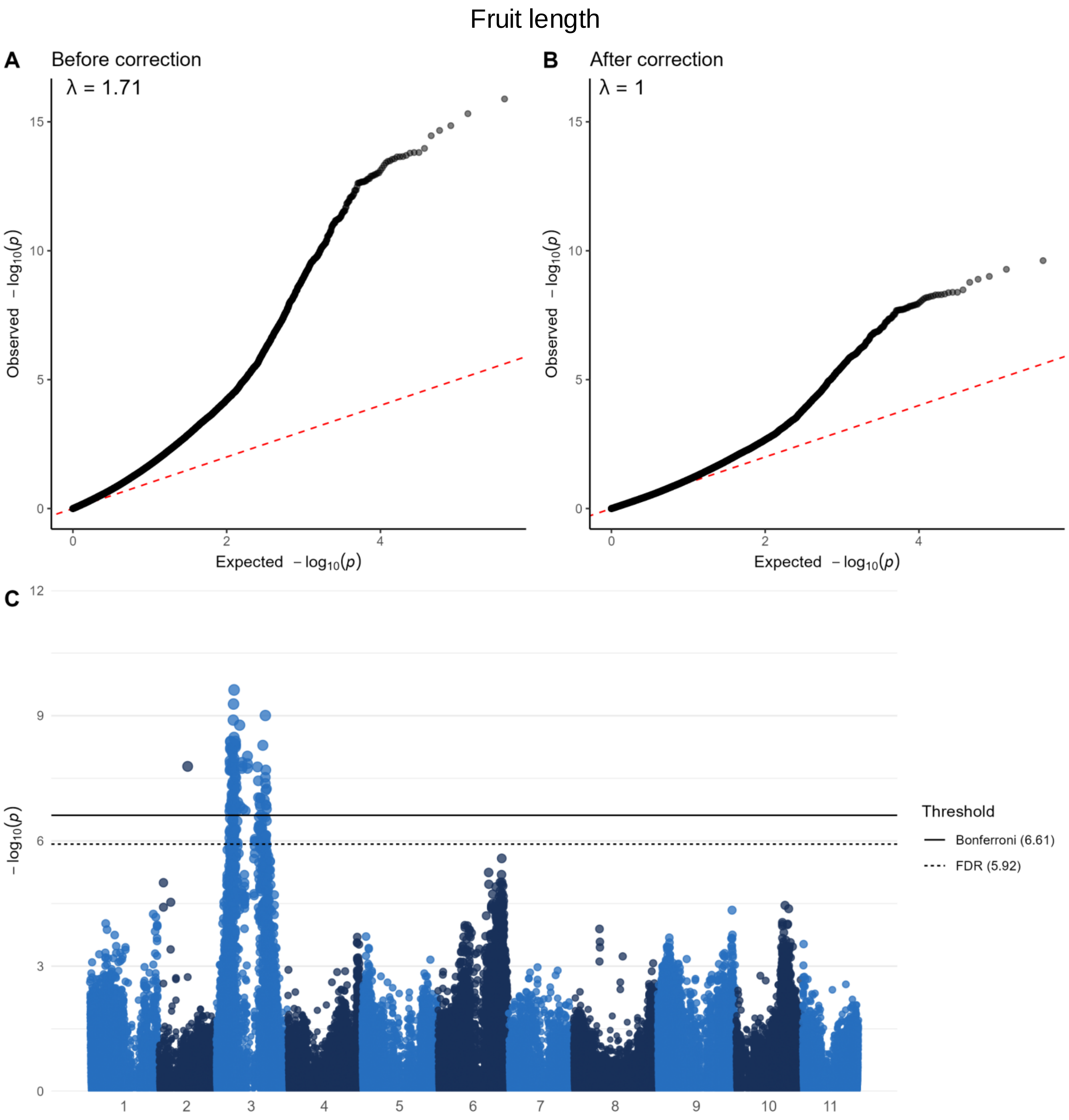

Figure S2I: QQ-plots of the p-values of the Kc model for fruit length before (**A**) and after (**B**) the correction by the inflation factor λ, and Manhattan plot (**C**) of the corrected p-values with the Bonferroni and FDR -$\log_{10}$(p-value) thresholds

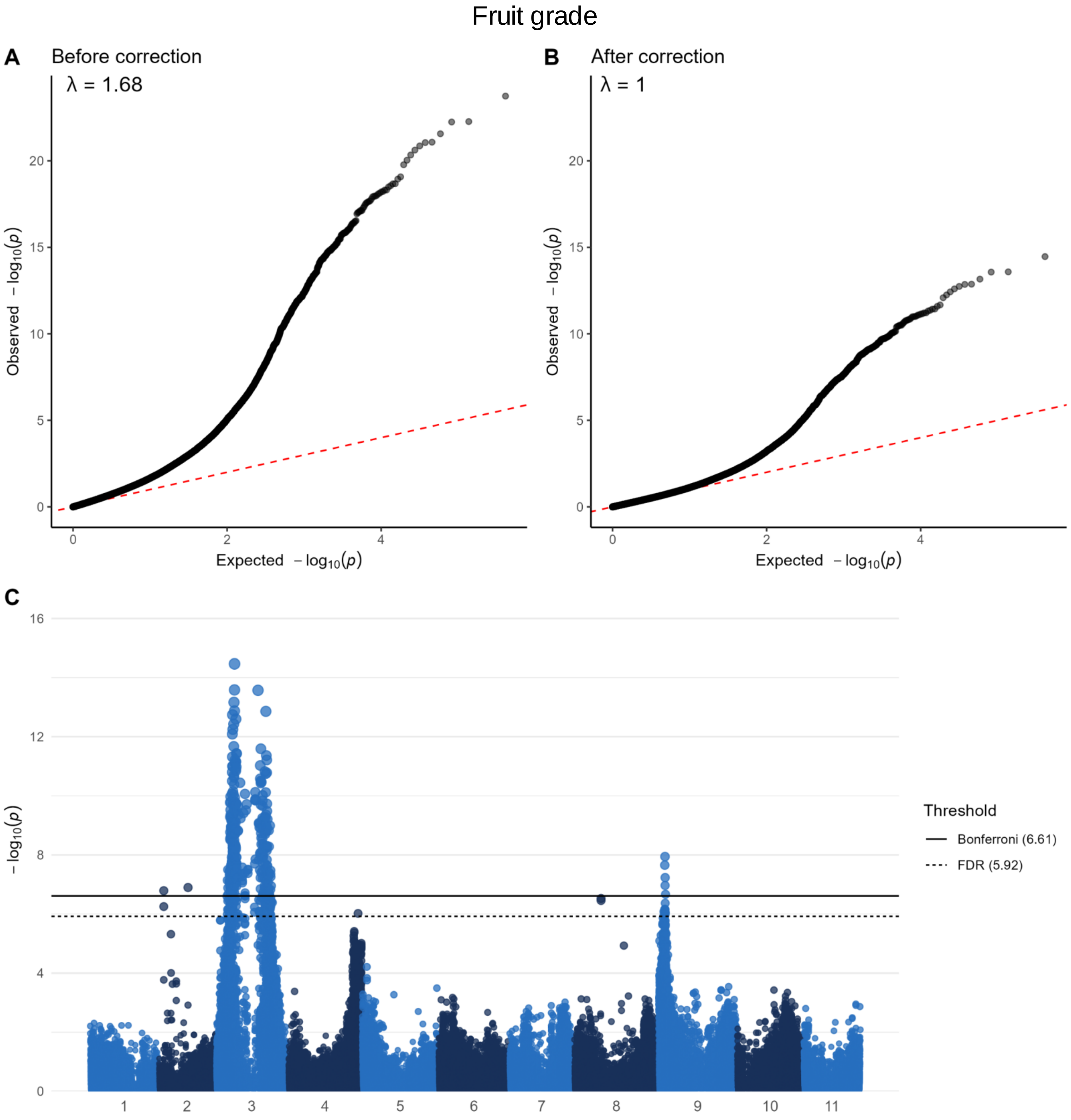

Figure S2J: QQ-plots of the p-values of the Kc model for fruit grade before (**A**) and after (**B**) the correction by the inflation factor λ, and Manhattan plot (**C**) of the corrected p-values with the Bonferroni and FDR -$\log_{10}$(p-value) thresholds

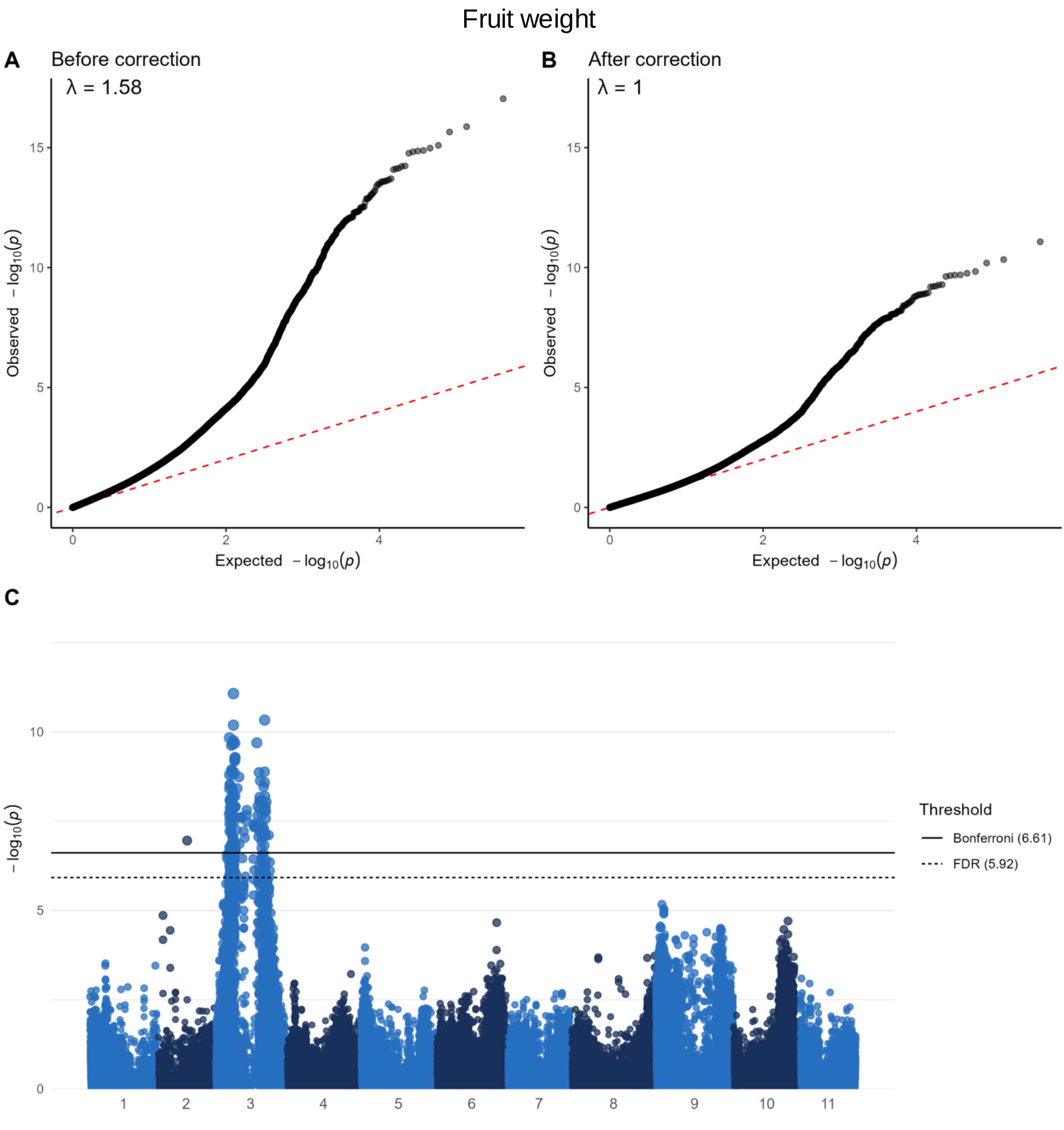

Figure S2K: QQ-plots of the p-values of the Kc model for fruit weight before (**A**) and after (**B**) the correction by the inflation factor λ, and Manhattan plot (**C**) of the corrected p-values with the Bonferroni and FDR -$\log_{10}$(p-value) thresholds

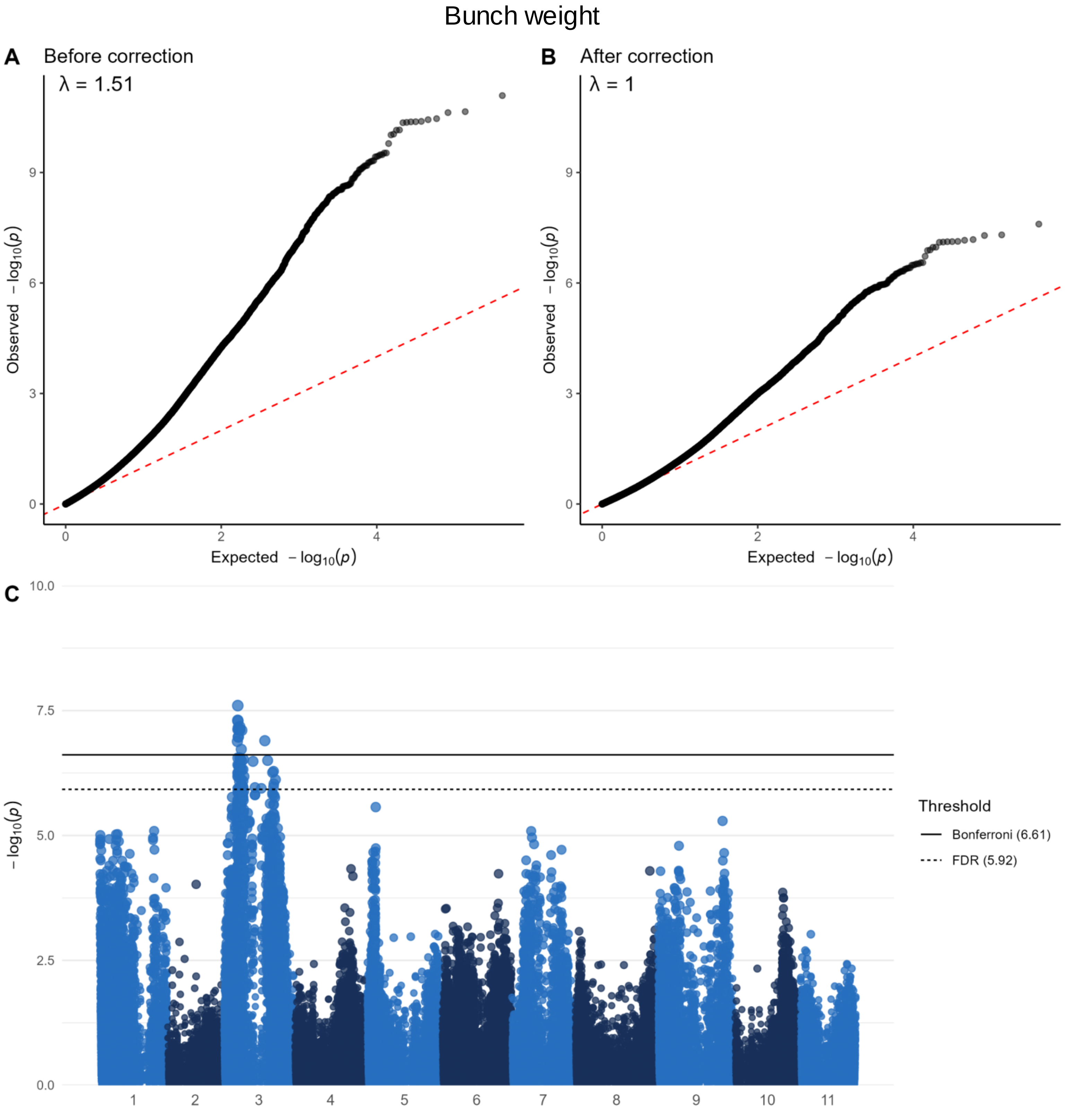

Figure S2L: QQ-plots of the p-values of the Kc model for fruit weight before (**A**) and after (**B**) the correction by the inflation factor λ, and Manhattan plot (**C**) of the corrected p-values with the Bonferroni and FDR $-\log_{10}$(p-value) thresholds

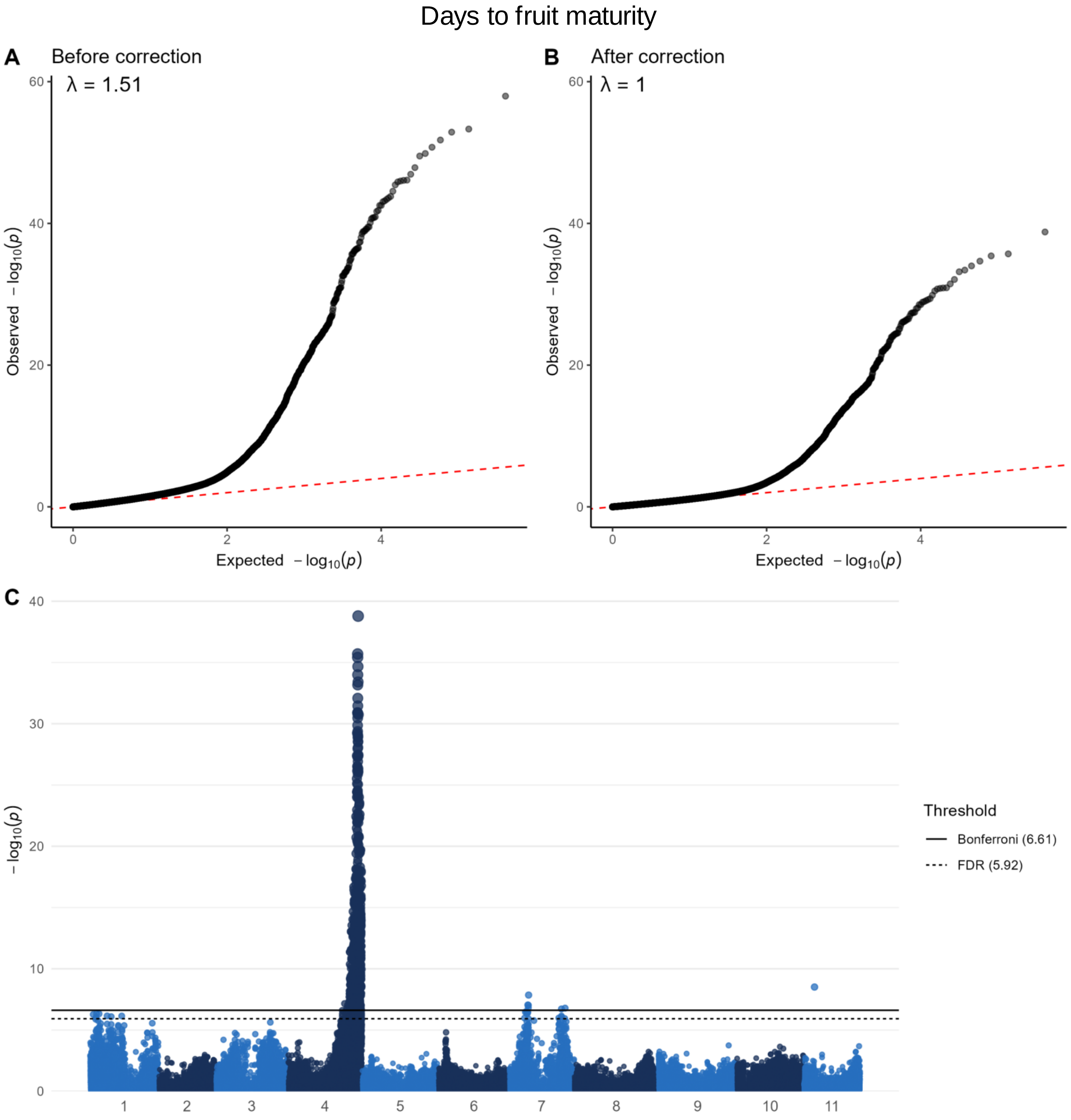

Figure S2M: QQ-plots of the p-values of the Kc model for days to fruit maturity before (**A**) and after (**B**) the correction by the inflation factor λ, and Manhattan plot (**C**) of the corrected p-values with the Bonferroni and FDR -$\log_{10}$(p-value) thresholds

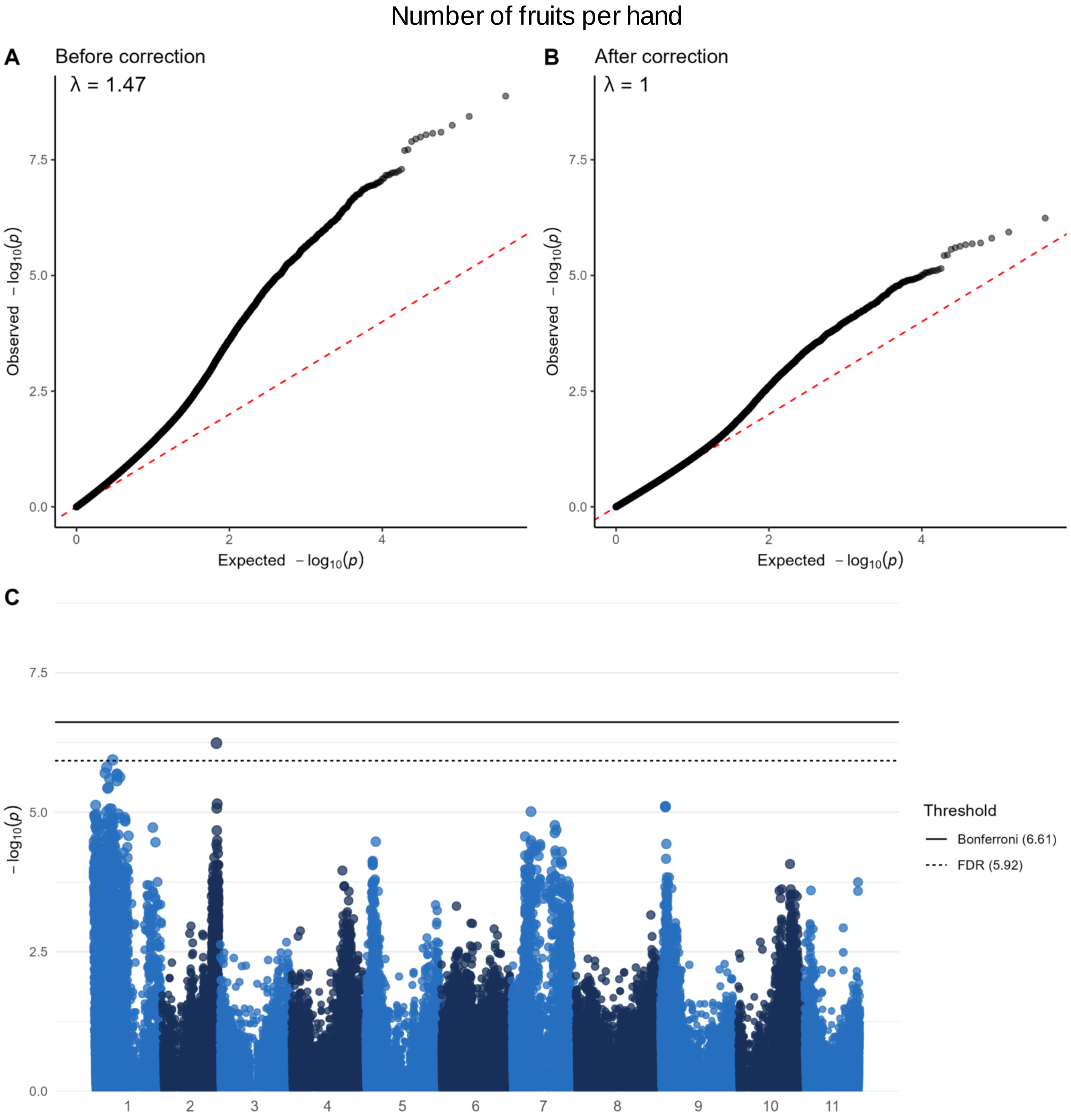

Figure S2N: QQ-plots of the p-values of the Kc model for number of fruits per hand before (**A**) and after (**B**) the correction by the inflation factor λ, and Manhattan plot (**C**) of the corrected p-values with the Bonferroni and FDR -$\log_{10}$(p-value) thresholds

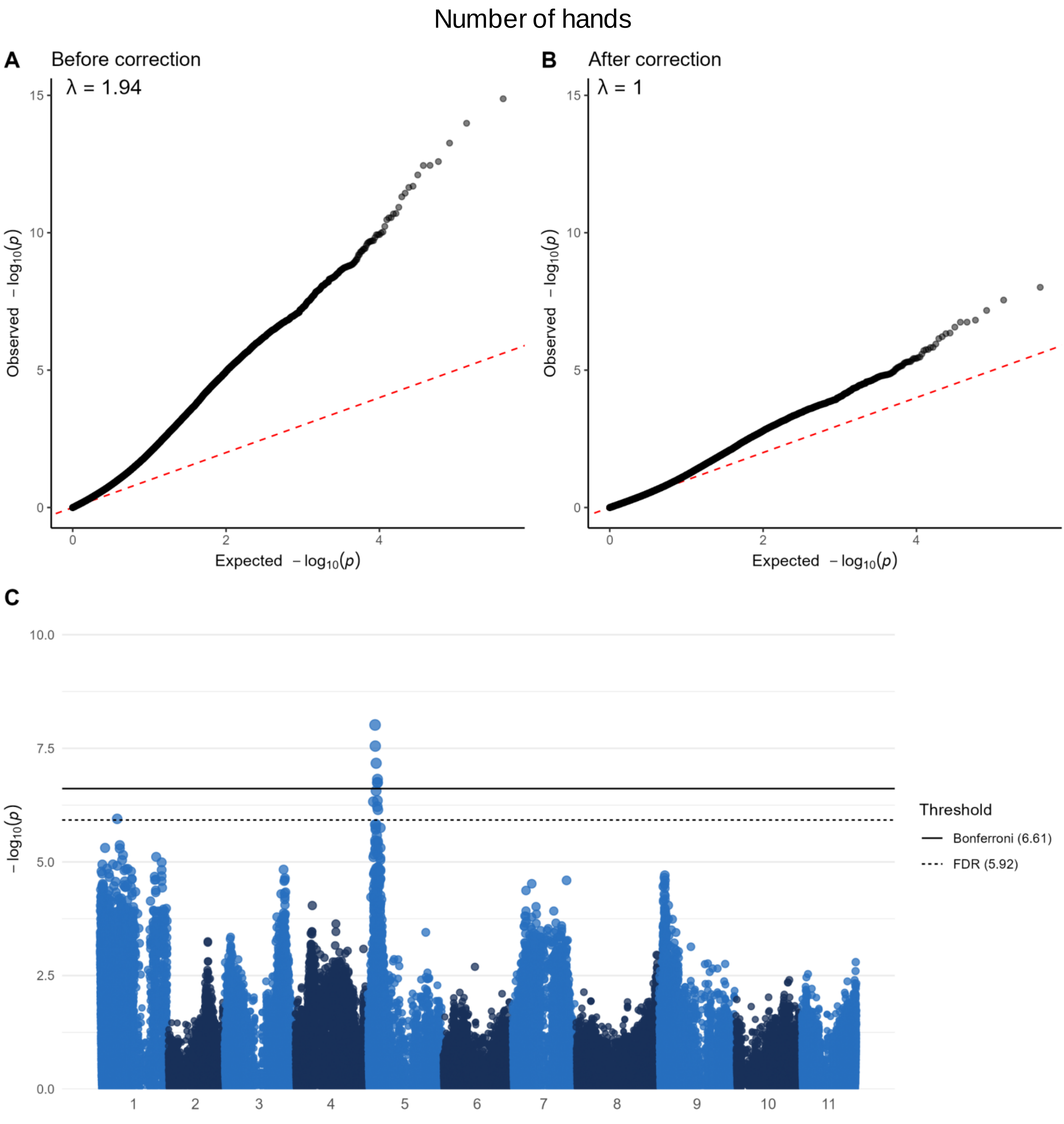

Figure S2O: QQ-plots of the p-values of the Kc model for number of hands before (**A**) and after (**B**) the correction by the inflation factor λ, and Manhattan plot (**C**) of the corrected p-values with the Bonferroni and FDR $-\log_{10}$(p-value) thresholds

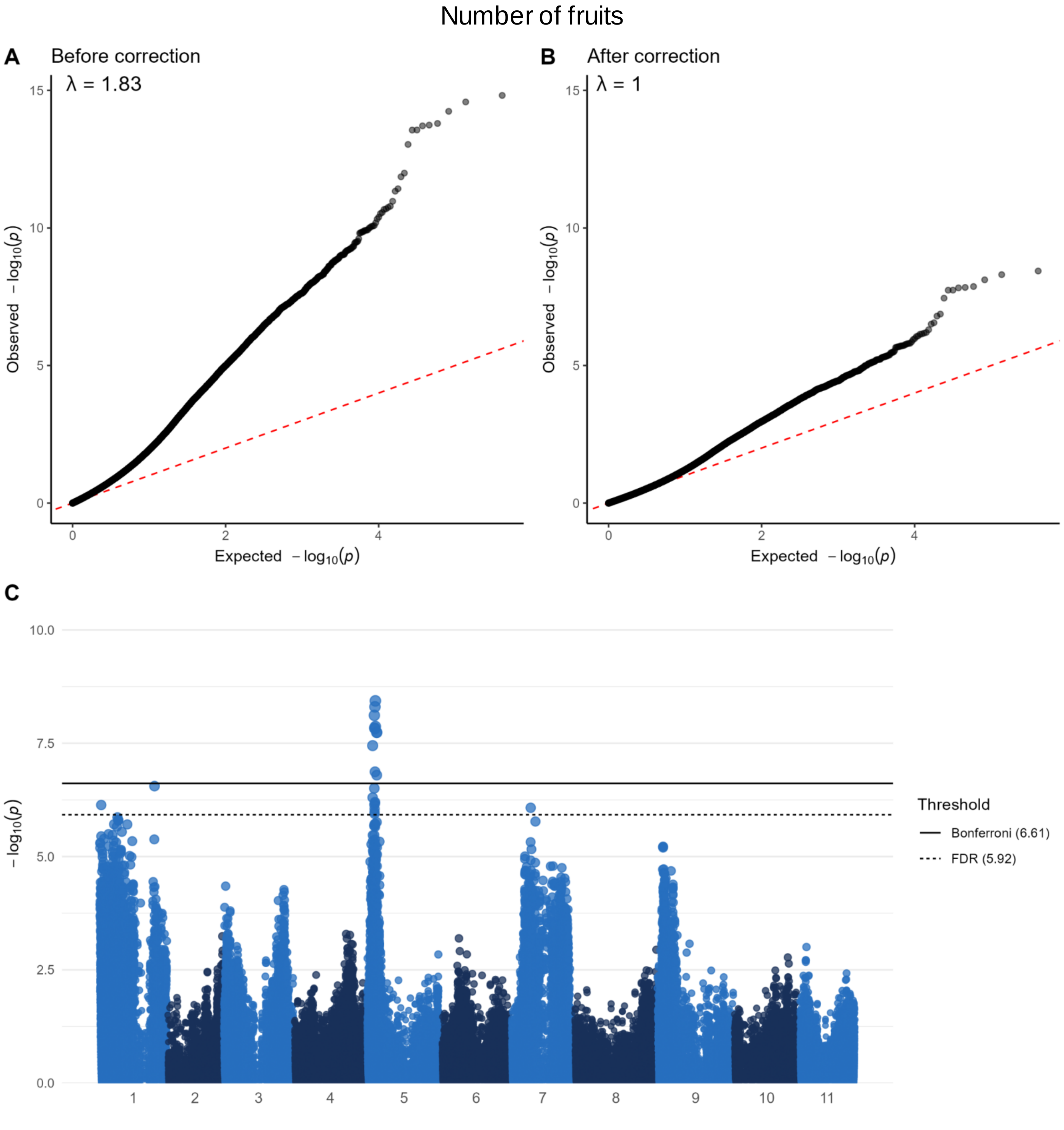

Figure S2P: QQ-plots of the p-values of the Kc model for number of fruits before (**A**) and after (**B**) the correction by the inflation factor λ, and Manhattan plot (**C**) of the corrected p-values with the Bonferroni and FDR -$\log_{10}$(p-value) thresholds

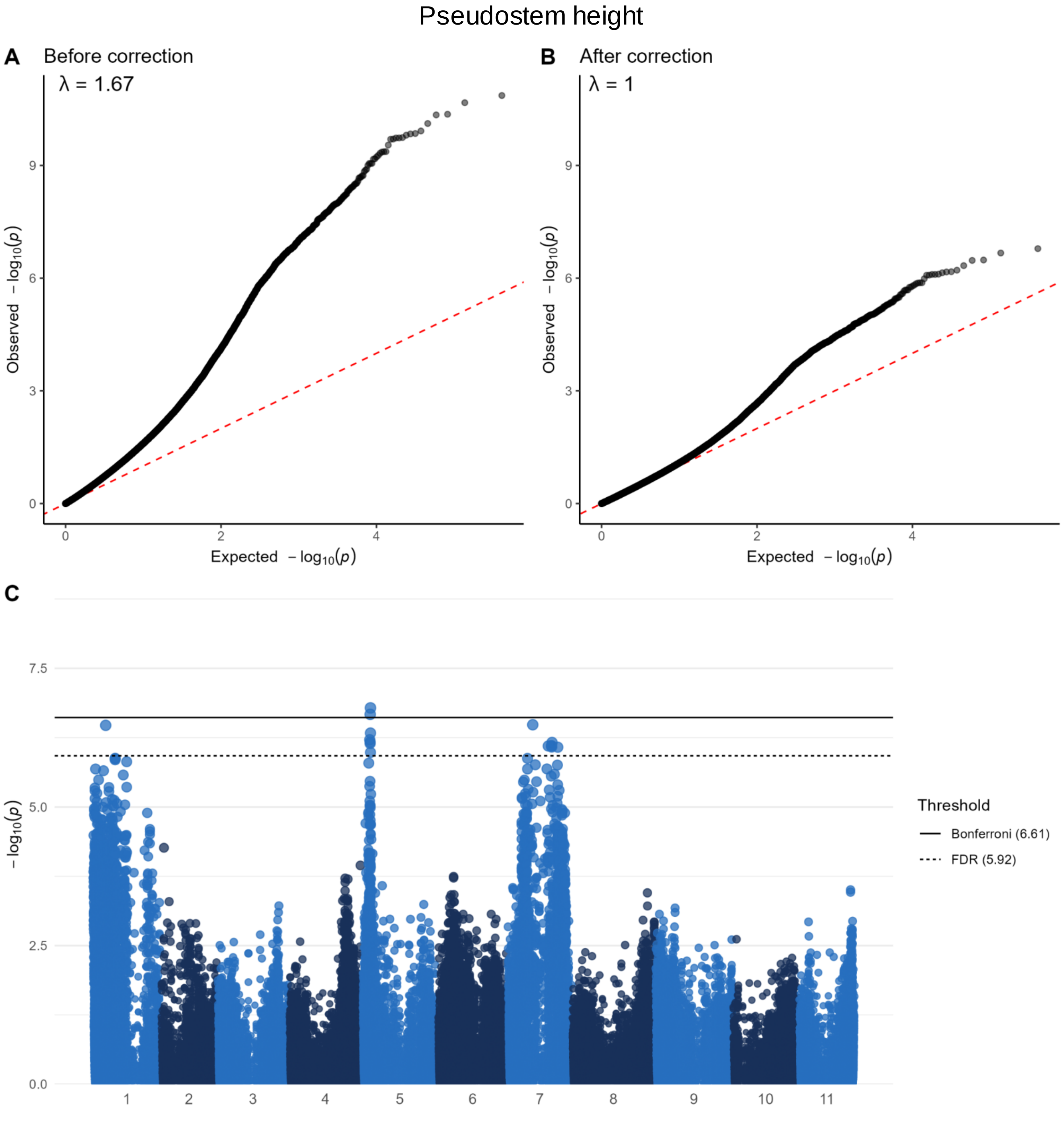

Figure S2Q: QQ-plots of the p-values of the Kc model for pseudostem height before (**A**) and after (**B**) the correction by the inflation factor λ, and Manhattan plot (**C**) of the corrected p-values with the Bonferroni and FDR -$\log_{10}$(p-value) thresholds

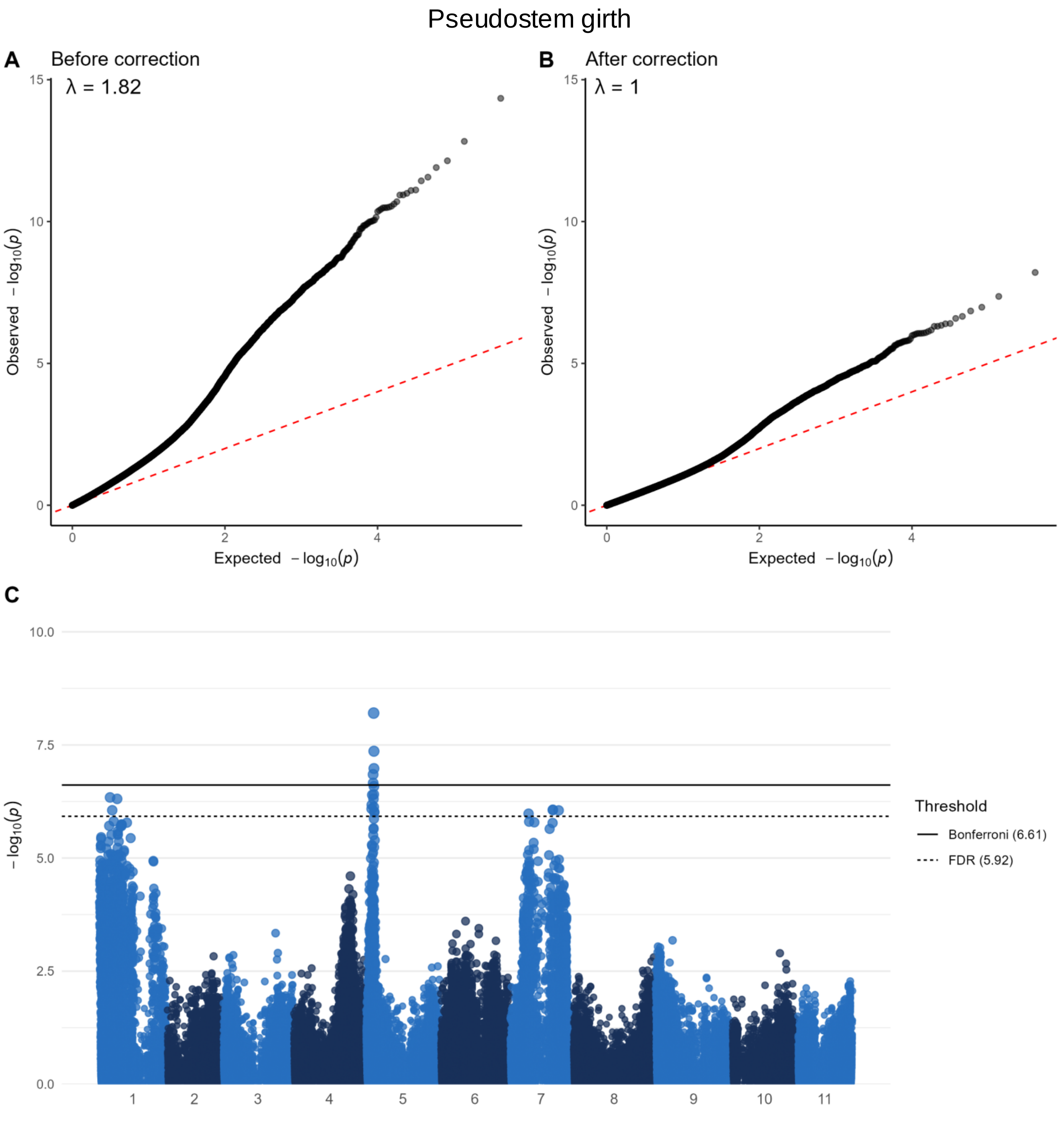

Figure S2R: QQ-plots of the p-values of the Kc model for pseudostem girth before (**A**) and after (**B**) the correction by the inflation factor λ, and Manhattan plot (**C**) of the corrected p-values with the Bonferroni and FDR -$\log_{10}$(p-value) thresholds

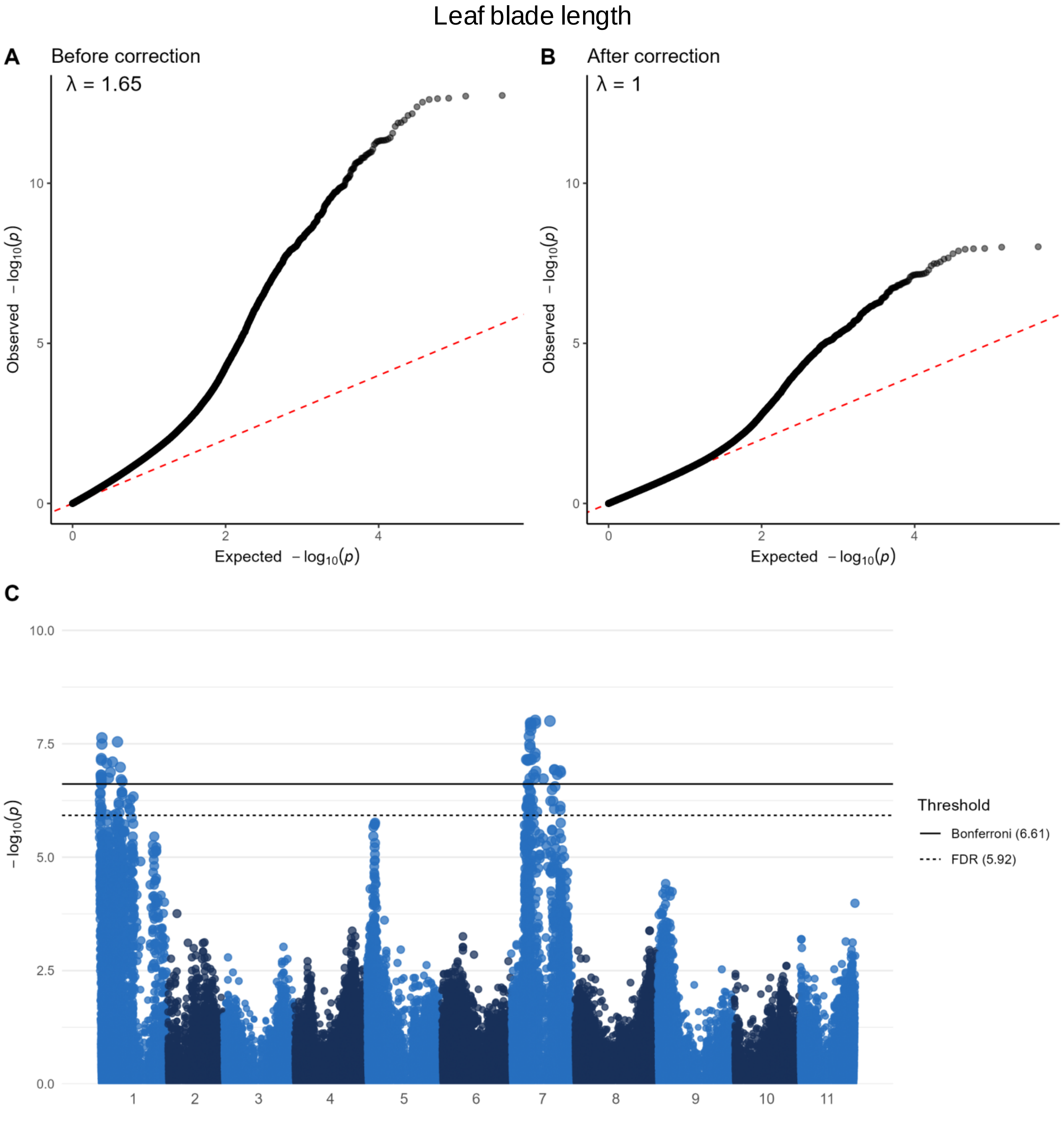

Figure S2S: QQ-plots of the p-values of the Kc model for leaf blade length before (**A**) and after (**B**) the correction by the inflation factor λ, and Manhattan plot (**C**) of the corrected p-values with the Bonferroni and FDR -$\log_{10}$(p-value) thresholds

Leaf blade width

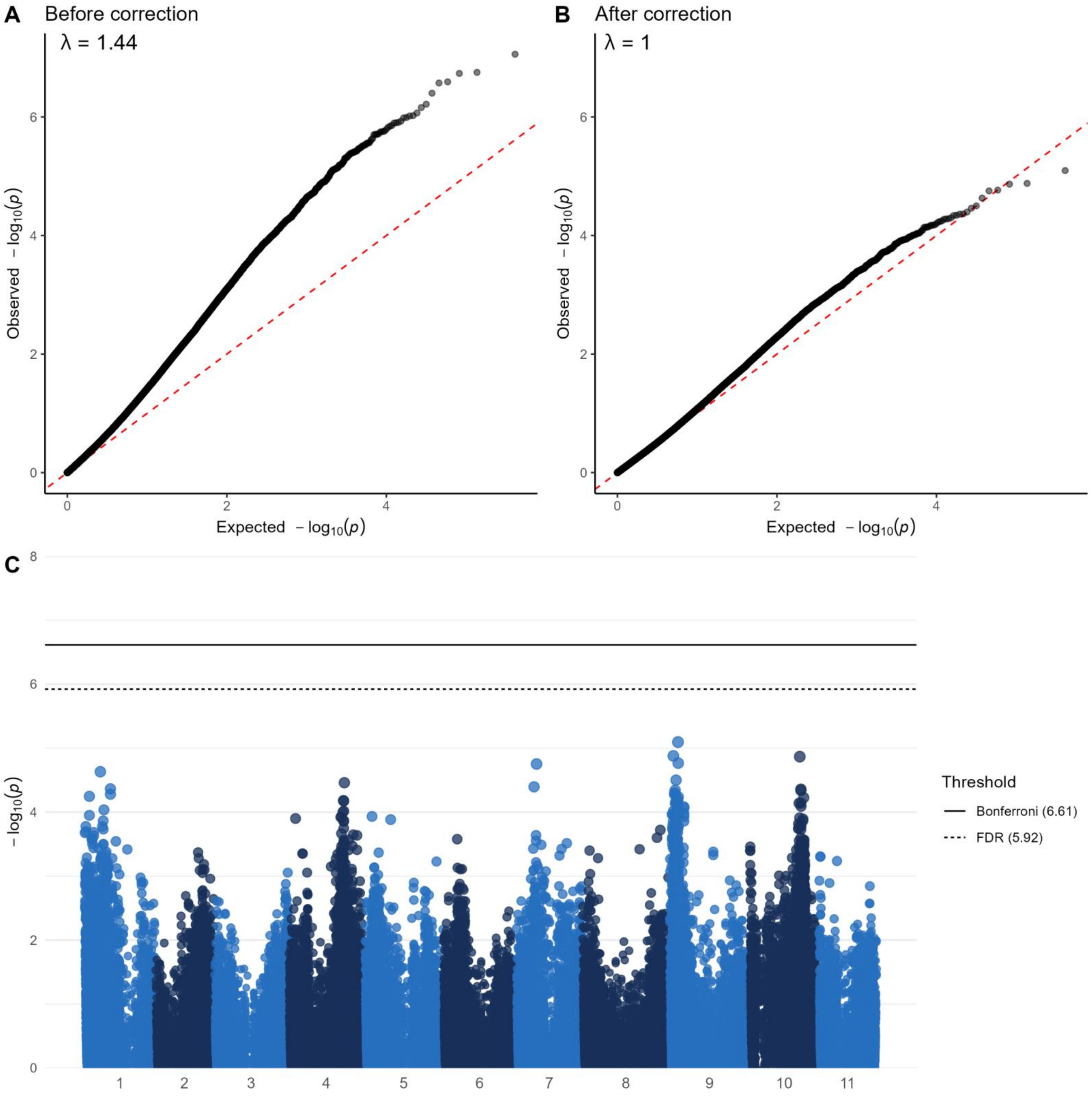

Figure S2T: QQ-plots of the p-values of the Kc model for leaf blade width before (**A**) and after (**B**) the correction by the inflation factor λ, and Manhattan plot (**C**) of the corrected p-values with the Bonferroni and FDR -$\log_{10}$(p-value) thresholds

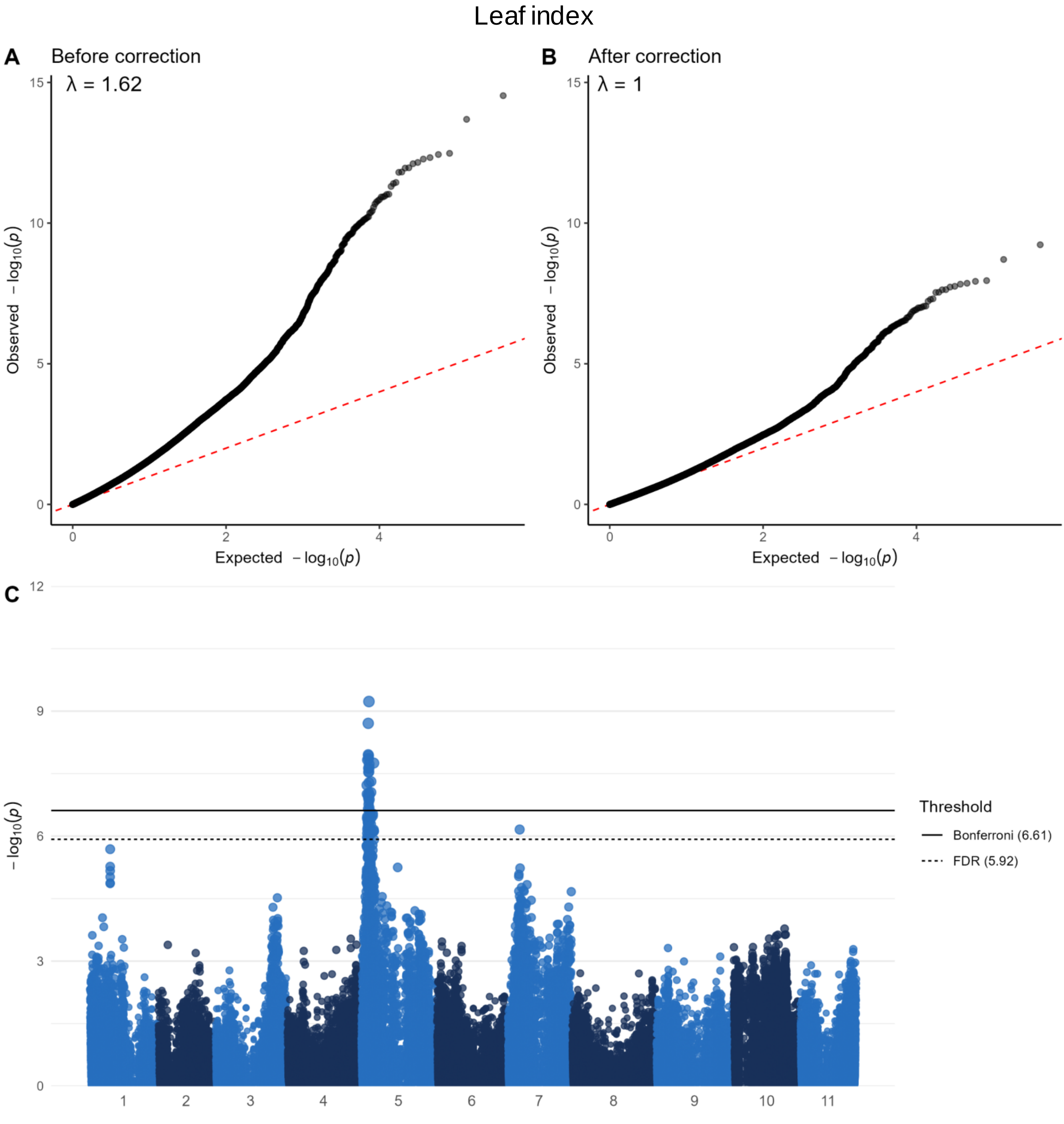

Figure S2U: QQ-plots of the p-values of the Kc model for leaf index before (**A**) and after (**B**) the correction by the inflation factor λ, and Manhattan plot (**C**) of the corrected p-values with the Bonferroni and FDR -$\log_{10}$(p-value) thresholds

Number of leaves at flowering

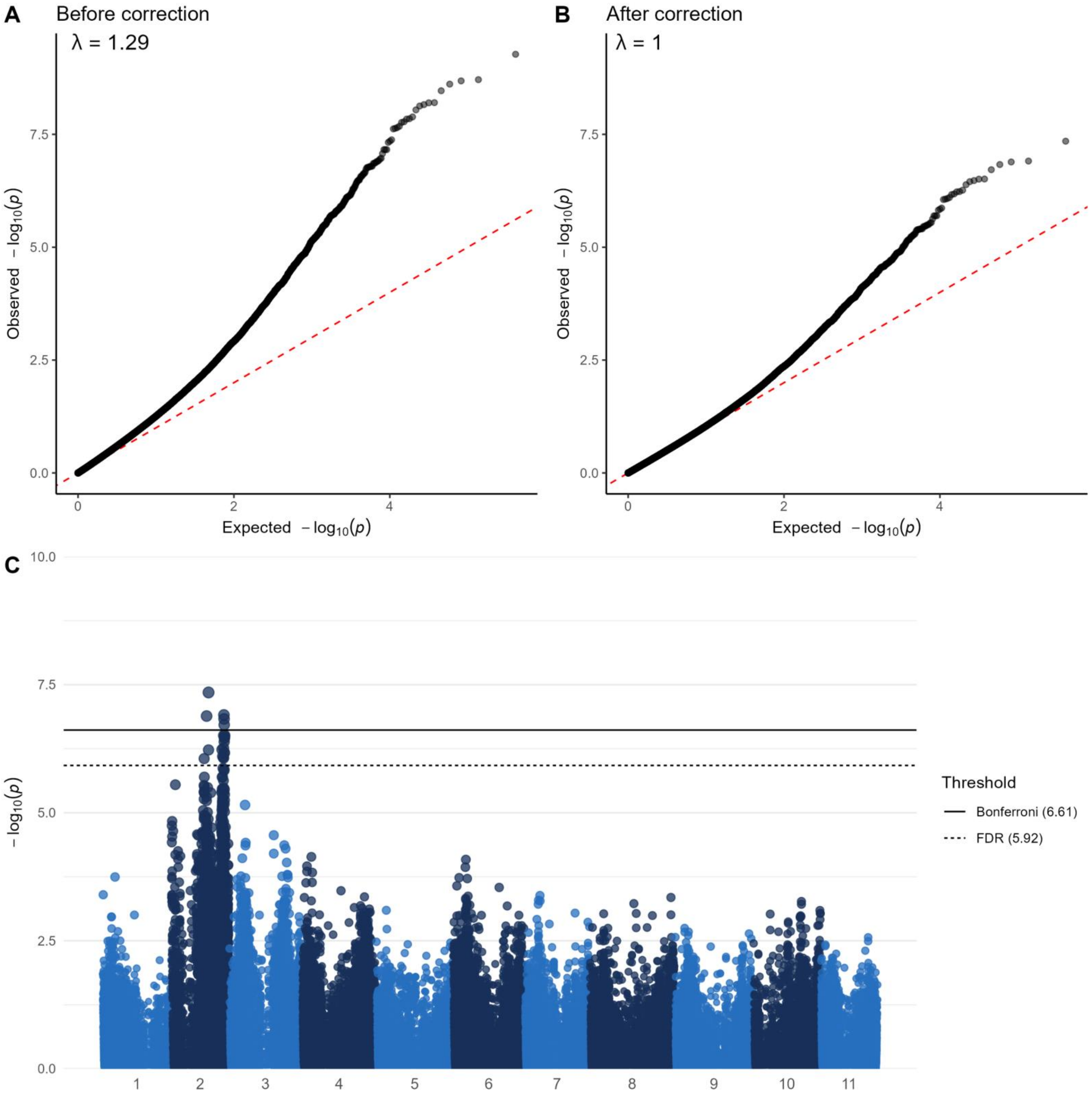

Figure S2V: QQ-plots of the p-values of the Kc model for number of leaves at flowering before (**A**) and after (**B**) the correction by the inflation factor λ, and Manhattan plot (**C**) of the corrected p-values with the Bonferroni and FDR -$\log_{10}$(p-value) thresholds

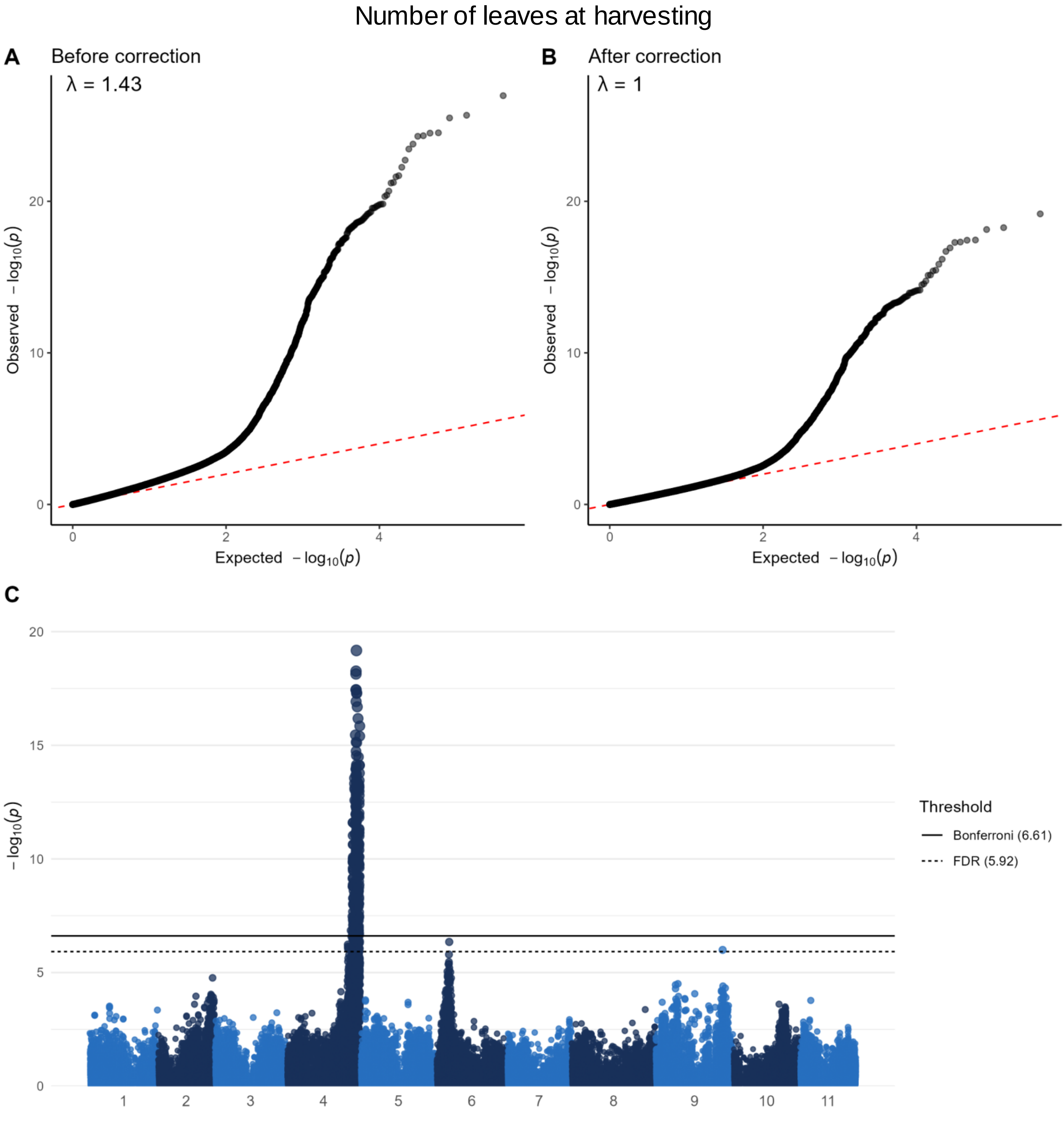

Figure S2W: QQ-plots of the p-values of the Kc model for number of leaves at harvesting before (**A**) and after (**B**) the correction by the inflation factor λ, and Manhattan plot (**C**) of the corrected p-values with the Bonferroni and FDR -$\log_{10}$(p-value) thresholds

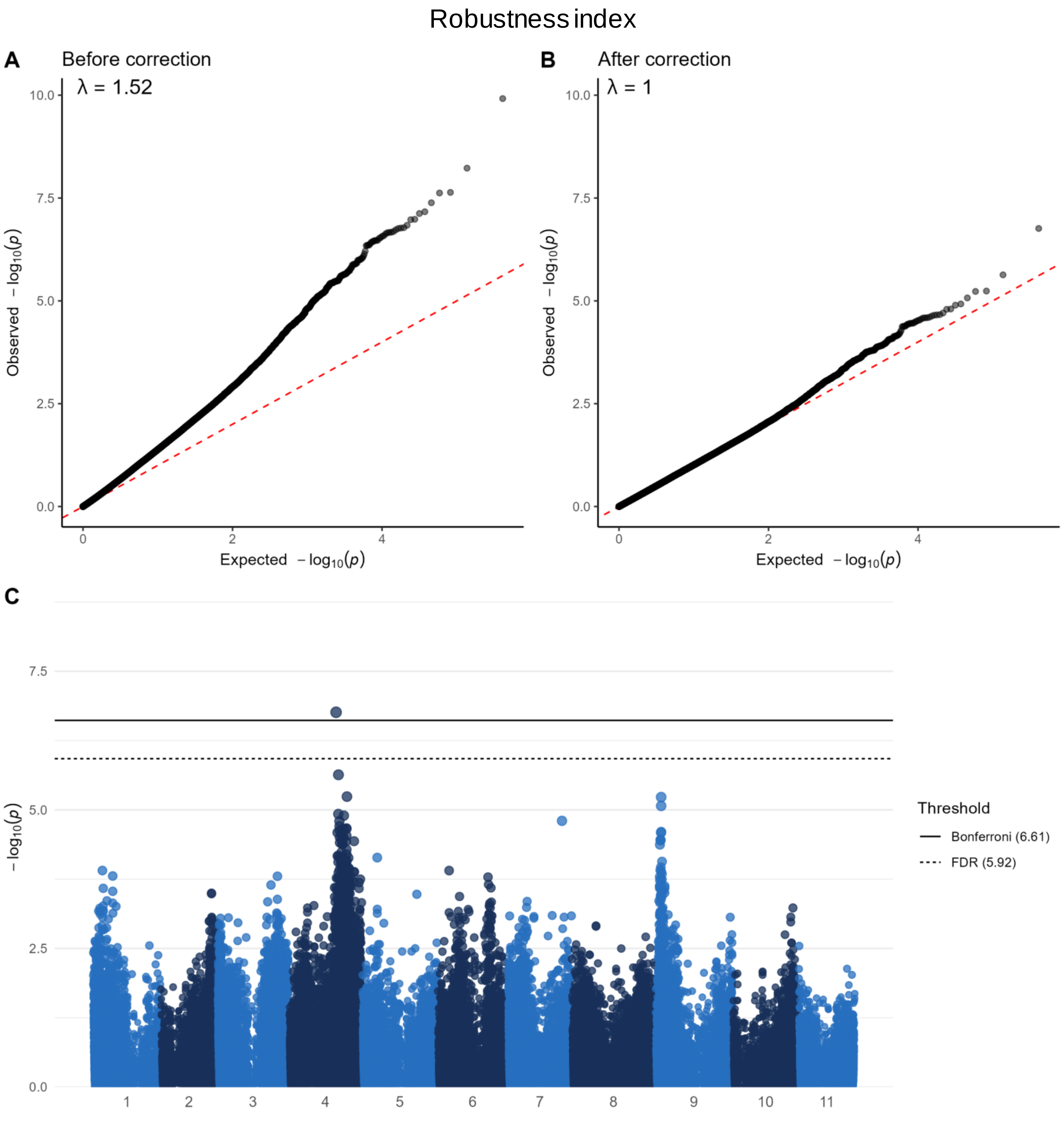

Figure S2X: QQ-plots of the p-values of the Kc model for robustness index before (**A**) and after (**B**) the correction by the inflation factor λ, and Manhattan plot (**C**) of the corrected p-values with the Bonferroni and FDR -$\log_{10}$(p-value) thresholds
