# Supplementary material for: Genome-wide association for agro-morphological traits in a triploid banana population with large chromosome rearrangements
Source: Hortic Res. 2024 Nov 6;12(2):uhae307. doi: 10.1093/hr/uhae307 (PMC11817881; doi:10.1093/hr/uhae307)
Supplement: Web_Material_uhae307 [file web_material_uhae307.zip › Figure_S3.pdf]

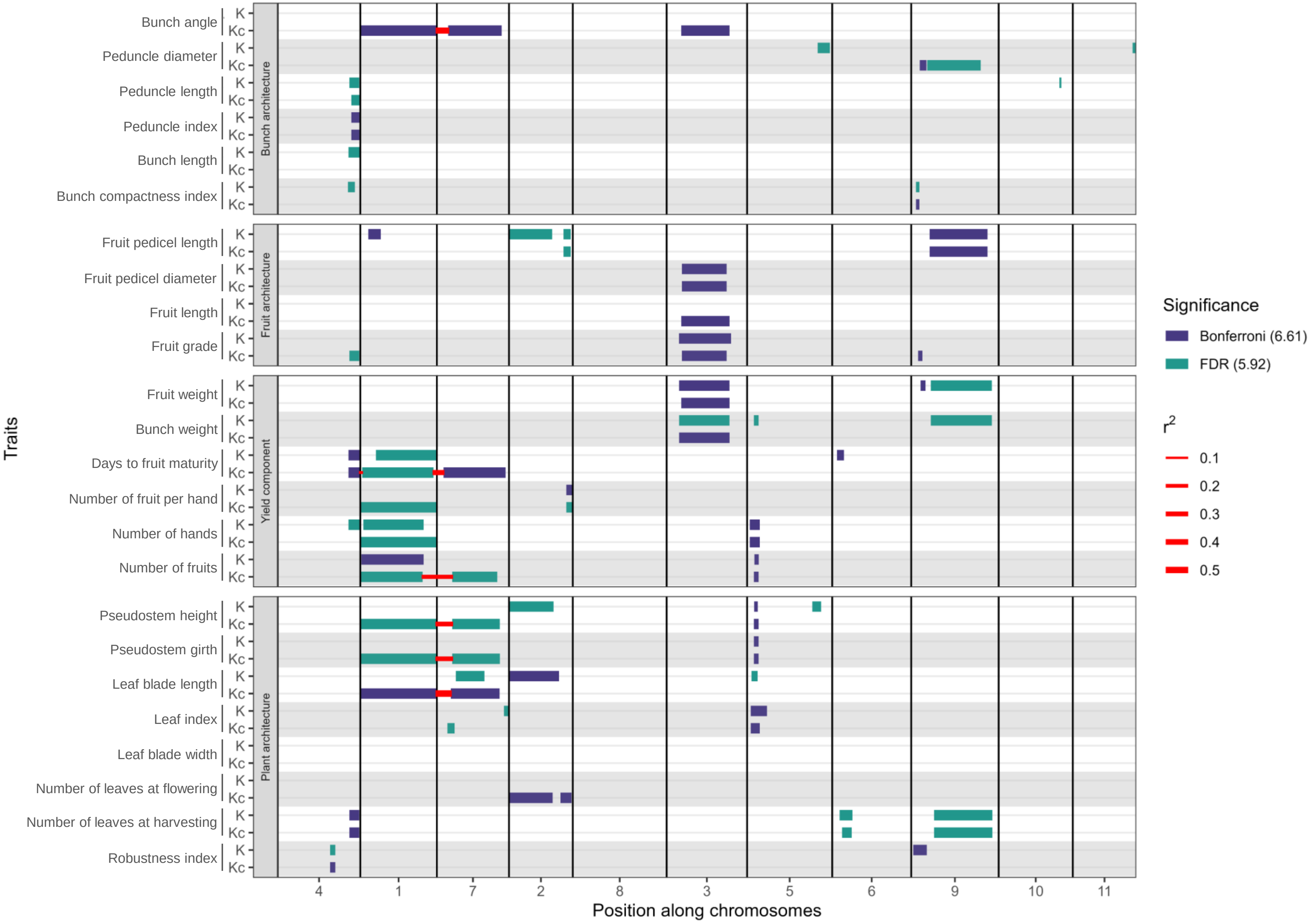

Figure S3: Localization of QTL LD intervals along chromosomes for each trait and GWAS model, according to two significance thresholds: Bonferroni (-log10(p) = 6.61) and FDR (-log10(p) = 5.92). Co-segregations between the most significant SNPs of each interval are indicated by a red segment with a width proportional to the level of LD (r^2) whose values are shown in Table S2. The continuous r^2 size scale is represented by discrete values from 0.1 to 0.5. The order of the chromosomes on the x-axis was chosen so as to position the chromosomes involved in a reciprocal translocation close to each other.
