# Supplementary material for: Genome-wide association for agro-morphological traits in a triploid banana population with large chromosome rearrangements
Source: Hortic Res. 2024 Nov 6;12(2):uhae307. doi: 10.1093/hr/uhae307 (PMC11817881; doi:10.1093/hr/uhae307)
Supplement: Web_Material_uhae307 [file web_material_uhae307.zip › Figure_S4.pdf]

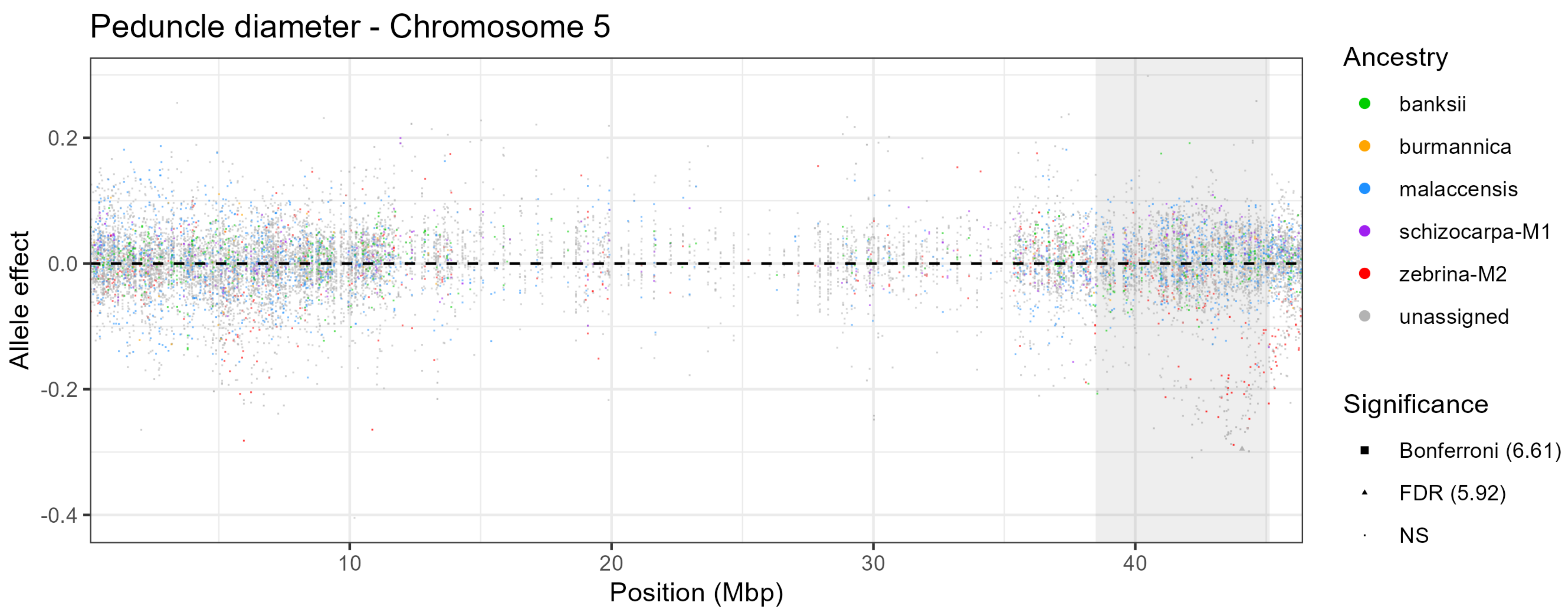


**Figure S4AA**: Estimated allele effects along chromosome 5 for peduncle diameter obtained using the K model. Dots are colored according to allele ancestry and shaped according to the level of significance of the test. When no ancestry could be assigned, the effect represented is that of the alternative allele. The QTL interval considered is indicated by a gray area.

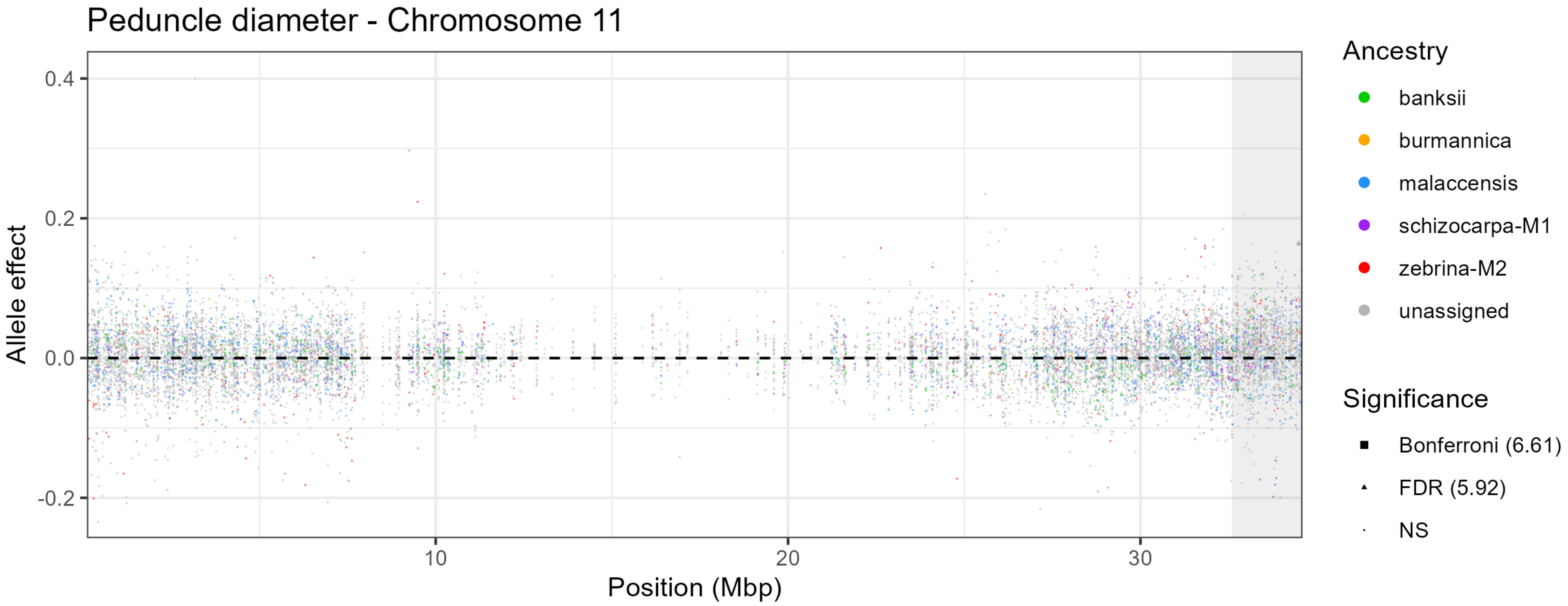


**Figure S4AB**: Estimated allele effects along chromosome 11 for peduncle diameter obtained using the K model. Dots are colored according to allele ancestry and shaped according to the level of significance of the test. When no ancestry could be assigned, the effect represented is that of the alternative allele. The QTL interval considered is indicated by a gray area.

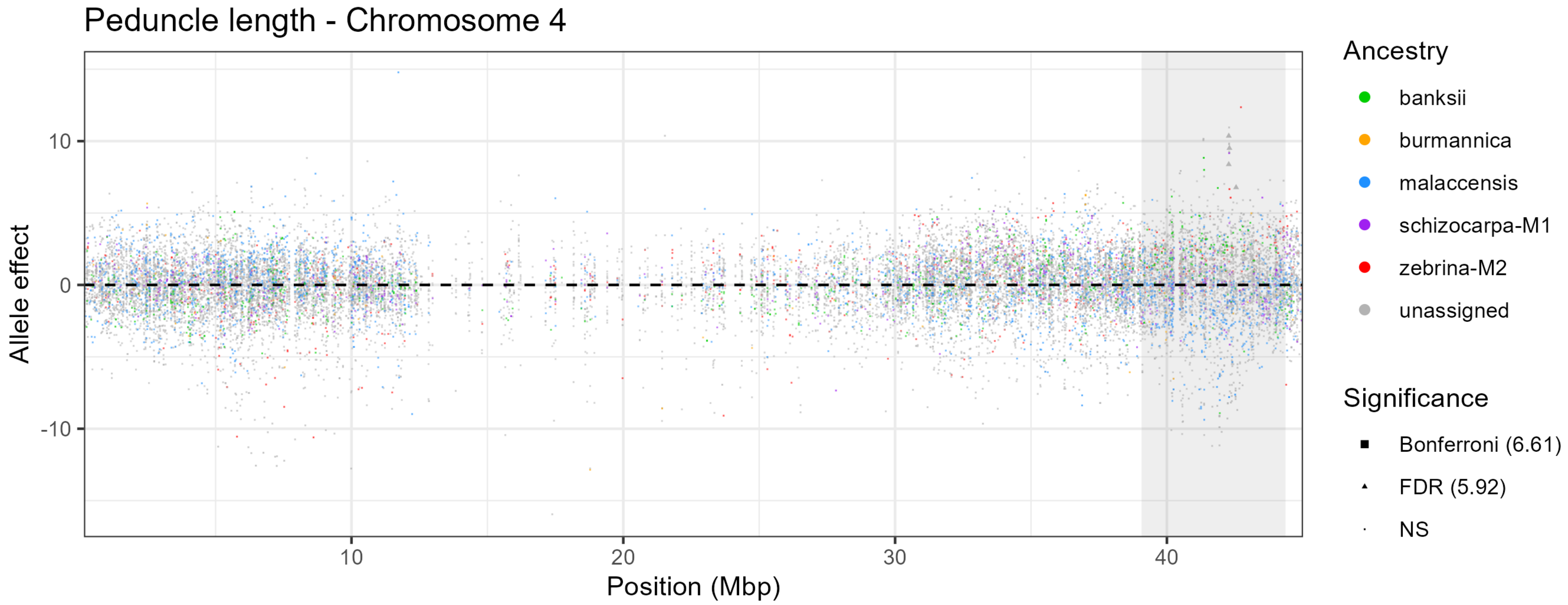


**Figure S4AC**: Estimated allele effects along chromosome 4 for peduncle length obtained using the K model. Dots are colored according to allele ancestry and shaped according to the level of significance of the test. When no ancestry could be assigned, the effect represented is that of the alternative allele. The QTL interval considered is indicated by a gray area.

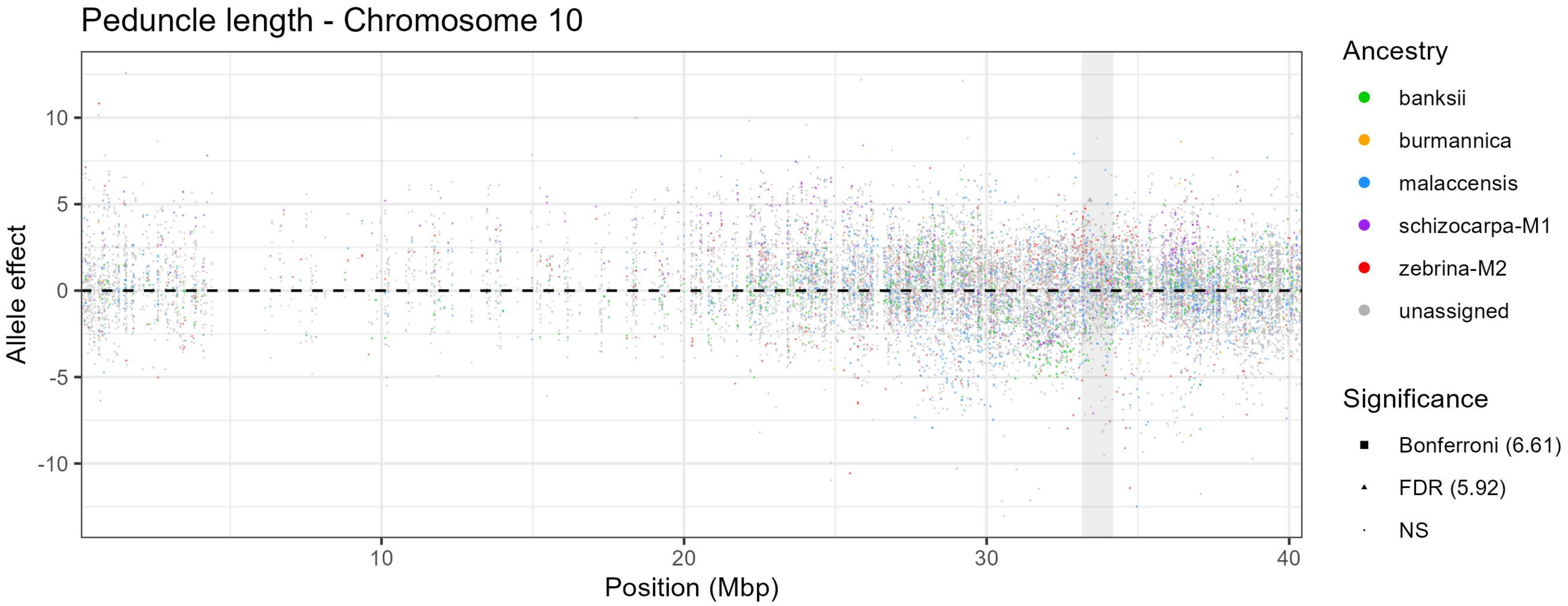


**Figure S4AD**: Estimated allele effects along chromosome 10 for peduncle length obtained using the K model. Dots are colored according to allele ancestry and shaped according to the level of significance of the test. When no ancestry could be assigned, the effect represented is that of the alternative allele. The QTL interval considered is indicated by a gray area.

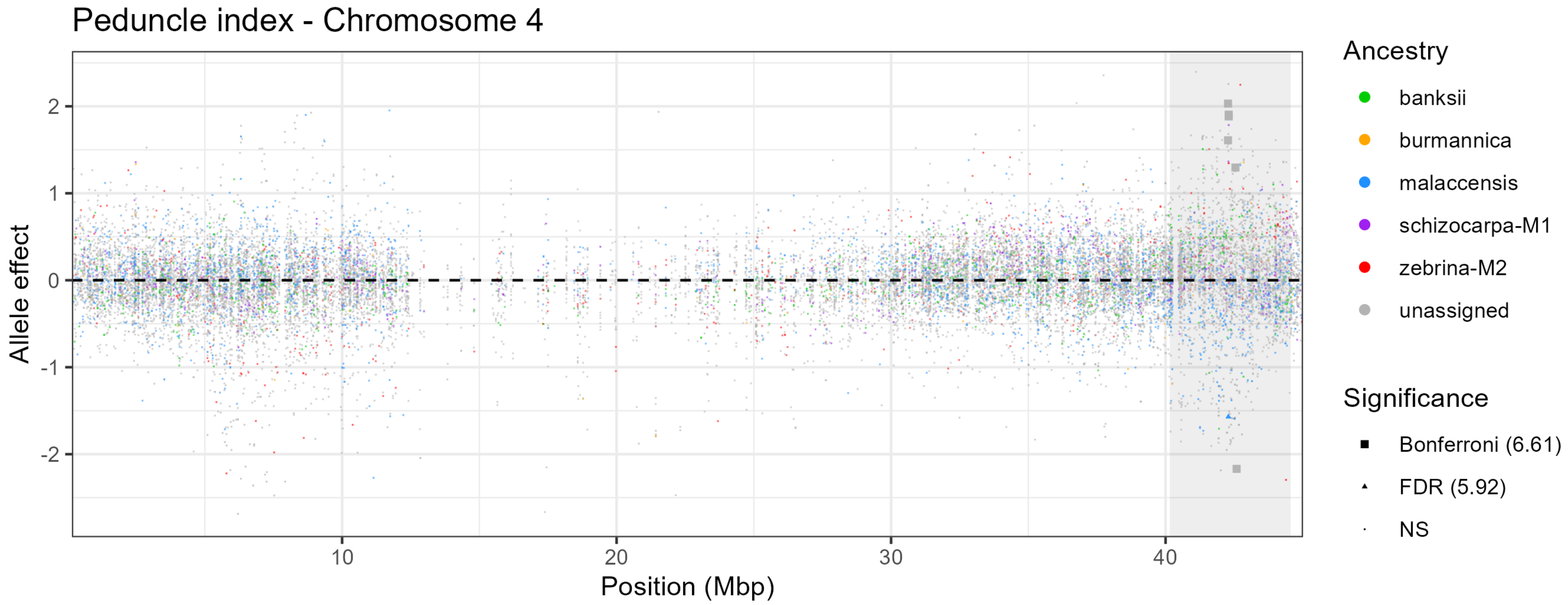


**Figure S4AE**: Estimated allele effects along chromosome 4 for peduncle index obtained using the K model. Dots are colored according to allele ancestry and shaped according to the level of significance of the test. When no ancestry could be assigned, the effect represented is that of the alternative allele. The QTL interval considered is indicated by a gray area.

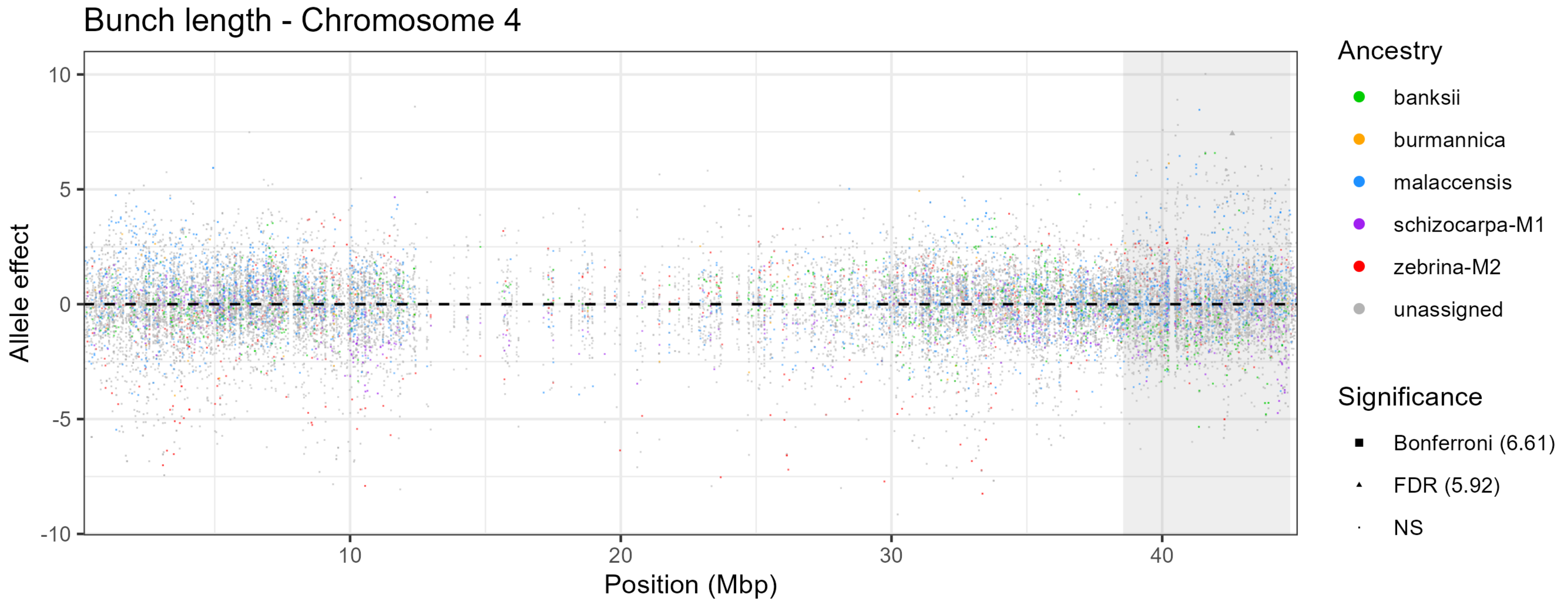


**Figure S4AF**: Estimated allele effects along chromosome 4 for bunch length obtained using the K model. Dots are colored according to allele ancestry and shaped according to the level of significance of the test. When no ancestry could be assigned, the effect represented is that of the alternative allele. The QTL interval considered is indicated by a gray area.

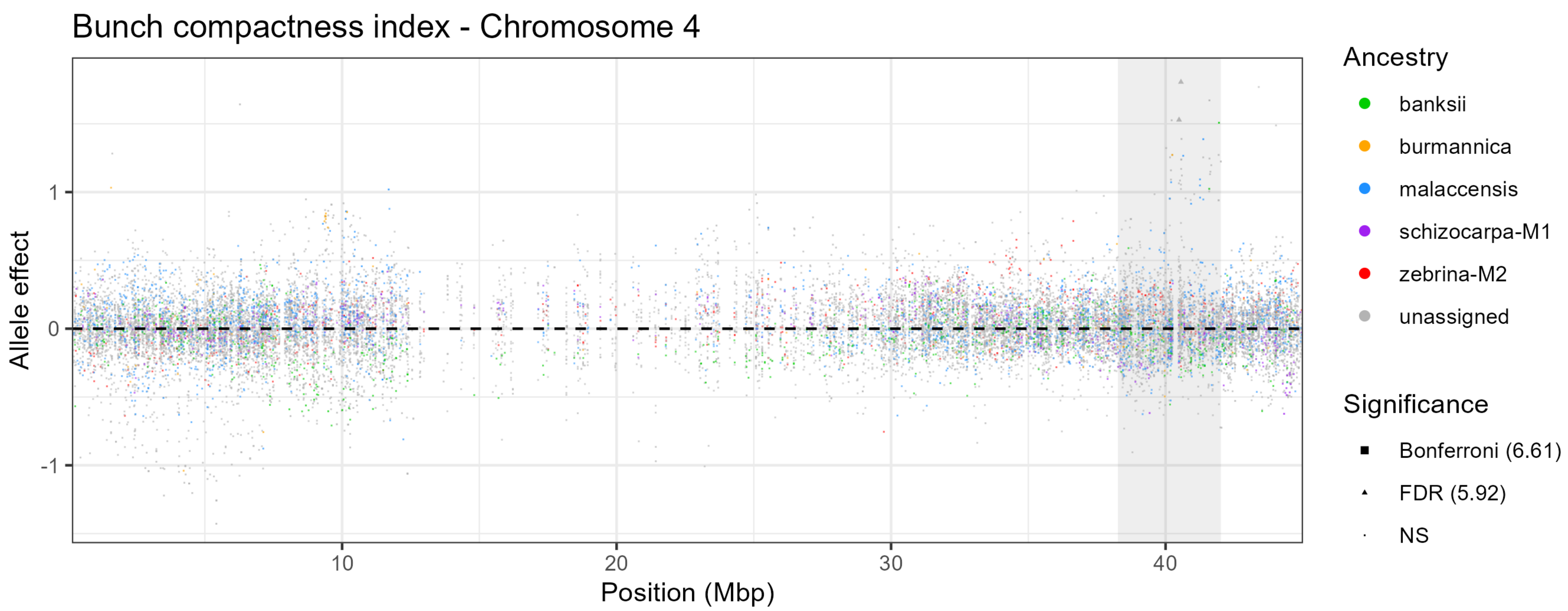


**Figure S4AG**: Estimated allele effects along chromosome 4 for bunch compactness index obtained using the K model. Dots are colored according to allele ancestry and shaped according to the level of significance of the test. When no ancestry could be assigned, the effect represented is that of the alternative allele. The QTL interval considered is indicated by a gray area.

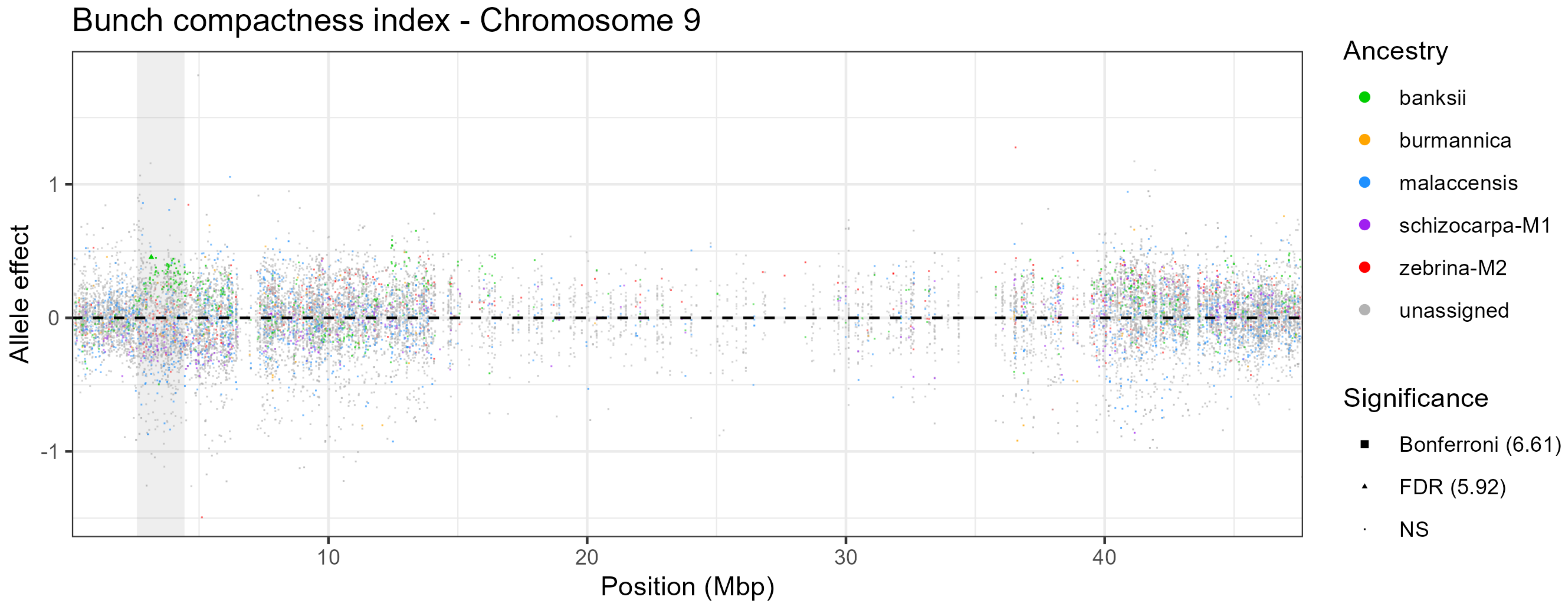


**Figure S4AH**: Estimated allele effects along chromosome 9 for bunch compactness index obtained using the K model. Dots are colored according to allele ancestry and shaped according to the level of significance of the test. When no ancestry could be assigned, the effect represented is that of the alternative allele. The QTL interval considered is indicated by a gray area.

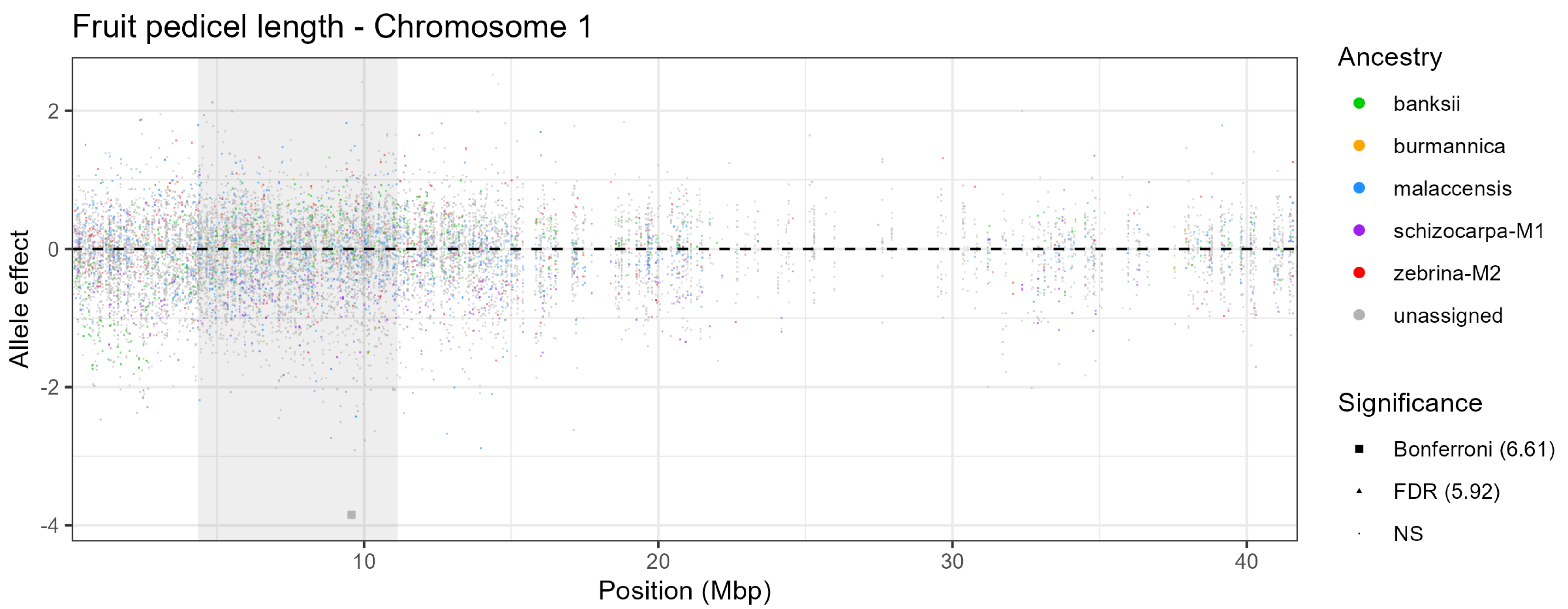


**Figure S4AI**: Estimated allele effects along chromosome 1 for fruit pedicel length obtained using the K model. Dots are colored according to allele ancestry and shaped according to the level of significance of the test. When no ancestry could be assigned, the effect represented is that of the alternative allele. The QTL interval considered is indicated by a gray area.

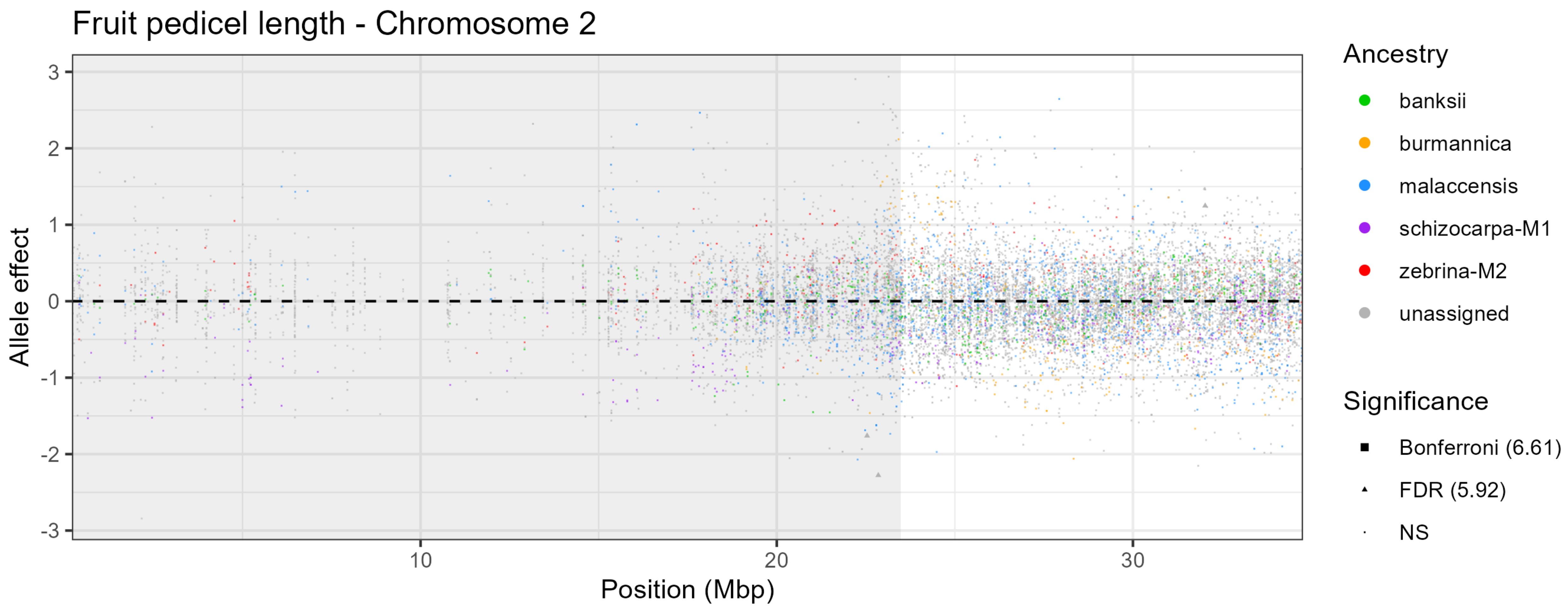


**Figure S4AJ**: Estimated allele effects along chromosome 2 for fruit pedicel length obtained using the K model. Dots are colored according to allele ancestry and shaped according to the level of significance of the test. When no ancestry could be assigned, the effect represented is that of the alternative allele. The QTL interval considered is indicated by a gray area.

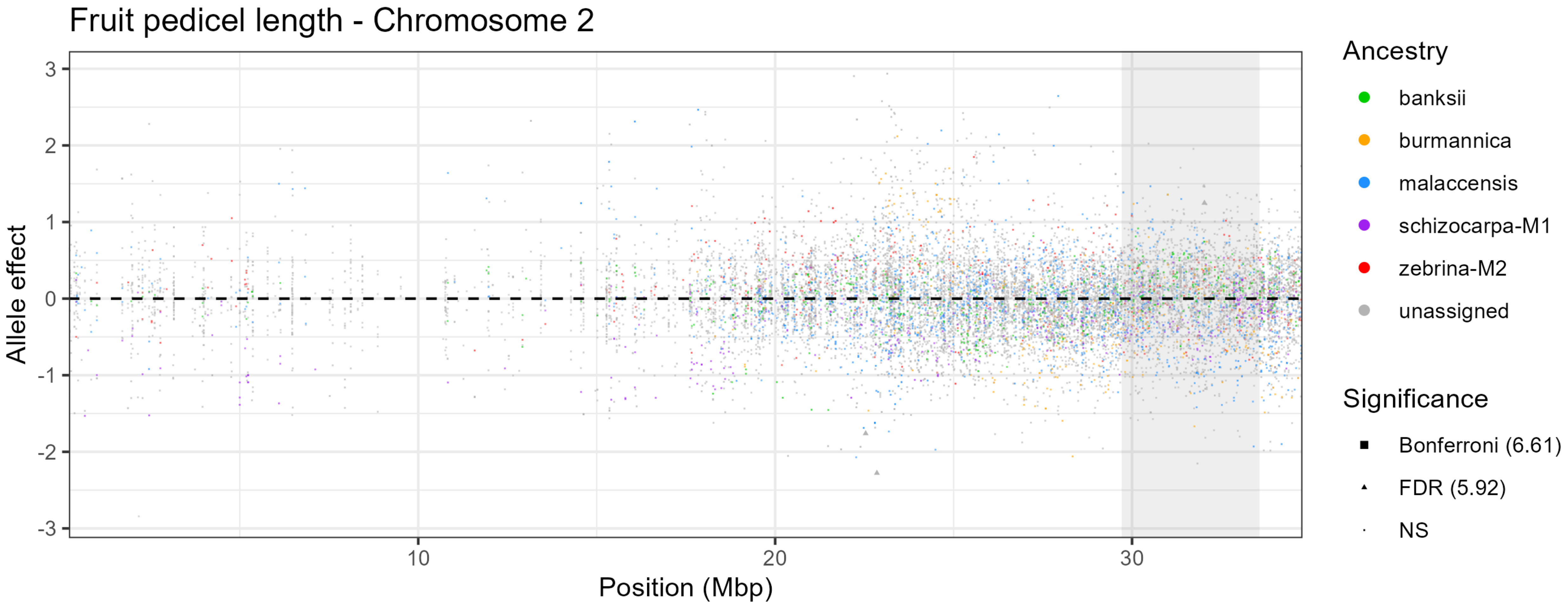


**Figure S4AK**: Estimated allele effects along chromosome 2 for fruit pedicel length obtained using the K model. Dots are colored according to allele ancestry and shaped according to the level of significance of the test. When no ancestry could be assigned, the effect represented is that of the alternative allele. The QTL interval considered is indicated by a gray area.

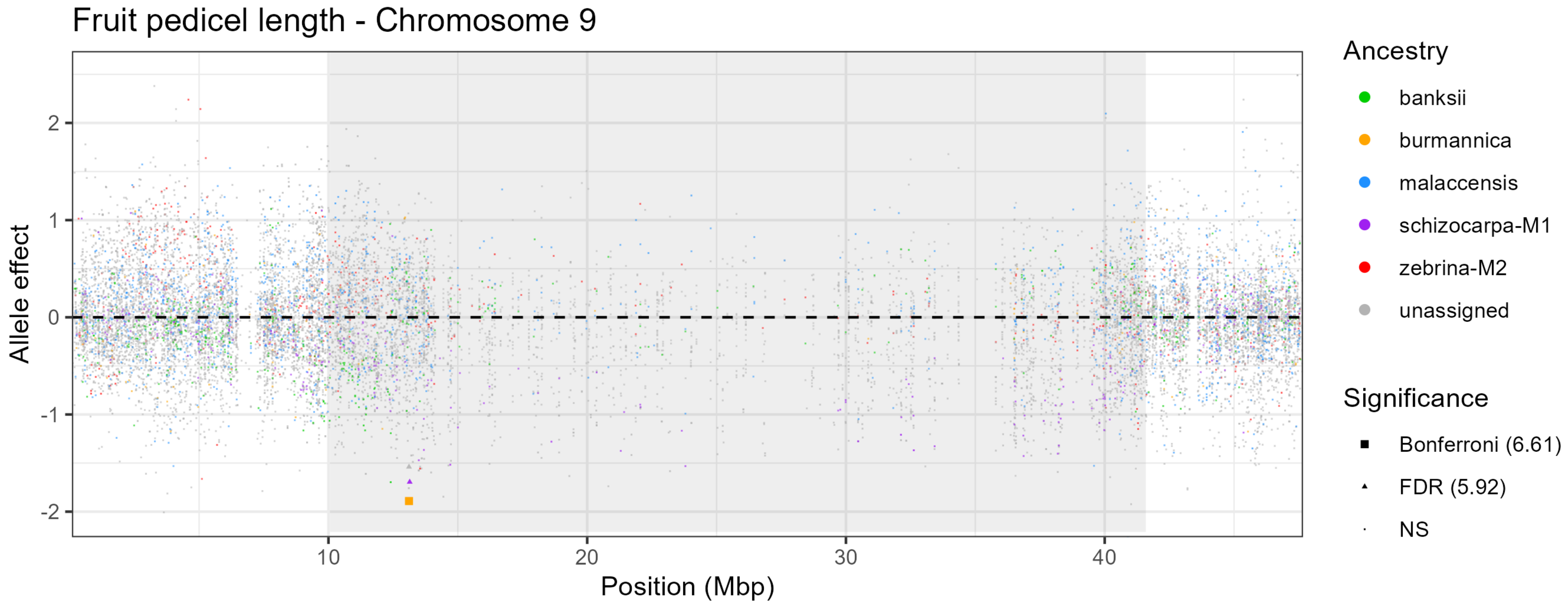


**Figure S4AL**: Estimated allele effects along chromosome 9 for fruit pedicel length obtained using the K model. Dots are colored according to allele ancestry and shaped according to the level of significance of the test. When no ancestry could be assigned, the effect represented is that of the alternative allele. The QTL interval considered is indicated by a gray area.

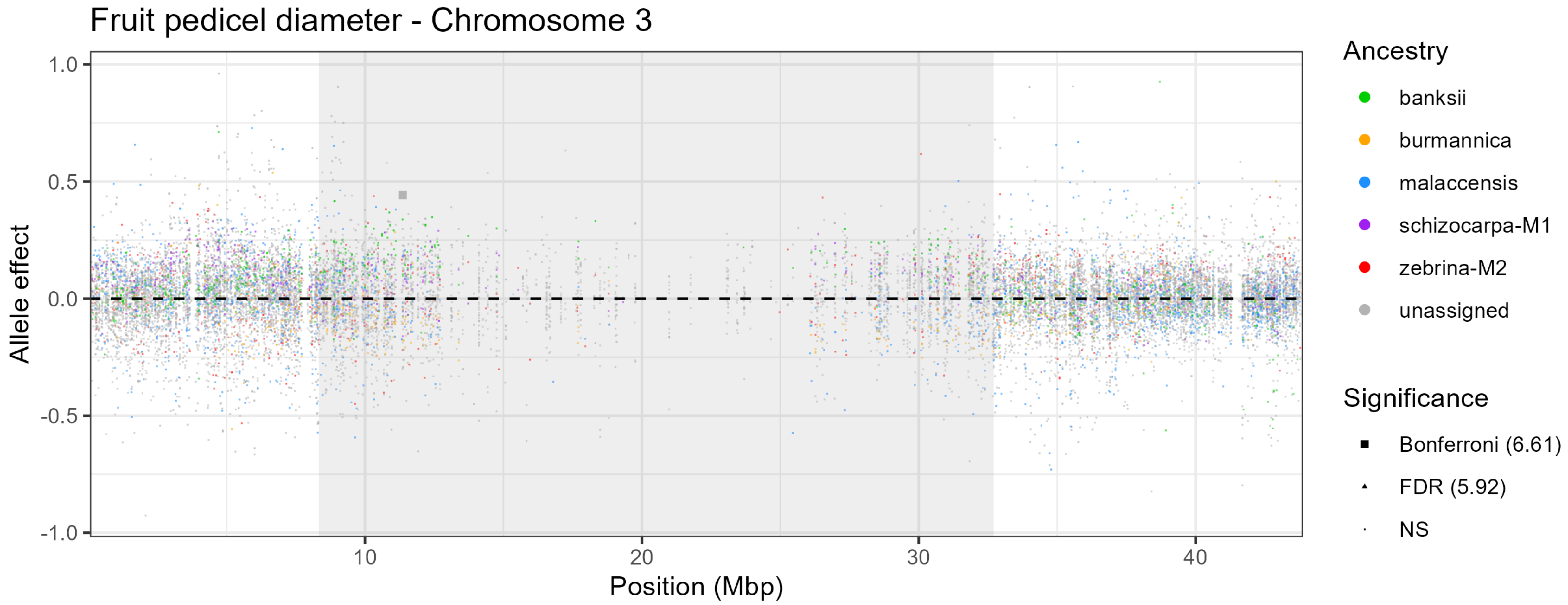


**Figure S4AM**: Estimated allele effects along chromosome 3 for fruit pedicel diameter obtained using the K model. Dots are colored according to allele ancestry and shaped according to the level of significance of the test. When no ancestry could be assigned, the effect represented is that of the alternative allele. The QTL interval considered is indicated by a gray area.

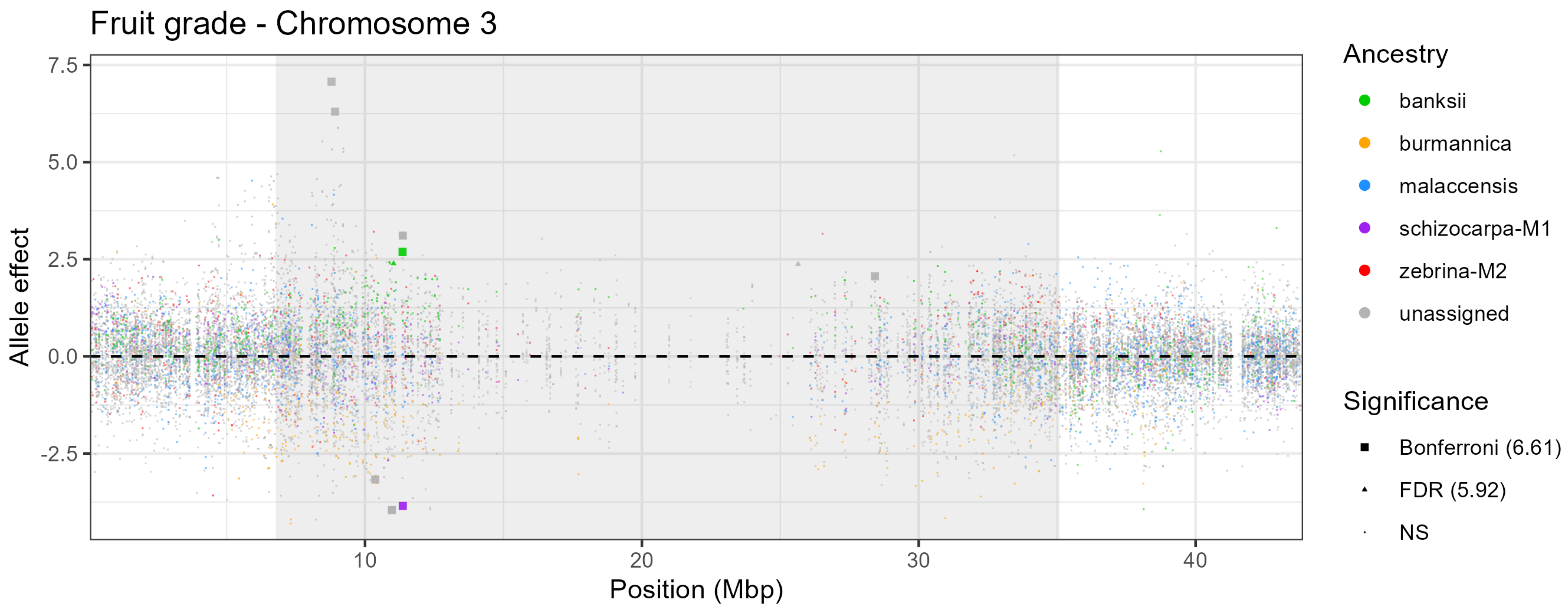


**Figure S4AN**: Estimated allele effects along chromosome 3 for fruit grade obtained using the K model. Dots are colored according to allele ancestry and shaped according to the level of significance of the test. When no ancestry could be assigned, the effect represented is that of the alternative allele. The QTL interval considered is indicated by a gray area.

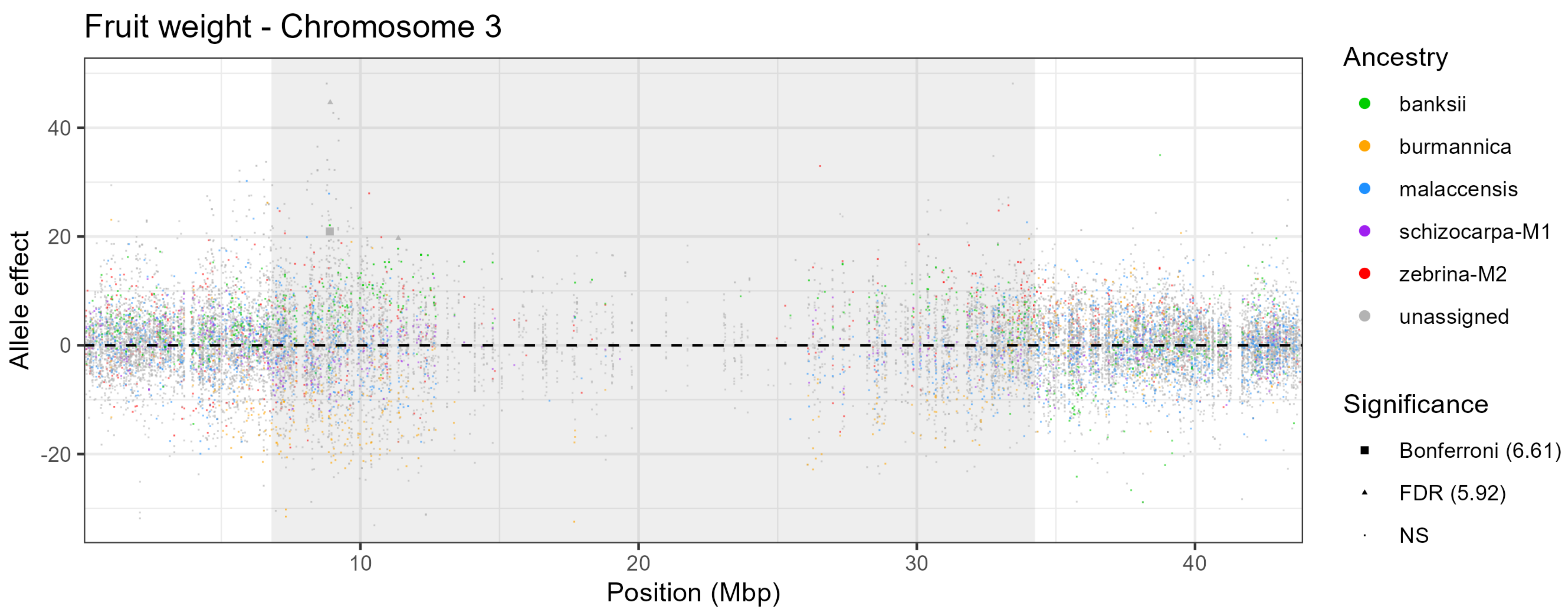


**Figure S4AO**: Estimated allele effects along chromosome 3 for fruit weight obtained using the K model. Dots are colored according to allele ancestry and shaped according to the level of significance of the test. When no ancestry could be assigned, the effect represented is that of the alternative allele. The QTL interval considered is indicated by a gray area.

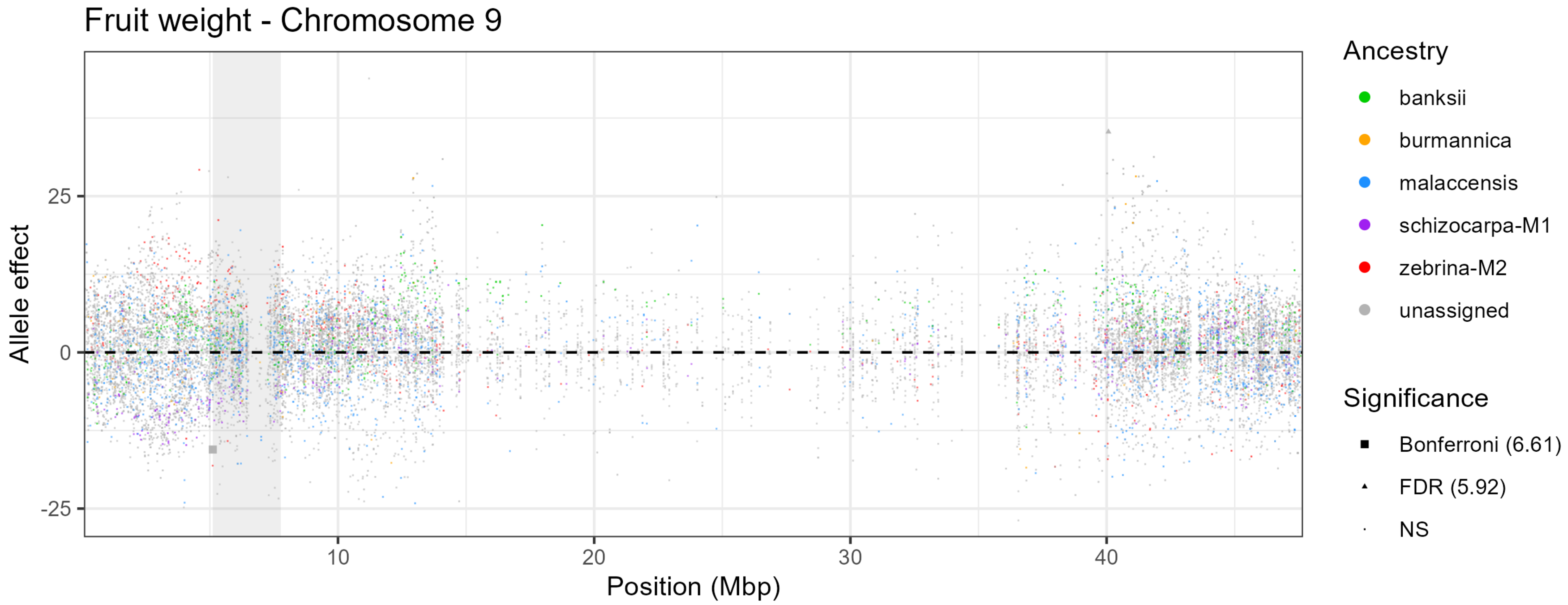


**Figure S4AP**: Estimated allele effects along chromosome 9 for fruit weight obtained using the K model. Dots are colored according to allele ancestry and shaped according to the level of significance of the test. When no ancestry could be assigned, the effect represented is that of the alternative allele. The QTL interval considered is indicated by a gray area.

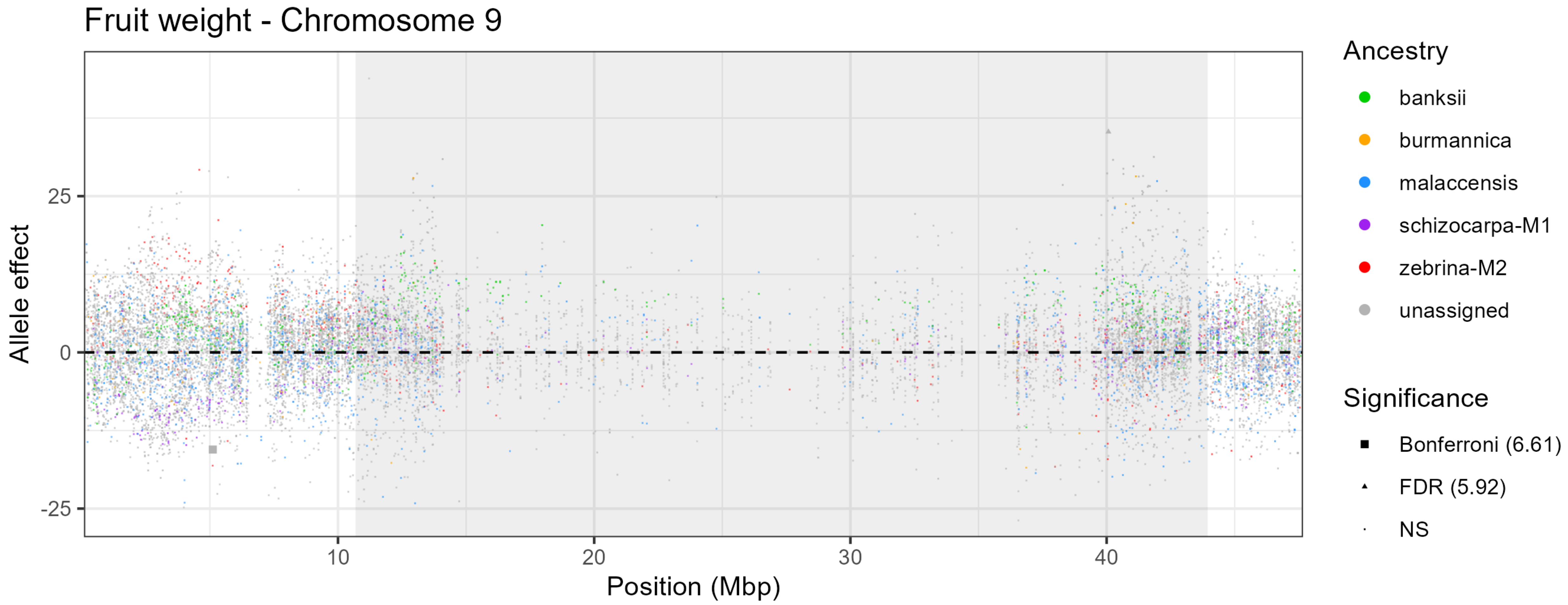


**Figure S4AQ**: Estimated allele effects along chromosome 9 for fruit weight obtained using the K model. Dots are colored according to allele ancestry and shaped according to the level of significance of the test. When no ancestry could be assigned, the effect represented is that of the alternative allele. The QTL interval considered is indicated by a gray area.

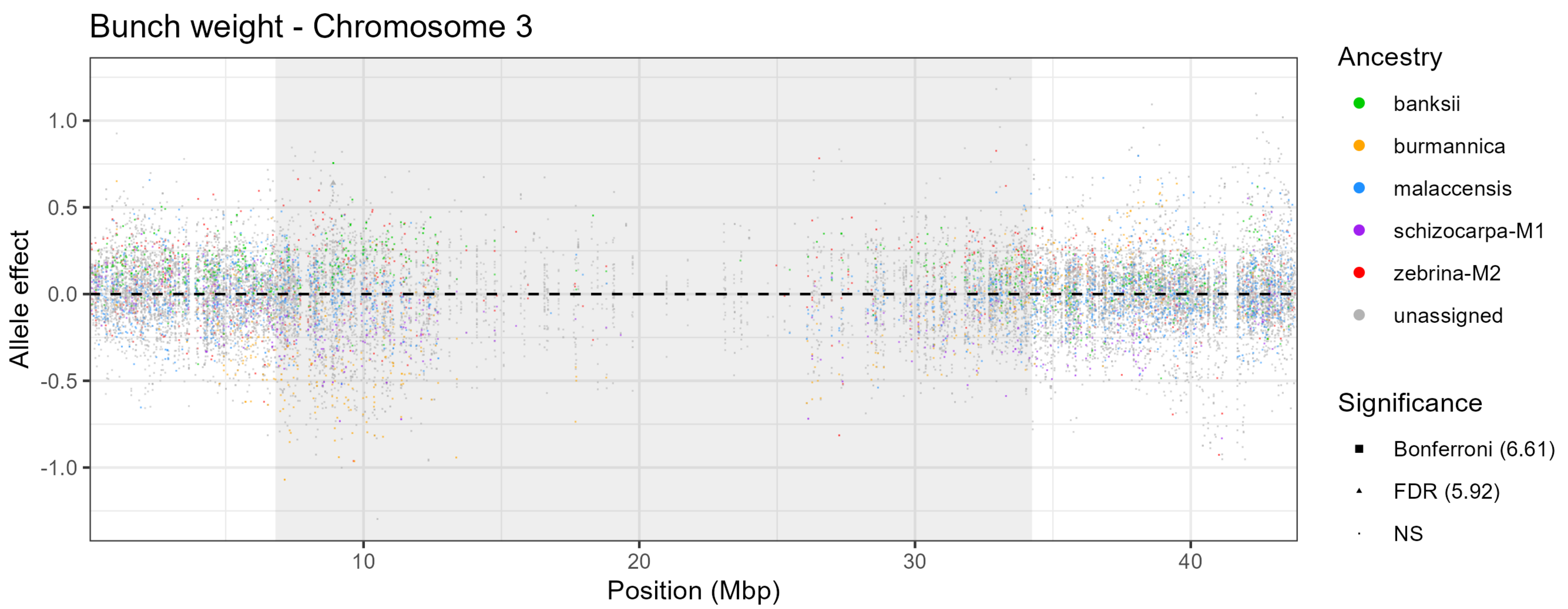


**Figure S4AR**: Estimated allele effects along chromosome 3 for bunch weight obtained using the K model. Dots are colored according to allele ancestry and shaped according to the level of significance of the test. When no ancestry could be assigned, the effect represented is that of the alternative allele. The QTL interval considered is indicated by a gray area.

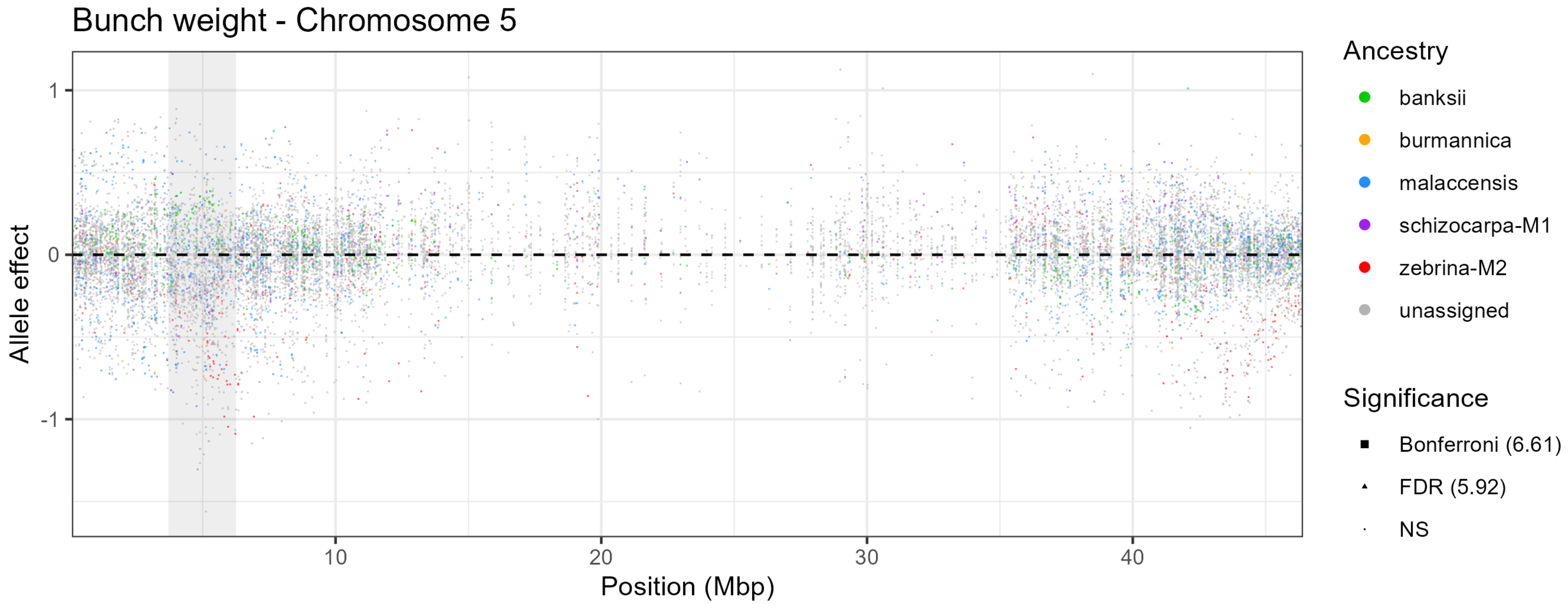


**Figure S4AS**: Estimated allele effects along chromosome 5 for bunch weight obtained using the K model. Dots are colored according to allele ancestry and shaped according to the level of significance of the test. When no ancestry could be assigned, the effect represented is that of the alternative allele. The QTL interval considered is indicated by a gray area.

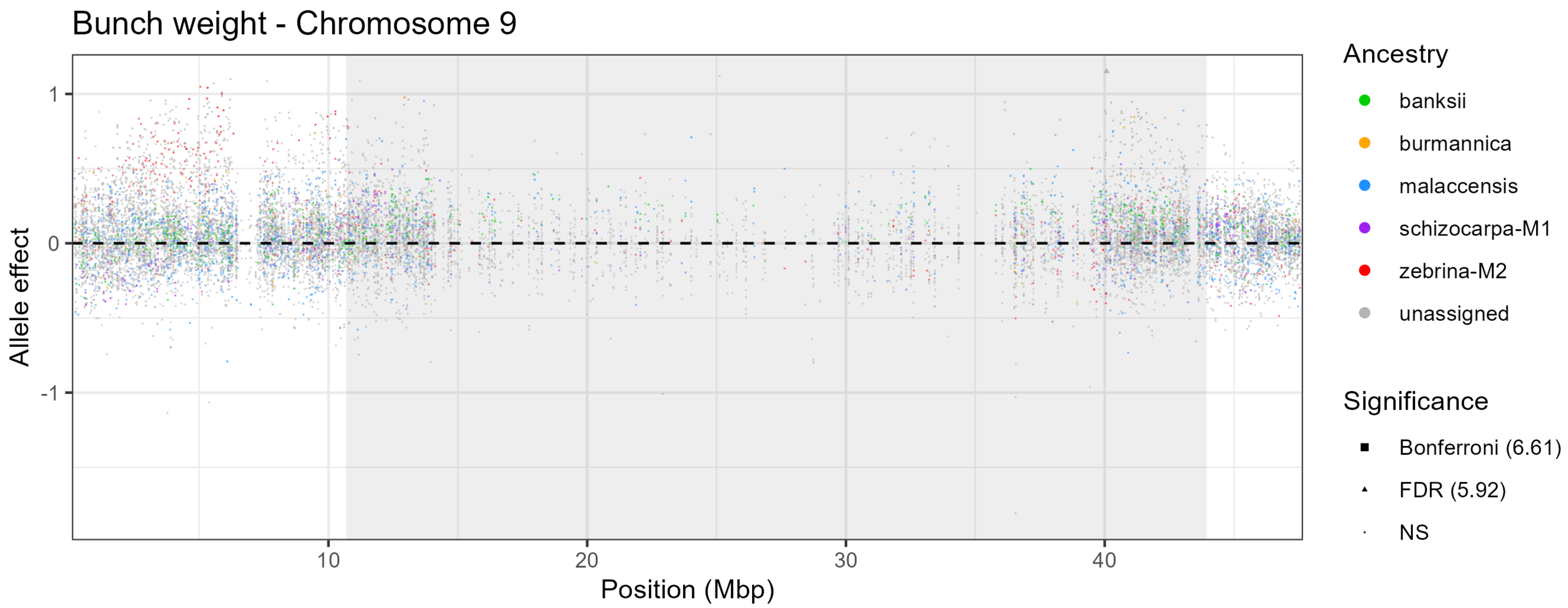


**Figure S4AT**: Estimated allele effects along chromosome 9 for bunch weight obtained using the K model. Dots are colored according to allele ancestry and shaped according to the level of significance of the test. When no ancestry could be assigned, the effect represented is that of the alternative allele. The QTL interval considered is indicated by a gray area.

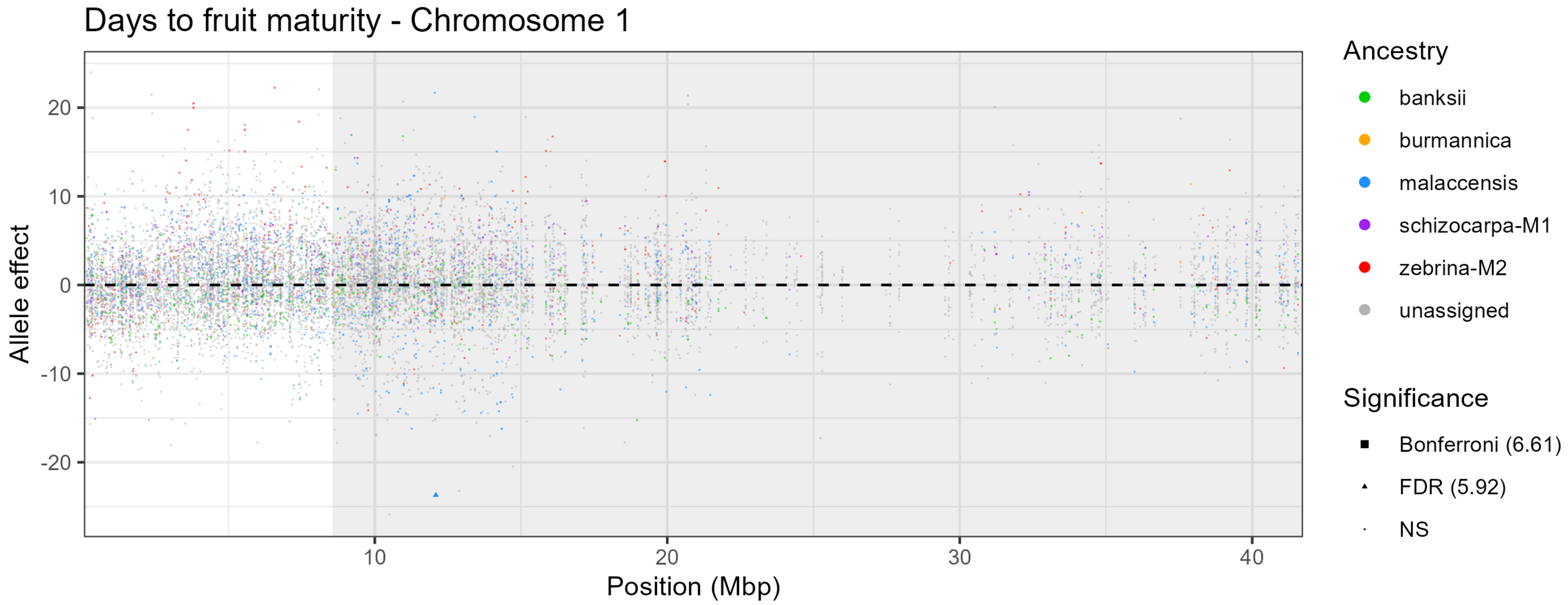


**Figure S4AU**: Estimated allele effects along chromosome 1 for days to fruit maturity obtained using the K model. Dots are colored according to allele ancestry and shaped according to the level of significance of the test. When no ancestry could be assigned, the effect represented is that of the alternative allele. The QTL interval considered is indicated by a gray area.

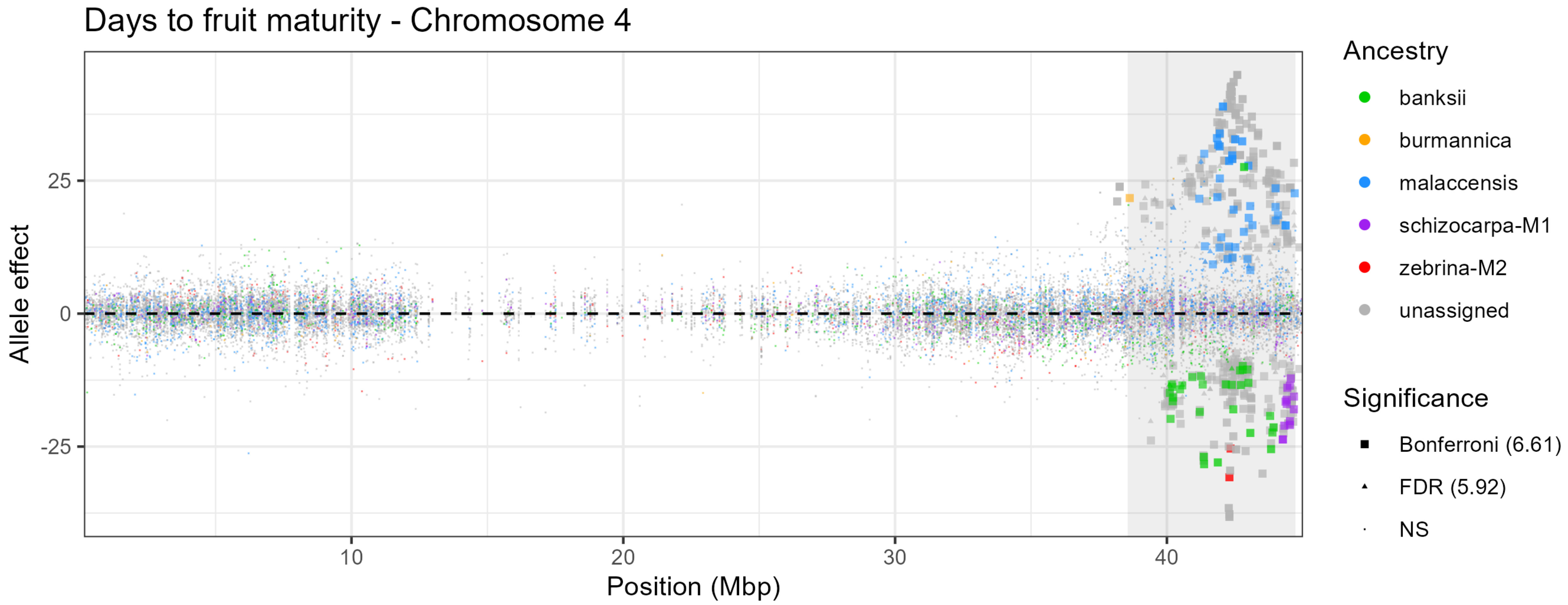


**Figure S4AV**: Estimated allele effects along chromosome 4 for days to fruit maturity obtained using the K model. Dots are colored according to allele ancestry and shaped according to the level of significance of the test. When no ancestry could be assigned, the effect represented is that of the alternative allele. The QTL interval considered is indicated by a gray area.

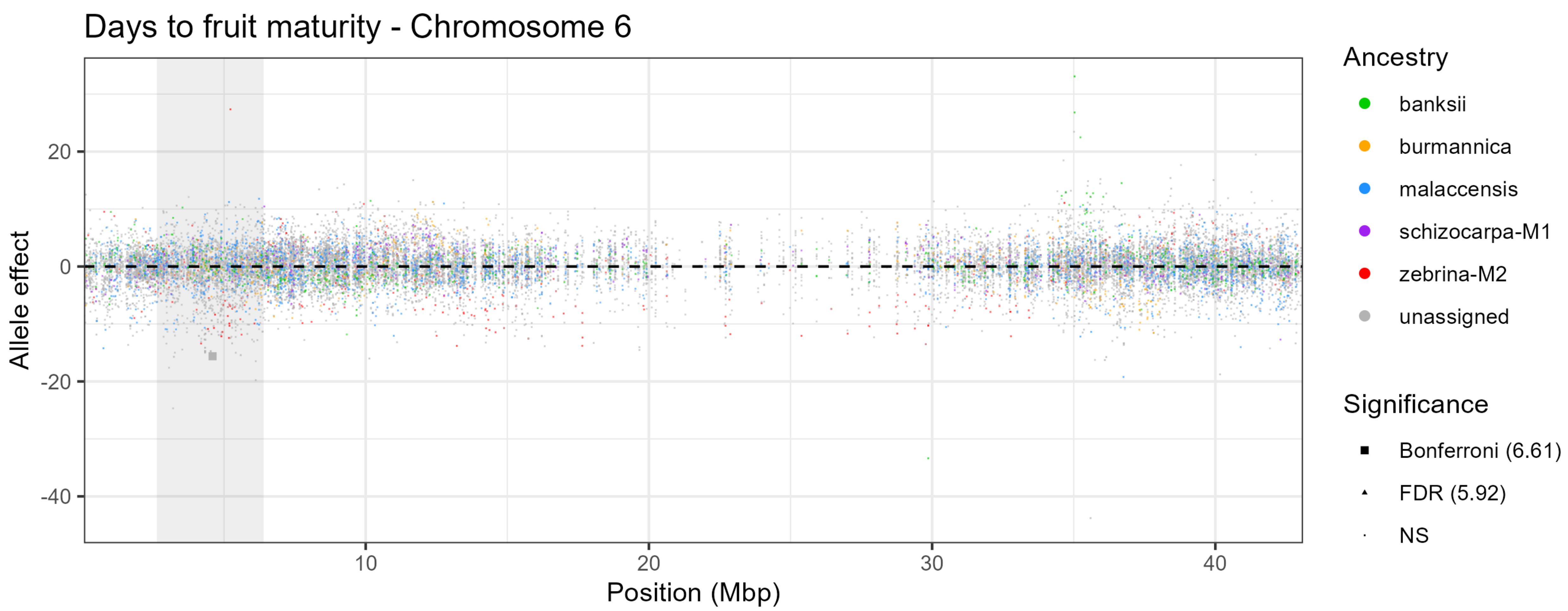


**Figure S4AW**: Estimated allele effects along chromosome 6 for days to fruit maturity obtained using the K model. Dots are colored according to allele ancestry and shaped according to the level of significance of the test. When no ancestry could be assigned, the effect represented is that of the alternative allele. The QTL interval considered is indicated by a gray area.

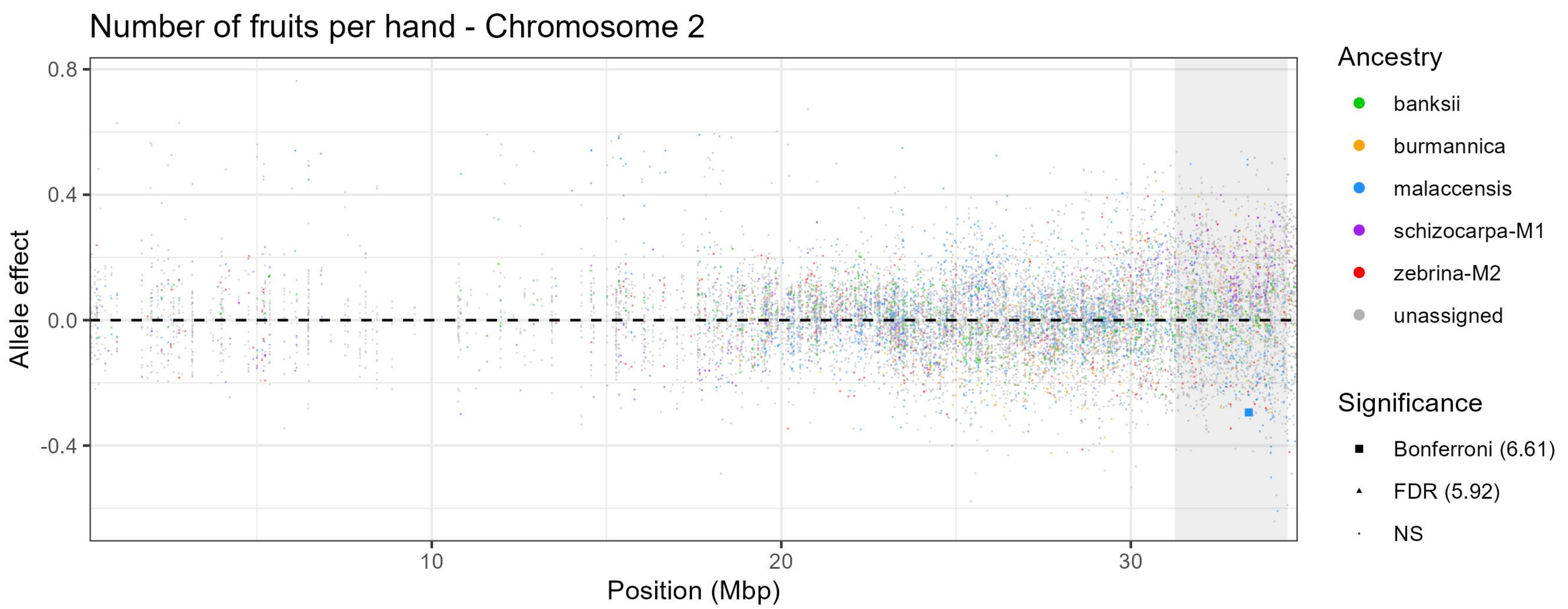


**Figure S4AX**: Estimated allele effects along chromosome 2 for number of fruits per hand obtained using the K model. Dots are colored according to allele ancestry and shaped according to the level of significance of the test. When no ancestry could be assigned, the effect represented is that of the alternative allele. The QTL interval considered is indicated by a gray area.

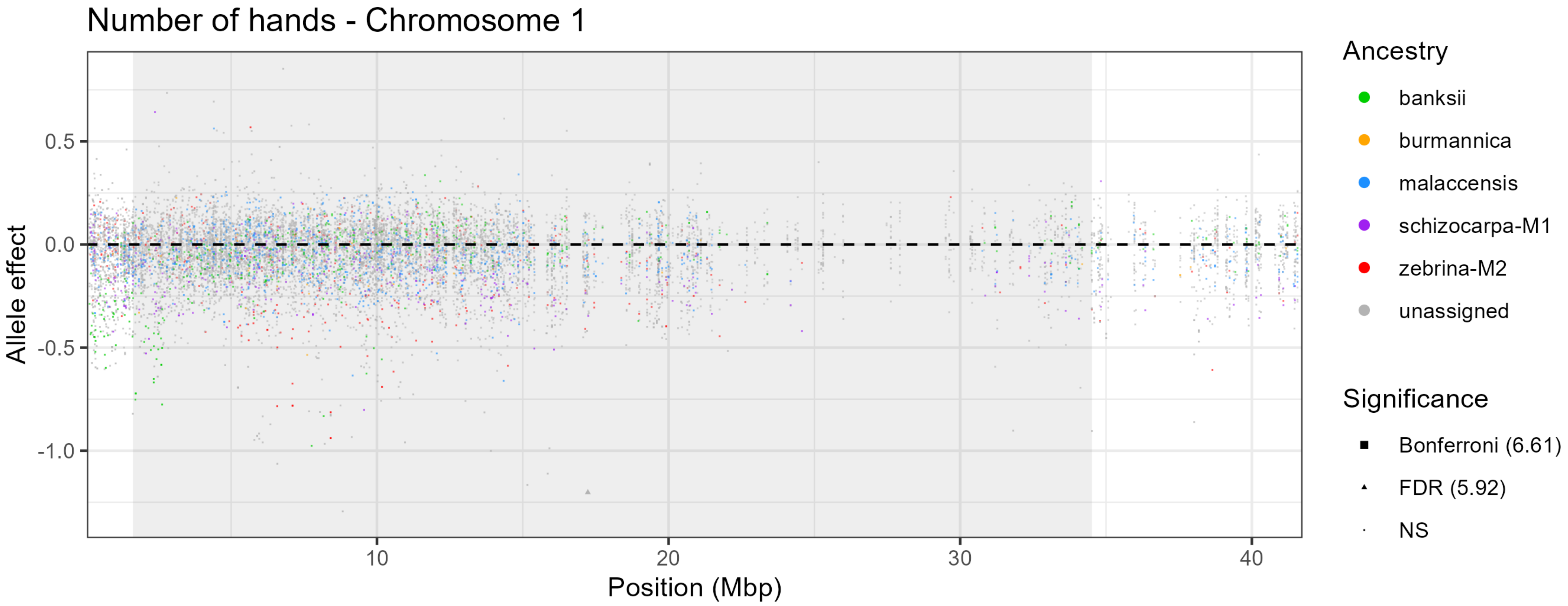


**Figure S4AY**: Estimated allele effects along chromosome 1 for number of hands obtained using the K model. Dots are colored according to allele ancestry and shaped according to the level of significance of the test. When no ancestry could be assigned, the effect represented is that of the alternative allele. The QTL interval considered is indicated by a gray area.

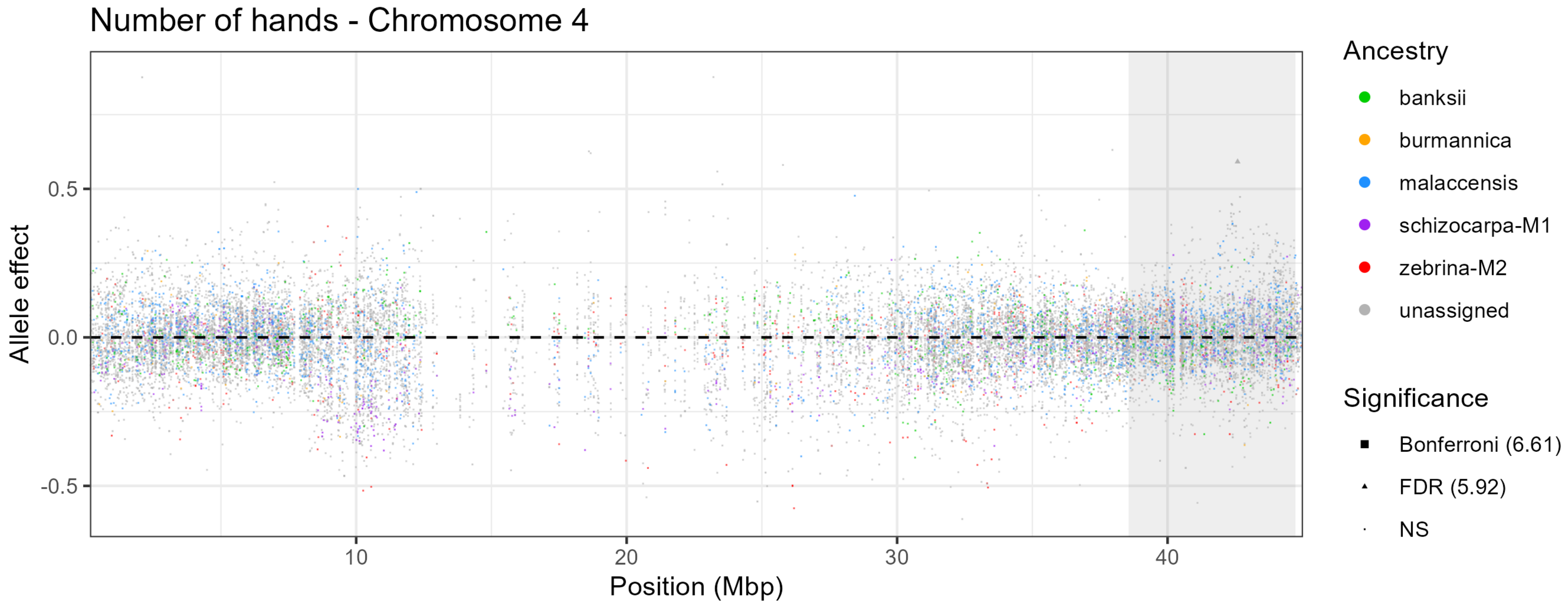


**Figure S4AZ**: Estimated allele effects along chromosome 4 for number of hands obtained using the K model. Dots are colored according to allele ancestry and shaped according to the level of significance of the test. When no ancestry could be assigned, the effect represented is that of the alternative allele. The QTL interval considered is indicated by a gray area.

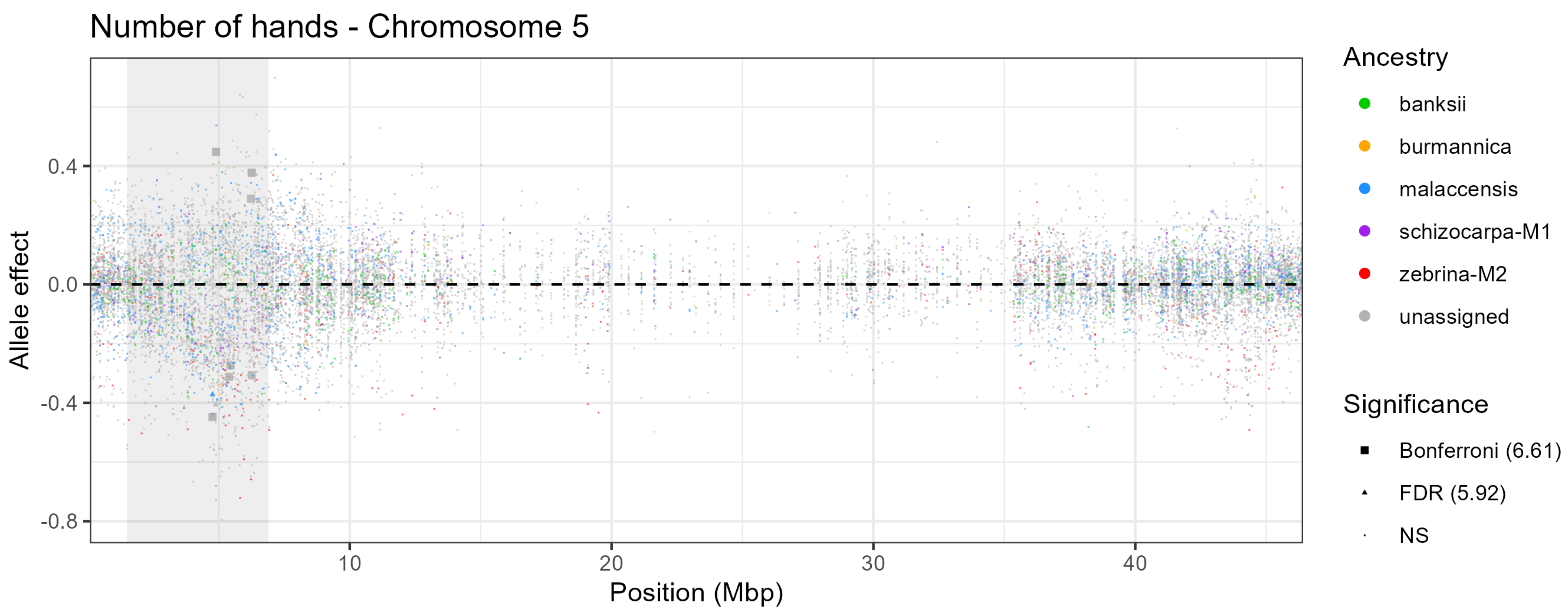


**Figure S4BA**: Estimated allele effects along chromosome 5 for number of hands obtained using the K model. Dots are colored according to allele ancestry and shaped according to the level of significance of the test. When no ancestry could be assigned, the effect represented is that of the alternative allele. The QTL interval considered is indicated by a gray area.

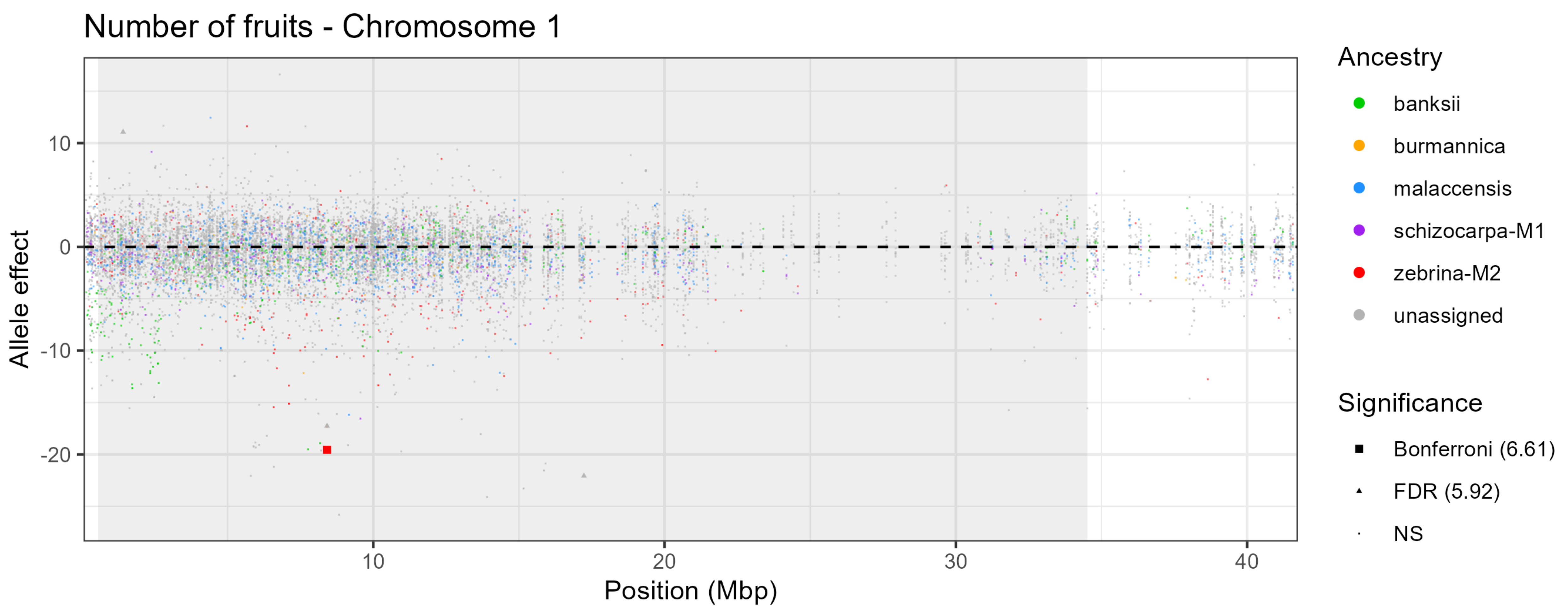


**Figure S4BB**: Estimated allele effects along chromosome 1 for number of fruits obtained using the K model. Dots are colored according to allele ancestry and shaped according to the level of significance of the test. When no ancestry could be assigned, the effect represented is that of the alternative allele. The QTL interval considered is indicated by a gray area.

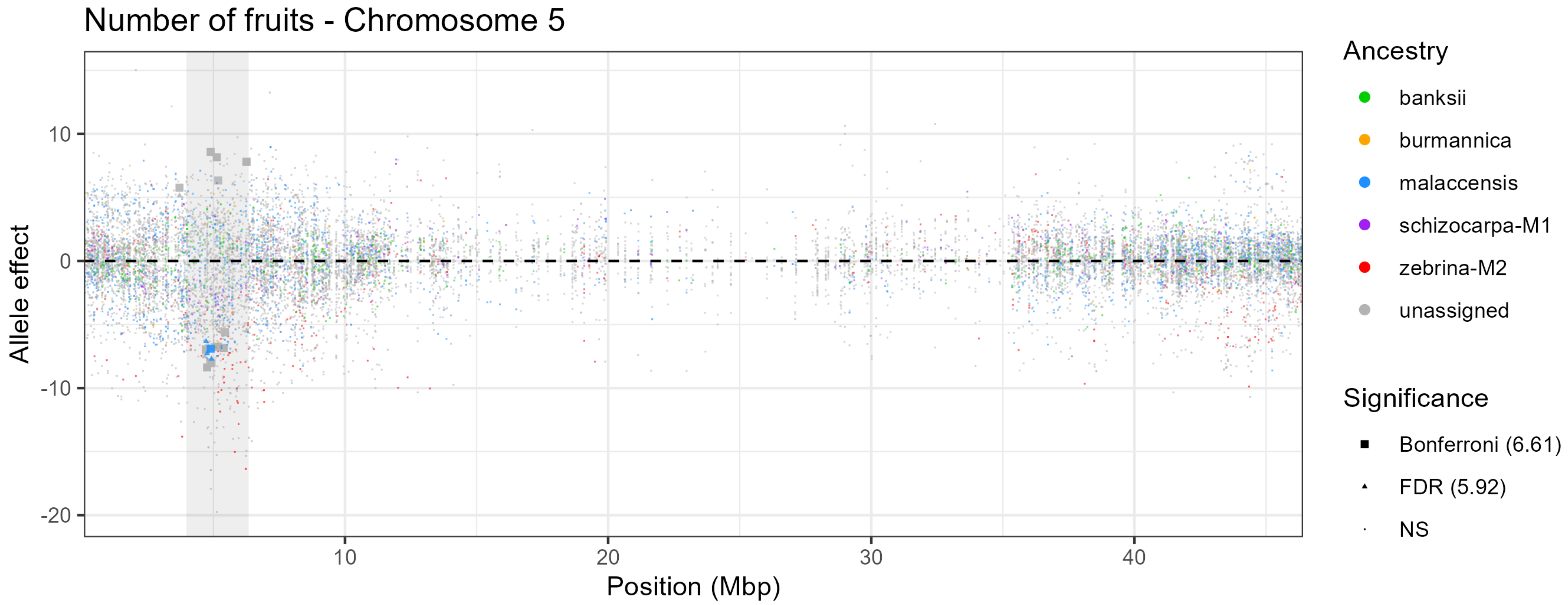


**Figure S4BC**: Estimated allele effects along chromosome 5 for number of fruits obtained using the K model. Dots are colored according to allele ancestry and shaped according to the level of significance of the test. When no ancestry could be assigned, the effect represented is that of the alternative allele. The QTL interval considered is indicated by a gray area.

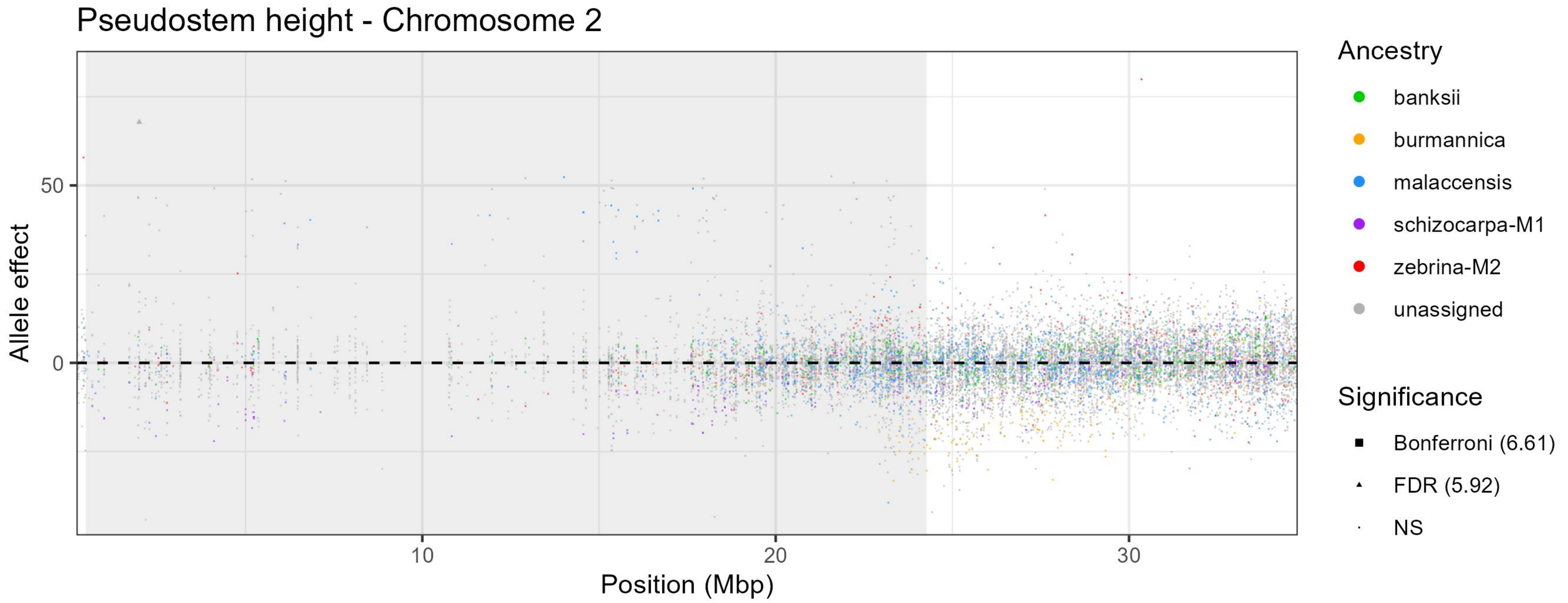


**Figure S4BD**: Estimated allele effects along chromosome 2 for pseudostem height obtained using the K model. Dots are colored according to allele ancestry and shaped according to the level of significance of the test. When no ancestry could be assigned, the effect represented is that of the alternative allele. The QTL interval considered is indicated by a gray area.

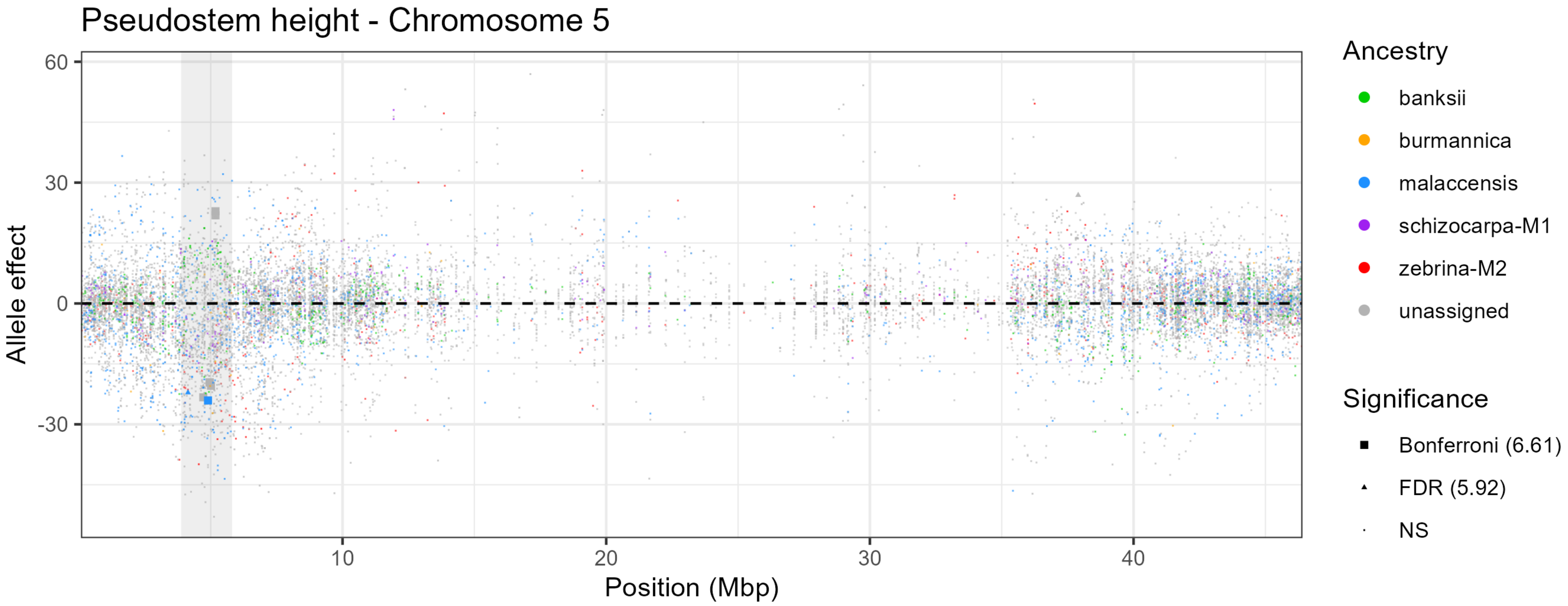


**Figure S4BE**: Estimated allele effects along chromosome 5 for pseudostem height obtained using the K model. Dots are colored according to allele ancestry and shaped according to the level of significance of the test. When no ancestry could be assigned, the effect represented is that of the alternative allele. The QTL interval considered is indicated by a gray area.

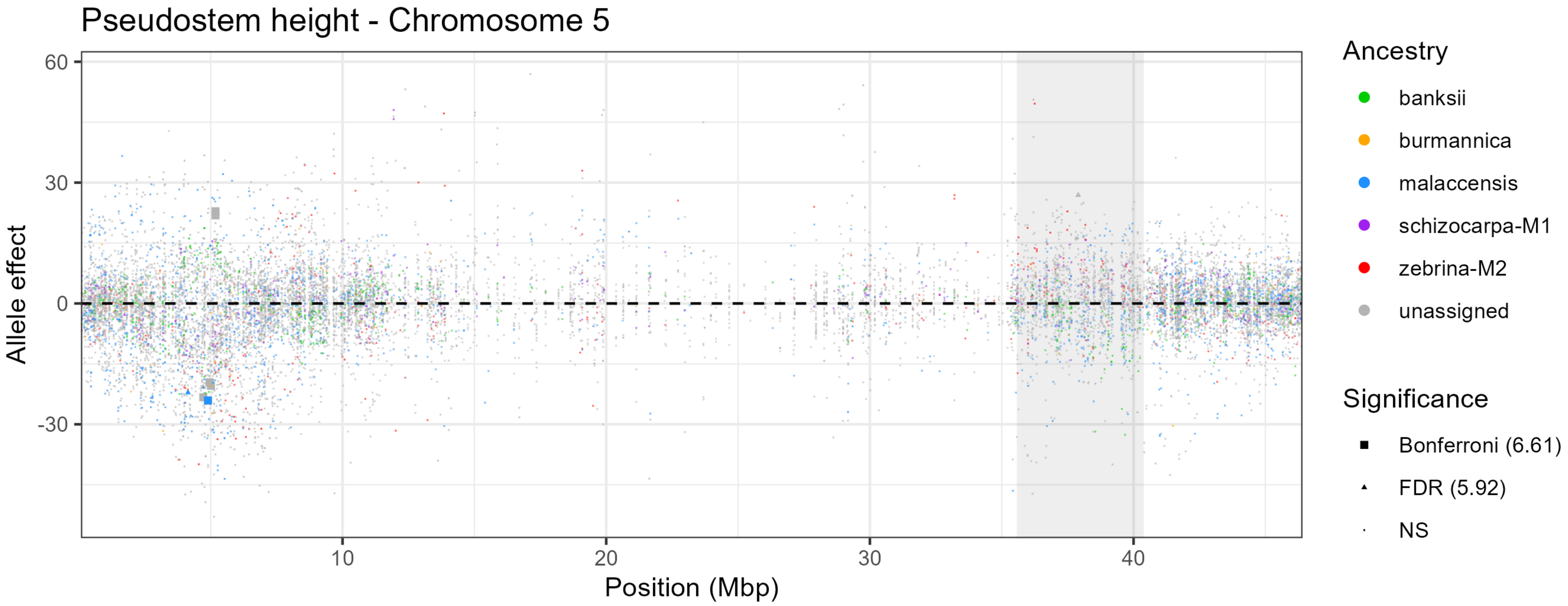


**Figure S4BF**: Estimated allele effects along chromosome 5 for pseudostem height obtained using the K model. Dots are colored according to allele ancestry and shaped according to the level of significance of the test. When no ancestry could be assigned, the effect represented is that of the alternative allele. The QTL interval considered is indicated by a gray area.

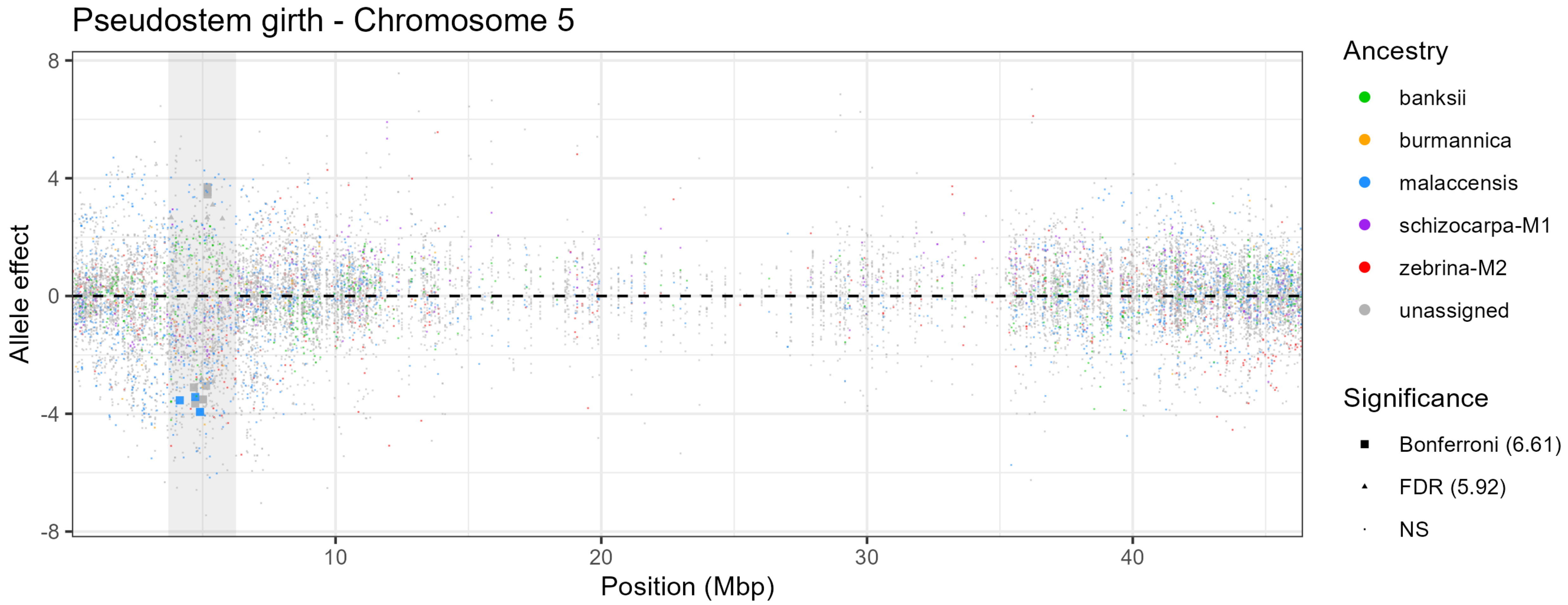


**Figure S4BG**: Estimated allele effects along chromosome 5 for pseudostem girth obtained using the K model. Dots are colored according to allele ancestry and shaped according to the level of significance of the test. When no ancestry could be assigned, the effect represented is that of the alternative allele. The QTL interval considered is indicated by a gray area.

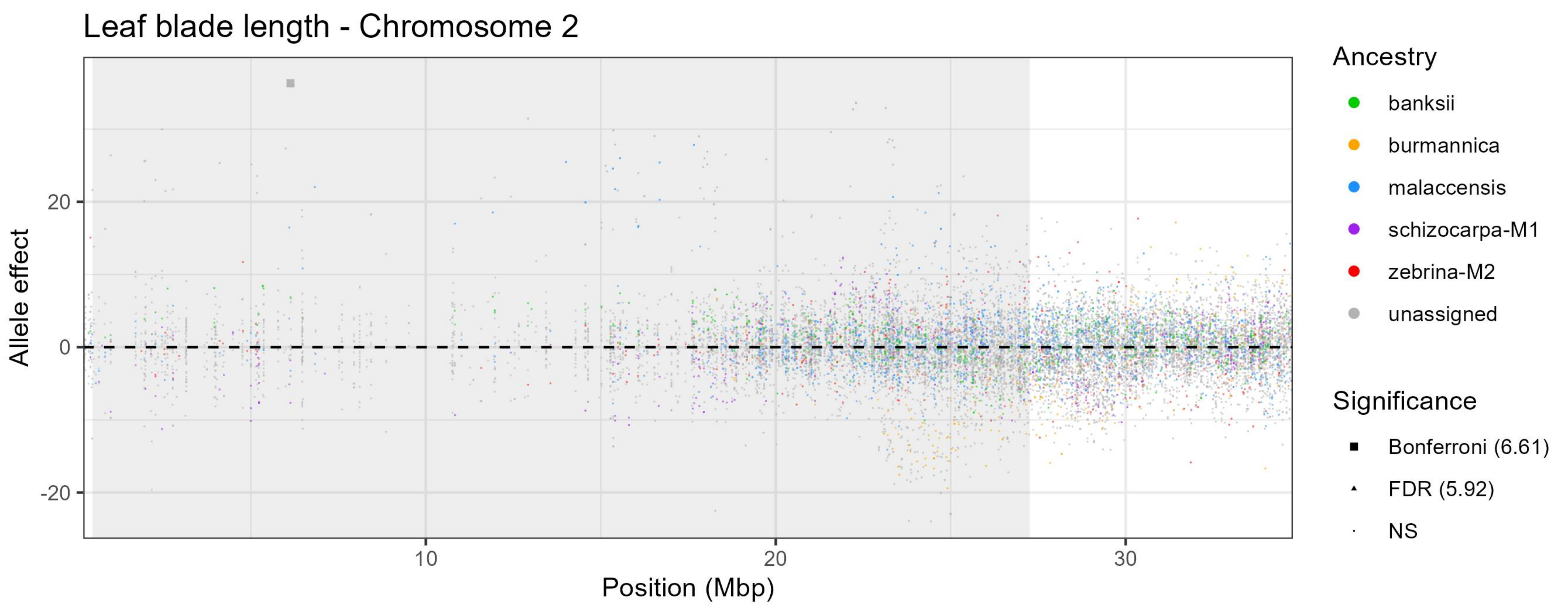


**Figure S4BH**: Estimated allele effects along chromosome 2 for leaf blade length obtained using the K model. Dots are colored according to allele ancestry and shaped according to the level of significance of the test. When no ancestry could be assigned, the effect represented is that of the alternative allele. The QTL interval considered is indicated by a gray area.

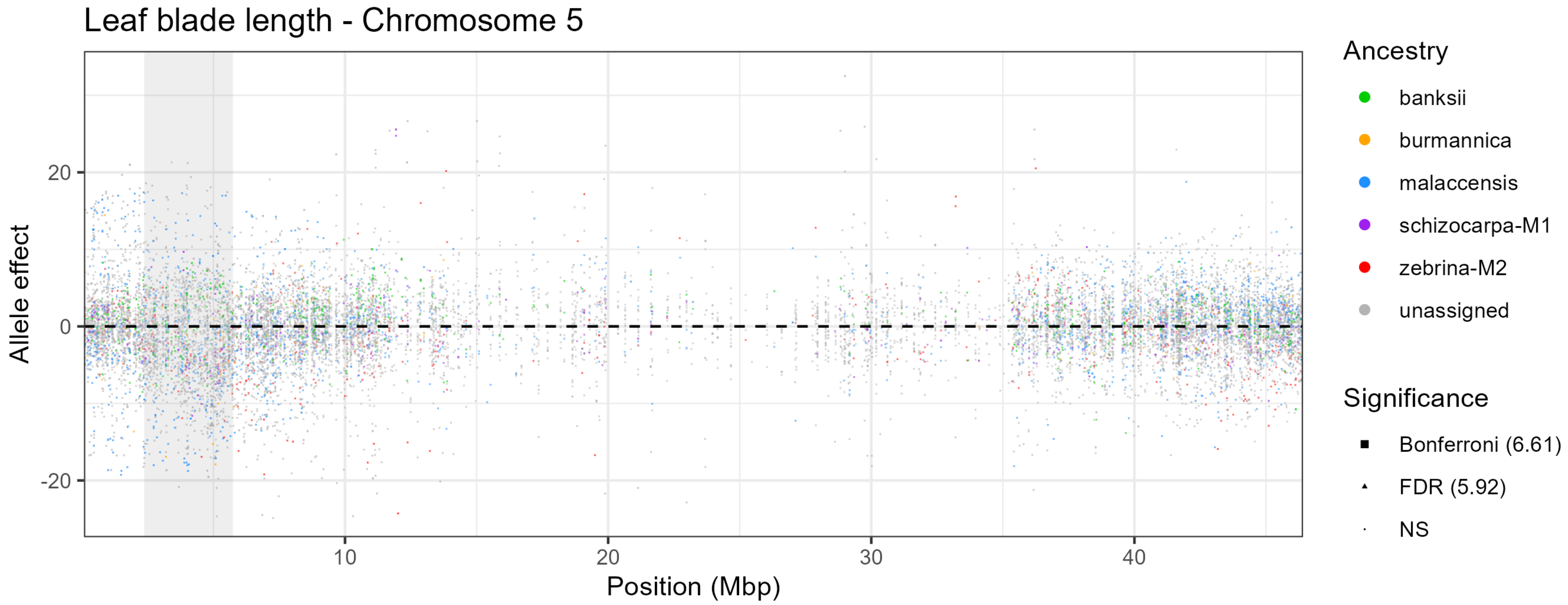


**Figure S4BI**: Estimated allele effects along chromosome 5 for leaf blade length obtained using the K model. Dots are colored according to allele ancestry and shaped according to the level of significance of the test. When no ancestry could be assigned, the effect represented is that of the alternative allele. The QTL interval considered is indicated by a gray area.

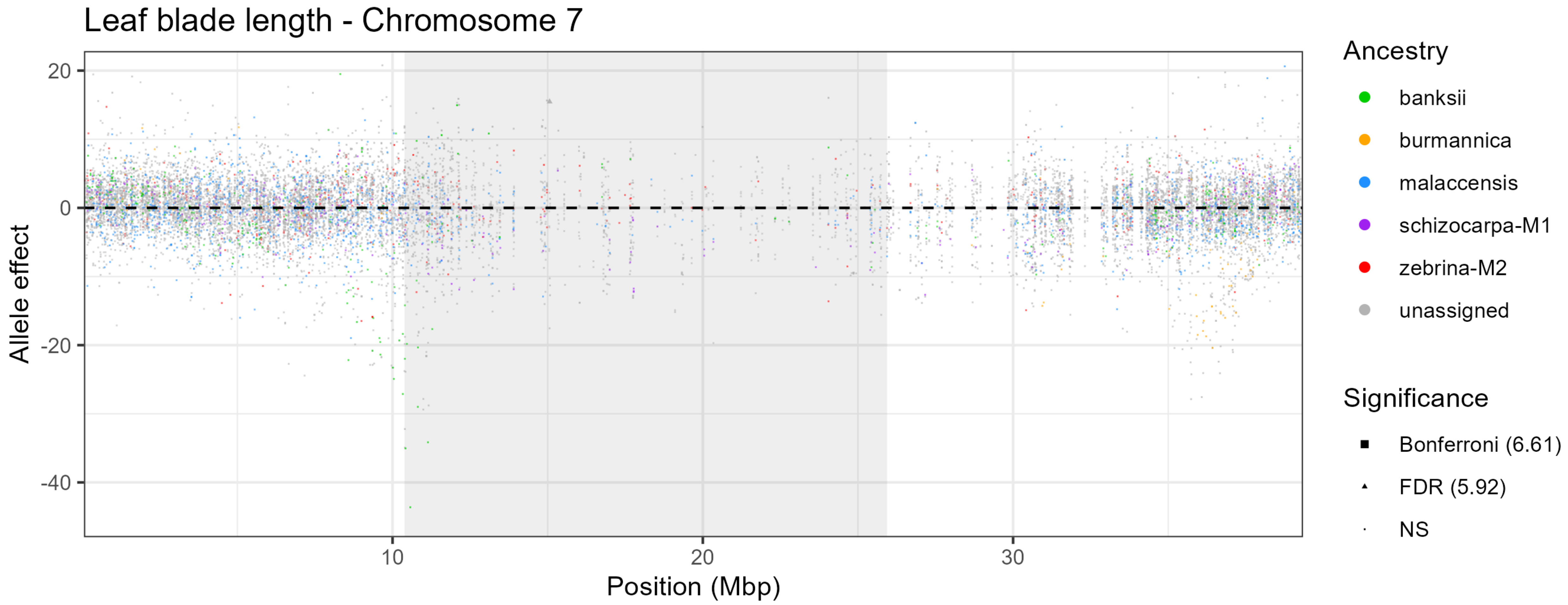


**Figure S4BJ**: Estimated allele effects along chromosome 7 for leaf blade length obtained using the K model. Dots are colored according to allele ancestry and shaped according to the level of significance of the test. When no ancestry could be assigned, the effect represented is that of the alternative allele. The QTL interval considered is indicated by a gray area.

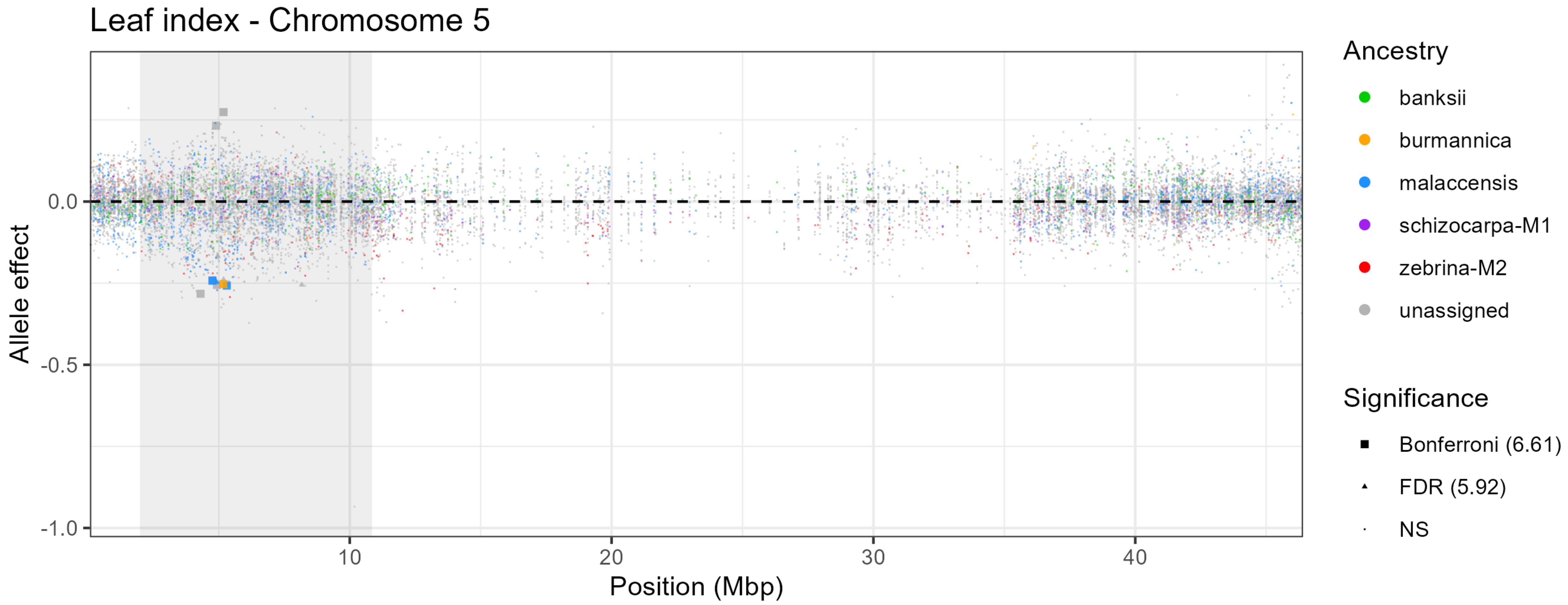


**Figure S4BK**: Estimated allele effects along chromosome 5 for leaf index obtained using the K model. Dots are colored according to allele ancestry and shaped according to the level of significance of the test. When no ancestry could be assigned, the effect represented is that of the alternative allele. The QTL interval considered is indicated by a gray area.

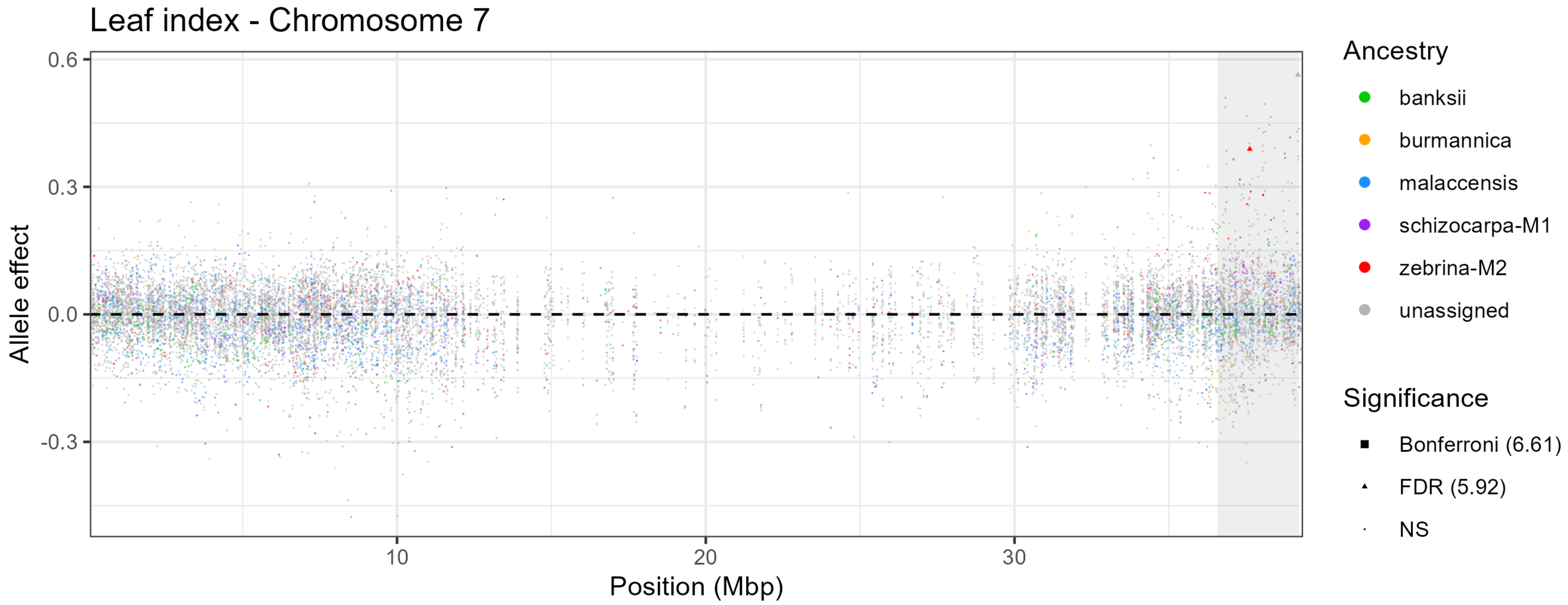


**Figure S4BL**: Estimated allele effects along chromosome 7 for leaf index obtained using the K model. Dots are colored according to allele ancestry and shaped according to the level of significance of the test. When no ancestry could be assigned, the effect represented is that of the alternative allele. The QTL interval considered is indicated by a gray area.

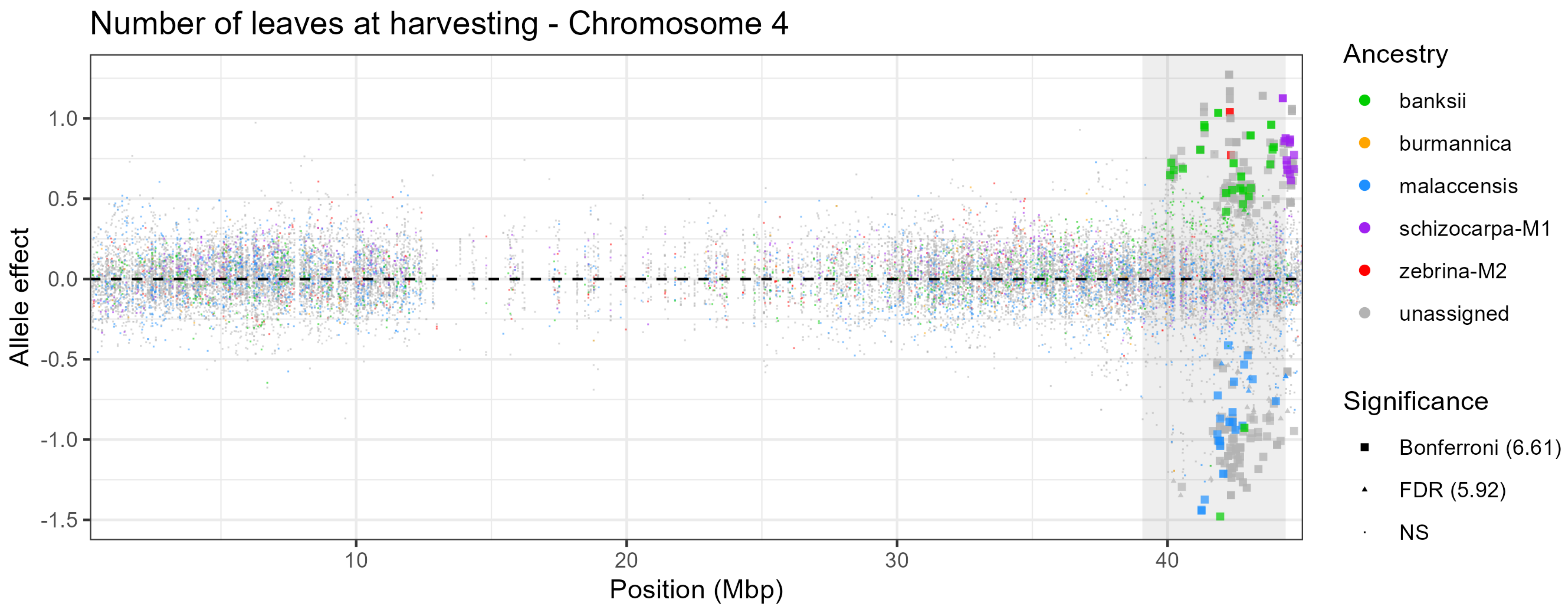


**Figure S4BM**: Estimated allele effects along chromosome 4 for number of leaves at harvesting obtained using the K model. Dots are colored according to allele ancestry and shaped according to the level of significance of the test. When no ancestry could be assigned, the effect represented is that of the alternative allele. The QTL interval considered is indicated by a gray area.

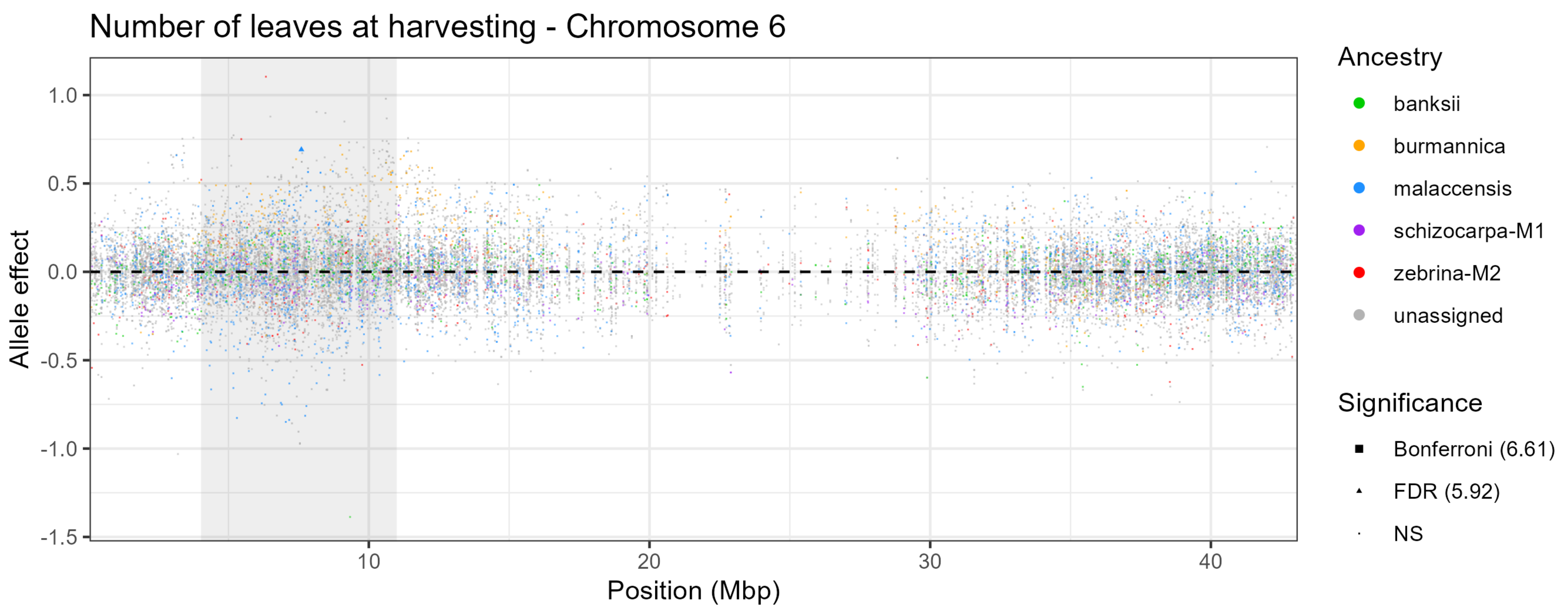


**Figure S4BN**: Estimated allele effects along chromosome 6 for number of leaves at harvesting obtained using the K model. Dots are colored according to allele ancestry and shaped according to the level of significance of the test. When no ancestry could be assigned, the effect represented is that of the alternative allele. The QTL interval considered is indicated by a gray area.

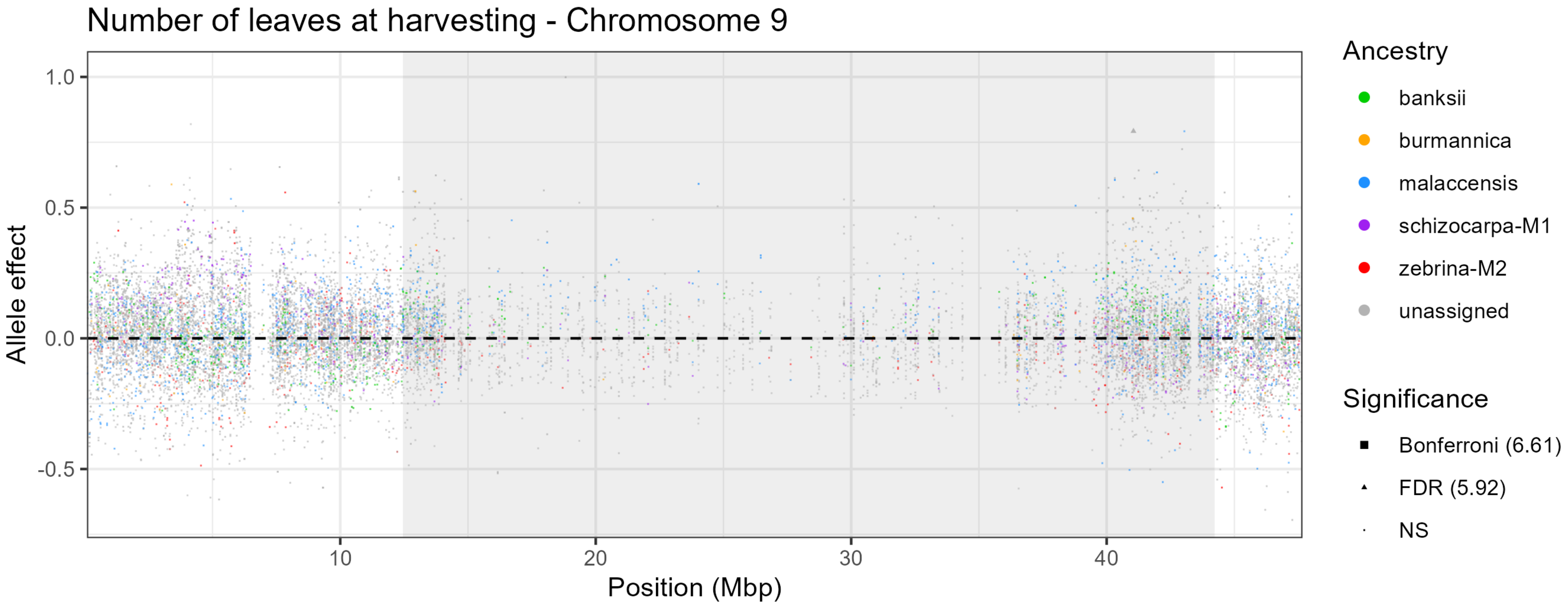


**Figure S4BO**: Estimated allele effects along chromosome 9 for number of leaves at harvesting obtained using the K model. Dots are colored according to allele ancestry and shaped according to the level of significance of the test. When no ancestry could be assigned, the effect represented is that of the alternative allele. The QTL interval considered is indicated by a gray area.

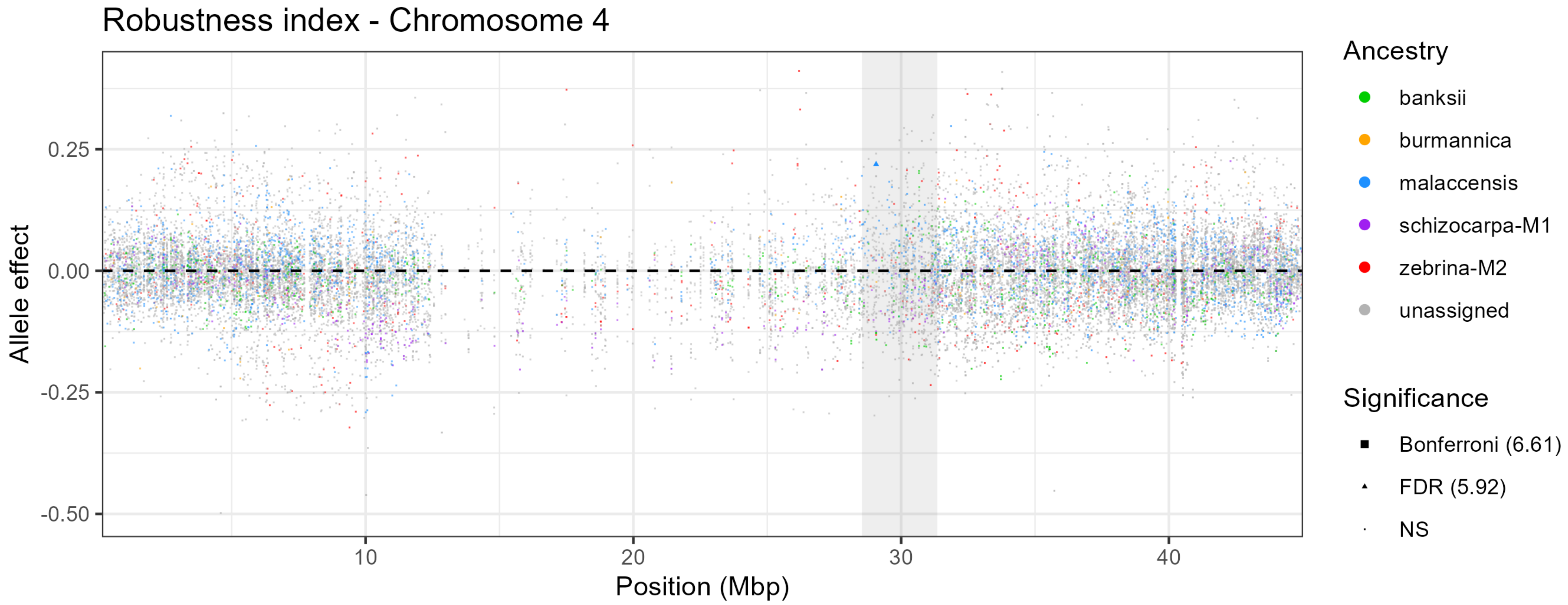


**Figure S4BP**: Estimated allele effects along chromosome 4 for robustness index obtained using the K model. Dots are colored according to allele ancestry and shaped according to the level of significance of the test. When no ancestry could be assigned, the effect represented is that of the alternative allele. The QTL interval considered is indicated by a gray area.

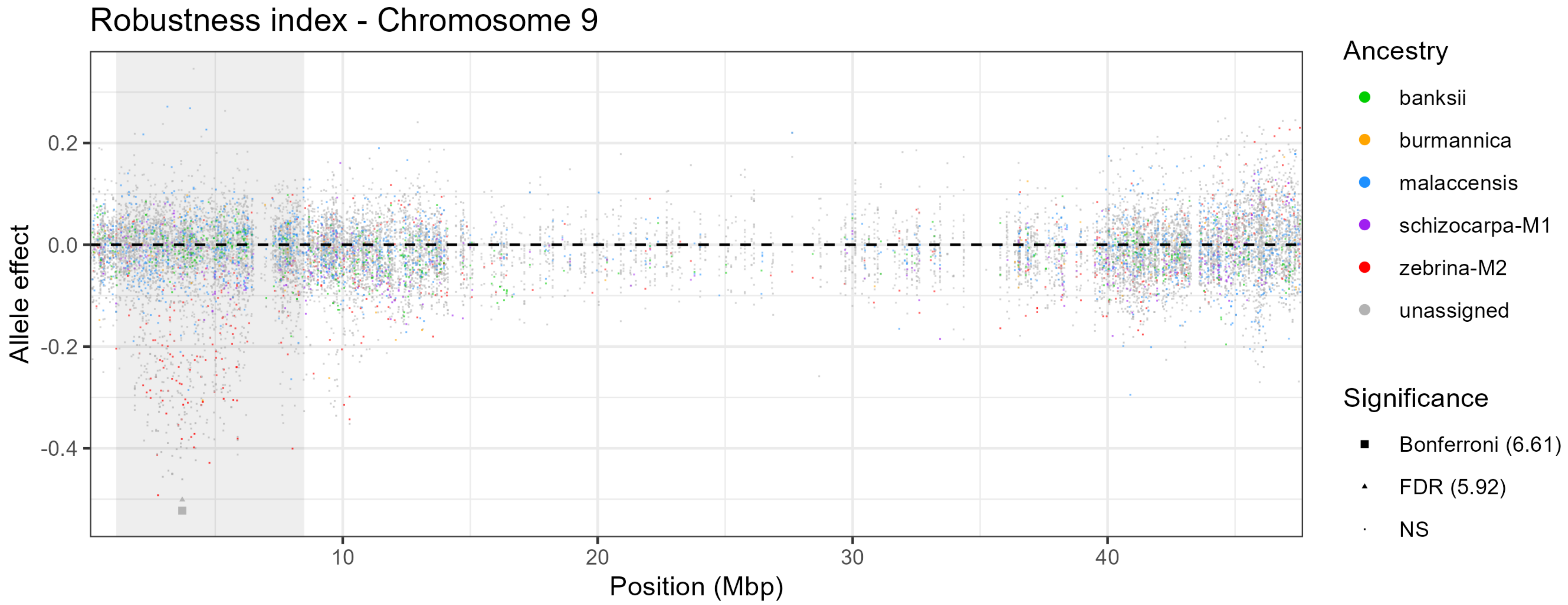


**Figure S4BQ**: Estimated allele effects along chromosome 9 for robustness index obtained using the K model. Dots are colored according to allele ancestry and shaped according to the level of significance of the test. When no ancestry could be assigned, the effect represented is that of the alternative allele. The QTL interval considered is indicated by a gray area.
