# Supplementary material for: Genome-wide association for agro-morphological traits in a triploid banana population with large chromosome rearrangements
Source: Hortic Res. 2024 Nov 6;12(2):uhae307. doi: 10.1093/hr/uhae307 (PMC11817881; doi:10.1093/hr/uhae307)
Supplement: Web_Material_uhae307 [file web_material_uhae307.zip › Figure_S5.pdf]

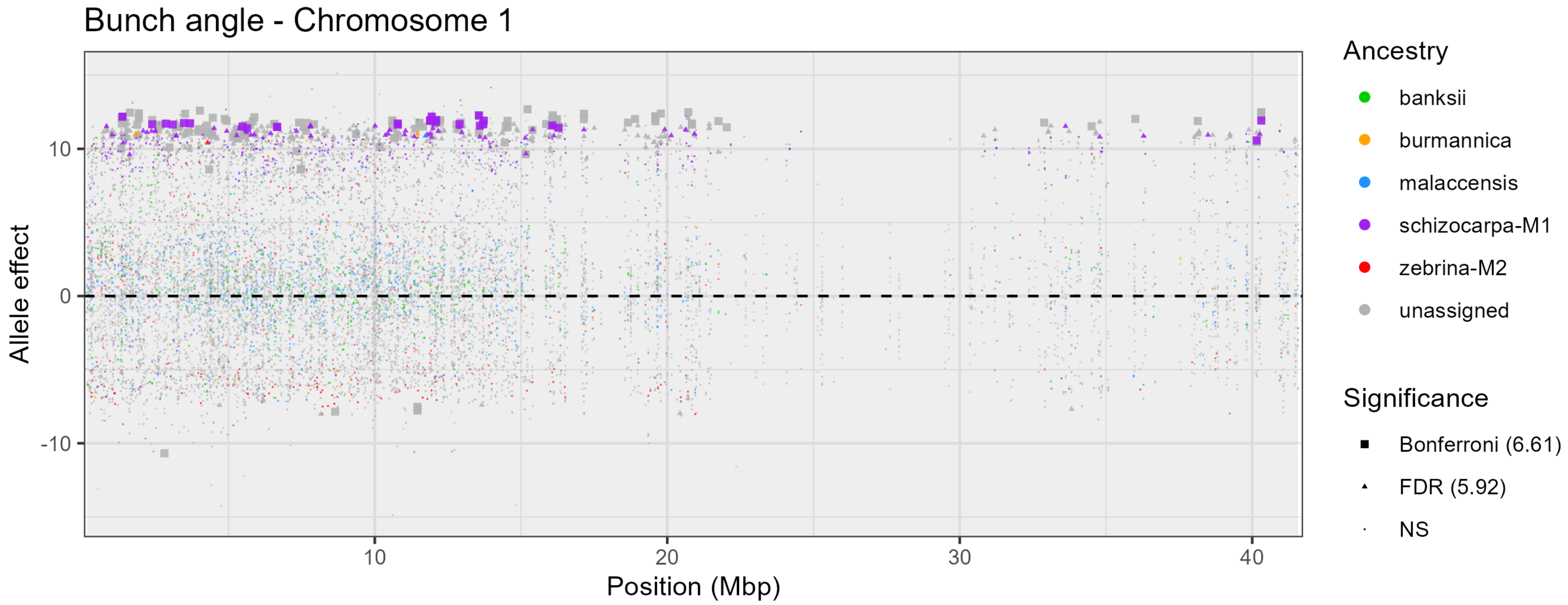

Figure S5AA: Estimated allele effects along chromosome 1 for bunch angle obtained using the Kc model. Dots are colored according to allele ancestry and shaped according to the level of significance of the test. When no ancestry could be assigned, the effect represented is that of the alternative allele. The QTL interval considered is indicated by a gray area.

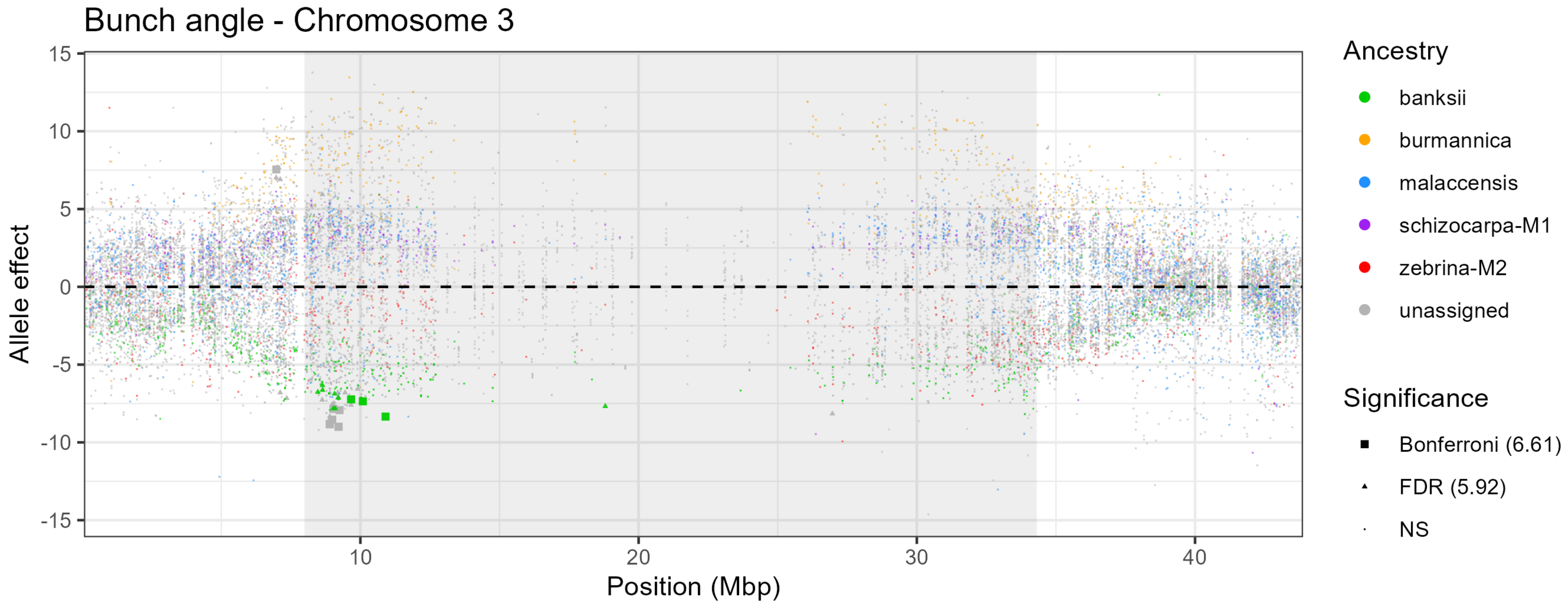

Figure S5AB: Estimated allele effects along chromosome 3 for bunch angle obtained using the Kc model. Dots are colored according to allele ancestry and shaped according to the level of significance of the test. When no ancestry could be assigned, the effect represented is that of the alternative allele. The QTL interval considered is indicated by a gray area.

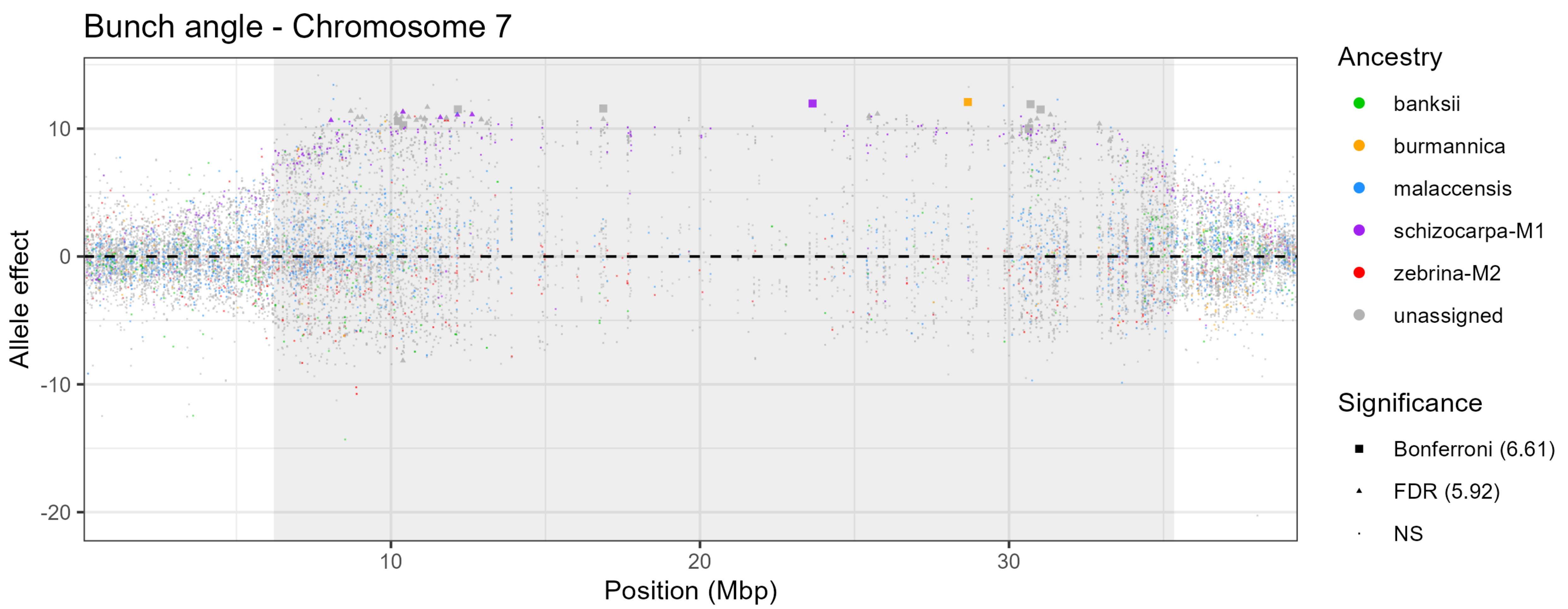

Figure S5AC: Estimated allele effects along chromosome 7 for bunch angle obtained using the Kc model. Dots are colored according to allele ancestry and shaped according to the level of significance of the test. When no ancestry could be assigned, the effect represented is that of the alternative allele. The QTL interval considered is indicated by a gray area.

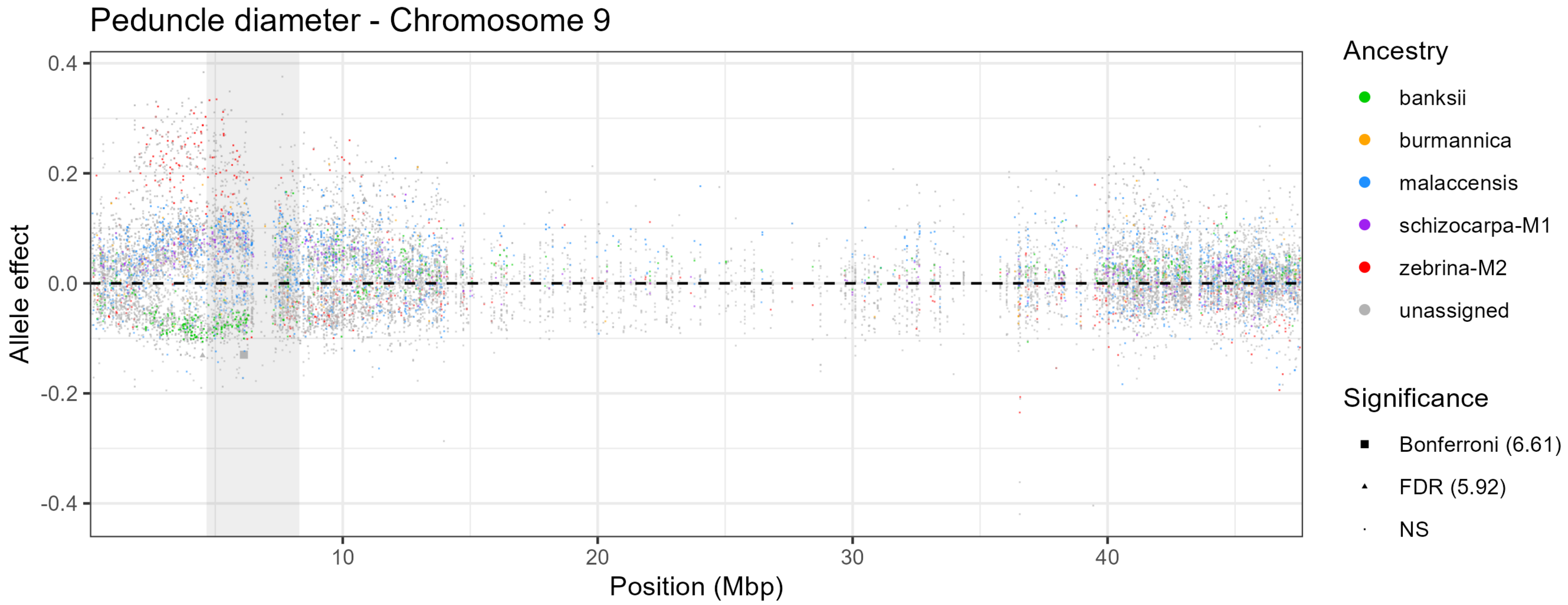

Figure S5AD: Estimated allele effects along chromosome 9 for peduncle diameter obtained using the Kc model. Dots are colored according to allele ancestry and shaped according to the level of significance of the test. When no ancestry could be assigned, the effect represented is that of the alternative allele. The QTL interval considered is indicated by a gray area.

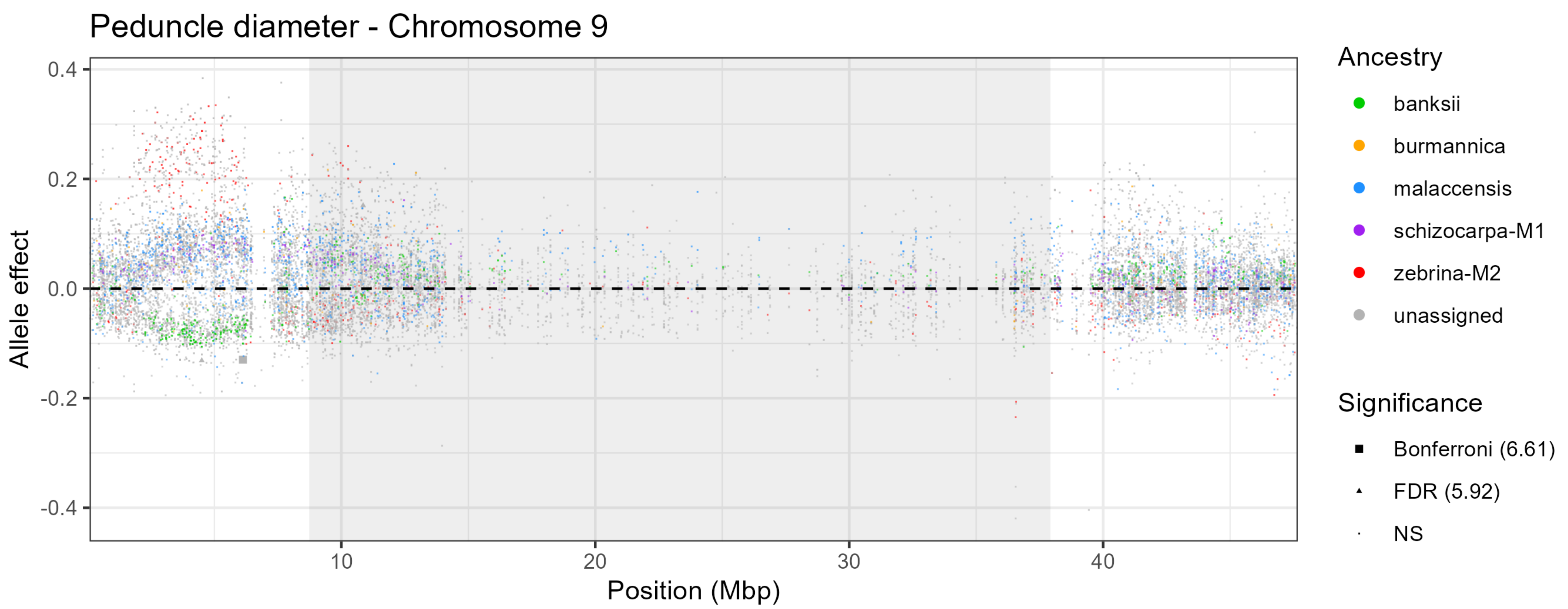

Figure S5AE: Estimated allele effects along chromosome 9 for peduncle diameter obtained using the Kc model. Dots are colored according to allele ancestry and shaped according to the level of significance of the test. When no ancestry could be assigned, the effect represented is that of the alternative allele. The QTL interval considered is indicated by a gray area.

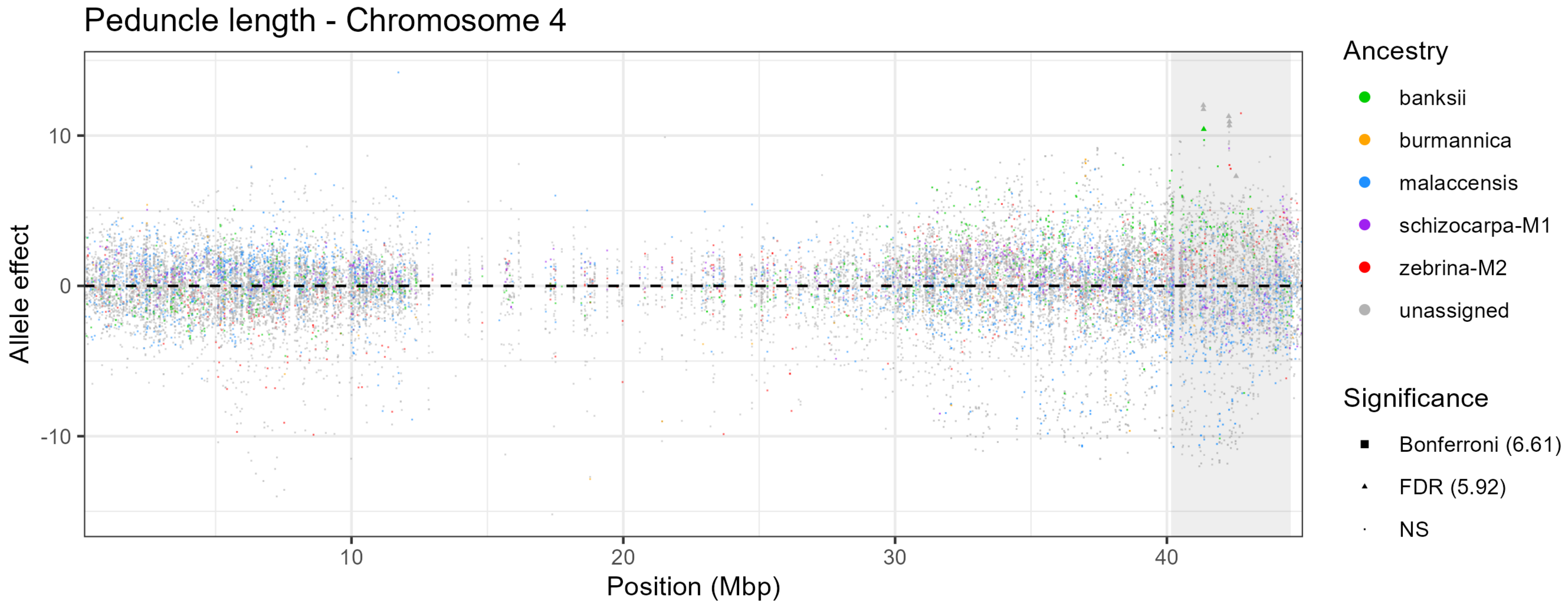

Figure S5AF: Estimated allele effects along chromosome 4 for peduncle length obtained using the Kc model. Dots are colored according to allele ancestry and shaped according to the level of significance of the test. When no ancestry could be assigned, the effect represented is that of the alternative allele. The QTL interval considered is indicated by a gray area.

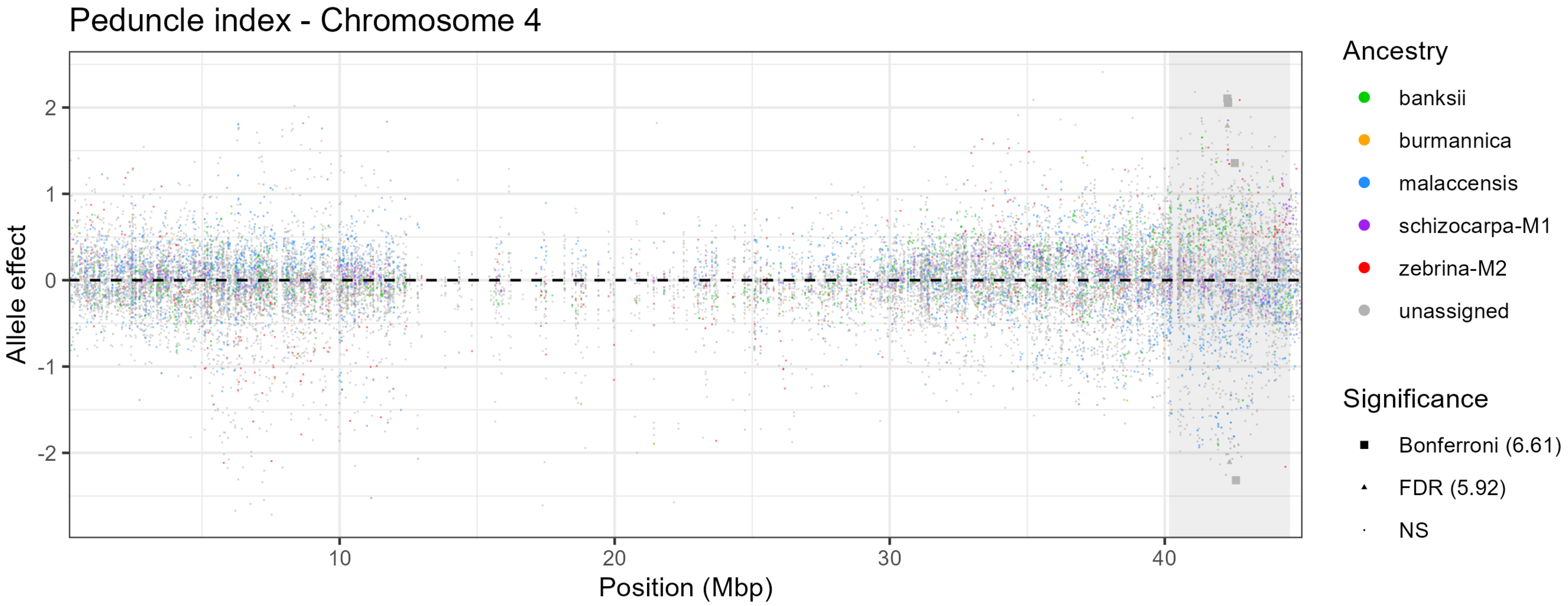

Figure S5AG: Estimated allele effects along chromosome 4 for peduncle index obtained using the Kc model. Dots are colored according to allele ancestry and shaped according to the level of significance of the test. When no ancestry could be assigned, the effect represented is that of the alternative allele. The QTL interval considered is indicated by a gray area.

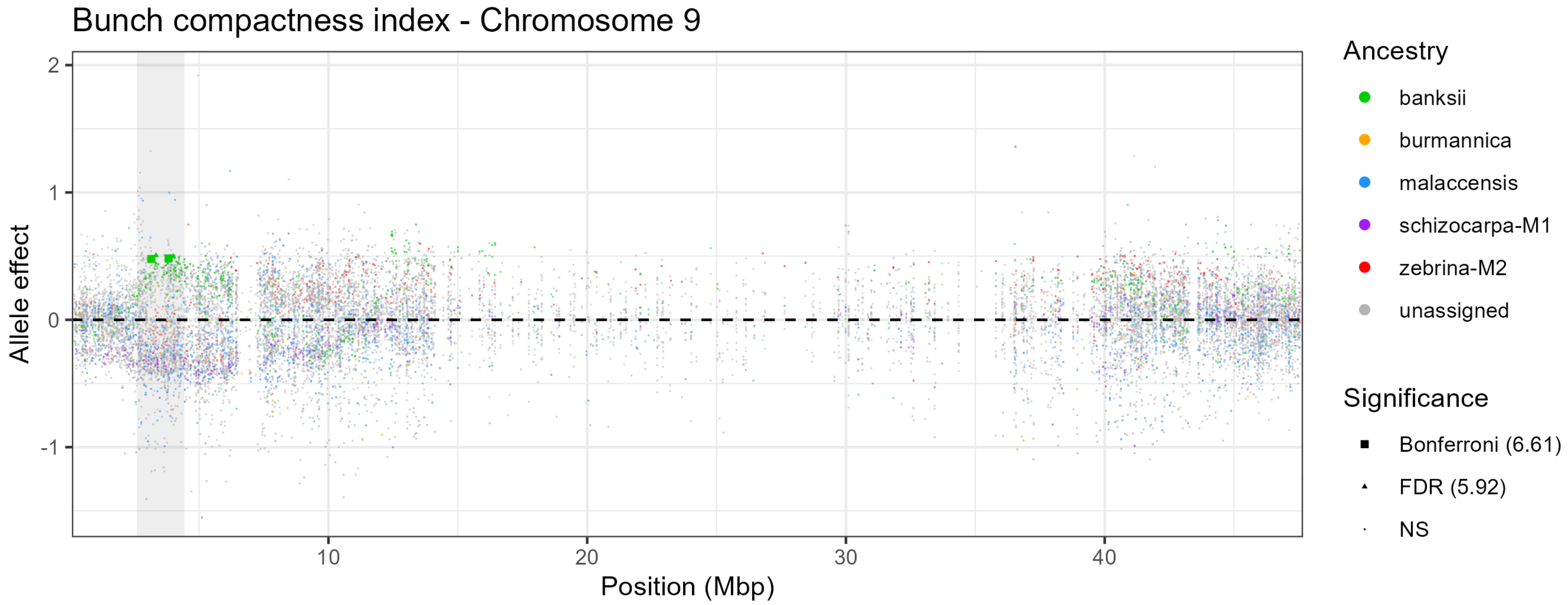

Figure S5AH: Estimated allele effects along chromosome 9 for bunch compactness index obtained using the Kc model. Dots are colored according to allele ancestry and shaped according to the level of significance of the test. When no ancestry could be assigned, the effect represented is that of the alternative allele. The QTL interval considered is indicated by a gray area.

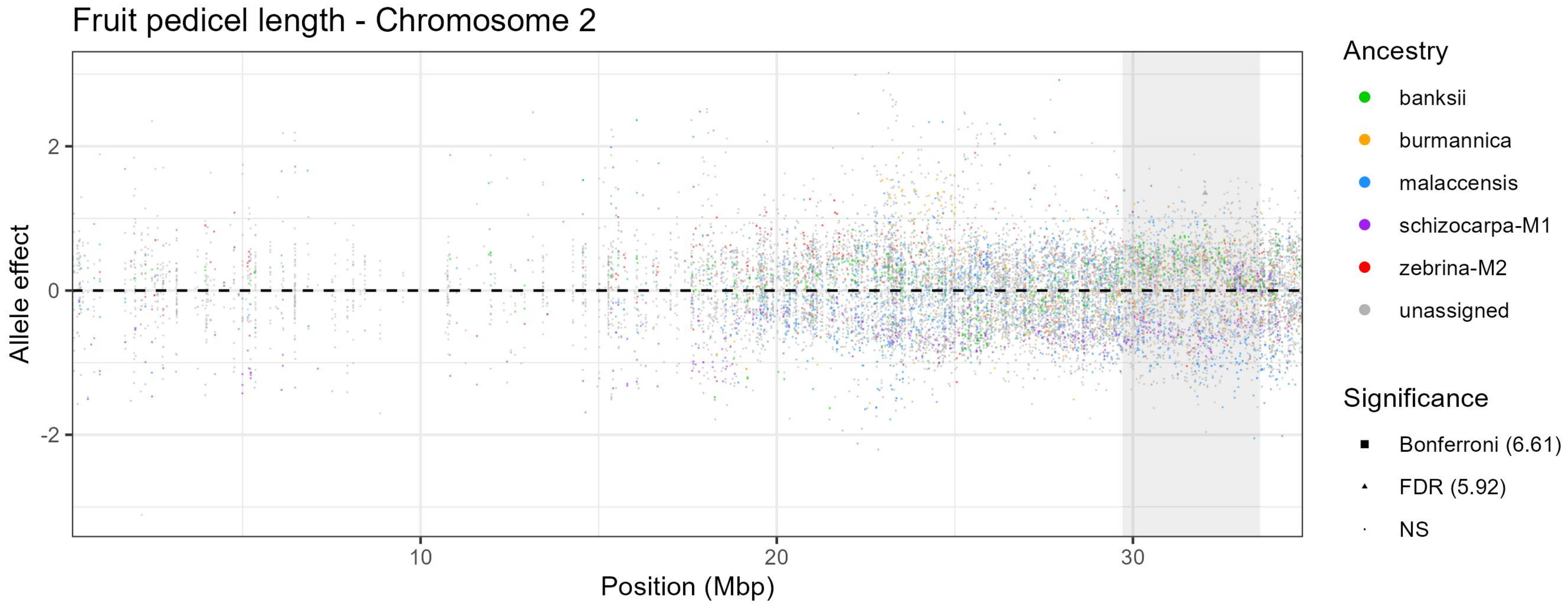

Figure S5AI: Estimated allele effects along chromosome 2 for peduncle length obtained using the Kc model. Dots are colored according to allele ancestry and shaped according to the level of significance of the test. When no ancestry could be assigned, the effect represented is that of the alternative allele. The QTL interval considered is indicated by a gray area.

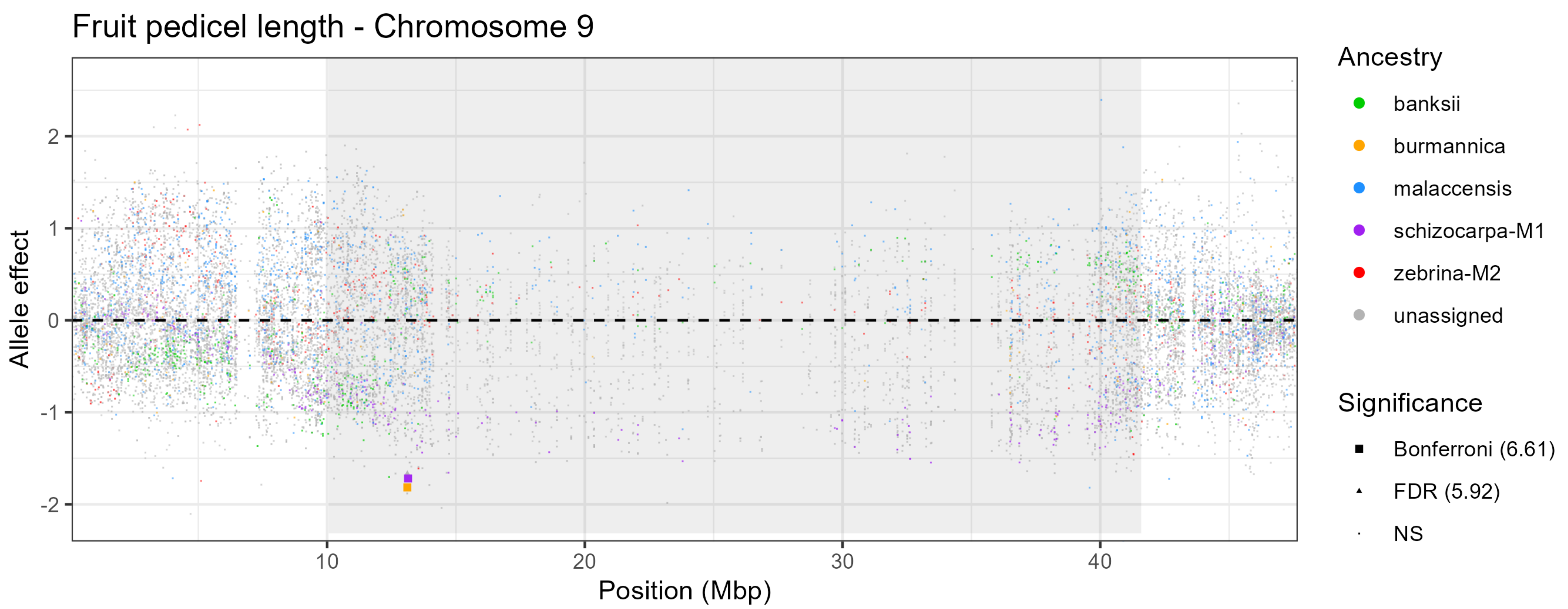

Figure S5AJ: Estimated allele effects along chromosome 9 for peduncle length obtained using the Kc model. Dots are colored according to allele ancestry and shaped according to the level of significance of the test. When no ancestry could be assigned, the effect represented is that of the alternative allele. The QTL interval considered is indicated by a gray area.

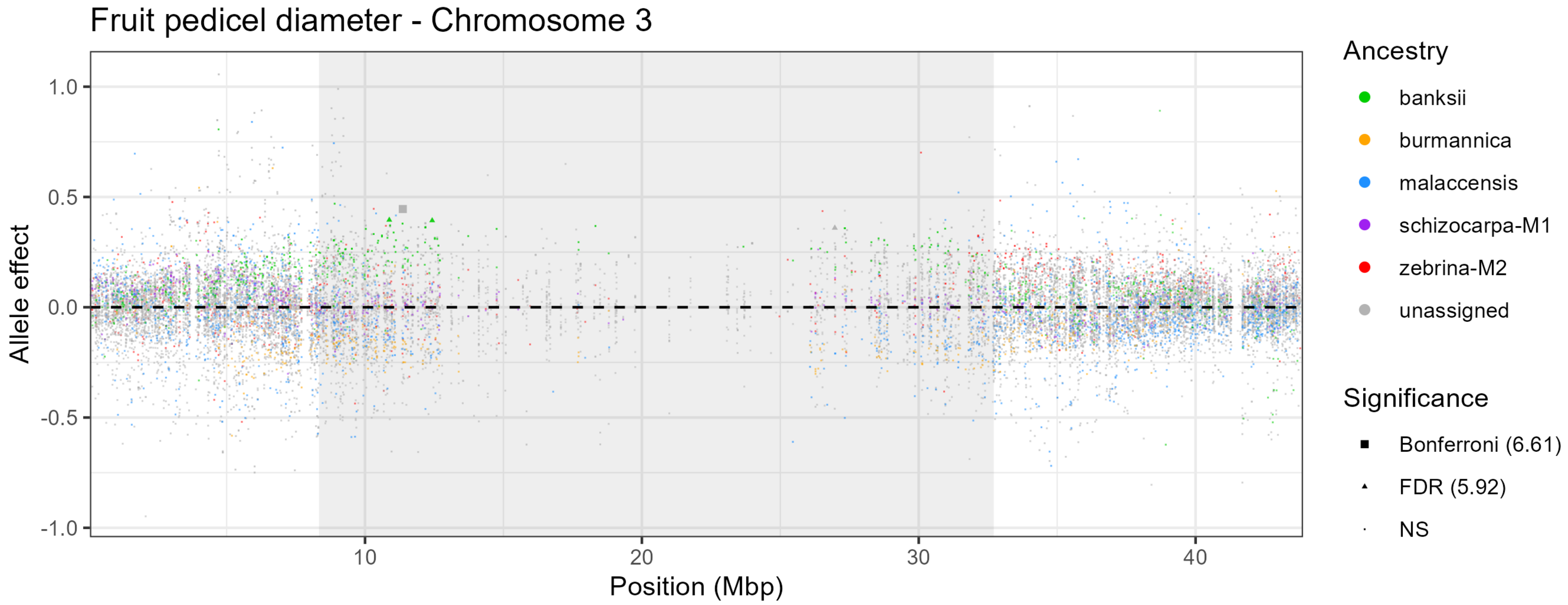

Figure S5AK: Estimated allele effects along chromosome 9 for fruit pedicel diameter obtained using the Kc model. Dots are colored according to allele ancestry and shaped according to the level of significance of the test. When no ancestry could be assigned, the effect represented is that of the alternative allele. The QTL interval considered is indicated by a gray area.

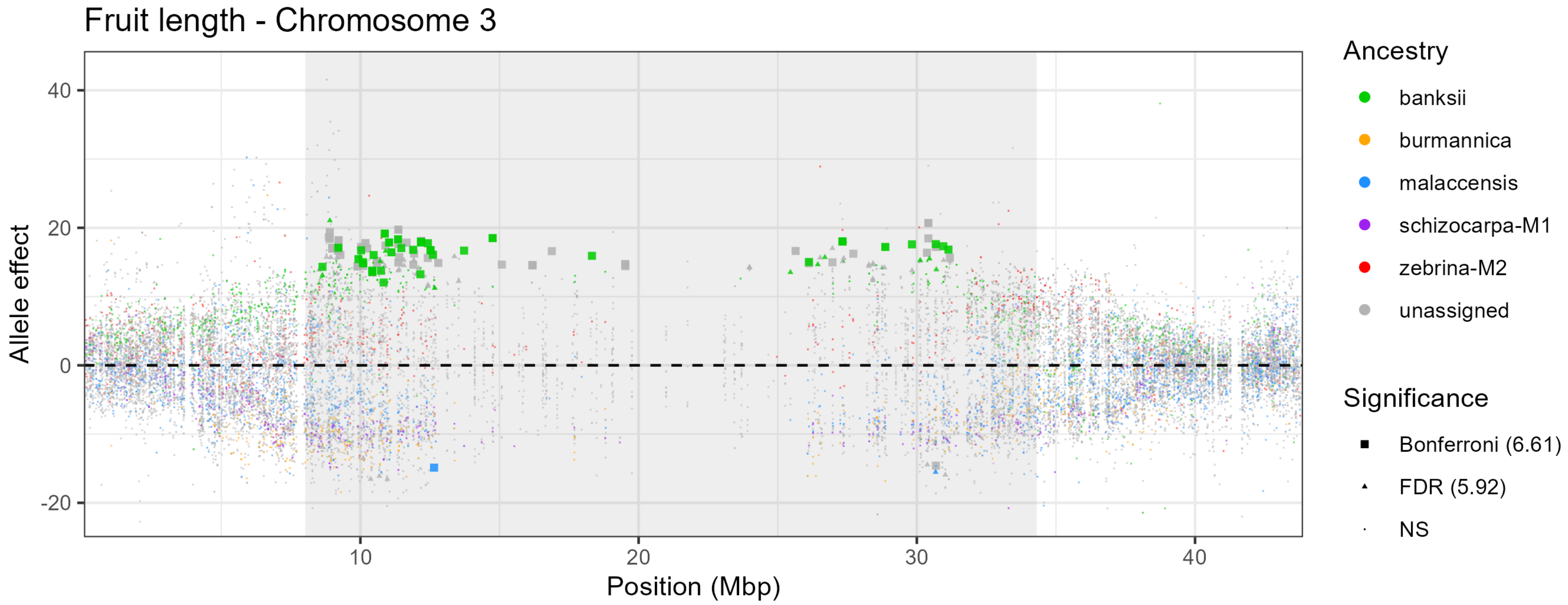

Figure S5AL: Estimated allele effects along chromosome 3 for fruit length obtained using the Kc model. Dots are colored according to allele ancestry and shaped according to the level of significance of the test. When no ancestry could be assigned, the effect represented is that of the alternative allele. The QTL interval considered is indicated by a gray area.

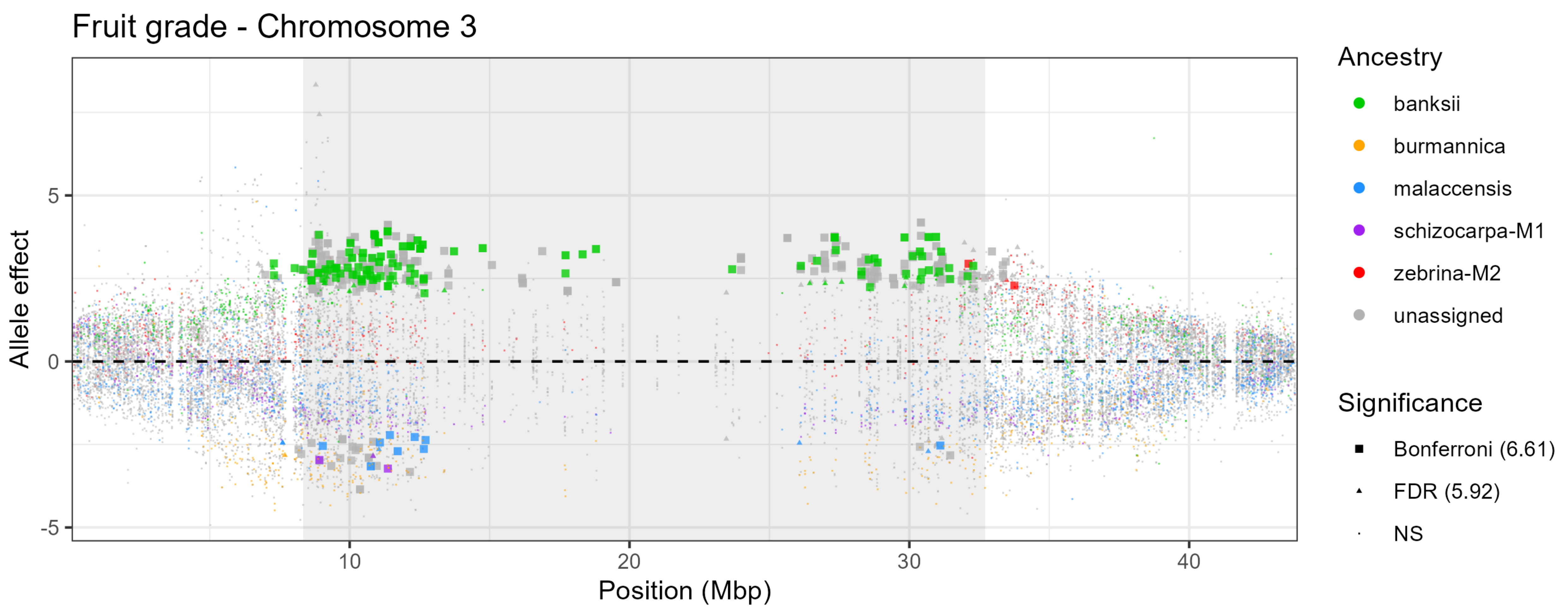

Figure S5AM: Estimated allele effects along chromosome 3 for fruit grade obtained using the Kc model. Dots are colored according to allele ancestry and shaped according to the level of significance of the test. When no ancestry could be assigned, the effect represented is that of the alternative allele. The QTL interval considered is indicated by a gray area.

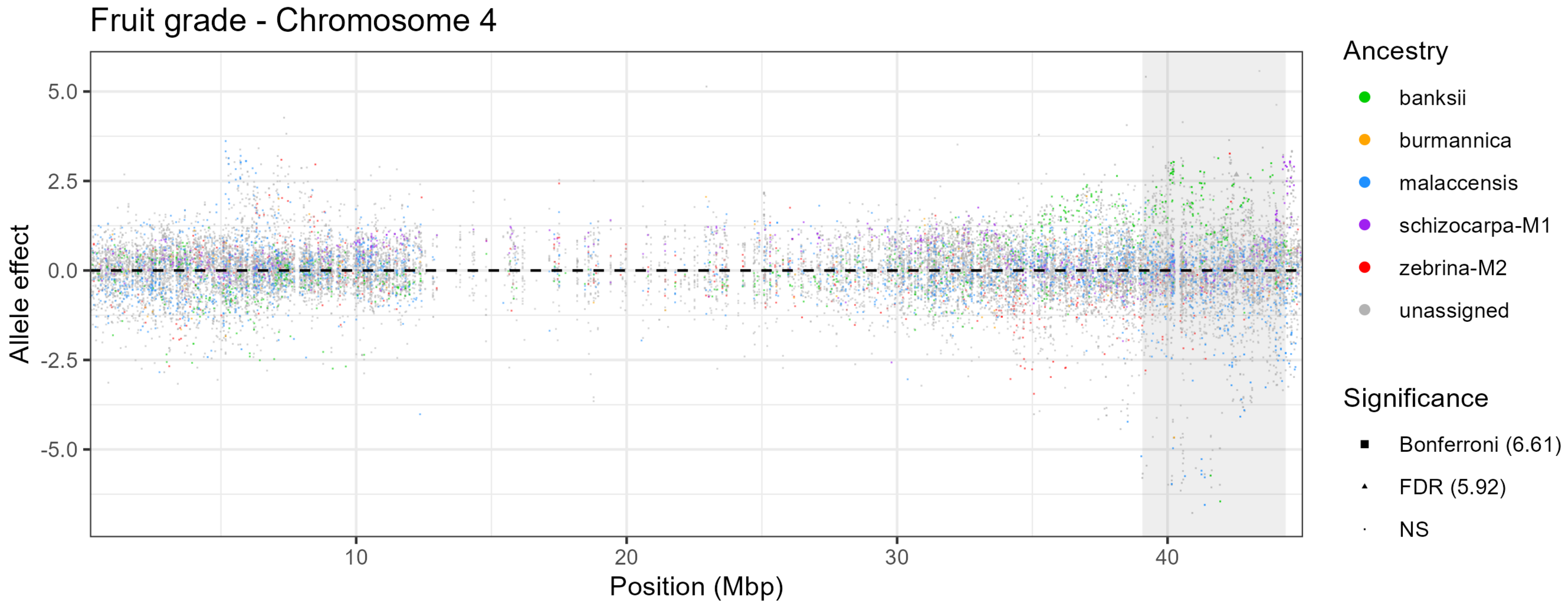

Figure S5AN: Estimated allele effects along chromosome 4 for fruit grade obtained using the Kc model. Dots are colored according to allele ancestry and shaped according to the level of significance of the test. When no ancestry could be assigned, the effect represented is that of the alternative allele. The QTL interval considered is indicated by a gray area.

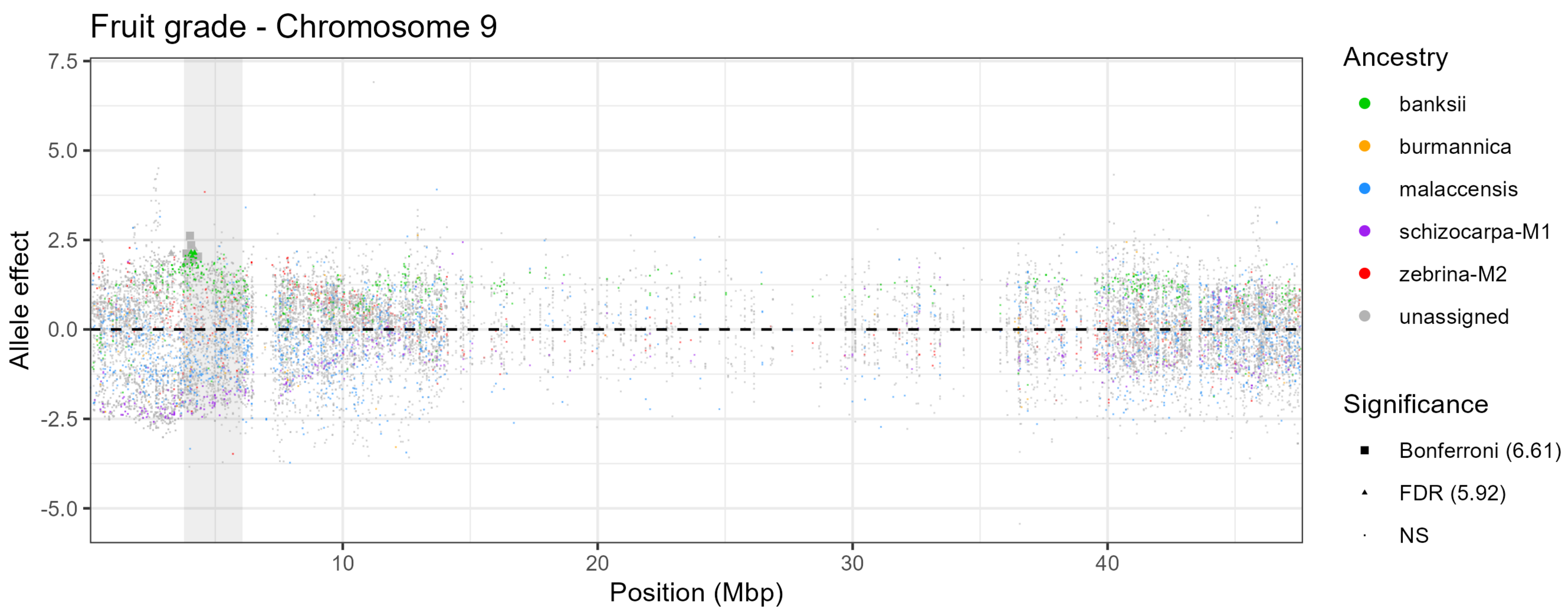

Figure S5AO: Estimated allele effects along chromosome 9 for fruit grade obtained using the Kc model. Dots are colored according to allele ancestry and shaped according to the level of significance of the test. When no ancestry could be assigned, the effect represented is that of the alternative allele. The QTL interval considered is indicated by a gray area.

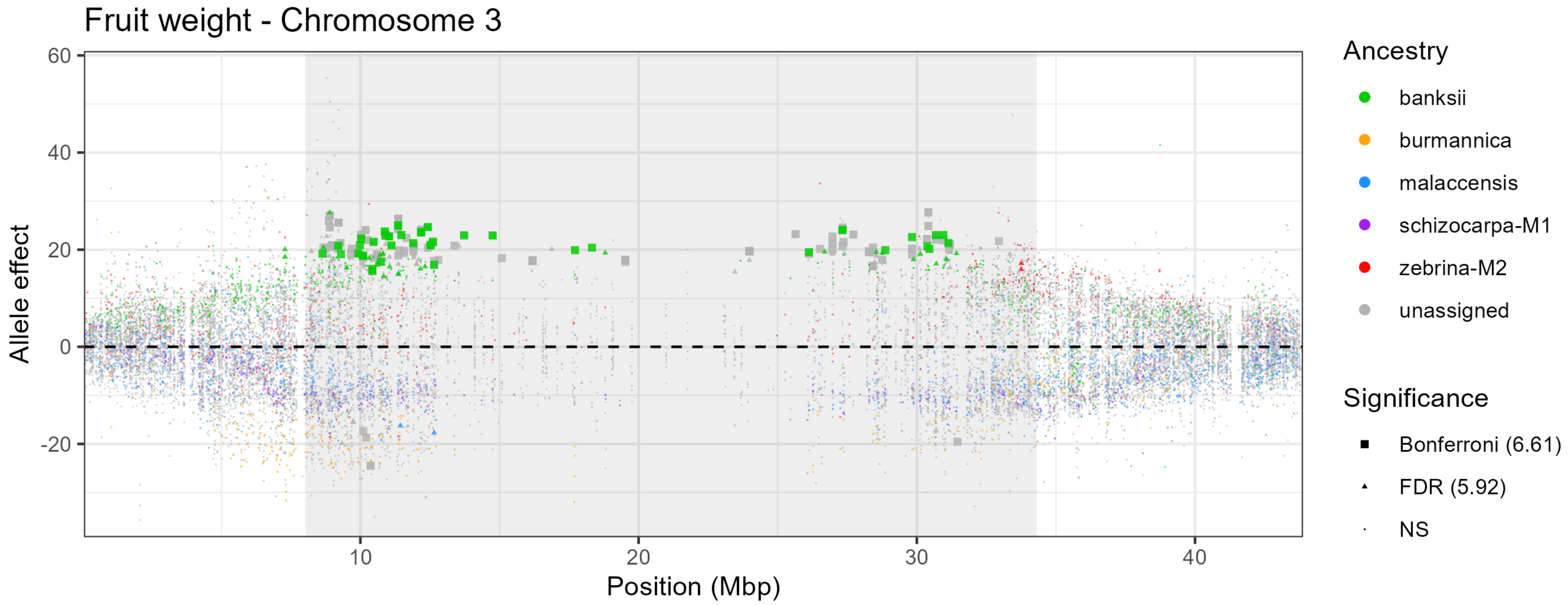

Figure S5AP: Estimated allele effects along chromosome 3 for fruit weight obtained using the Kc model. Dots are colored according to allele ancestry and shaped according to the level of significance of the test. When no ancestry could be assigned, the effect represented is that of the alternative allele. The QTL interval considered is indicated by a gray area.

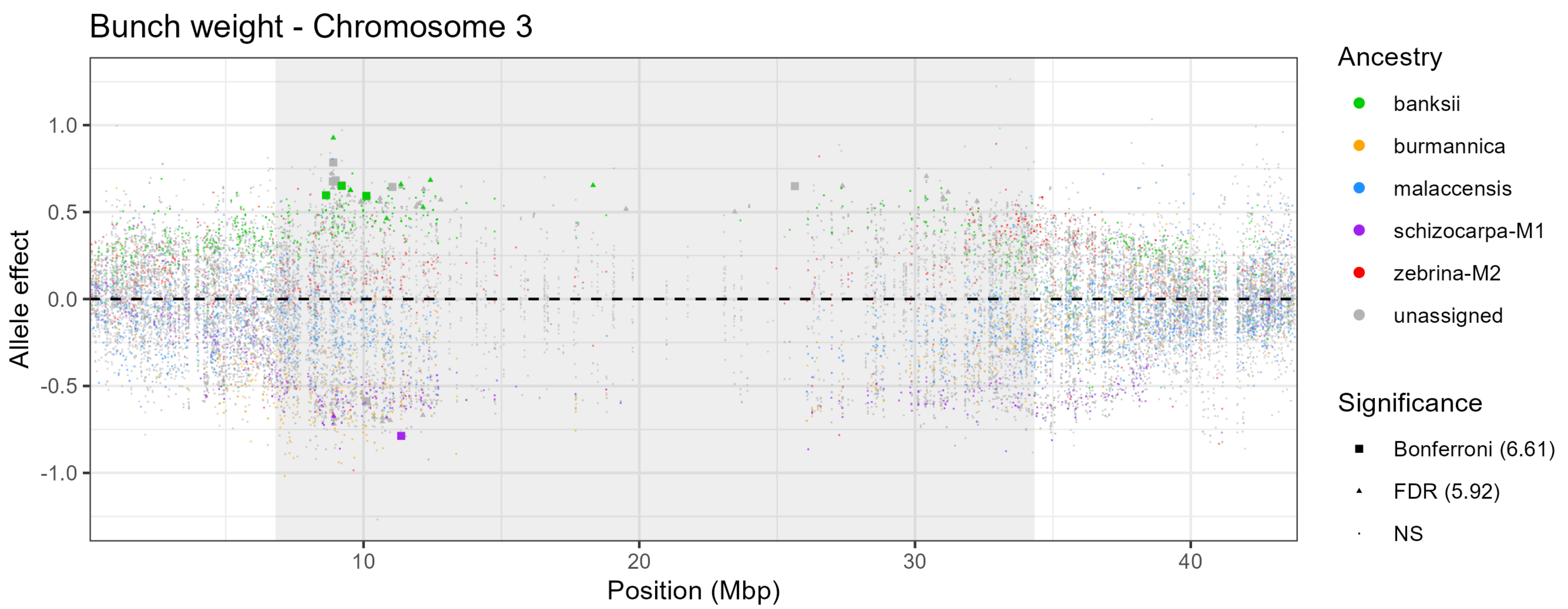

Figure S5AQ: Estimated allele effects along chromosome 3 for bunch weight obtained using the Kc model. Dots are colored according to allele ancestry and shaped according to the level of significance of the test. When no ancestry could be assigned, the effect represented is that of the alternative allele. The QTL interval considered is indicated by a gray area.

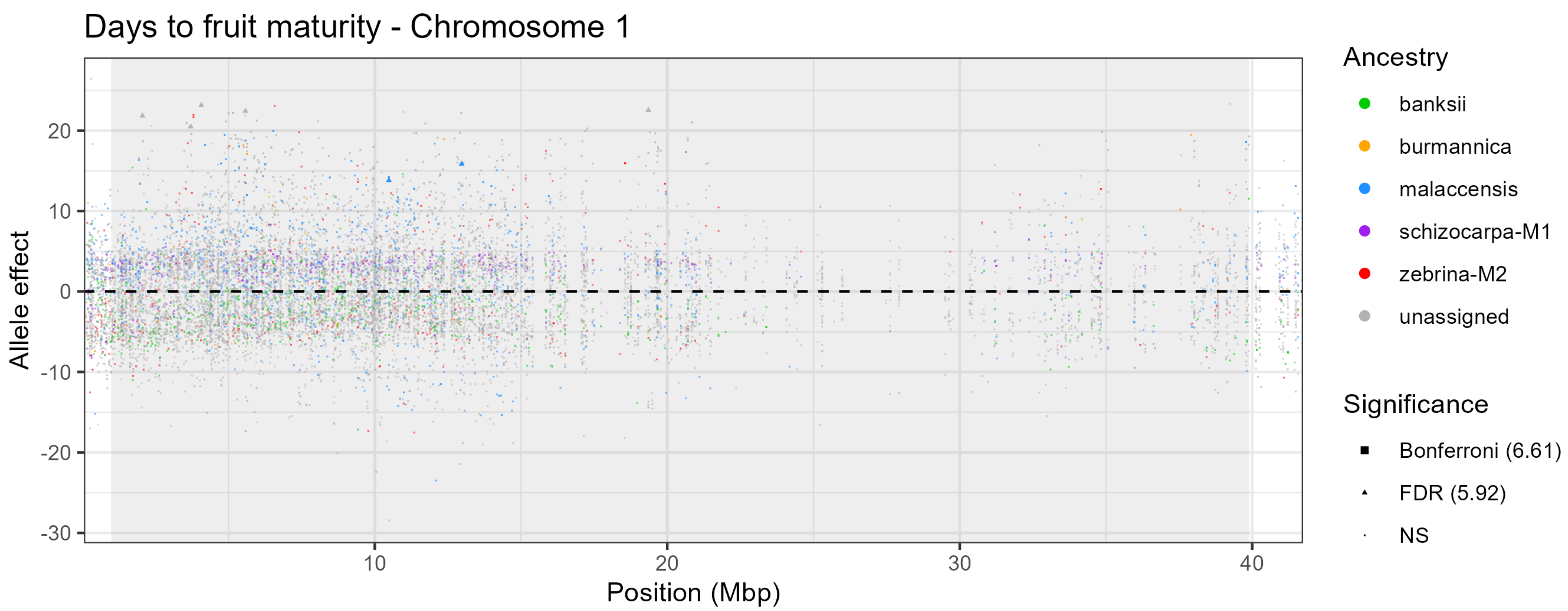

Figure S5AR: Estimated allele effects along chromosome 1 for days to fruit maturity obtained using the Kc model. Dots are colored according to allele ancestry and shaped according to the level of significance of the test. When no ancestry could be assigned, the effect represented is that of the alternative allele. The QTL interval considered is indicated by a gray area.

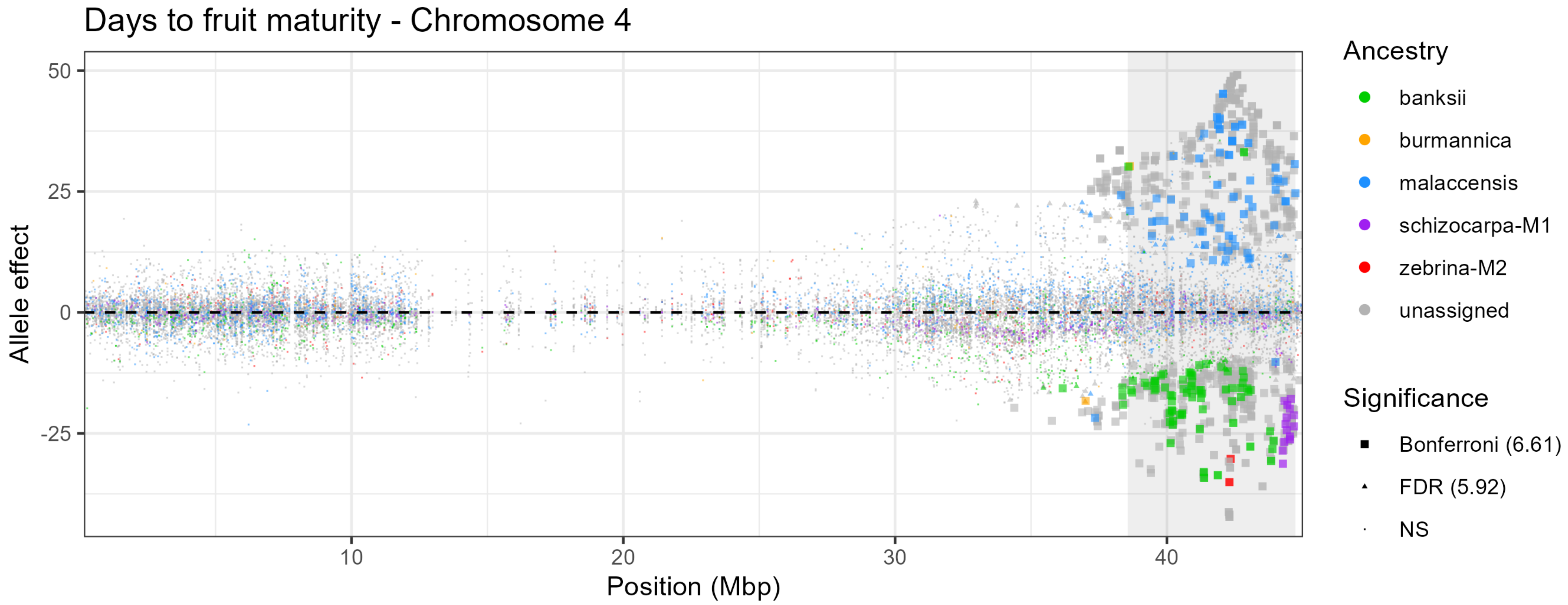

Figure S5AS: Estimated allele effects along chromosome 4 for days to fruit maturity obtained using the Kc model. Dots are colored according to allele ancestry and shaped according to the level of significance of the test. When no ancestry could be assigned, the effect represented is that of the alternative allele. The QTL interval considered is indicated by a gray area.

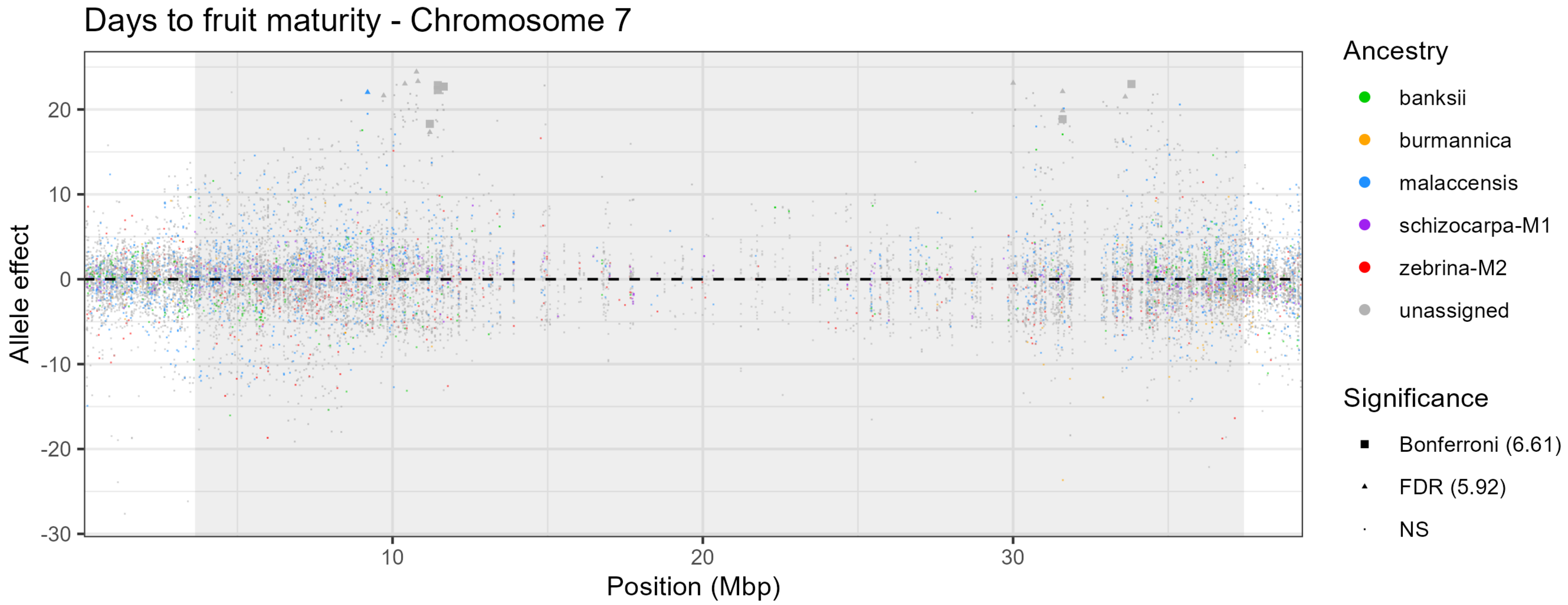

Figure S5AT: Estimated allele effects along chromosome 7 for days to fruit maturity obtained using the Kc model. Dots are colored according to allele ancestry and shaped according to the level of significance of the test. When no ancestry could be assigned, the effect represented is that of the alternative allele. The QTL interval considered is indicated by a gray area.

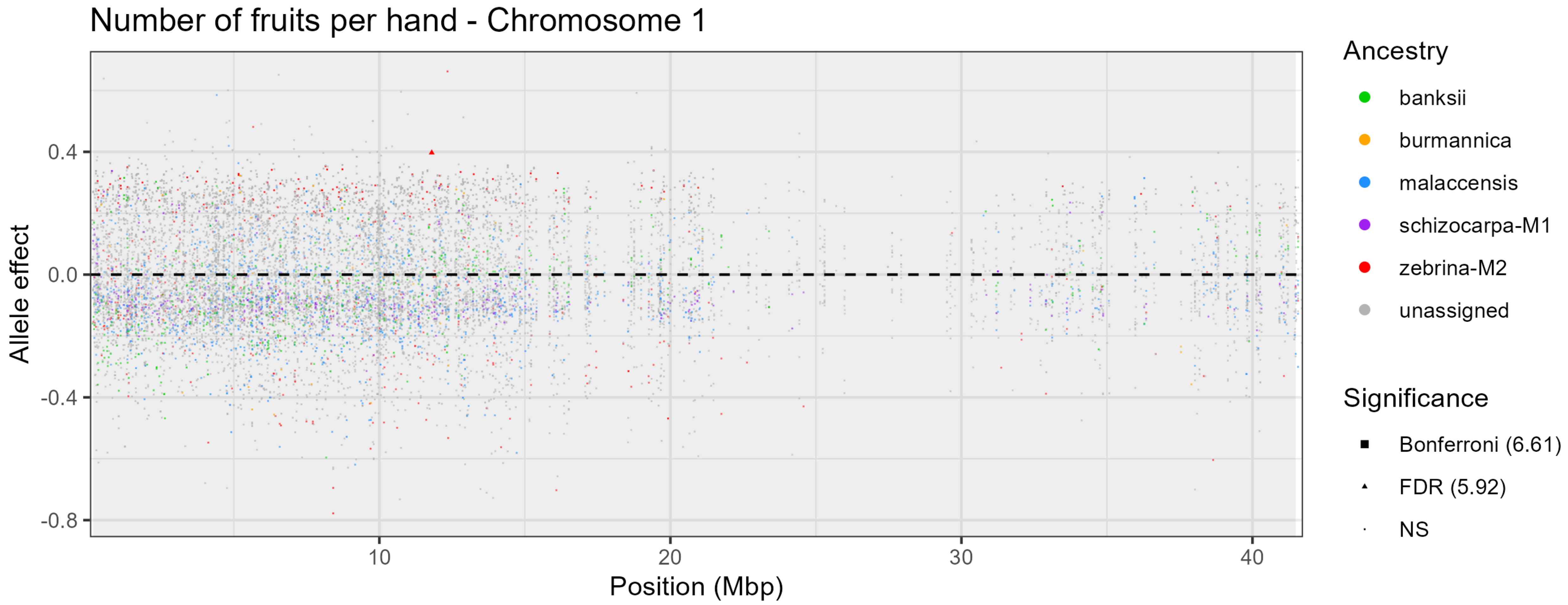

Figure S5AU: Estimated allele effects along chromosome 1 for number of fruits per hand obtained using the Kc model. Dots are colored according to allele ancestry and shaped according to the level of significance of the test. When no ancestry could be assigned, the effect represented is that of the alternative allele. The QTL interval considered is indicated by a gray area.

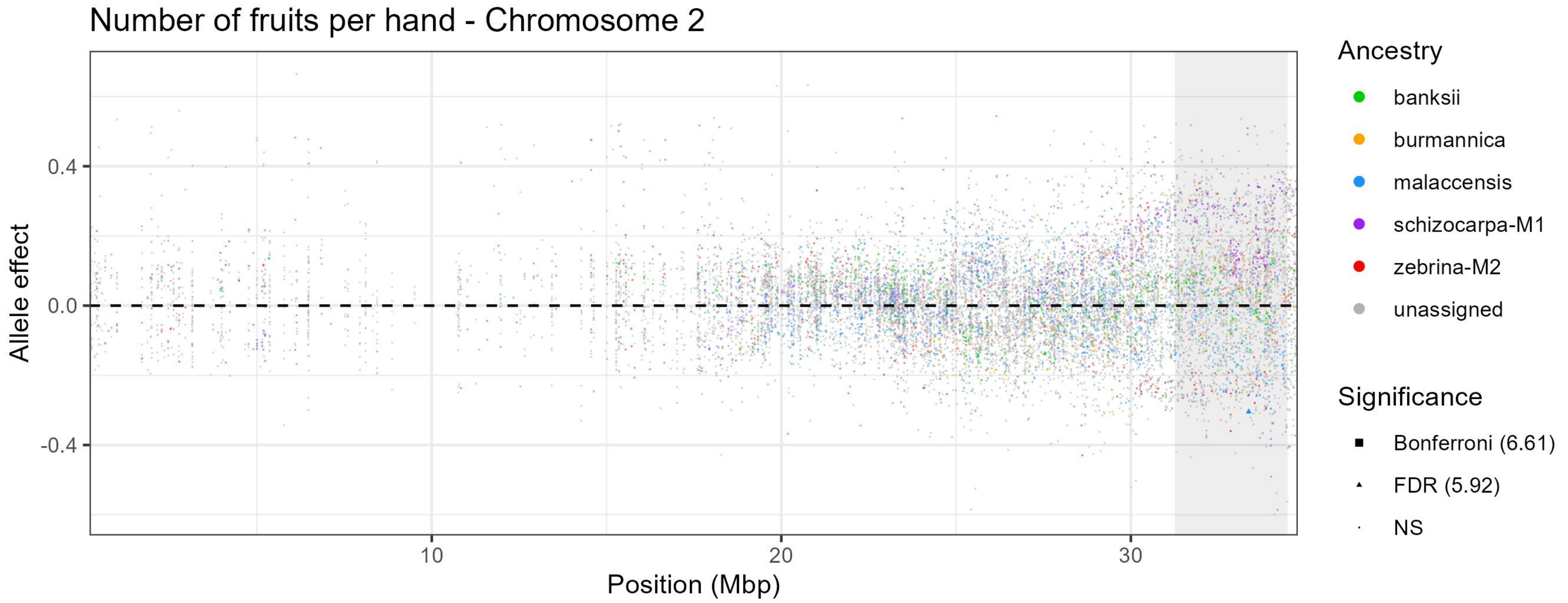

Figure S5AV: Estimated allele effects along chromosome 2 for number of fruits per hand obtained using the Kc model. Dots are colored according to allele ancestry and shaped according to the level of significance of the test. When no ancestry could be assigned, the effect represented is that of the alternative allele. The QTL interval considered is indicated by a gray area.

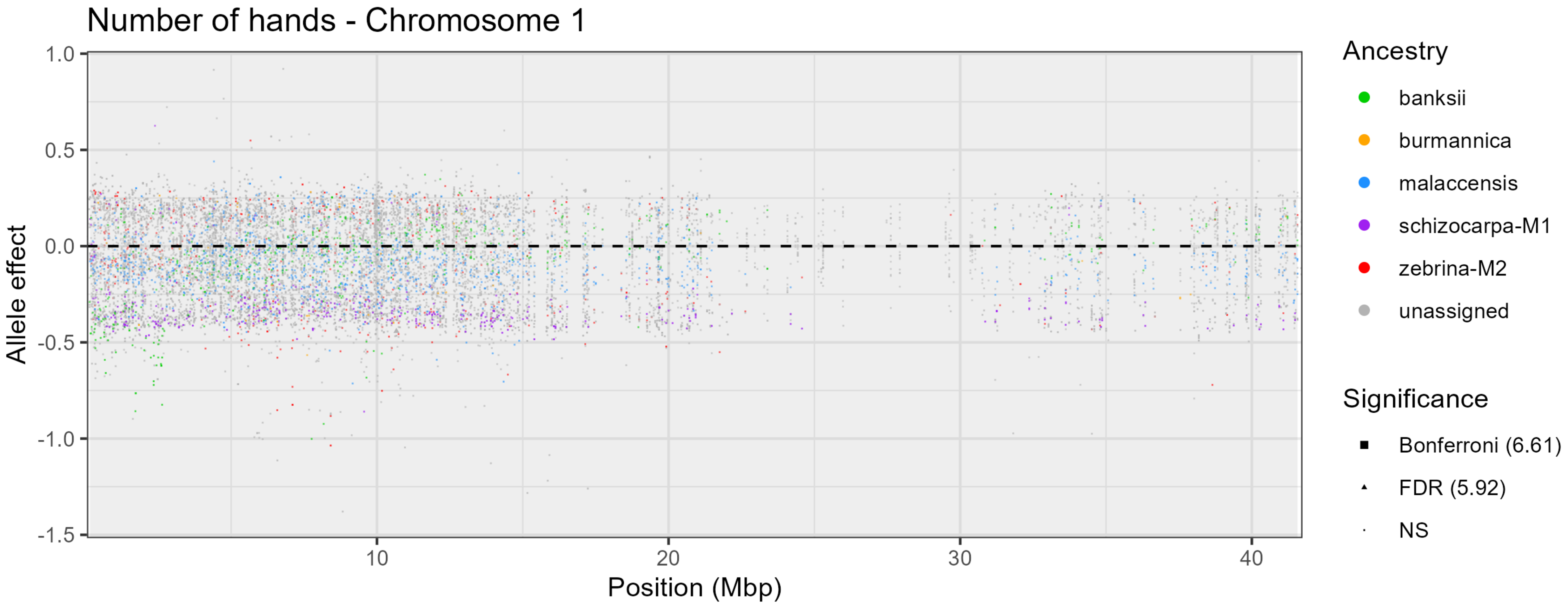

Figure S5AW: Estimated allele effects along chromosome 1 for number of hands obtained using the Kc model. Dots are colored according to allele ancestry and shaped according to the level of significance of the test. When no ancestry could be assigned, the effect represented is that of the alternative allele. The QTL interval considered is indicated by a gray area.

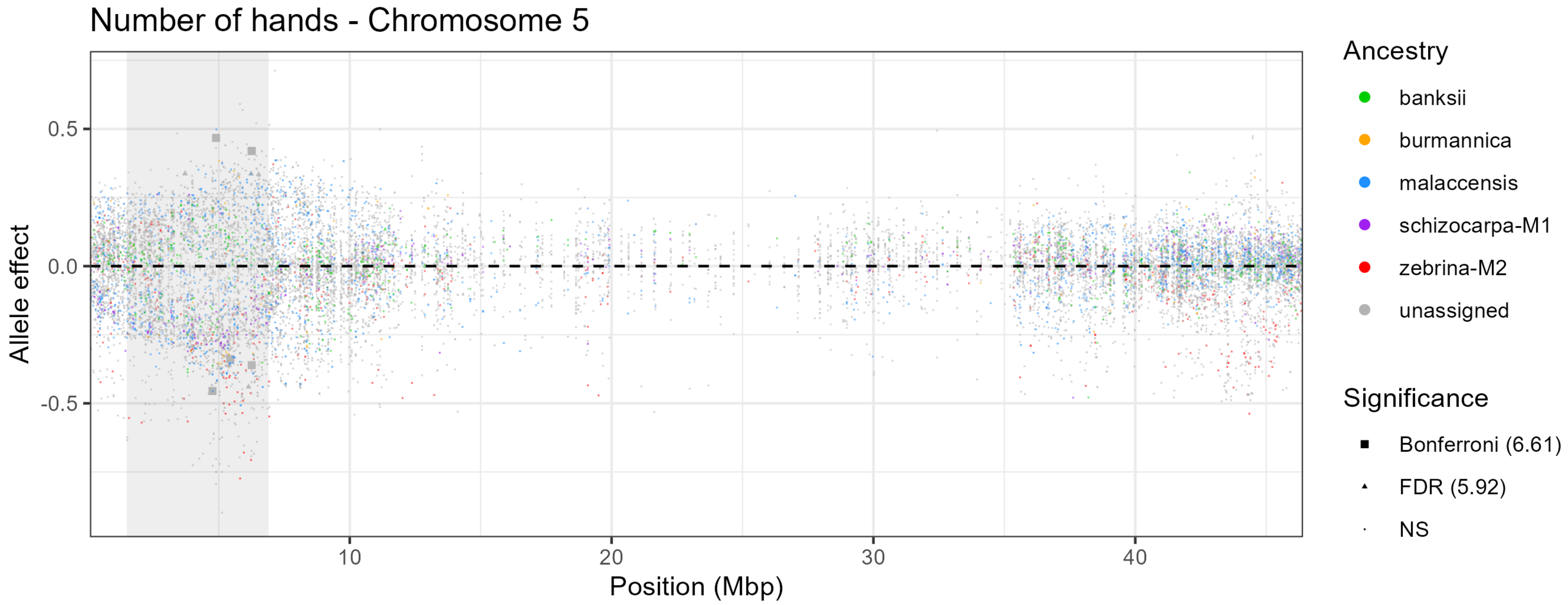

Figure S5AX: Estimated allele effects along chromosome 5 for number of hands obtained using the Kc model. Dots are colored according to allele ancestry and shaped according to the level of significance of the test. When no ancestry could be assigned, the effect represented is that of the alternative allele. The QTL interval considered is indicated by a gray area.

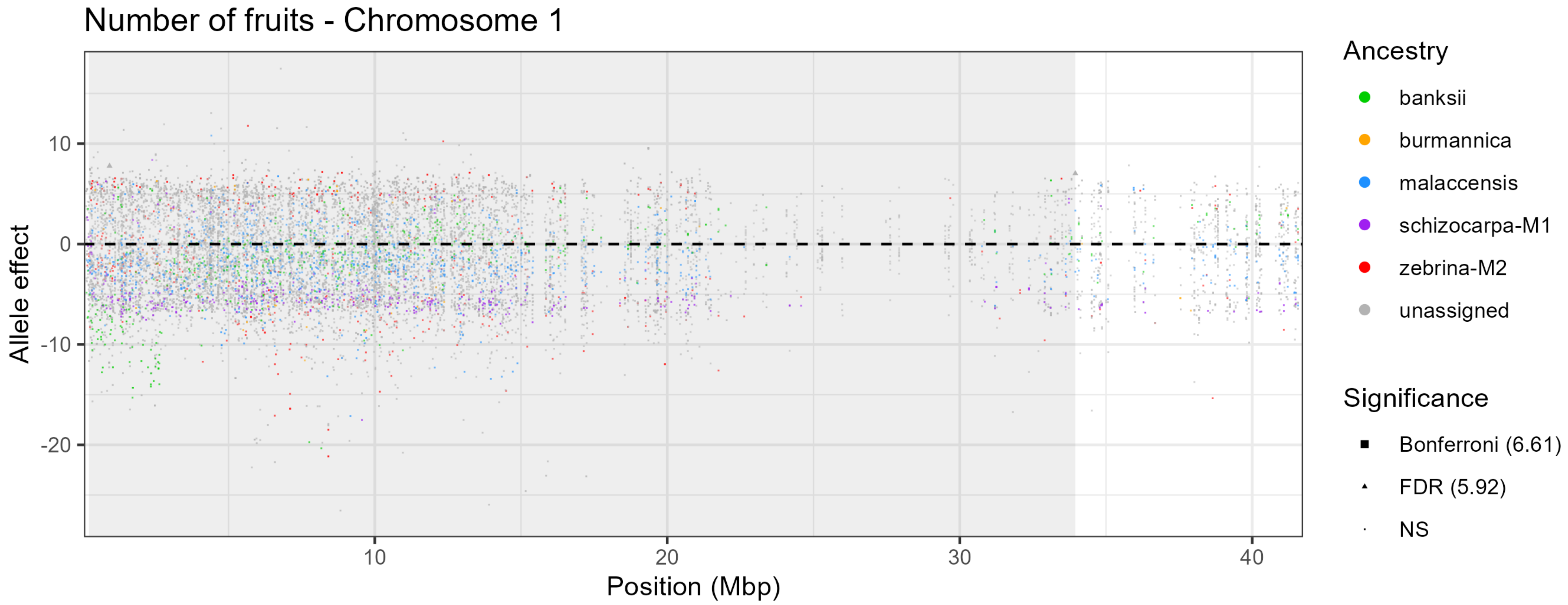

Figure S5AY: Estimated allele effects along chromosome 1 for number of fruits obtained using the Kc model. Dots are colored according to allele ancestry and shaped according to the level of significance of the test. When no ancestry could be assigned, the effect represented is that of the alternative allele. The QTL interval considered is indicated by a gray area.

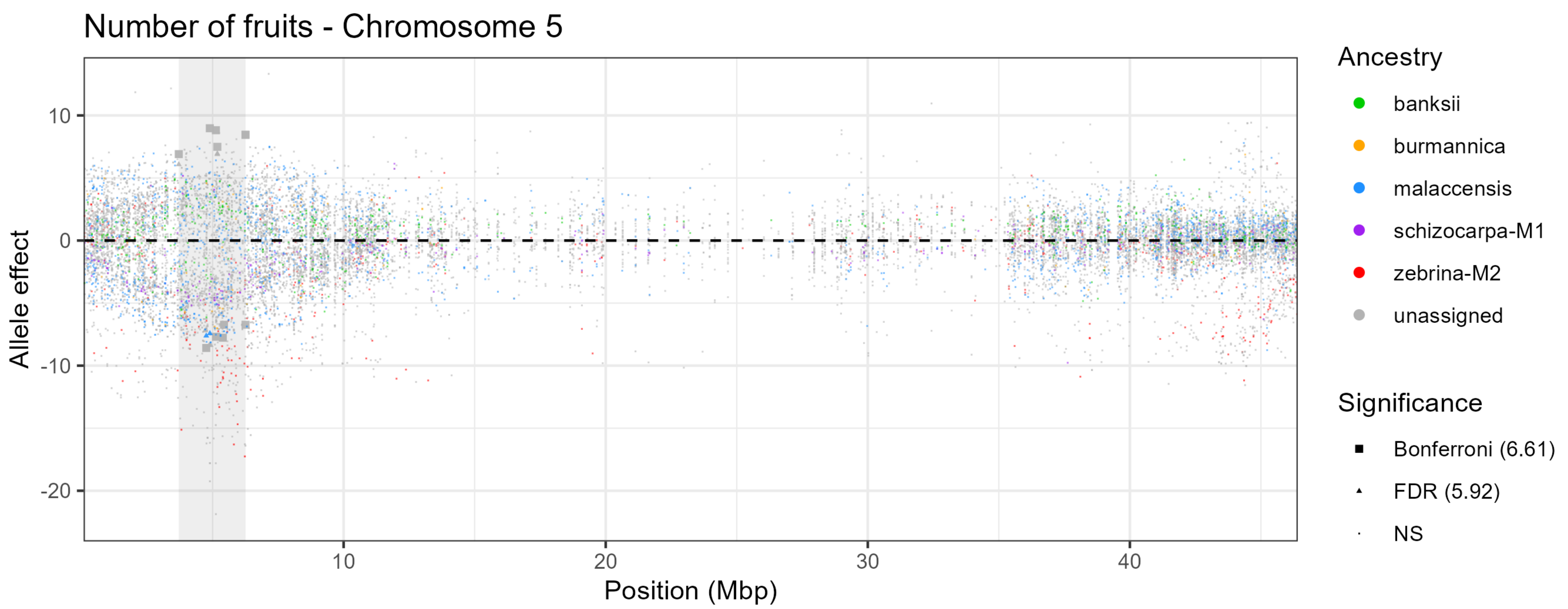

Figure S5AZ: Estimated allele effects along chromosome 5 for number of fruits obtained using the Kc model. Dots are colored according to allele ancestry and shaped according to the level of significance of the test. When no ancestry could be assigned, the effect represented is that of the alternative allele. The QTL interval considered is indicated by a gray area.

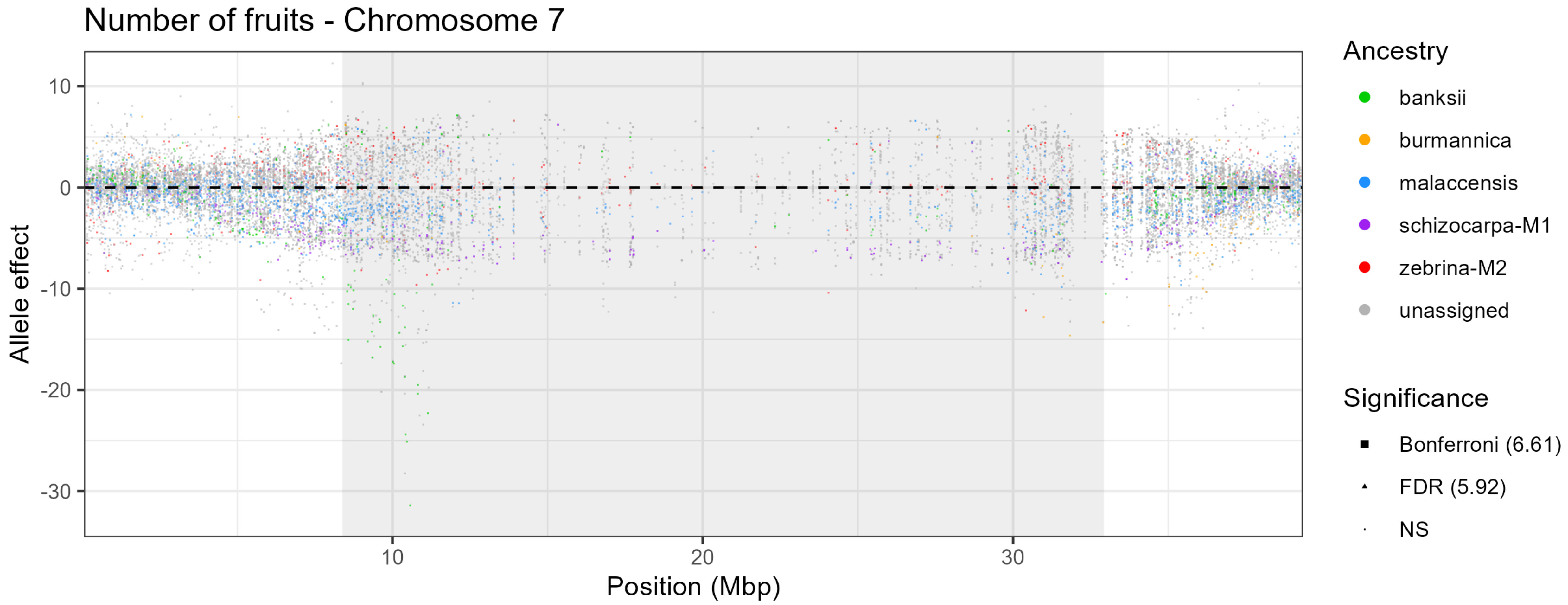

Figure S5BA: Estimated allele effects along chromosome 7 for number of fruits obtained using the Kc model. Dots are colored according to allele ancestry and shaped according to the level of significance of the test. When no ancestry could be assigned, the effect represented is that of the alternative allele. The QTL interval considered is indicated by a gray area.

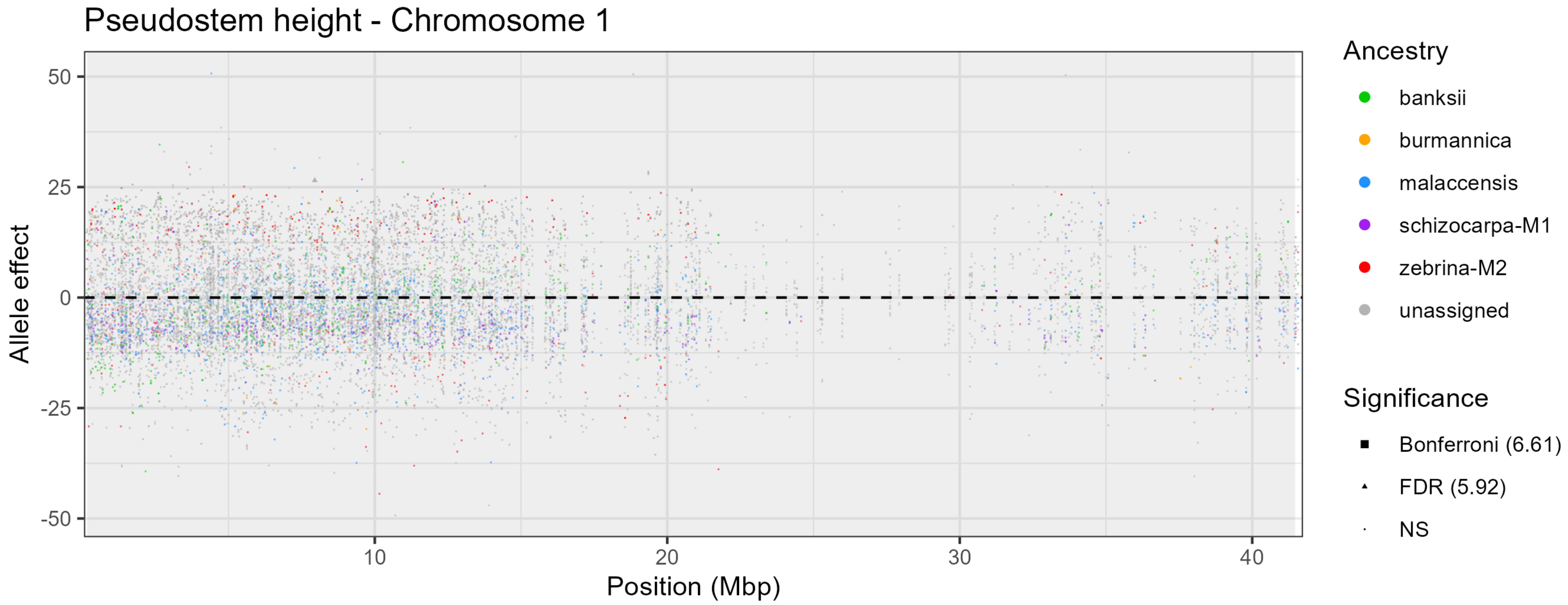

Figure S5BB: Estimated allele effects along chromosome 1 for pseudostem height obtained using the Kc model. Dots are colored according to allele ancestry and shaped according to the level of significance of the test. When no ancestry could be assigned, the effect represented is that of the alternative allele. The QTL interval considered is indicated by a gray area.

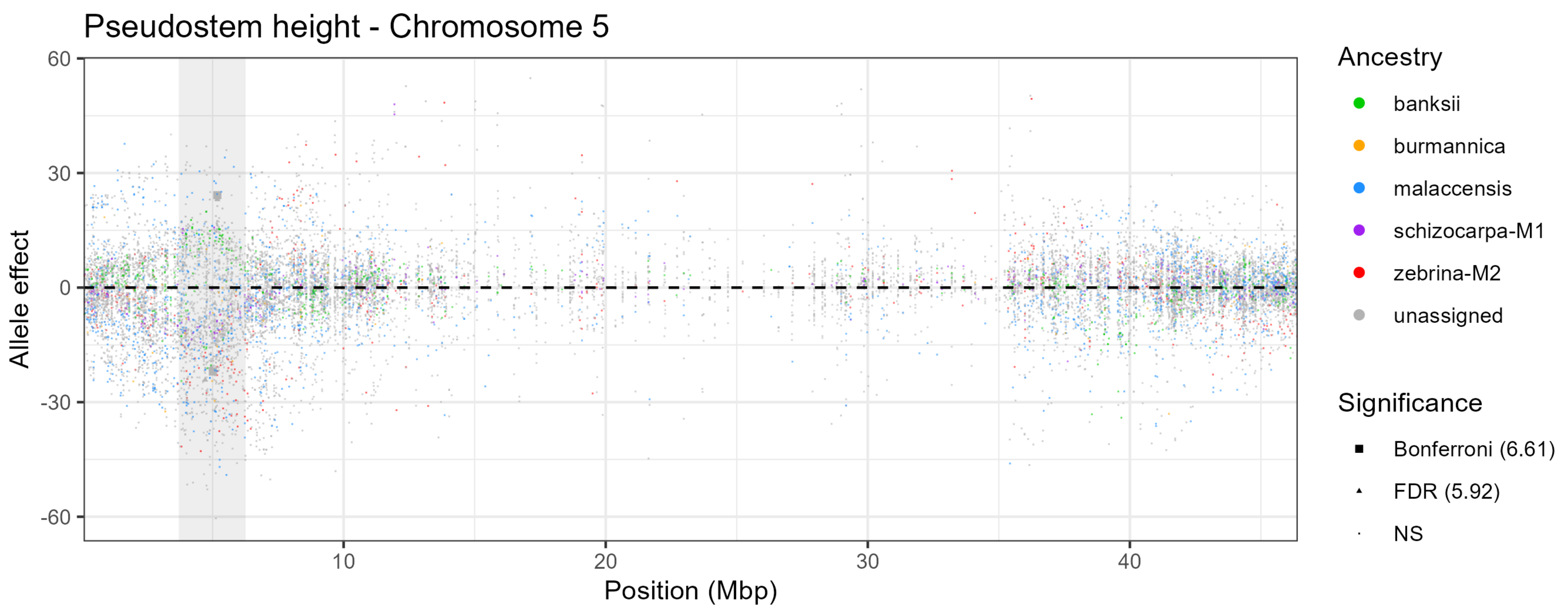

Figure S5BC: Estimated allele effects along chromosome 5 for pseudostem height obtained using the Kc model. Dots are colored according to allele ancestry and shaped according to the level of significance of the test. When no ancestry could be assigned, the effect represented is that of the alternative allele. The QTL interval considered is indicated by a gray area.

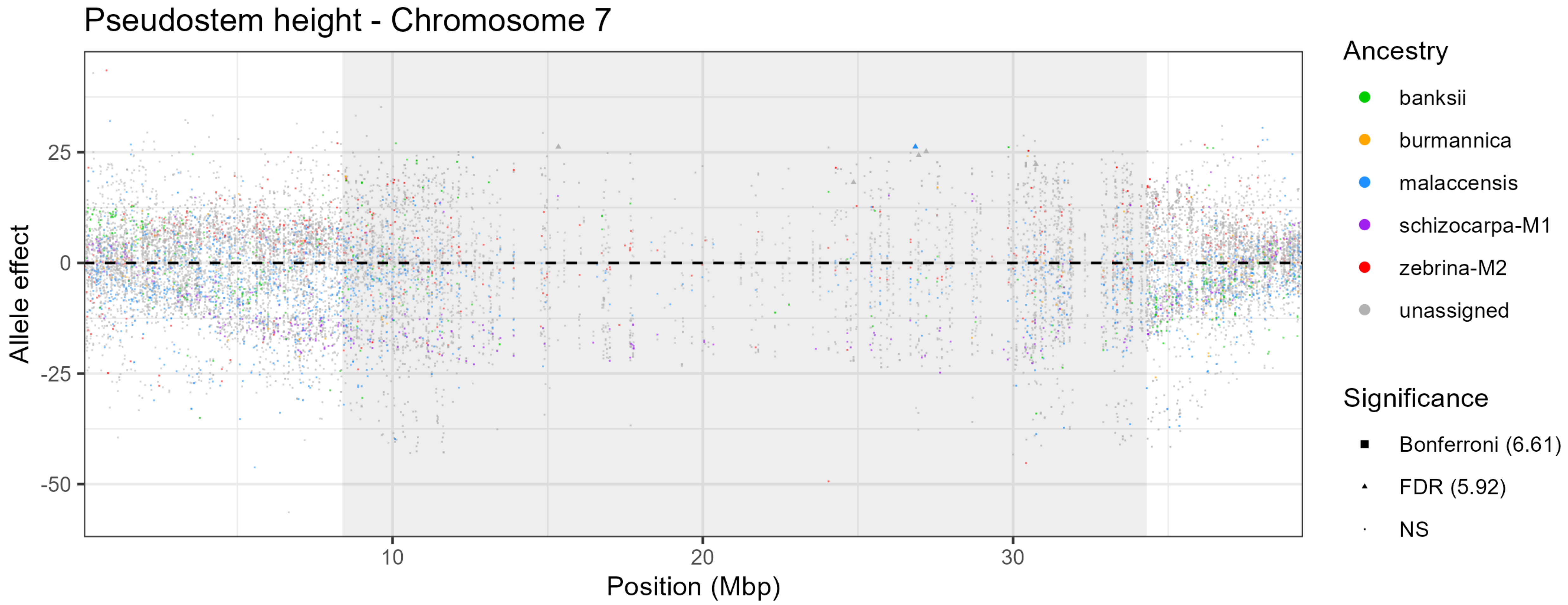

Figure S5BD: Estimated allele effects along chromosome 7 for pseudostem height obtained using the Kc model. Dots are colored according to allele ancestry and shaped according to the level of significance of the test. When no ancestry could be assigned, the effect represented is that of the alternative allele. The QTL interval considered is indicated by a gray area.

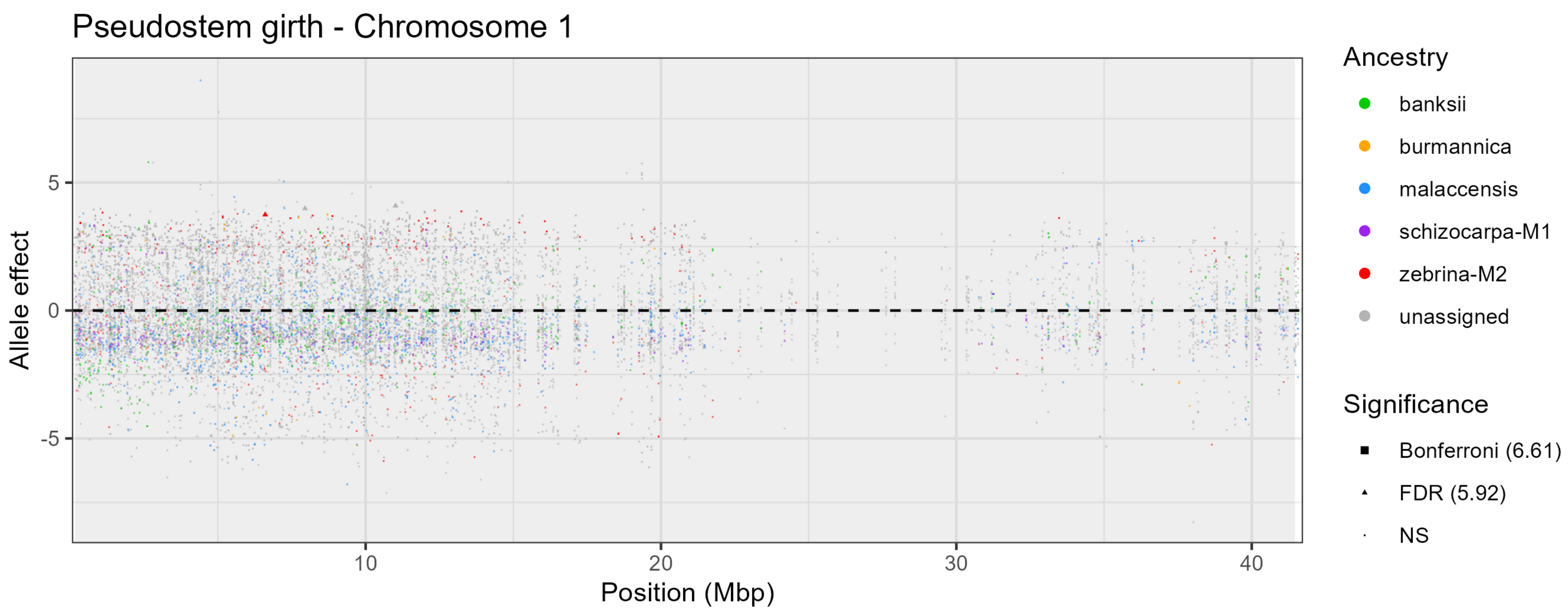

Figure S5BE: Estimated allele effects along chromosome 1 for pseudostem girth obtained using the Kc model. Dots are colored according to allele ancestry and shaped according to the level of significance of the test. When no ancestry could be assigned, the effect represented is that of the alternative allele. The QTL interval considered is indicated by a gray area.

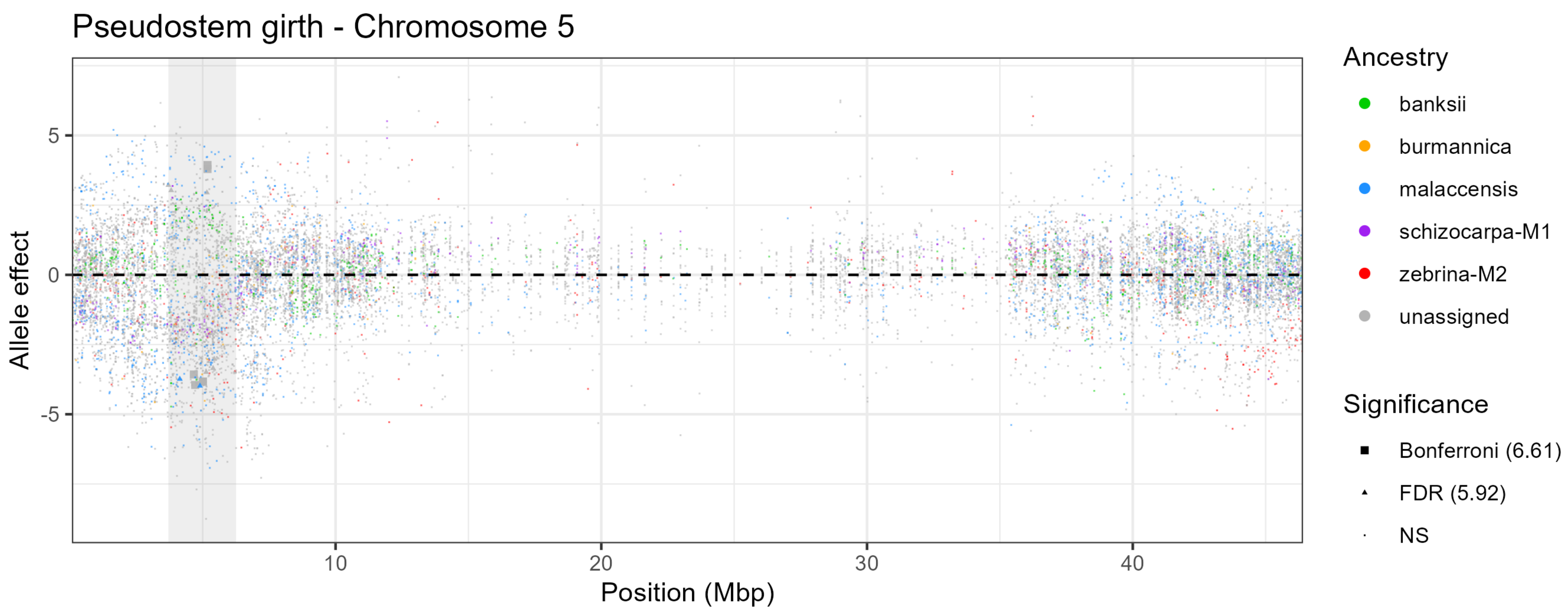

Figure S5BF: Estimated allele effects along chromosome 5 for pseudostem girth obtained using the Kc model. Dots are colored according to allele ancestry and shaped according to the level of significance of the test. When no ancestry could be assigned, the effect represented is that of the alternative allele. The QTL interval considered is indicated by a gray area.

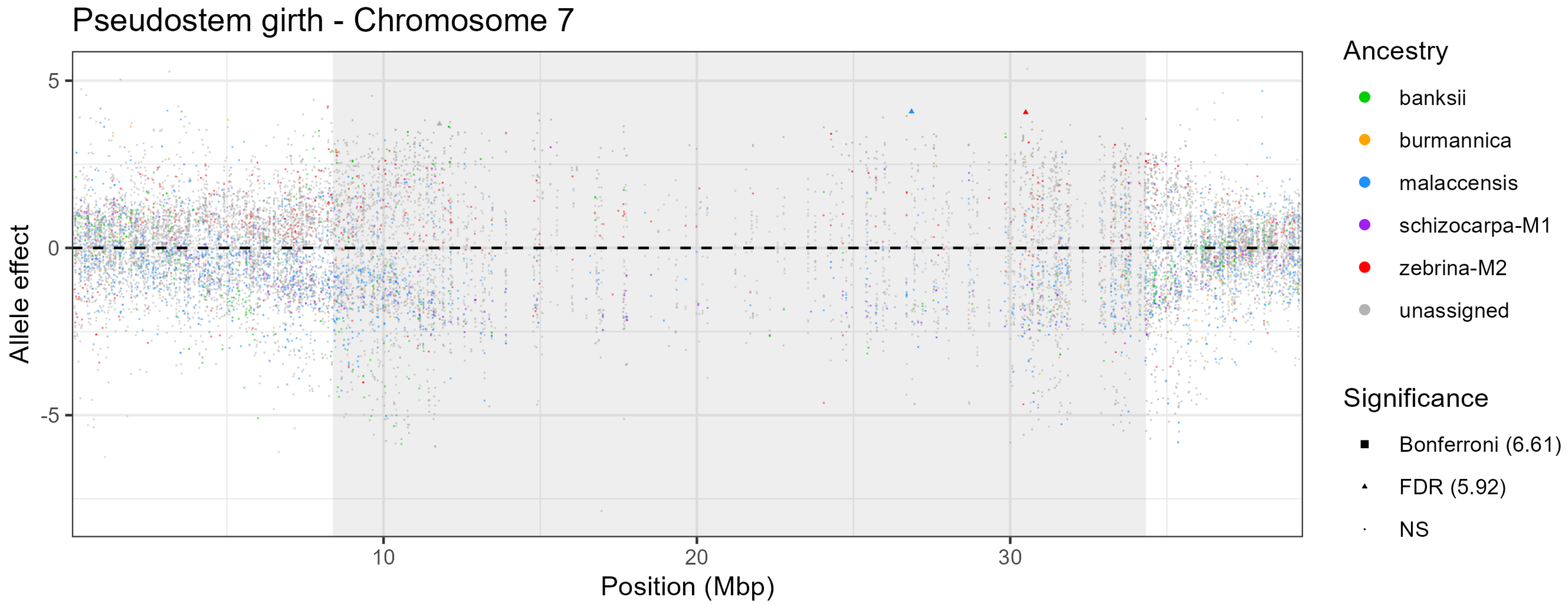

Figure S5BG: Estimated allele effects along chromosome 7 for pseudostem girth obtained using the Kc model. Dots are colored according to allele ancestry and shaped according to the level of significance of the test. When no ancestry could be assigned, the effect represented is that of the alternative allele. The QTL interval considered is indicated by a gray area.

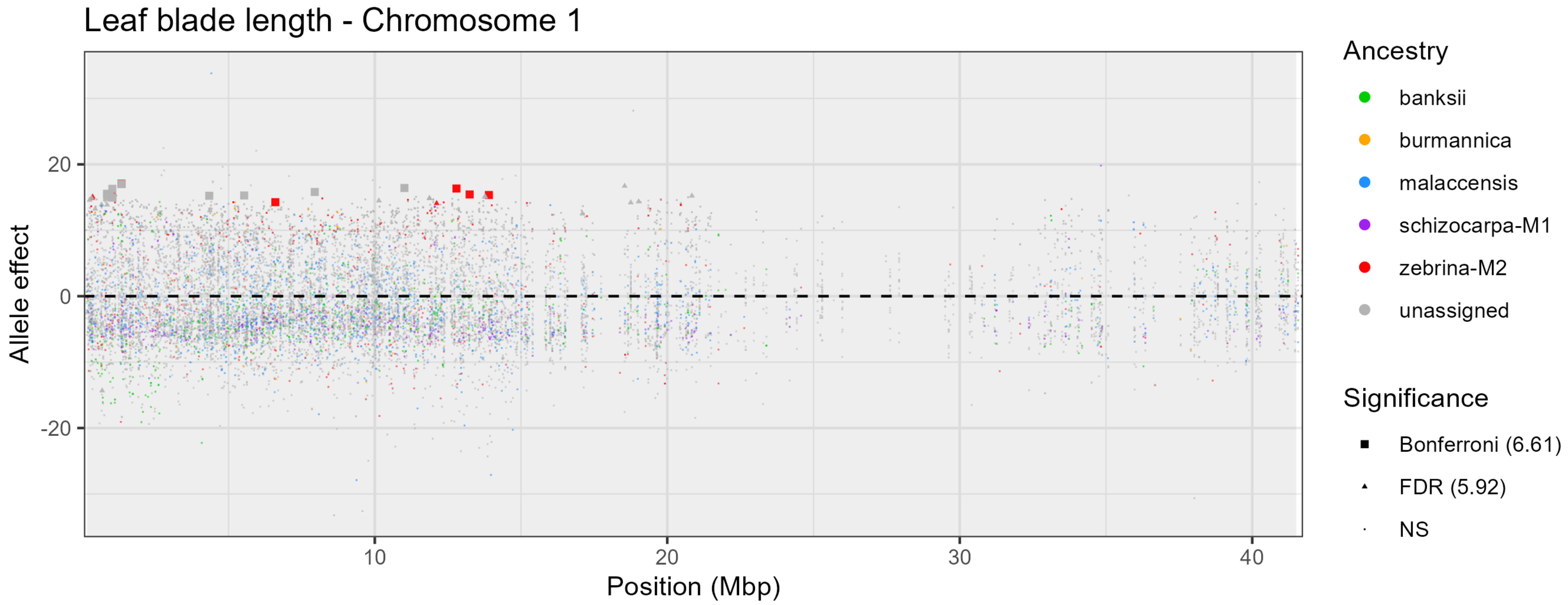

Figure S5BH: Estimated allele effects along chromosome 1 for leaf blade length obtained using the Kc model. Dots are colored according to allele ancestry and shaped according to the level of significance of the test. When no ancestry could be assigned, the effect represented is that of the alternative allele. The QTL interval considered is indicated by a gray area.

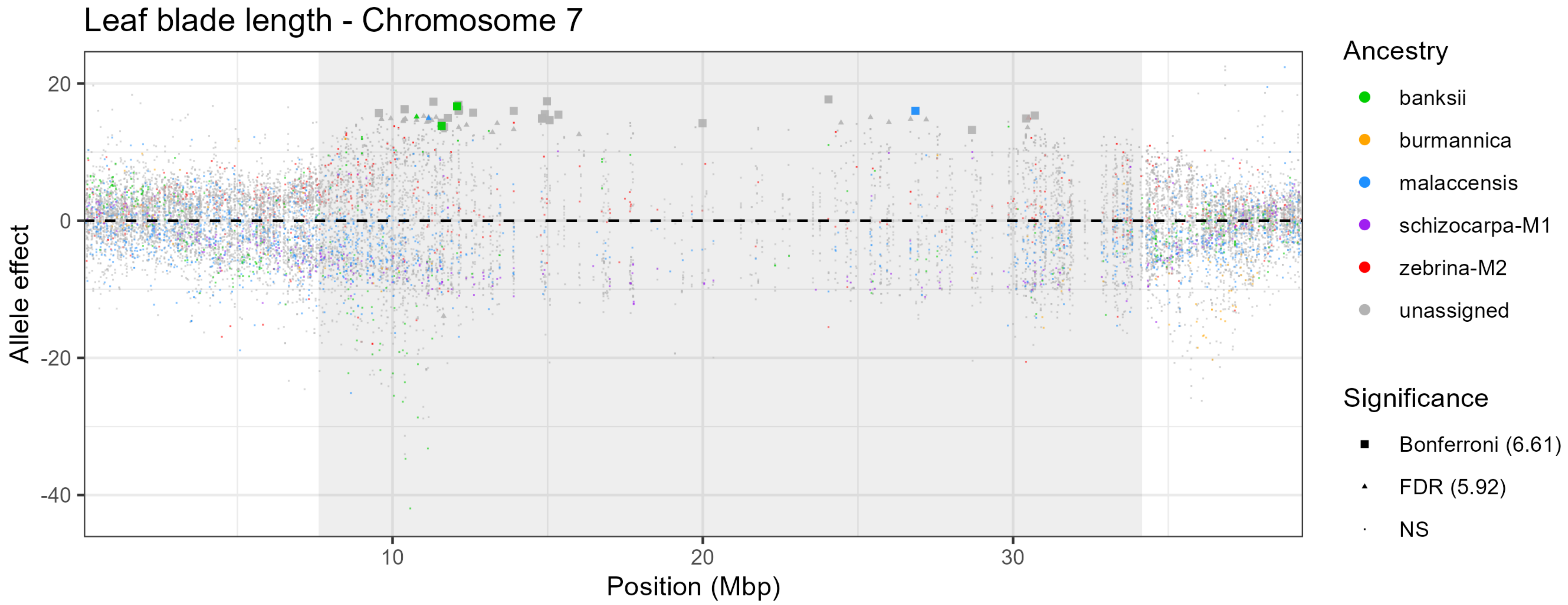

Figure S5BI: Estimated allele effects along chromosome 7 for leaf blade length obtained using the Kc model. Dots are colored according to allele ancestry and shaped according to the level of significance of the test. When no ancestry could be assigned, the effect represented is that of the alternative allele. The QTL interval considered is indicated by a gray area.

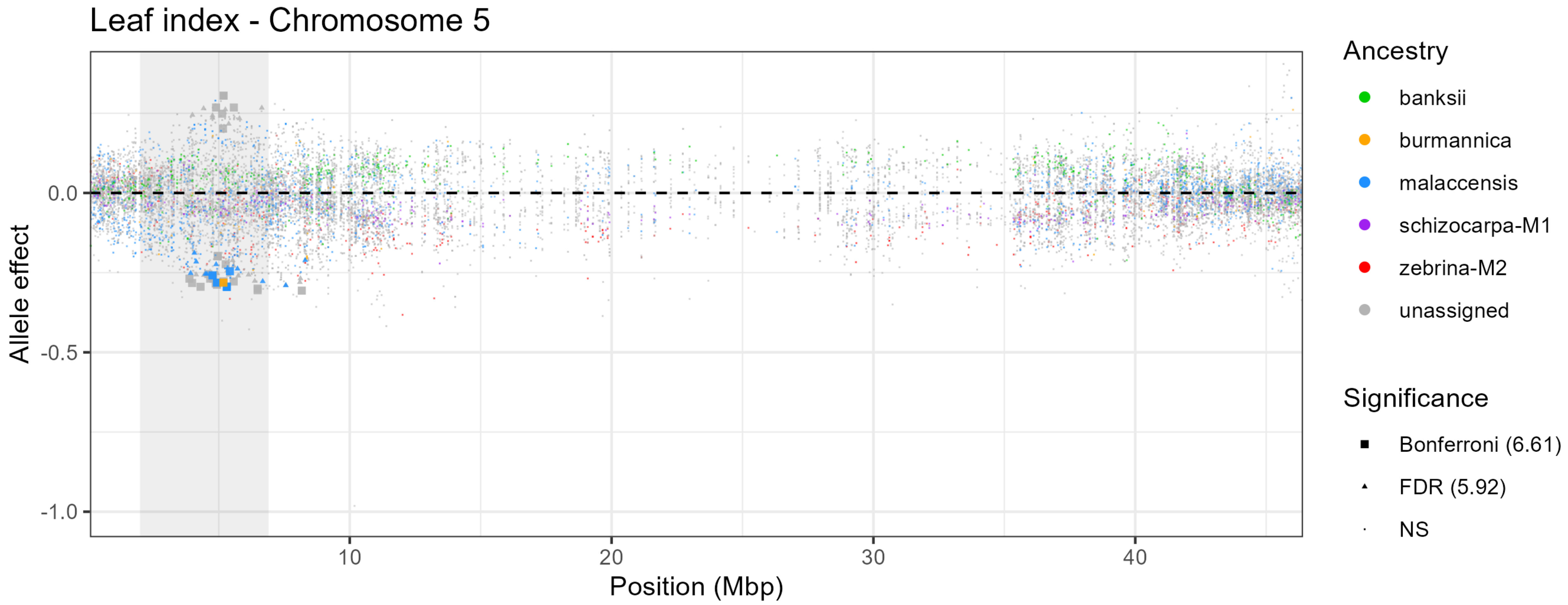

Figure S5BJ: Estimated allele effects along chromosome 5 for leaf index obtained using the Kc model. Dots are colored according to allele ancestry and shaped according to the level of significance of the test. When no ancestry could be assigned, the effect represented is that of the alternative allele. The QTL interval considered is indicated by a gray area.

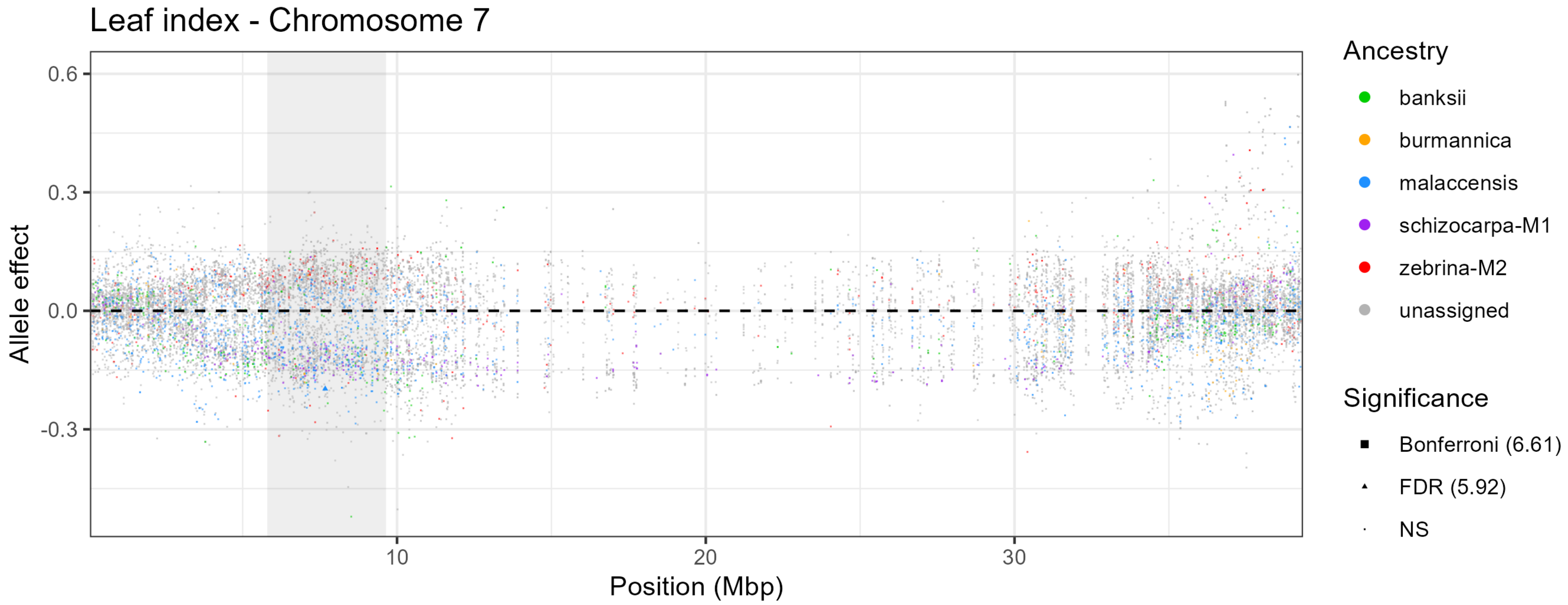

Figure S5BK: Estimated allele effects along chromosome 7 for leaf index obtained using the Kc model. Dots are colored according to allele ancestry and shaped according to the level of significance of the test. When no ancestry could be assigned, the effect represented is that of the alternative allele. The QTL interval considered is indicated by a gray area.

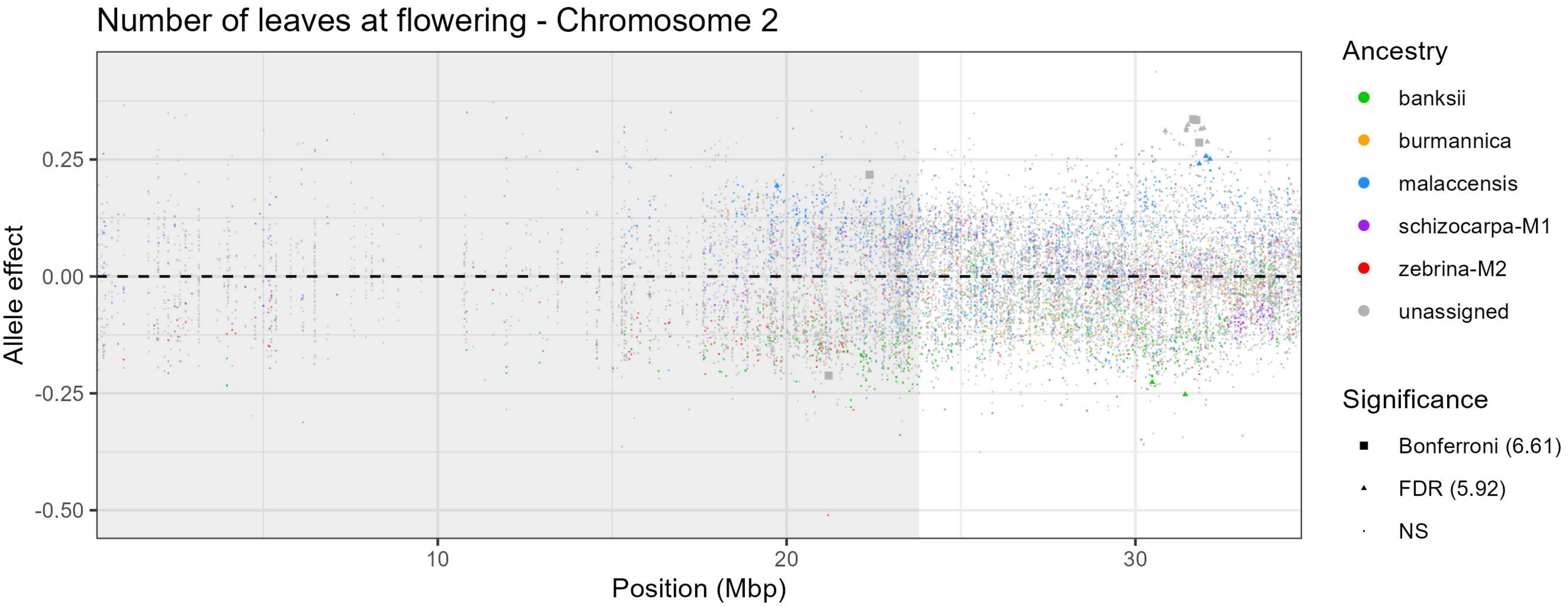

Figure S5BL: Estimated allele effects along chromosome 2 for number of leaves at flowering obtained using the Kc model. Dots are colored according to allele ancestry and shaped according to the level of significance of the test. When no ancestry could be assigned, the effect represented is that of the alternative allele. The QTL interval considered is indicated by a gray area.

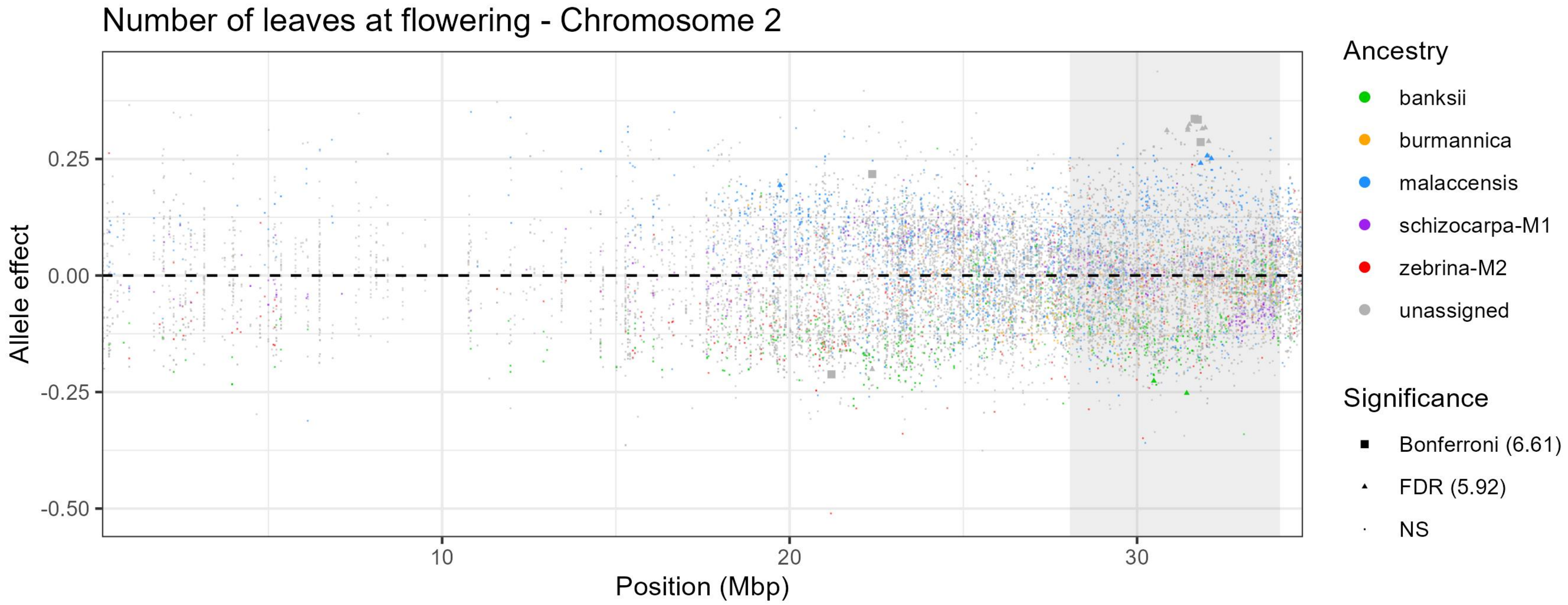

Figure S5BM: Estimated allele effects along chromosome 2 for number of leaves at flowering obtained using the Kc model. Dots are colored according to allele ancestry and shaped according to the level of significance of the test. When no ancestry could be assigned, the effect represented is that of the alternative allele. The QTL interval considered is indicated by a gray area.

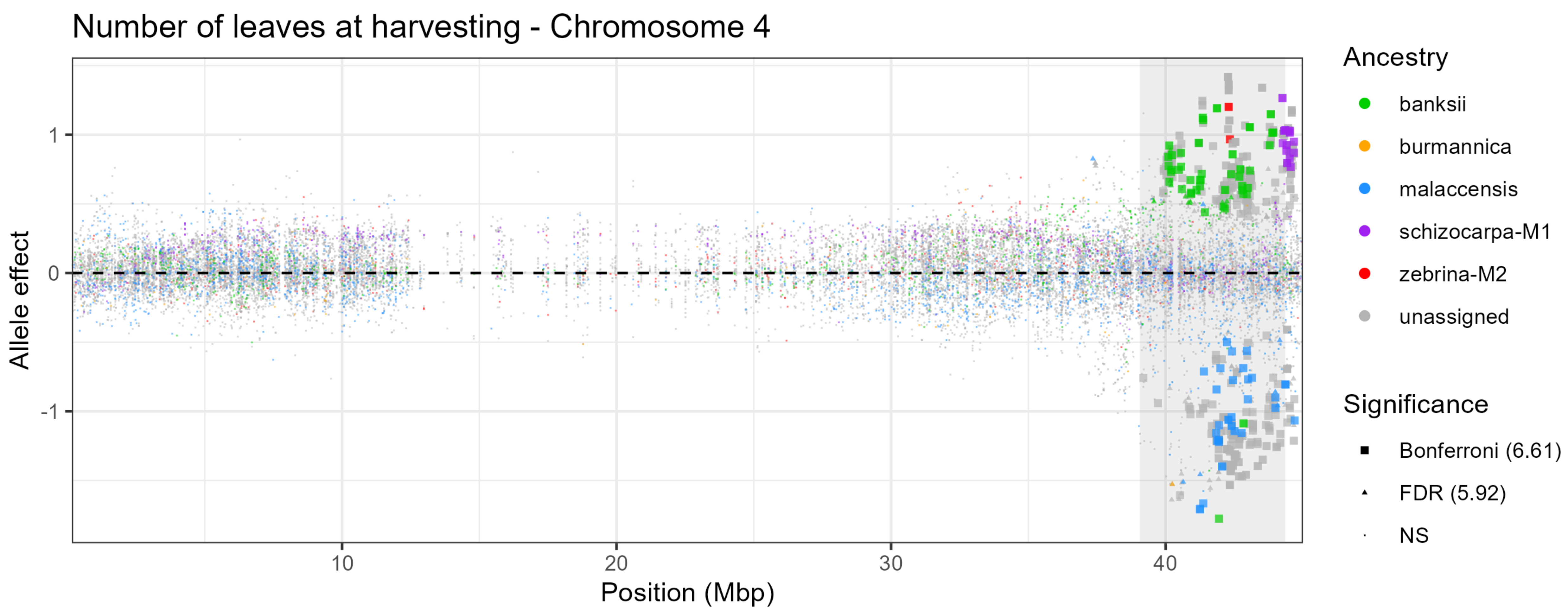

Figure S5BN: Estimated allele effects along chromosome 4 for number of leaves at harvesting obtained using the Kc model. Dots are colored according to allele ancestry and shaped according to the level of significance of the test. When no ancestry could be assigned, the effect represented is that of the alternative allele. The QTL interval considered is indicated by a gray area.

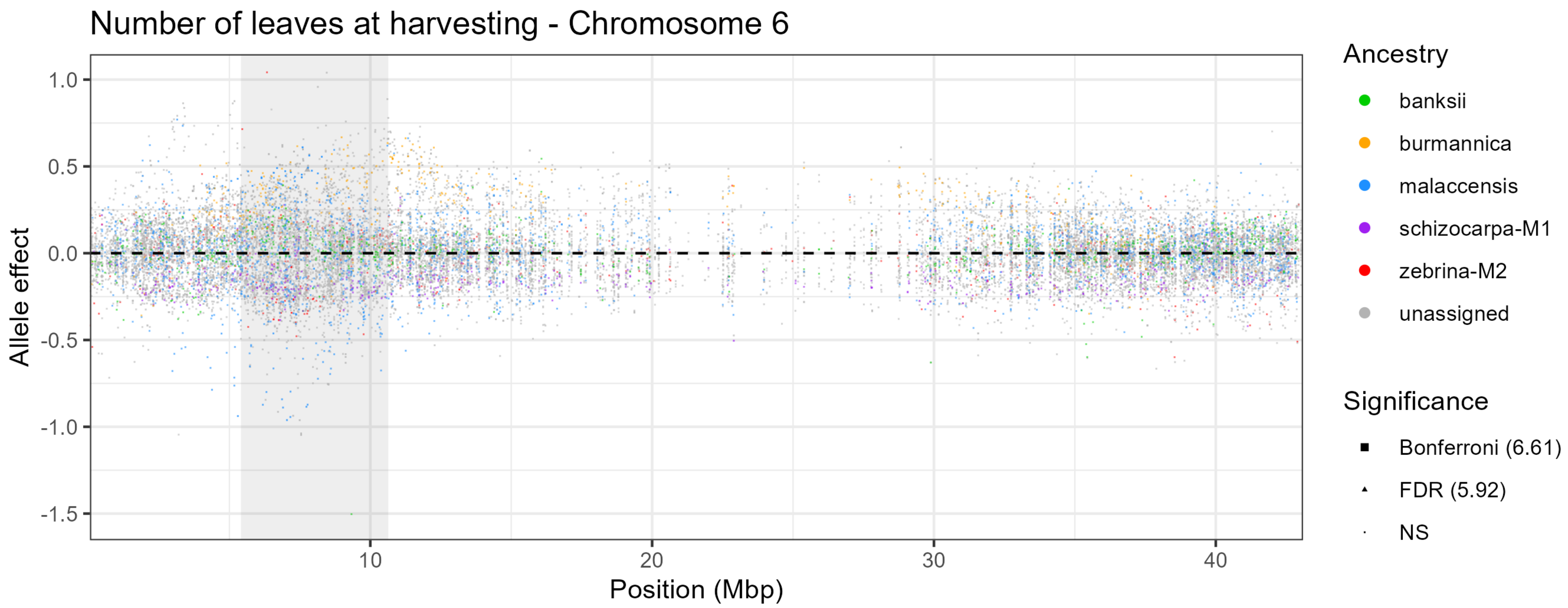

Figure S5BO: Estimated allele effects along chromosome 6 for number of leaves at harvesting obtained using the Kc model. Dots are colored according to allele ancestry and shaped according to the level of significance of the test. When no ancestry could be assigned, the effect represented is that of the alternative allele. The QTL interval considered is indicated by a gray area.

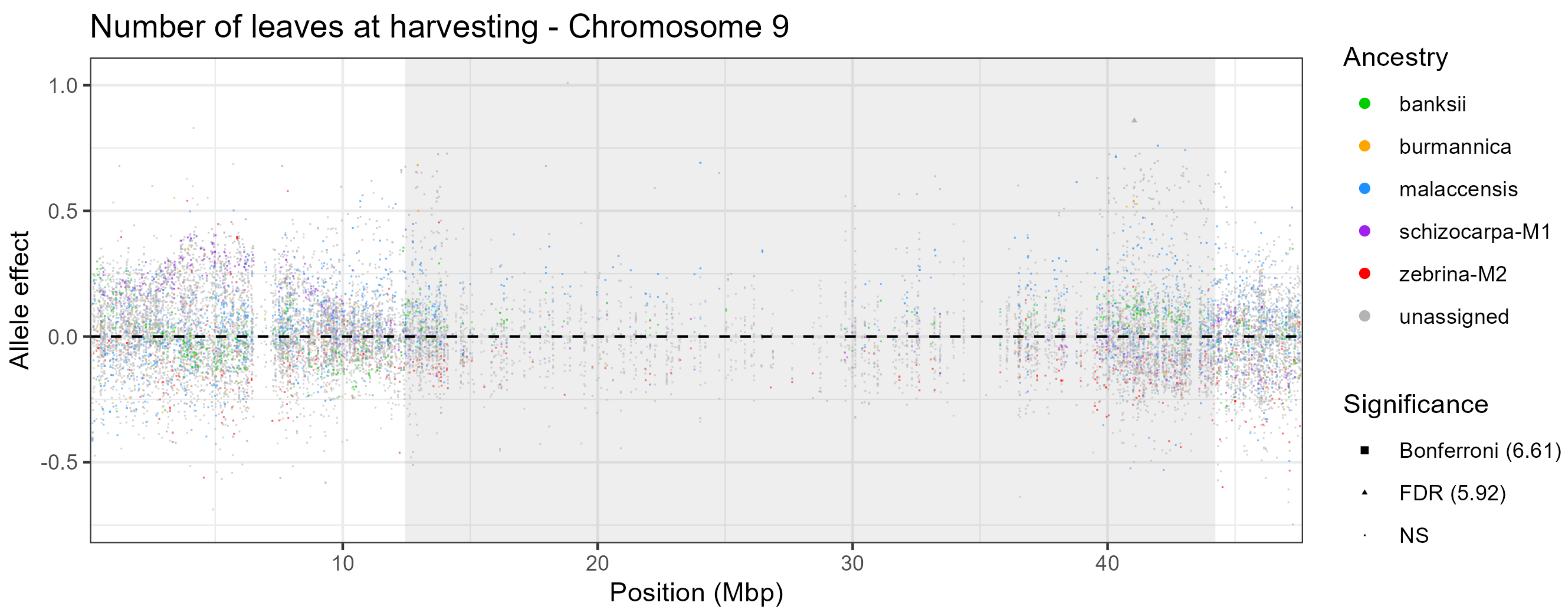

Figure S5BP: Estimated allele effects along chromosome 9 for number of leaves at harvesting obtained using the Kc model. Dots are colored according to allele ancestry and shaped according to the level of significance of the test. When no ancestry could be assigned, the effect represented is that of the alternative allele. The QTL interval considered is indicated by a gray area.

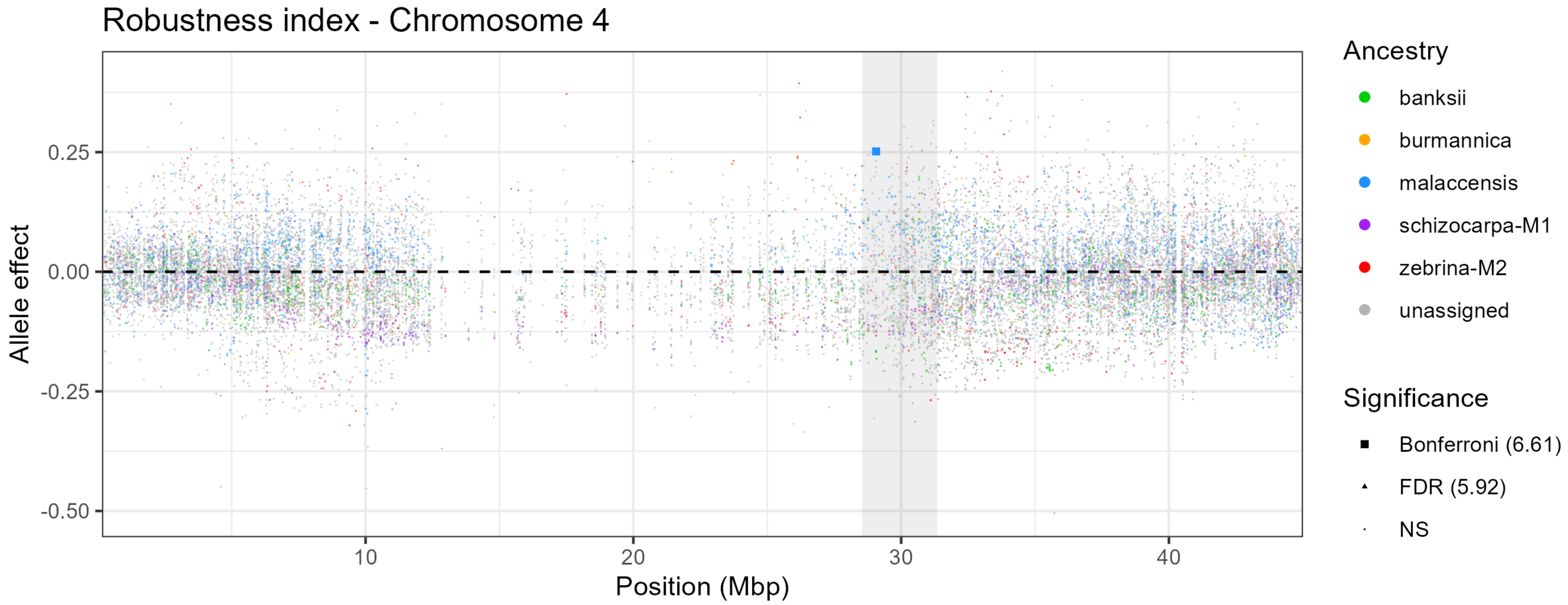

Figure S5BQ: Estimated allele effects along chromosome 4 for robustness index obtained using the Kc model. Dots are colored according to allele ancestry and shaped according to the level of significance of the test. When no ancestry could be assigned, the effect represented is that of the alternative allele. The QTL interval considered is indicated by a gray area.
